# Long-Term Changes in the Distributions of Larval and Adult Fish in the Northeast U.S. Shelf Ecosystem

**DOI:** 10.1371/journal.pone.0137382

**Published:** 2015-09-23

**Authors:** Harvey J. Walsh, David E. Richardson, Katrin E. Marancik, Jonathan A. Hare

**Affiliations:** 1 National Oceanic and Atmospheric Administration, National Marine Fisheries Service, Northeast Fisheries Science Center, Narragansett, Rhode Island, United States of America; 2 Integrated Statistics, Narragansett, Rhode Island, United States of America; Department of Agriculture, AUSTRALIA

## Abstract

Many studies have documented long-term changes in adult marine fish distributions and linked these changes to climate change and multi-decadal climate variability. Most marine fish, however, have complex life histories with morphologically distinct stages, which use different habitats. Shifts in distribution of one stage may affect the connectivity between life stages and thereby impact population processes including spawning and recruitment. Specifically, many marine fish species have a planktonic larval stage, which lasts from weeks to months. We compared the spatial distribution and seasonal occurrence of larval fish in the Northeast U.S. Shelf Ecosystem to test whether spatial and temporal distributions changed between two decades. Two large-scale ichthyoplankton programs sampled using similar methods and spatial domain each decade. Adult distributions from a long-term bottom trawl survey over the same time period and spatial area were also analyzed using the same analytical framework to compare changes in larval and adult distributions between the two decades. Changes in spatial distribution of larvae occurred for 43% of taxa, with shifts predominately northward (i.e., along-shelf). Timing of larval occurrence shifted for 49% of the larval taxa, with shifts evenly split between occurring earlier and later in the season. Where both larvae and adults of the same species were analyzed, 48% exhibited different shifts between larval and adult stages. Overall, these results demonstrate that larval fish distributions are changing in the ecosystem. The spatial changes are largely consistent with expectations from a changing climate. The temporal changes are more complex, indicating we need a better understanding of reproductive timing of fishes in the ecosystem. These changes may impact population productivity through changes in life history connectivity and recruitment, and add to the accumulating evidence for changes in the Northeast U.S. Shelf Ecosystem with potential to impact fisheries and other ecosystem services.

## Introduction

Numerous studies have documented changes in marine fish distributions over the past four decades, and several have attributed these changes to climate and fishing pressure. In the northern hemisphere, shifts in adult distributions are generally poleward (toward the north) or offshore to deeper water. This pattern has been observed in many of the species examined in the North Sea [1, 2], about a quarter of the species analyzed in the Bering Sea [3], and approximately half the species studied along the Northeast U.S. (NEUS) Shelf Ecosystem [4] with both fishing pressure and climate linked to the changes [5]. Other studies report similar results from other areas around the globe [6–8]. Climate and fishing pressure also have been identified as important factors forcing changes in the abundance of adult marine fish [2, 9, 10]. Changes in distribution and abundance are projected to continue in the future if waters continue to warm as a result of climate change [8, 11].

Many marine fish species have complex life histories with morphologically distinct life stages utilizing discrete habitats [12, 13]. A majority of marine fish species that reside in the NEUS Shelf Ecosystem spawn pelagic eggs, and many of those with demersal eggs (e.g., Atlantic Herring, Winter Flounder) have planktonic larvae [14, 15]. Adults typically spawn tens of thousands to millions of small eggs (< 2 mm). Eggs hatch into larvae that are planktonic and susceptible to movement by water currents [16]. After a period of time (days to months), larvae transition to a juvenile stage, which often requires distinct habitats from those used by earlier stages and by adults. After several months to years, juveniles transition to adults, completing the life cycle. There are many variations on this general theme [17], but the critical element is the same: morphologically discrete life stages often use discrete habitats. Arising from this general marine life history strategy is the need for connections between life stages and habitats; at the end of each stage, individuals need to be in a time and place suitable for accessing habitats for the next stage [18, 19].

Most studies examining changes in marine fish distributions have focused on adult stages and relatively little is known regarding changes in early life stage distributions (eggs, larvae, and juveniles) [20]. On the west coast of the United States, changes in larval distribution between 1951 and 2002 were found in approximately half of the taxa examined and shifts were related to exploitation and habitat type [21, 22]. Exploited species shifted distributions more than unexploited in response to environmental change [22] and epipelagic vertically migrating species shifted significantly poleward (i.e., northward) more frequently than non-migrating mesopelagic species [21]. Changes in timing of larval occurrence have been linked to changes in temperature in the English Channel [23] with larval occurrence shifting by 10 to 30 days depending on the species. Some species spawned earlier in the season as water temperatures warmed earlier.

Changes in adult and early life stage distributions can affect connectivity, recruitment, and population growth rates (i.e, population productivity). Larval distribution is largely determined by spawning location and advection [24, 25]. Adults of many species make large migrations to specific spawning grounds during the same season year after year [26–28], presumably to increase the chances of larval survival [19]. Much of the variability in population abundance is determined by variable survival of early life stages [16, 29, 30]. Changes in spawning location and timing could result in early life stage dispersal to inadequate habitats for continuation of the life cycle. Alternatively, consistent spawning locations and timing could interact with changing advective or thermal environments and result in larvae not arriving to suitable habitats for the continuation of the life cycle [31].

To examine larval distributions and evaluate these expectations, we used larval and adult fish data collected by NOAA Northeast Fisheries Science Center. The adult data have been collected over the scale of the Northeast U.S. Shelf Ecosystem annually since the 1960s and previous analyses demonstrated that adult distributions are shifting northward for half the species examined [4, 32]. The larval data were collected over the scale of the ecosystem in two separate programs. Other ichthyoplankton programs have been conducted in the region, but not at the scale of the entire ecosystem (see [33]). Specifically, we tested whether the distributions of larvae and adults were different between two decades: 1977–1987 and 1999–2008. The analysis was different from other studies, which examined center of biomass over time [4, 32]. Since the larval fish programs were not continuous over time, we compared distributions between the two decades over the NEUS Shelf Ecosystem. We did not designate specific stock areas in order to evaluate whether adults or larvae were shifting among stock areas.

Our goals were to 1) examine changes in the spatial distributions of larval fish in the NEUS Shelf, 2) examine changes in larval seasonal occurrence in the NEUS Shelf, 3) compare changes in larval distributions to changes in adult distributions, and 4) evaluate changing distributions relative to regional occurrence, timing of larval occurrence, management status of adults, and habitat used by adults. Based on recent meta-analyses [34], studies from other regions [21, 22] and studies in the NEUS [4, 32], we developed and compared our results to several *a priori* expectations. Waters in the NEUS Shelf Ecosystem were warming [35, 36] but warming was greater in summer than in winter [36]. Thus, we expect: 1a) for species that predominate in the Mid-Atlantic Bight, Southern New England, and Georges Bank regions, a majority of larval distributions will shift either northwards or to deeper areas of the shelf, in conjunction with observed shifts in adults [4, 32] and consistent with observations of increasing water temperatures on the shelf [36–38]; 1b) for species that predominate in the Georges Bank and Gulf of Maine regions shifts in larval distributions will be to deeper areas of the shelf owing to increased bottom temperature, but not necessarily northward owing to complex bathymetry [39]; 2a) northward shifts will be more frequent for larvae occurring in summer than winter owing to the seasonal differences in warming [36]; 2b) alternatively, northward shifts will be more frequent for larvae occurring in winter than summer because cold water restricts species northern range limits in winter [5, 9], and northern habitat will become available as the shelf warms [36–38]; 3) timing of larval occurrence will shift later in the fall and winter as the warm season extends and will shift earlier in the spring as seasonal warming starts earlier [36]; 4) shifts in larval distribution will be comparable to shifts in adult distribution indicating coherent shifts across life stages; 5) shifts in larval distribution will be more frequent for pelagic fish than demersal fish owing to their ability to move and their relative independence from bottom habitats [40]; 6) shifts in larval distribution will be more frequent for managed fish than unmanaged fish owing to their increased sensitivity to change resulting from the combination of stressors (climate and fishing) [5].

## Materials and Methods

### Larval Collections

**Fig 1:**
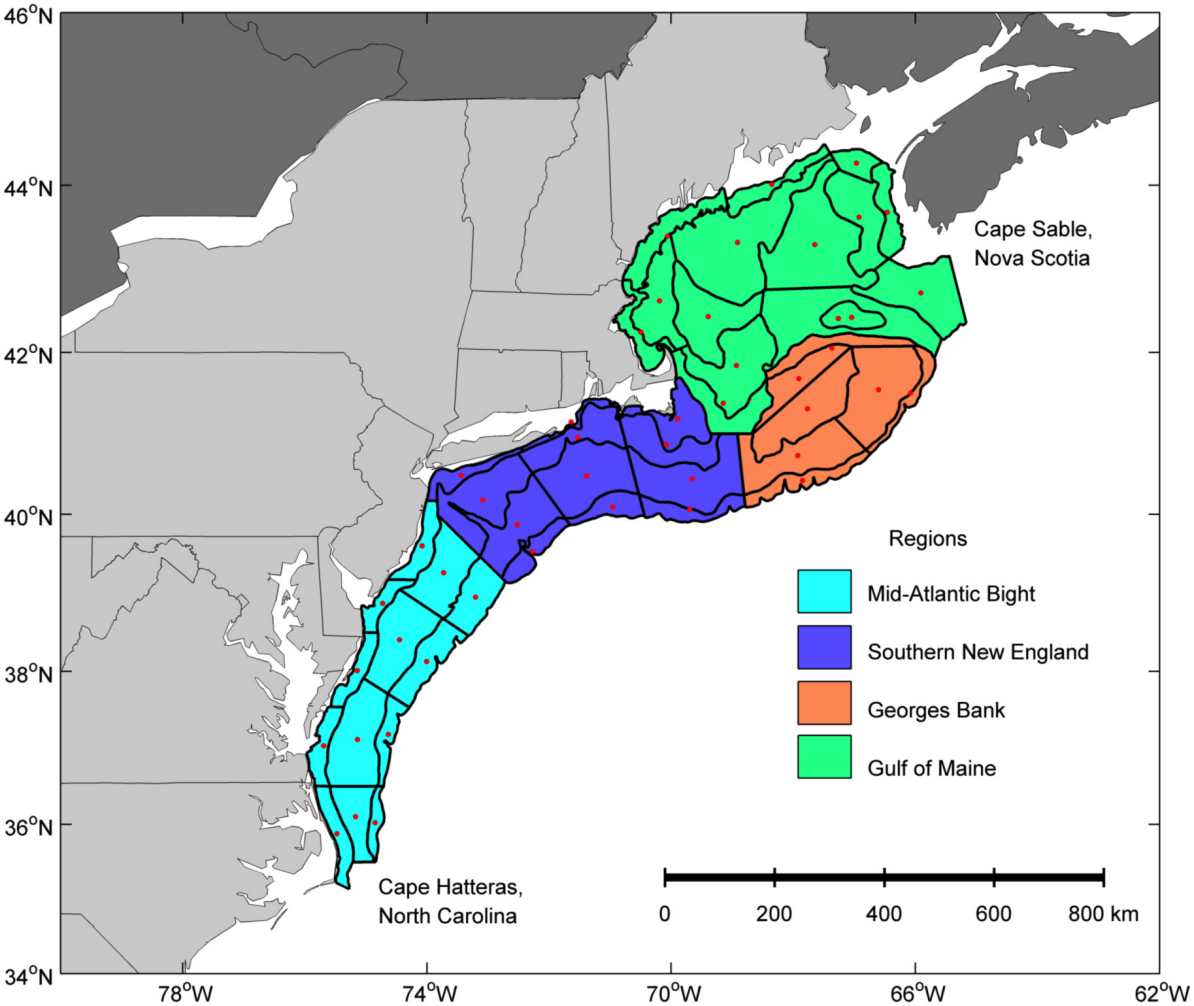
Sample strata and regions of the Northeast U.S. Shelf Ecosystem. Bottom trawl and ichthyoplankton surveys have been conducted over the past 40 years in the NEUS Shelf. Station data were grouped spatially based on the current 47 Ecosystem Monitoring strata (black polygons). The center points of each of the 47 strata (red dots) were used to estimate the distance (km) north of Cape Hatteras, North Carolina and the distance from the 200-m isobath. The strata were grouped into four regions of the shelf with similar hydrographic characteristics: Mid-Atlantic Bight (cyan), Southern New England (blue), Georges Bank (orange), and Gulf of Maine (green).

The Northeast Fisheries Science Center (NEFSC) has conducted several ichthyoplankton collection programs in the NEUS Shelf over the past forty years, with the longest duration and most complete spatial coverage from the Marine Resources Monitoring, Assessment, and Prediction program (MARMAP, 1977–1987) and Ecosystem Monitoring (EcoMon) Program (1999—present) [33]. Both MARMAP and EcoMon were designed as multi-species plankton surveys, and sampling effort covered the entire NEUS Shelf from Cape Hatteras, North Carolina, to Cape Sable, Nova Scotia (Fig 1), four to eight times per year [33]. The number of days sampled per year averaged 154 (range 116 to 203) [33], and varied primarily due to ship availability and weather conditions during cruises. MARMAP used mainly a fixed station design covering the sample area of each survey approximately evenly [41, 42]. EcoMon sampled the same spatial extent of the shelf as MARMAP, but used a random-stratified design based on the NEFSC bottom trawl survey design. The number of plankton strata (n = 47) was lower than the bottom trawl survey (n = 108) as the narrow inshore stratum and the offshore shelf-break stratum of the bottom trawl survey were combined in the EcoMon plankton sampling design. The area encompassed by each stratum determined the number of samples in that stratum. The number of stations sampled during an EcoMon survey was approximately 30% less than that of MARMAP.

The basic station protocols were very similar for MARMAP and EcoMon, and provided consistency in sampling [43, 44]. Ichthyoplankton samples were collected both day and night using a 61-cm bongo net. Net tow speed was approximately 1.5 knots. Double oblique tows were a minimum of 5-minutes in duration, and fished from the surface to within 5-m of the seabed or to a maximum depth of 200-m. The volume filtered of all collections was measured with mechanical flowmeters mounted across the mouth of each net. Flowmeters were calibrated at least annually in a test-tank. Mesh size of the net differed between the programs, and was 505-μm during MARMAP and 333-μm during EcoMon.

Sample processing was very similar among the two programs [43, 44]. Processing of most samples was conducted at the Morski Instytut Rybacki in Szczecin, Poland; the remaining samples were processed at the NEFSC or the Atlantic Reference Center, St Andrews, Canada. Larvae were identified to the lowest possible taxon and enumerated for each sample. Taxon abundance for each station was standardized to number under 10 • m^-2^ sea surface [45].

### Adult Collections

The NEFSC has conducted bottom trawl surveys for juvenile and adult fish in the NEUS Shelf over the past four decades [46]. Briefly, the NEFSC conducted spring (March-April) and fall (September-October) bottom trawl surveys using a stratified random design. All fish for each species were counted and weighed. Literature values of the estimated median size at maturity (50th percentile) were used for most species to determine size at maturity (Table A in S1 File). Only mature adults were included in our analyses to examine potential changes in spawning distribution. Changes in survey sampling have occurred over the past 4 decades [47], most notably in 2009 with the introduction of a new research vessel and trawl gear. For this study we used only data prior to the vessel change.

### Analysis of Spatial Distributions

Larval and adult distributions were compared between two time periods: 1977–1987 (MARMAP, n = 11 years) and 1999–2008 (EcoMon, n = 10 years) based on annual relative proportion rather than absolute abundance using the survey-design strata and non-parametric statistics. The comparison between two discrete time periods was necessitated by the larval data not being collected continuously over the time period. Relative proportion was used to describe spatial distribution because the absolute abundance of many taxa has changed dramatically between the two time periods, often due to fishing pressure and management practices [5, 48]. Non-parametric statistics were then used to test whether relative proportion in a stratum differed between the two sampling periods.

Relative proportion of larvae was calculated yearly for each of the 47 EcoMon strata for six bi-monthly seasons (January-February, March-April, May-June, July-August, September-October, and November-December). The mean number of larvae per stratum was estimated from all tows made within a stratum in a single bi-monthly season in each year. The absolute number of larvae within the stratum was estimated by multiplying the mean abundance of larvae within a stratum in the bi-monthly season and year by stratum area (m^2^). Thus, there were six estimates of larval abundance for each stratum per year, one for each season. The relative proportion of larvae within a stratum for each bi-monthly season and year was estimated by dividing the estimated absolute number of larvae within a stratum in a single bi-monthly season and year by the sum of all strata from all six bi-monthly seasons in a given year.

Larvae were examined in three or four consecutive bi-monthly seasons based on their rank of seasonal relative proportion in the NEUS Shelf. The top three ranked seasons were examined for most taxa since the bi-monthly period of maximum yearly abundance (e.g., 1^st^ ranked: March-April) was usually bracketed by the adjoining two seasons (e.g., 2^nd^ ranked: January-February and 3^rd^ ranked: May-June). However, if the maximum season was not bracketed by the second and third ranked seasons, then four seasons were examined (e.g., 2^nd^ ranked: November-December, 1^st^ ranked: January-February, 4^th^ ranked: March-April, and 3^rd^ ranked: May-June). Thus, there were three or four estimates of larval abundance for each stratum per year, one for each bi-monthly season examined. The bi-monthly seasons examined for each taxon is shown in (S1 File).

Relative proportions of adult fish were calculated yearly for each of the 47 EcoMon strata for two seasons: spring (March-April) and fall (September-October). The EcoMon strata, which combine some trawl survey strata, were used to allow comparisons to be made among the life stages, and the larval strata were originally developed using the stratum design of the bottom trawl. The number of adults per trawl was calculated by summing the number of fish caught greater than or equal to the estimated median size at maturity based on the swept area of the trawl. All trawls completed within a stratum, year, and season were averaged, and then this mean was multiplied by the stratum area (km^2^) to estimate stratum abundance. Thus, there were two estimates of adult abundance for each stratum per year, one for each season (spring and fall). The relative proportion of adults in a stratum and year for each season was estimated by dividing the estimated number of adults within a stratum and year in a single season by the sum of all strata from the year and season.

The Durbin-Watson coefficient [49] was used to test for autocorrelation among years for each stratum. Coefficients for annual stratum proportions for each life stage and season (i.e., larvae, adults—spring collected, adults—fall collected) were calculated for each taxon for all years, and in 4.7% (463 of 9770) of the strata, the null hypothesis was rejected that the residuals were uncorrelated, using an alpha value of 0.05. In 95.3% of the strata the null hypothesis was not rejected, and thus, we did not consider autocorrelation to be a problem in the analyses.

We used a Kruskal-Wallis test to evaluate whether the relative proportion in a stratum was different between the two time periods. For larvae, each stratum was tested for each of the three or four bi-monthly seasons, with relative proportion in a stratum for year within the decadal periods treated as replicates: 1977–1987 (n = 11 years) and 1999–2008 (n = 10 years). Similarly, for adults each stratum was tested for spring and fall. To prevent including strata with rare collections of a taxon, only strata that made up at least 99% of the empirical cumulative distribution for that taxon were included in the analyses. The total number and range (south to north) of strata for each taxon and life stage is shown in (Table A in S1 File).

Changes in distribution were examined for 58 taxa (the lowest possible taxonomic level of larval identification for some taxa was genus; Tables 1 and 2). Both larval and adult life stages were examined for 27 taxa. For the remaining 32 taxa, only larvae were examined for 18 taxa and only adults were examined for 13 taxa.

**Table 1:** Change in spatial distribution and seasonal occurrence of larval fish in the Northeast U.S. Shelf Ecosystem. Distribution in the annual relative proportions among strata between 1977–1987 and 1999–2008 and among bimonthly season of larval fish was tested using Kruskal-Wallis chi-square. By examining changes in slopes using linear analyses, distributional changes were analyzed in: the along-shelf, the cross-shelf, and the depth directions. Distributional shifts were then classified into the categories: no significant change (blank), southward shift (South), northward shift (North), inshore shift (In), offshore shift (Off), shallower (Shallow), and deeper (Deep). Seasonal changes were examined for the three highest ranked bi-monthly seasons based on annual relative proportion. The resulting change was classified as no significant change (blank), earlier (E), or later (L). Spatial and seasonal shifts were examined relative to regional occurrence (M—GB = Mid-Atlantic Bight to Georges Bank, S—GOM = Southern New England to Gulf of Maine, and GB—GOM = Georges Bank to Gulf of Maine), timing of larval occurrence (W = winter, Sp = spring, Su = summer, and F = fall), management status (M = managed, N = not managed), and habitat type (D = demersal, P = pelagic).

Family	Taxon	Region	Larval Season	Managed	Habitat	Along-Shelf	Cross-Shelf	Depth	Seasonal Shift
Clupeidae	*Brevoortia tyrannus*	M—GB	F	M	P				L
	*Clupea harengus*	GB—GOM	F	M	P		Off		
Gonostomatidae	*Cyclothone* spp.	M—GB	Su	N	P				
Myctophidae	*Benthosema* spp.	M—GB	W	N	P				E
	*Ceratoscopelus maderensis*	M—GB	Su	N	P	North			
	*Diaphus* spp.	M—GB	Su	N	P				
Gadidae	*Gadus morhua*	S—GOM	W	M	D	North		Deep	
	*Melanogrammus aeglefinus*	GB—GOM	W	M	D				E
	*Pollachius virens*	GB—GOM	W	M	P		Off	Shallow	L
Lotidae	*Enchelyopus cimbrius*	S—GOM	Su	N	D	North			E
Phycidae	*Urophycis* spp.	M—GB	Su	N	D				
Merlucciidae	*Merluccius albidus*	S—GOM	Su	M	P				L
	*Merluccius bilinearis*	S—GOM	Su	M	P	North			
Lophiidae	*Lophius americanus*	M—GB	Su	M	D	North			L
Sebastidae	*Sebastes* spp.	S—GOM	Sp	M	D				
Triglidae	*Prionotus* spp.	M—GB	Su	N	D				L
Cottidae	*Myoxocephalus aenaeus*	S—GOM	Sp	N	D	North			
	*Myoxocephalus octodecemspinosus*	S—GOM	W	N	D				
Serranidae	*Centropristis striata*	M—GB	Su	M	D	North	In		L
Pomatomidae	*Pomatomus saltatrix*	M—GB	Su	M	P				
Sciaenidae	*Cynoscion regalis*	M—GB	Su	M	D				E
	*Leiostomus xanthurus*	M—GB	W	M	D				
	*Menticirrhus* spp.	M—GB	Su	M	D				L
	*Micropogonias undulatus*	M—GB	Su	M	D	North	In		
Labridae	*Tautoga onitis*	M—GB	Su	M	D				E
	*Tautogolabrus adspersus*	S—GOM	Su	N	D	North			
Stichaeidae	*Ulvaria subbifurcata*	S—GOM	Sp	N	D				
Pholidae	*Pholis gunnellus*	S—GOM	W	N	D				E
Anarhichadidae	*Anarhichas* spp.	GB—GOM	W	M	D				E
Ammodytidae	*Ammodytes* spp.	S—GOM	W	N	D	North		Deep	L
Scombridae	*Auxis* spp.	M—GB	Su	N	P		In		
	*Scomber scombrus*	S—GOM	Su	M	P		In		L
Stromateidae	*Peprilus* spp.	M—GB	Su	M	P				
Scophthalmidae	*Scophthalmus aquosus*	M—GB	Su	M	D	North			E
Paralichthyidae	*Citharichthys arctifrons*	M—GB	Su	N	D	North	In		L
	*Etropus* spp.	M—GB	Su	N	D	North			
	*Paralichthys dentatus*	M—GB	F	M	D				
	*Hippoglossina oblonga*	M—GB	Su	N	D	North			L
Paralichthyidae	*Syacium* spp.	M—GB	Su	N	D				
Bothidae	*Bothus* spp.	M—GB	Su	N	D				
Pleuronectidae	*Glyptocephalus cynoglossus*	S—GOM	Sp	M	D				
	*Hippoglossoides platessoides*	S—GOM	Sp	M	D				
	*Limanda ferruginea*	M—GB	Sp	M	D	North			E
	*Pseudopleuronectes americanus*	S—GOM	W	M	D				E
Cynoglossidae	*Symphurus* spp.	M—GB	Su	N	D				L

**Table 2:** Change in spatial distribution of adult fish in the Northeast U.S. Shelf Ecosystem. Distribution in the annual relative proportions among strata between 1977–1987 and 1999–2008 of spring- and fall-collected adult fish were tested separately using Kruskal-Wallis chi-square. By examining changes in slopes using linear analyses, changes were analyzed in: the along-shelf, the cross-shelf, and the depth directions. Results were classified into categories for each distributional shift: no significant change (blank), southward shift (South), northward shift (North), inshore shift (In), offshore shift (Off), shallower (Shallow), and deeper (Deep). Spatial shifts were examined relative to regional occurrence (M—GB = Mid-Atlantic Bight to Georges Bank, S—GOM = Southern New England to Gulf of Maine, and GB—GOM = Georges Bank to Gulf of Maine), management status (M = managed, N = not managed), and habitat type (D = demersal, P = pelagic).

					Spring Survey			Fall Survey		
Family	Taxon	Region	Managed	Habitat	Along-Shelf	Cross-Shelf	Depth	Along-Shelf	Cross-Shelf	Depth
Squalidae	*Squalus acanthias*	S—GOM	M	D				South		
Rajidae	*Amblyraja radiate*	GB—GOM	M	D						
	*Leucoraja erinacea*	M—GB	M	D	South			South		
	*Leucoraja garmani*	M—GB	M	D						
	*Leucoraja ocellata*	S—GOM	M	D						
	*Malacoraja senta*	GB—GOM	M	D				North		
	*Raja eglanteria*	M—GB	M	D						Deep
Clupeidae	*Clupea harengus*	S—GOM	M	P		Off				Deep
Gadidae	*Gadus morhua*	S—GOM	M	D			Deep			
	*Melanogrammus aeglefinus*	GB—GOM	M	D						
	*Pollachius virens*	GB—GOM	M	P			Deep			Deep
Lotidae	*Brosme brosme*	GB—GOM	M	D						
	*Enchelyopus cimbrius*	S—GOM	N	D						
Phycidae	*Urophycis chuss*	S—GOM	M	D	North	In	Deep	North	In	Deep
	*Urophycis tenuis*	S—GOM	M	D	North		Deep			Deep
Merlucciidae	*Merluccius albidus*	S—GOM	M	P	South					Shallow
	*Merluccius bilinearis*	S—GOM	M	P	North	In	Deep	North	In	
Lophiidae	*Lophius americanus*	S—GOM	M	D	North		Deep			
Sebastidae	*Sebastes* spp.	GB—GOM	M	D		Off				
Cottidae	*Myoxocephalus octodecemspinosus*	S—GOM	N	D	North					
Serranidae	*Centropristis striata*	M—GB	M	D						
Pomatomidae	*Pomatomus saltatrix*	M—GB	M	P						
Sparidae	*Stenotomus chrysops*	M—GB	M	P						
Sciaenidae	*Cynoscion regalis*	M—GB	M	D				North		
	*Leiostomus xanthurus*	M—GB	M	D						
	*Micropogonias undulatus*	M—GB	M	D					In	
Zoarcidae	*Zoarces americanus*	S—GOM	M	D				North		
Anarhichadidae	*Anarhichas* spp.	GB—GOM	M	D					In	
Ammodytidae	*Ammodytes* spp.	S—GOM	N	D						
Scombridae	*Scomber scombrus*	S—GOM	M	P	North	In		North		
Stromateidae	*Peprilus* spp.	M—GB	M	P		In			In	Shallow
Scophthalmidae	*Scophthalmus aquosus*	M—GB	M	D	North					
Paralichthyidae	*Citharichthys arctifrons*	M—GB	N	D	North				In	
	*Paralichthys dentatus*	M—GB	M	D	North			North		
Paralichthyidae	*Hippoglossina oblonga*	M—GB	N	D			Deep	North		
Pleuronectidae	*Glyptocephalus cynoglossus*	M—GB	M	D			Deep			
	*Hippoglossoides platessoides*	GB—GOM	M	D		Off			Off	
	*Hippoglossus hippoglossus*	GB—GOM	M	D						
	*Limanda ferruginea*	S—GOM	M	D	North		Deep			
	*Pseudopleuronectes americanus*	S—GOM	M	D						

We then examined spatial patterns using the Kruskal-Wallis Chi-square test statistics to infer larger-scale changes in the distribution. The stratum-specific Kruskal-Wallis H [49] values were considered a measure of the magnitude of change. Three linear regressions were calculated using the strata Kruskal-Wallis H as the independent variable and along-shelf distance (km), cross-shelf distance (km), and depth (m) values as the dependent variables to test whether distribution changes were coherent in the along-shelf, cross-shelf, and depth dimensions. The center points of each of the 47 strata (Fig 1) were estimated using MATLAB (The Mathworks 2008). The distance (km) north of Cape Hatteras, North Carolina and the distance (km) from 200-m isobath were calculated for each center point following Nye et al. [4]. Depth (m) of each stratum was calculated as the average depth of all stations sampled in the stratum. The cross-shelf distance and depth of strata of the NEUS shelf are not correlated due to the complex bathymetry of Georges Bank and Gulf of Maine (Pearson's correlation coefficient = -0.21, p-value = 0.16). Kruskal-Wallis H values for each stratum were set to negative if the relative proportion for a stratum was greater during 1977–1987, and positive if the proportion was greater during 1999–2008. For larvae, each stratum had three or four values depending on the number of bi-monthly seasons, and regressions were calculated for the combined bi-monthly seasons. For adults, each stratum had two values (spring and fall), and regressions were calculated for the spring and fall trawl surveys separately. The slope of each line was tested using a linear regression model [49] to determine whether it was significantly different than zero. No change in distribution was indicated with a slope that was not significantly different than zero. A significant change in slope was used to determine the change in distribution for each of the three linear analyses. A negative along-shelf slope was indicative of a southward shift, and positive along-shelf slope was indicative of a northward shift in distribution. A negative cross-shelf slope was indicative of an inshore shift, and positive cross-shelf slope was indicative of an offshore shift in distribution. A negative depth slope was indicative of a shift deeper, and a positive slope was indicative of a shift shallower in distribution.

### Analysis of Timing of Larval Occurrence

We compared timing of larval occurrence between two time periods: 1977–1987 (MARMAP, n = 11 years) and 1999–2008 (EcoMon, n = 10 years), using similar methods used to examine changes in distribution. Larvae were examined for the three highest ranked bi-monthly seasons based on annual relative proportion using the survey-design strata and non-parametric statistics. We again limited to only strata that made up at least 99% of the empirical cumulative distribution. We used a Kruskal-Wallis test to evaluate whether the relative proportion in a bi-monthly season was different between the two time periods. We then examined changes in mean relative proportion between the two time periods across all six seasons for taxa with a significant change among the time periods. If the relative annual proportion between the two time periods was not significantly different for any of the three highest ranked bi-monthly seasons, the taxon was classified as exhibiting no change. If there was a significant increase in annual proportion earlier in the season, the resulting change was classified as earlier. If there was a significant increase in annual proportion later in the season, the resulting change was classified as later. Changes in timing of larval occurrence were examined for 45 taxa (Table 1).

### Analysis of Synchrony in Larval and Adult Distributions

Synchrony of changes in distribution patterns across life-stage was explored for the 27 taxa where both larval and adult changes in distribution were examined. If neither life stage had a significant shift in distribution the taxon was classified as exhibiting no change. If both life stages had a significant shift in distribution and were shifting similarly in at least one direction (along-shelf, cross-shelf, or depth; e.g., larvae shifting northward and spring-collected adults shifting northward and deeper), the taxon was classified as in sync. If one life stage exhibited a significant shift and the other life stage exhibited a significant shift in another direction (e.g., one stage shifting northward and the other shifting inshore) or exhibited no significant shift (e.g., one stage shifting northward and the other not shifting), the taxon was classified as out of sync. In addition, a linear regression was calculated between the change in larval distributions and the change in adult distributions. This analysis used the slope of the linear regressions of the Kruskal-Wallis H values and the three distribution dimensions: along-shelf (km), cross-shelf (km), and depth (m). Adults from the spring and fall trawl surveys were compared separately to larvae combined across the three or four larval bi-monthly seasons. The slope of each regression was tested to determine whether the metrics of larval change and adult change were significantly related. A non-significant slope would indicate changes in distribution among life stages were not related for most taxa. A significant positive slope would indicate both life stages of most taxa were changing similarly. A significant negative slope would indicate both life stages of most taxa were changing dissimilarly.

### Relationship of Spatial and Seasonal Distribution Changes to Region, Season, Management Status and Habitat

Changes in distribution and timing of larval occurrence were also examined relative to regional occurrence, timing of larval occurrence in the NEUS Shelf, management status, and habitat. Regional occurrence was designated based on which strata were occupied by each taxon and life-stage (Table A in S1 File) and classified as: Mid-Atlantic Bight to Georges Bank, Southern New England to Gulf of Maine, and Georges Bank to Gulf of Maine (Fig 1). Timing of larval occurrence in the NEUS Shelf was based on the three highest ranked bi-monthly seasons for each taxon and classified as: winter, spring, summer, and fall. Management status and habitat use for all taxa and life stages were determined based on habitat use by the adult stage and whether or not the adults are part of a managed fishery. Management status categories were managed or not managed. Habitat categories were demersal or pelagic. Contingency tables were used to test three null hypotheses for the larval life stage: 1) no relationships among spatial changes in distribution across regions of occurrence, timing of larval occurrence, management status, or habitat type, 2) no relationships among the synchrony of spatial changes in distribution (in or out of sync) across regions of occurrence, timing of larval occurrence, management status, or habitat type and 3) no relationships among seasonal changes in occurrence across regions of occurrence, timing of larval occurrence, management status, or habitat type. The same null hypotheses were examined for the adult life stage, except for the timing of larval occurrence.

## Results

### Analysis of Spatial Distributions

**Fig 2:**
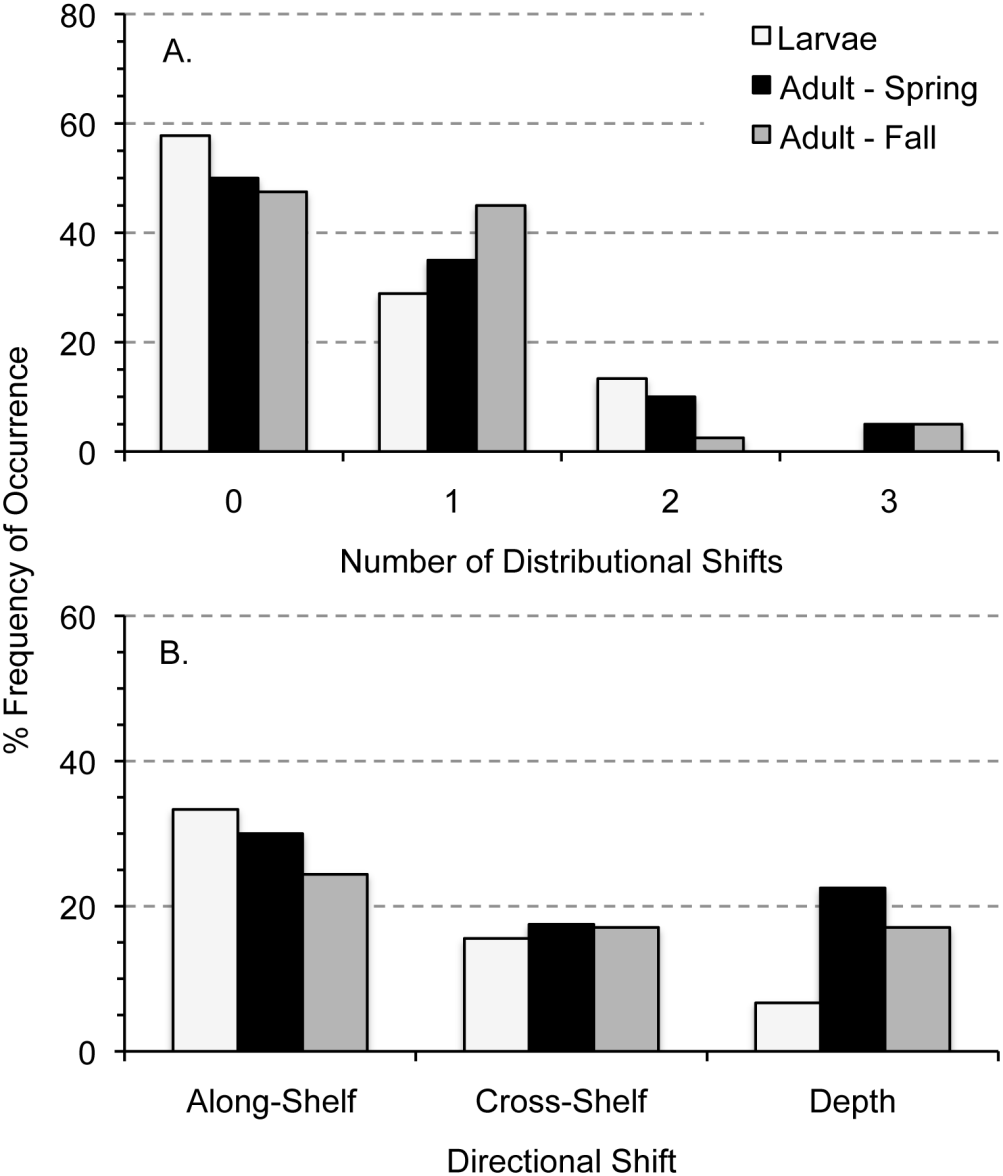
Percent frequency of occurrence in number of significant distributional shifts and significant directional shifts for taxon and life stages in the Northeast U.S. Shelf Ecosystem. Significant differences in annual relative proportions were used to examine three directional shifts of larvae and spring- and fall-collected adults separately; therefore, individual taxon could have shifted in as many as three directions (A). Slopes that were significantly different than zero indicated a significant change in distribution in one of the directions: along-shelf, cross-shelf, or depth (B).

Approximately half the taxa in this study had at least one directional change in distribution comparing 1977–1987 to 1999–2008 (Fig 2A). Forty-three percent of larval taxa (n = 19) exhibited change compared to 50% of adult taxa (n = 28). About 36% of taxa, of both life stages, showed evidence of a single change of direction: along-shelf, cross-shelf, and depth. Twelve percent of taxa exhibited a change in more than one direction, with adults the only life stage changing in all three directions (Fig 2A). Larvae and adults had distribution changes more frequently in the along-shelf direction than the other two directions (Fig 2B). Both life stages shifted in similar frequency in the cross-shelf, while larvae shifted less frequently in depth than adults (Fig 2B).

**Fig 3:**
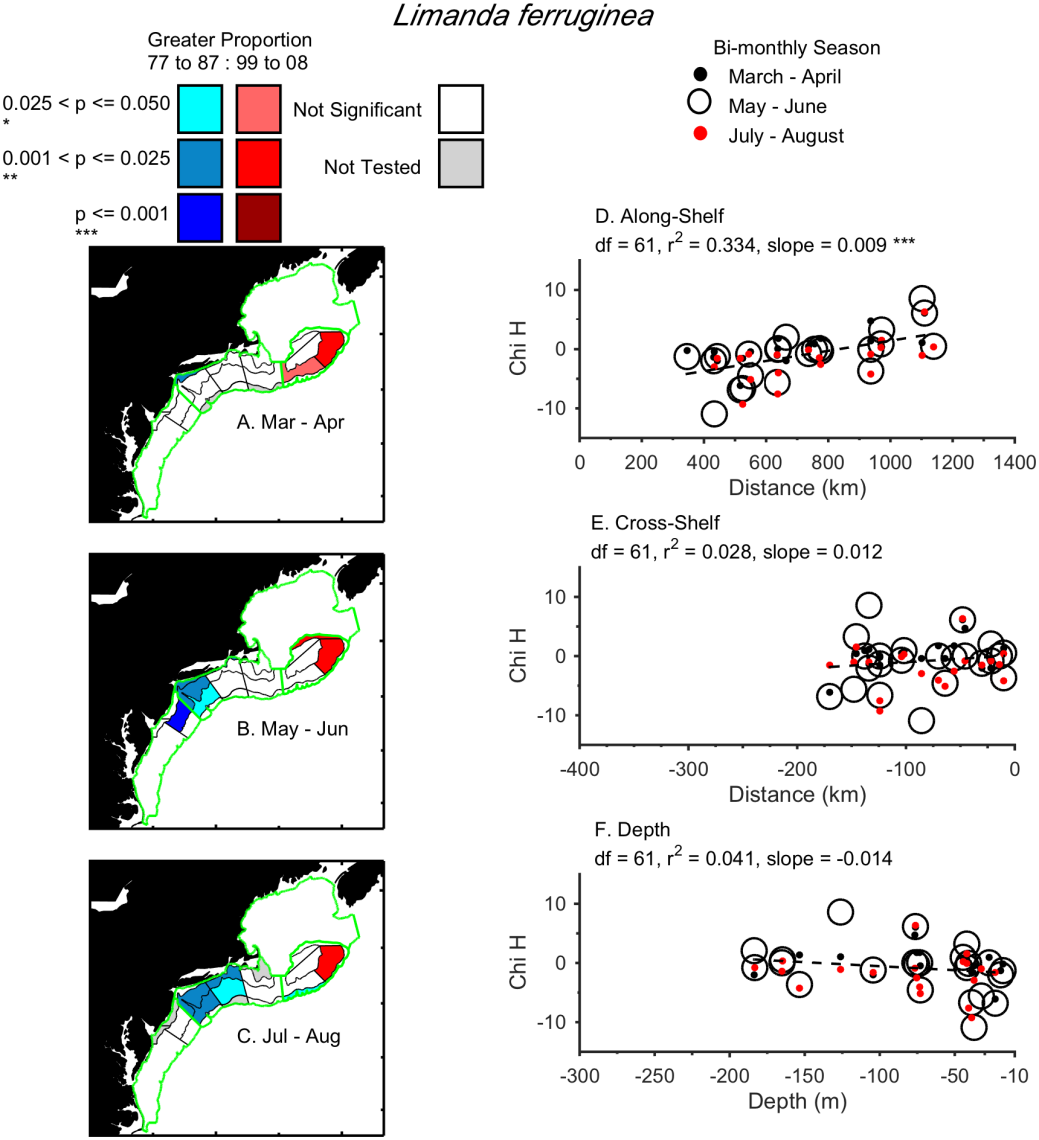
Change in distribution of larval *Limanda ferruginea* (Yellowtail Flounder) in the Northeast U.S. Shelf Ecosystem. Locations of strata with significant differences in annual relative proportions of larval *Limanda ferruginea* between 1977–1987 and 1999–2008 in March—April (A), and May—June (B), and July—August (C), were used to examine directional shifts. Linear regressions of the Kruskal-Wallis chi-square (Chi H) for each stratum were tested for each direction: along-shelf (C), cross-shelf (D), and depth (E) to determine if slopes were significantly different than zero, and classify the significant directional shifts. Larval *Limanda ferruginea* shifted significantly northward.

**Fig 4:**
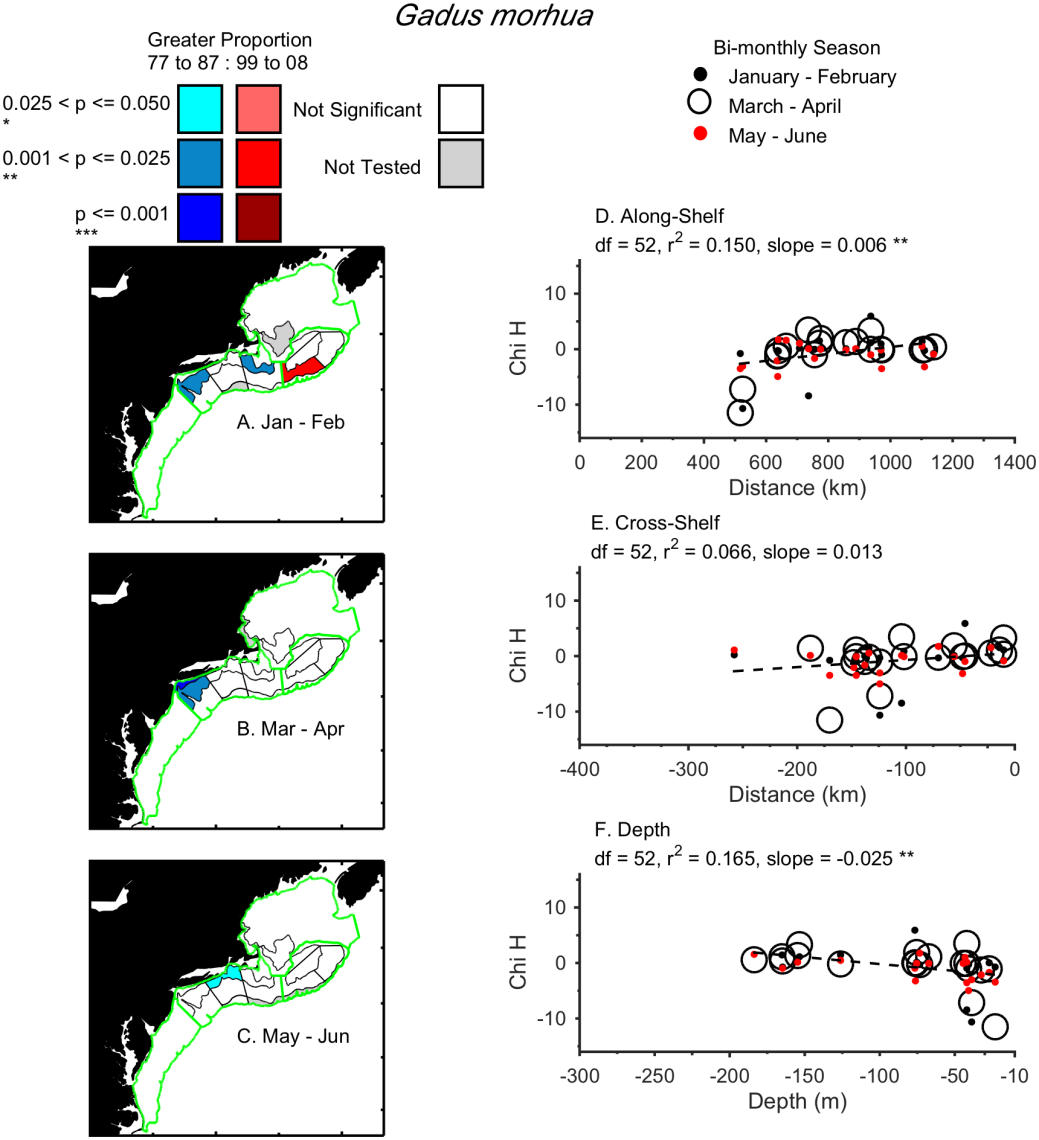
Change in distribution of larval *Gadus morhua* (Atlantic Cod) in the Northeast U.S. Shelf Ecosystem. Locations of strata with significant differences in annual relative proportions of larval *Gadus morhua* between 1977–1987 and 1999–2008 in January—February (A), March—April (B), and May—June (C), were used to examine directional shifts. Linear regressions of the Kruskal-Wallis chi-square (Chi H) for each stratum were tested for each direction: along-shelf (C), cross-shelf (D), and depth (E) to determine if slopes were significantly different than zero, and classify the significant directional shifts. Larval *Gadus morhua* shifted significantly northward and deeper.

**Fig 5:**
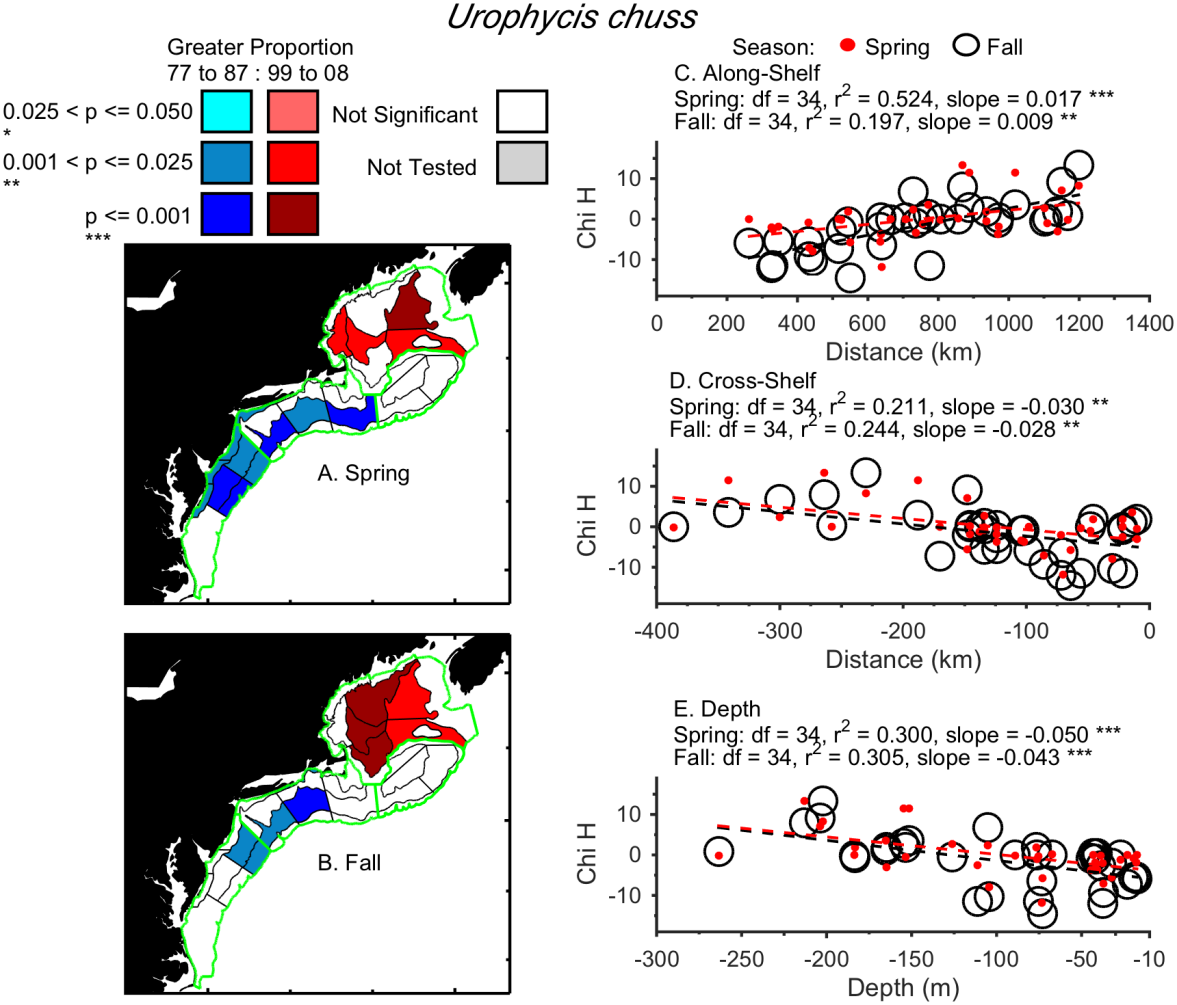
Change in distribution of adult *Urophycis chuss* (Red Hake) in the Northeast U.S. Shelf Ecosystem. Locations of strata with significant differences in annual relative proportions of adult *Urophycis chuss* between 1977–1987 and 1999–2008 in the spring (A) and fall (B) were used to examine directional shifts. Linear regressions of the Kruskal-Wallis chi-square (Chi H) for each stratum were tested for each direction: along-shelf (C), cross-shelf (D), and depth (E) to determine if slopes were significantly different than zero for each season, and classify the significant directional shifts. Adult *Urophycis chuss* shifted significantly northward, inshore, and deeper in the spring and fall.

Three examples of different distribution shifts are provided. Larval *Limanda ferruginea* shifted in a single direction, northward, during March to August from 1977–1987 to 1999–2008 (Fig 3). There were proportionally fewer larvae in the northern Mid-Atlantic Bight and Southern New England and more on northeast Georges Bank in recent years compared to the 1970’s and 1980’s. Larval *Gadus morhua* shifted in two directions, both northward and deeper, during January to June (Fig 4). From 1999–2008, larvae were proportionally less abundant in Southern New England and more abundant on the southern flank of Georges Bank, which is deeper and further north. Finally, adult *Urophycis chuss* shifted significantly northwards, inshore, and deeper, during both the spring and fall (Fig 5). By shifting into the Gulf of Maine in recent years, *Urophycis chuss* moved northward, and both inshore (away from the shelf-break) and into deeper water (Fig 5). Results for each taxon by life-stage are summarized in Table 1 (larvae) and Table 2 (adults) and (Figs A—CG in S1 File).

**Fig 6:**
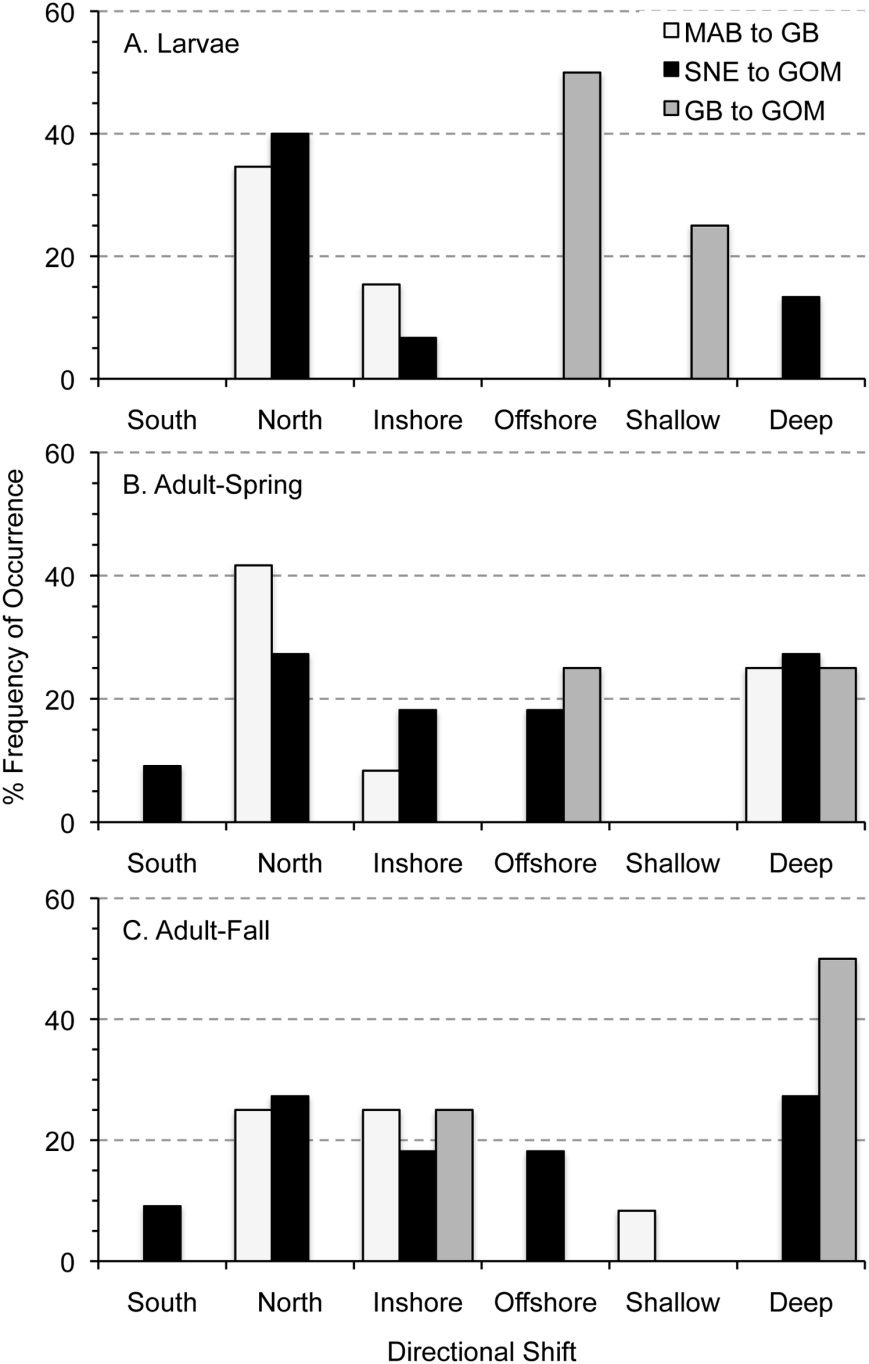
Percent frequency of occurrence of directional shifts in distribution among taxa by life stage and regions of the Northeast U.S. Shelf Ecosystem. Occurrence of each type of distributional shift among larvae (A) and spring- (B) and fall-collected adults (C) were grouped by taxa that occurred primarily in the Mid-Atlantic Bight to Georges Bank (MAB to GB), Southern New England to Gulf of Maine (SNE to GOM), and Georges Bank to Gulf of Maine (GB to GOM) to examine patterns in relation to regional occurrence.

Changes in spatial distributions were aggregated by regional occurrence, timing of larval occurrence, management status, and habitat type for larvae and adults to examine broad taxa patterns. Relationships among directional shifts in distribution were significantly related to regional occurrence for larvae, but not adults (Table 3). Larvae that occurred primarily in the Mid-Atlantic to Georges Bank shifted mainly northward, and more in the cross-shelf and less in depth than expected from random (Fig 6A). Adults of the same region shifted mainly northward and deeper in the spring and northward (Fig 6B) and inshore in the fall (Fig 6C). Southern New England to Gulf of Maine larval taxa shifted northward (Fig 6A), but shifted less than expected in the cross-shelf direction (Table 3). Adult changes in distribution for Southern New England to Gulf of Maine taxa were more variable in both spring and fall (Fig 6B and 6C). Georges Bank to Gulf of Maine larvae shifted more than expected in both the cross-shelf and depth directions (Table 3), and moved offshore and shallower (Fig 6A). Adults of Georges Bank to Gulf of Maine shifted offshore and deeper in the spring (Fig 6B), and deeper and inshore in the fall (Fig 6C). Widening of the shelf to the north and the movement of some taxa into the Gulf of Maine, which is both inshore (in the reference system used here) and deeper, explain these seemingly contradictory results (inshore and deeper).

**Table 3:** Relationships among regional occurrence, timing of larval occurrence, management status, and habitat type to directional shifts and synchrony for taxa in the Northeast U.S. Shelf Ecosystem. Contingency tables were used to test the null hypotheses: that there were no relationships among spatial changes in distribution across region, larval season, management status, or habitat type. Relationships were examined relative to regional occurrence (M—GB = Mid-Atlantic Bight to Georges Bank, S—GOM = Southern New England to Gulf of Maine, and GB—GOM = Georges Bank to Gulf of Maine), larval season (W = winter, Sp = spring, Su = summer, and F = fall), management status (M = managed, N = not managed), and habitat type (D = demersal, P = pelagic). Significant relationships (p ≤ 0.05) are shown and were classified into categories for directional shift and synchrony: no significant relationship (NS), no data (ND), more shifts than expected (More), fewer shifts than expected (Less), more taxa out of sync than expected (Out).

		Directional Shift				
			Adult		Synchrony	
	Direction	Larvae	Spring	Fall	Spring	Fall
Regional Occurrence	Along-shelf					
	Cross-shelf	M-GB = More, S-GOM = Less, GB-GOM = More	NS	NS	NS	NS
	Depth	M-GB = Less, S-GOM = Less, GB-GOM = More	NS	NS	NS	NS
Larval Season	Along-shelf	NS	ND	ND	NS	NS
	Cross-shelf	NS	ND	ND	NS	NS
	Depth	NS	ND	ND	NS	NS
Management Status	Along-shelf	NS	NS	NS	NS	NS
	Cross-shelf	NS	NS	NS	NS	NS
	Depth	NS	NS	NS	NS	NS
Habitat Type	Along-shelf	NS	NS	NS	NS	NS
	Cross-shelf	P = More	P = More	NS	P = Out	P = Out
	Depth	NS	NS	P = More	NS	P = Out

**Fig 7:**
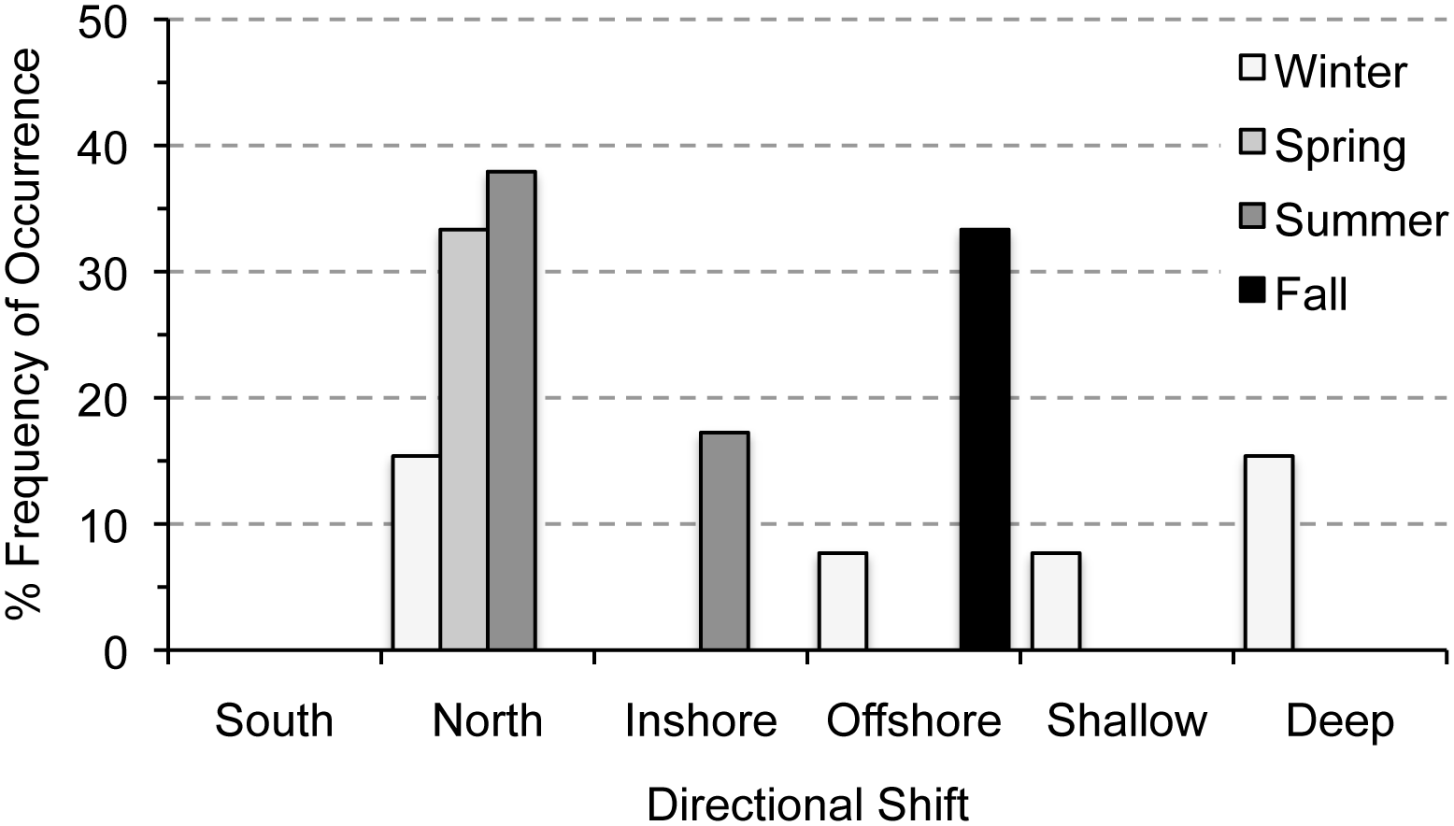
Percent frequency of occurrence of directional shifts in distribution among larval taxa by seasonal occurrence in the Northeast U.S. Shelf Ecosystem. Occurrence of each type of distributional shift for larvae were grouped by season to examine patterns in relation to seasonal occurrence.

Relationships among directional shifts in distribution and seasonal timing of larval occurrence were examined for the larval stage only, and not found to be significant (Table 3). About 55% of summer taxa had a significant change in distribution, and shifted northward and inshore (Fig 7). Slightly fewer winter taxa shifted distributions (46%), and exhibited the most variability (Fig 7). Spring and fall taxa shifted the least (33%). Spring taxa shifted only northward, and fall taxa only shifted offshore (Fig 7).

**Fig 8:**
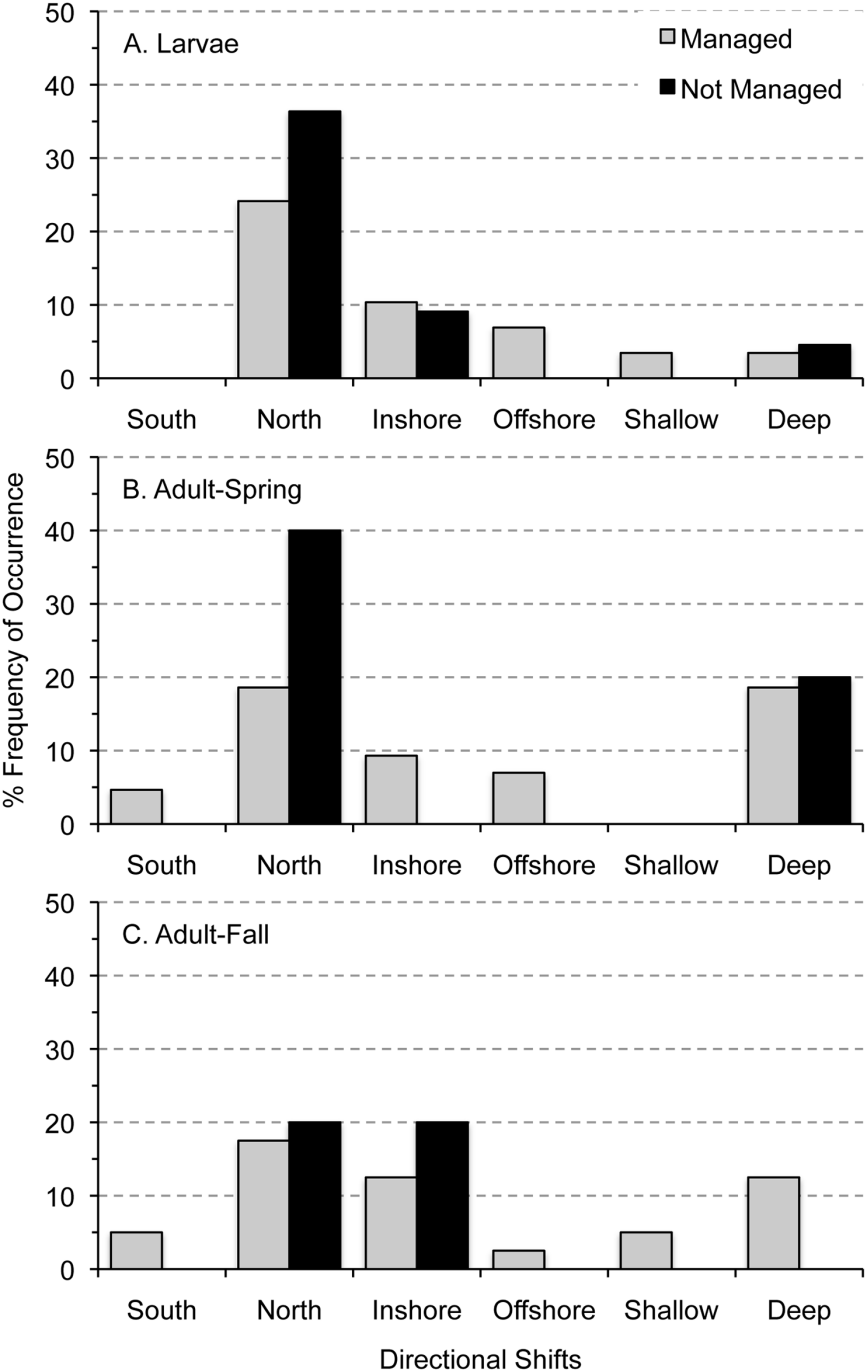
Percent frequency of occurrence of directional shifts in distribution among taxa by life-stage and management status in the Northeast U.S. Shelf Ecosystem. Occurrence of each type of distributional shift among taxa of larvae (A) and spring- (B) and fall-collected adults (C) were grouped as managed and not managed to examine patterns in relation to management status.

Relationships among directional shifts in distribution and management status were not significant for larvae or adults (Table 3). Larvae of managed taxa shifted less (55%) than taxa that are not managed (60%), and managed taxa were more variable in directional shifts when they did shift distributions (Fig 8A). Adults of the two categories shifted distribution for about 50% of the taxa (Table 2), and managed taxa were again more variable in directional shifts when they did shift distributions (Fig 8B and 8C). Taxa that are not managed shifted mainly northward for spring-collected adults (Fig 8B), and adults in the fall shifted northward and inshore (Fig 8C).

**Fig 9:**
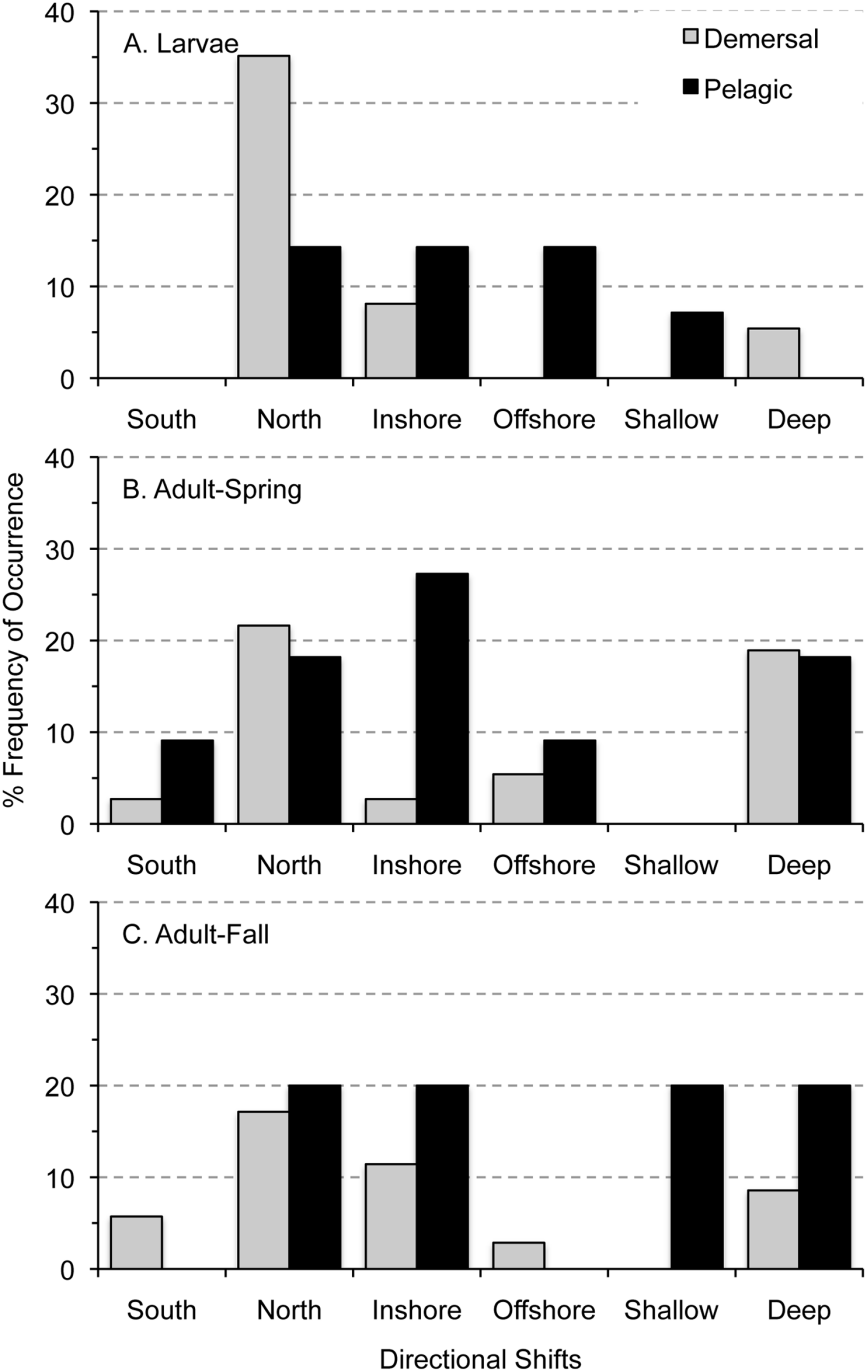
Percent frequency of occurrence of directional shifts in distribution among taxa by life-stage and habitat type in the Northeast U.S. Shelf Ecosystem. Occurrence of each type of distributional shift among taxa of larvae (A) and spring- (B) and fall-collected adults (C) were grouped based on adult use of the demersal and pelagic habitat to examine patterns in relation to habitat type.

Relationships among directional shifts in distribution and habitat type were significant for larvae and adults (Table 3). Pelagic taxa of both larvae (57%) and adults (75%) shifted distributions (Tables 1 and 2). As larvae, demersal taxa shifted northward, and pelagic taxa were more variable (Fig 9A) and shifted more in the cross-shelf direction than expected (Table 3). Demersal adult taxa shifted mostly northward and deeper in the spring (Fig 9B) and northward, inshore, and deeper in the fall (Fig 9C). Pelagic taxa were more variable than demersal taxa as adults (Fig 9B and 9C), and shifted more than expected in both the cross-shelf and depth directions (Table 3).

### Analysis of Timing of Larval Occurrence

**Fig 10:**
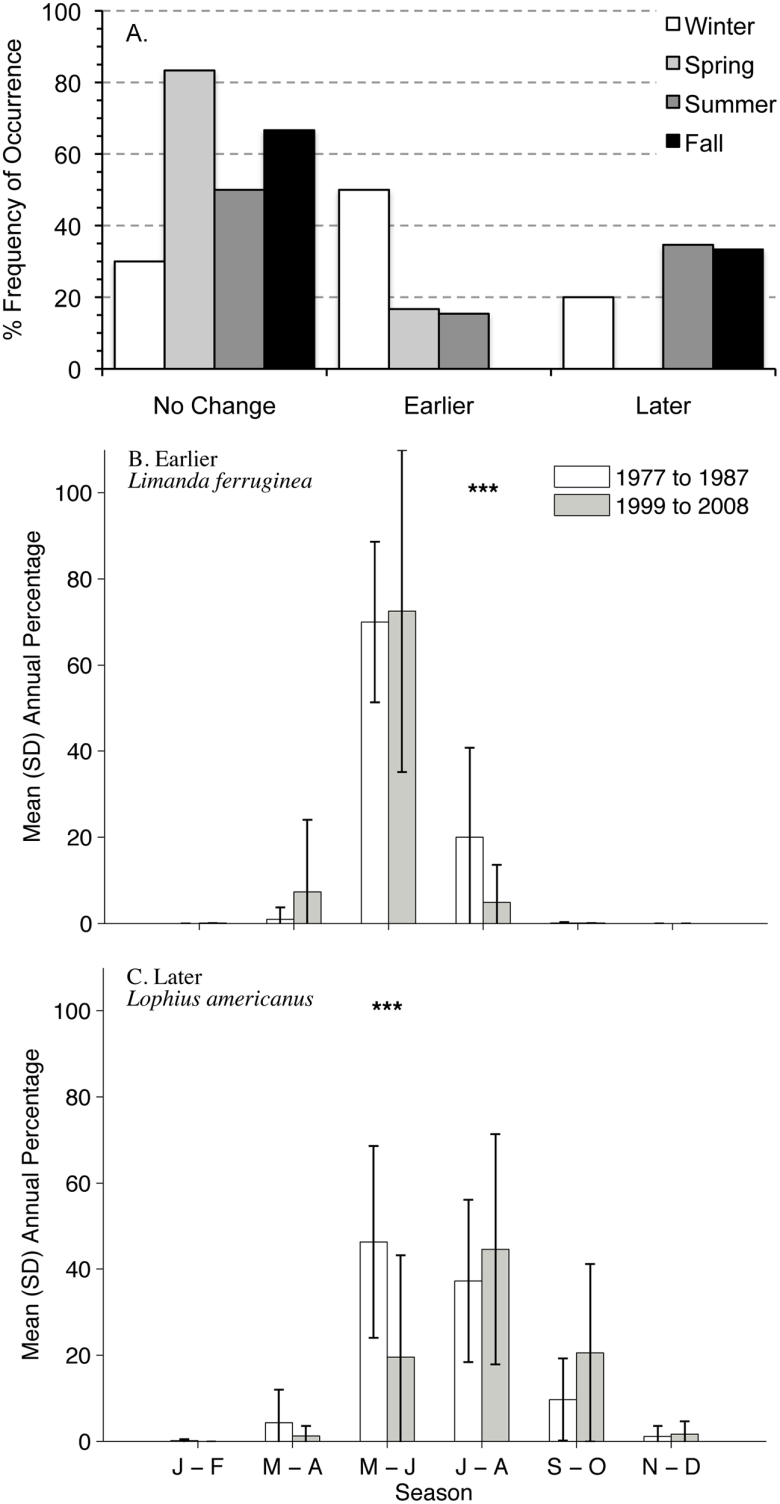
Percentage of taxa classified by change in timing of larval occurrence and examples of earlier and later shifts in the Northeast U.S. Shelf Ecosystem. Changes in relative proportion of larvae in bi-monthly seasonal larval collections between 1977–1987 and 1999–2008 were tested using Kruskal-Wallis chi-square. Seasonal changes were classified as no change, earlier, or later and grouped by the larval season of occurrence in the NEUS Shelf (A). Examples of temporal shifts include: *Limanda ferruginea* (B), which occurred earlier in the season during the 1999–2008 period and *Lophius americanus* (C), which occurred later in the season. * 0.025 < p ≤ 0.05, ** 0.001 < p ≤ 0.025, *** p ≤ 0.001.

Changes in timing of larval occurrence were detected for 49% of the 45 taxa examined (Table 1) with the highest frequency of change for winter taxa and lowest for spring taxa (Fig 10A). When a shift in timing of larval occurrence was detected, winter and spring taxa shifted most frequently earlier in the season and summer and fall taxa shifted later (Fig 10A). A majority of detected shifts in seasonality were a change in relative abundance earlier or later in the season of larval occurrence rather than a change in the timing of peak larval occurrence. For example, the peak in abundance of *Limanda ferruginea* remained in May—June, but abundance was higher in 1999–2008 during March—April and significantly lower in July—August than in 1977–1987 (Fig 10B). About 35% of the taxa (8 taxa) experienced shifts in the timing of peak annual abundance. For example, the peak abundance of *Lophius americanus*, shifted from May—June in 1977–1987 to July—August in 1999–2008 (Fig 10C). Results for each taxon are summarized in Table 1 and the S1 File. Seasonal shifts in larval occurrence were not related to regional occurrence (p = 0.61), timing of larval occurrence (p = 0.15), management status (p = 0.49), or habitat type (p = 0.33).

### Analysis of Synchrony in Larval and Adult Distributions

**Fig 11:**
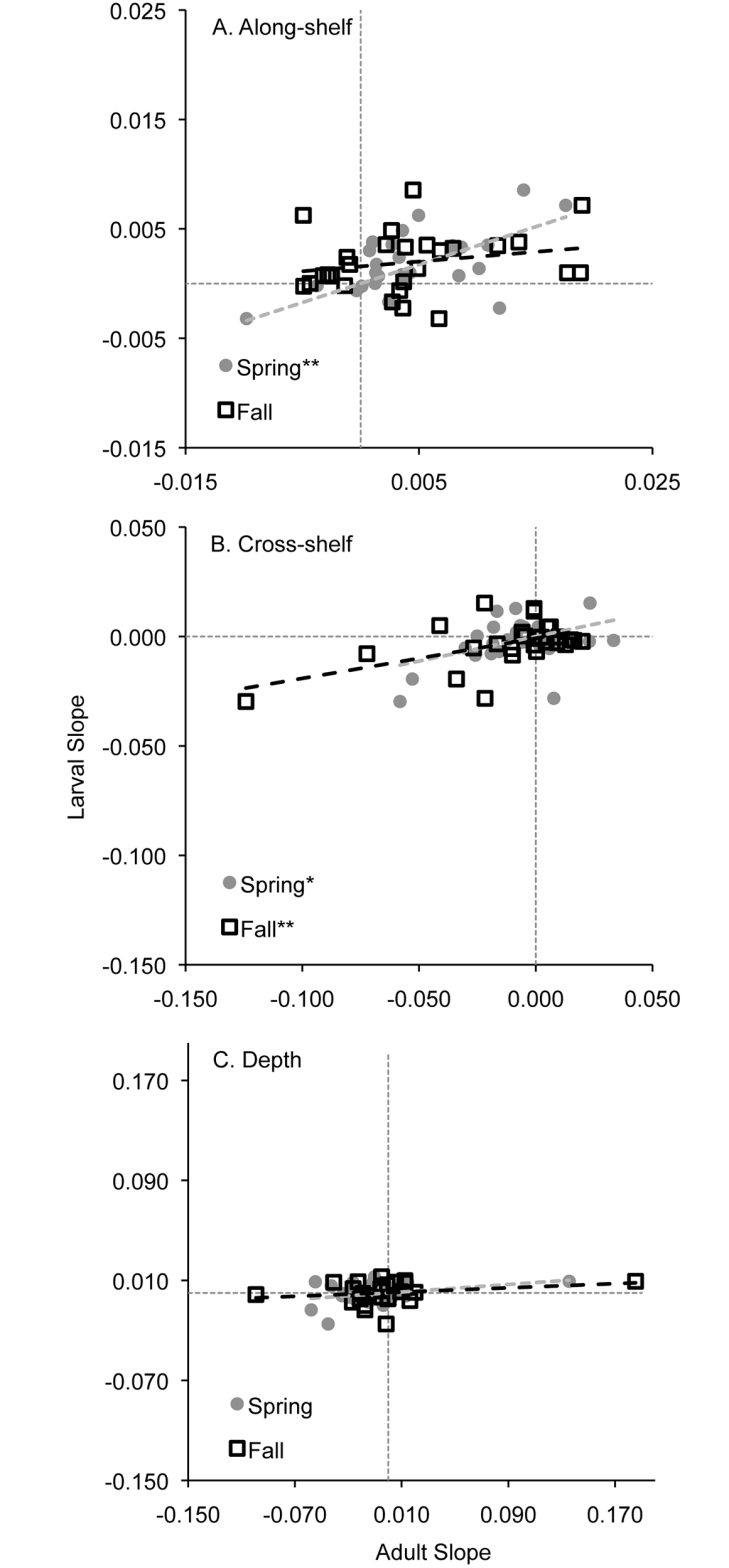
Relationship between directional shifts in distribution of larvae and adults in the Northeast U.S. Shelf Ecosystem. Slopes of the linear regressions of the Kruskal-Wallis H values were used for each the 27 taxa where both life stages were examined in the along-shelf (A), cross-shelf (B), and depth (C) directions. Adults from the spring and fall trawl surveys were compared separately to larvae, and the slope of each regression line was tested to determine whether it was significantly different from zero, indicating the metrics of larval change and adult change were significantly related. * 0.025 < p ≤ 0.05, ** 0.001 < p ≤ 0.025, *** p ≤ 0.001.

Distribution shifts were in sync for 10 of the 27 taxa where both larvae and adults were examined, and out of sync for 13 taxa (Table 4). The remaining four taxa exhibited no significant change in distribution for either the larvae or adults (Table 4). Combining all taxa, along-shelf and cross-shelf shifts in distribution were correlated when comparing slope values between larvae and adults (Fig 11). Larvae were also more frequently in sync with spring-collected than fall-collected adults (Table 4). Significant positive slopes occurred during the spring for along-shelf (Fig 11A) and cross-shelf distributions (Fig 11B), and synchrony was most frequent for northward shifts (Table 4). During the fall, significant positive slopes occurred for cross-shelf distributions (Fig 11B), and synchrony occurred for both inshore and northward shifts (Table 4). Depth was the least correlated between the two life stages, with slopes near zero (Fig 11C). However, *Gadus morhua* was in sync with deeper distributions during the spring in the most recent decade for both life stages (Table 4).

**Table 4:** Synchrony of change in spatial distribution of larvae and adults in the Northeast U.S. Shelf Ecosystem. For the 27 taxa where both larval and adult distribution patterns were examined, life-stage synchrony in change patterns was explored in the: along-shelf, cross-shelf, and depth directions for each season adults were collected (spring and fall). Individual taxa were classified as no change if no significant change in distribution was detected for either life stage. If both life stages had a significant shift in distribution and were shifting in the same direction, the taxon was classified as in sync. If one life-stage exhibited a significant shift and the other life-stage exhibited a significant shift in another direction or exhibited no significant shift, the taxon was classified as out of sync.

			Directional Change		
				Adult	
Taxon	Synchrony	Pattern	Larval	Spring	Fall
*Merluccius bilinearis*	In	Both Seasons	North	North, Inshore	North, Inshore
*Citharichthys arctifrons*	In	Both Seasons	North, Inshore	North	Inshore
*Gadus morhua*	In	Spring	Deep, North	Deep	
*Scomber scombrus*	In	Spring	Inshore	Inshore	
*Lophius americanus*	In	Spring	North	North	
*Scophthalmus aquosus*	In	Spring	North	North	
*Limanda ferruginea*	In	Spring	North	North, Deep	
*Clupea harengus*	In	Spring	Offshore	Offshore	
*Micropogonias undulatus*	In	Fall	Inshore, North		Inshore
*Hippoglossina oblonga*	In	Fall	North		North
*Ammodytes* spp.	Out	Larval shift only	North, Deep		
*Enchelyopus cimbrius*	Out	Larval shift only	North		
*Centropristis striata*	Out	Larval shift only	North, Inshore		
*Peprilus* spp.	Out	Adult shift only-Both		Inshore	Inshore, Shallow
*Paralichthys dentatus*	Out	Adult shift only-Both		North	North
*Hippoglossoides platessoides*	Out	Adult shift only-Both		Offshore	Offshore
*Merluccius albidus*	Out	Adult shift only-Both		South	Shallow
*Glyptocephalus cynoglossus*	Out	Adult shift only-Spring		Deep	
*Myoxocephalus octodecemspinosus*	Out	Adult shift only-Spring		North	
*Sebastes* spp.	Out	Adult shift only-Spring		Offshore	
*Anarhichas* spp.	Out	Adult shift only-Fall			Inshore
*Cynoscion regalis*	Out	Adult shift only-Fall			North
*Pollachius virens*	Out	Both-Out of Sync	Offshore, Shallow	Deep	Deep
*Melanogrammus aeglefinus*	No Change	No Change			
*Pomatomus saltatrix*	No Change	No Change			
*Leiostomus xanthurus*	No Change	No Change			
*Pseudopleuronectes americanus*	No Change	No Change			

Synchrony in distribution changes among life stages was related to habitat type but not regional occurrence, larval season, or management status (Table 3). Pelagic taxa were more likely to be out of sync among life stages than demersal taxa. Life stages were out of sync in the cross-shelf direction during the spring, and in the cross-shelf and depth directions in the fall (Table 3).

## Discussion

The spatial distribution of approximately half the taxa examined in this study changed in one or more directions from 1977–1987 to 1999–2008. Most of the shifts were northward (23 taxa), potentially in response to warming water temperatures of the ecosystem [36–38]. Sea surface temperatures of the Northeast Large Marine Ecosystem have warmed 0.23°C over a similar time period (1982–2006) [37], with spatial and seasonal variation across the ecosystem [36]. Depth averaged water temperature on a cross-shelf transect of the northern Mid-Atlantic Bight increased an average of 0.026°C per year from 1977–2013, with an increased rate (0.11°C) since 2002 [38]. Both cold-temperate (e.g., *Gadus morhua*, *Urophycis chuss*, and *Limanda ferruginea*) and warm-temperate (e.g., *Citharichthys arctifrons* and *Cynoscion regalis*) taxa shifted northwards. Cold-temperate taxa may be shifting to remain in preferred water temperatures and warm-temperate may be expanding into newly available habitats [11, 50]. Adults of three taxa exhibited southward shifts: *Squalus acanthias*, *Leucoraja erinacea*, and *Merluccius albidus*. The specific reasons for the shifts of these three species is unknown, but these southward shifts demonstrate that species are responding differently under the same ecosystem scale forcing (e.g., warming). These species-specific differences could in part be explained by differences in a realized thermal niche [11], but could also be due to differences in response to fishing pressure (e.g., [51]) and potentially other stressors (e.g., trophic interactions, contaminants, habitat alteration) [52].

Distribution shifts identified in this study also occurred with water depth and in the cross-shelf direction. Approximately two-thirds of the cross-shelf shifts were inshore and three-quarters of the depth shifts were to deeper waters. Prior studies have identified shifts into deeper water (e.g., [2, 4]). Movement into deeper, but more inshore waters can be explained in part by movement of species into the Gulf of Maine (e.g., *Urophycis chuss*, *Merluccius bilinearis*), which is deeper and more inshore (in the reference system used here). The complexities of the bathymetry of the Gulf of Maine may provide refuge for species as climate continues to change (see [53]). In addition to movement into the Gulf of Maine, several species (e.g., *Scomber scomber*, *Peprilus triacanthus*) moved inshore in the Mid-Atlantic Bight suggesting a greater use of inshore areas in the recent decade (see [50]).

Regional occurrence affected distribution shifts of larvae. Part of our first expectation that larvae found in the Mid-Atlantic Bight and Southern New England would shift northwards was supported (Table 5). The second part, in which larvae in the Gulf of Maine region would shift into deeper parts of the shelf rather than northward, was partially supported (Table 5). Larvae in the Georges Bank to Gulf of Maine regions did not shift northward, but contrary to expectations, shifted offshore and shallower. The complex bathymetry of the Gulf of Maine and the increasing proportion of many taxa on Georges Bank in the recent decade can potentially explain this seemingly counterintuitive result. Overall, our results agree with other studies in that there are general patterns (e.g., northward shifts in distribution) [4, 54], but these patterns do not dictate the distribution of all species.

**Table 5:** *A priori* expectations and results for change in spatial distribution and seasonal occurrence of larval fish in the Northeast U.S. Shelf Ecosystem. Based on recent meta-analyses [34], studies from other regions [21, 22] and studies in the Northeast U.S. Shelf Ecosystem [4, 32], we developed several *a priori* expectations.

	Expectation		Analyses
Expectation	Result	Figs & Tables	Result
1a) Mid-Atlantic Bight, Southern New England, and Georges Bank larvae will shift either northwards or deeper	Supported	Fig 6A and 6B	Taxa from these regions shifted mainly northwards.
1b) Georges Bank and Gulf of Maine larvae will be deeper not northward	Partially Supported	Fig 6C	Larvae moved primarily to Georges Bank, which is offshore and shallower
2a) Northward shifts will be more frequent for larvae occurring in summer than winter	Supported	Fig 7	Summer taxa shifted the most, and mainly northwards.
2b) Northward shifts will be more frequent for larvae occurring in winter than summer	Not Supported	Fig 7	Winter taxa shifted less than summer taxa, but some taxa shifted northwards and deeper.
3) Timing of larval occurrence will shift later in the fall and winter and will shift earlier in the spring	Partially supported	Fig 10	Fall taxa shifted later and spring taxa shifted earlier, but more winter taxa shifted earlier.
4) Shifts in larval distribution will be comparable to shifts in adult distribution	Partially supported	Fig 11, Table 4	13 taxa out of sync and 10 in sync.
5) Shifts in larval distribution will be more frequent for pelagic than demersal taxa	Supported	Fig 9, Tables 1 & 3	Slightly more pelagic taxa (46%) shifted than demersal (41%). Pelagic taxa were also more variable and shifted in the cross-shelf more than expected than random.
6) Shifts in larval distribution will be more frequent for managed fish than unmanaged taxa	Not Supported	Fig 8, Table 1	Slightly fewer managed taxa (40%) shifted than unmanaged (45%).

Significant seasonal differences in distribution shifts were also identified. The second expectation, that northward shifts will be more frequent for larvae occurring in summer than winter, was supported (Table 5). The alternate expectation, northward shifts will be more frequent for larvae occurring in winter than summer, was not well supported (Table 5). Winter taxa shifted distributions less frequently than summer taxa, but many winter taxa shifted earlier in their timing of larval occurrence (Fig 10A). The migration timing and movement patterns of the adults may explain some of this complexity. Some adult taxa exhibited shifts in distribution in both spring and fall, but slightly more than half (52%) the taxa exhibited shifts in only one season (Table 2). Seasonal differences in adult distribution have been noted in another study that examined four species in detail [5]; distribution shifts were more evident in spring than in fall. In our study, adult distribution shifts were observed evenly in both spring and fall (Table 2). The NEUS Shelf Ecosystem has a very strong seasonal temperature cycle [36], varying as much as 18°C in sea surface temperature annually, and a number of species migrate seasonally [14]. Thermal habitats may be more limiting during certain seasons [53–55] and this will vary by species [11]. Understanding these seasonal dynamics could lead to insights into longer-term distribution responses to climate change.

A change in the timing of larval occurrence was observed in approximately 50% of taxa indicating potential changes in spawning time. Most of the changes were a shift in the shoulder season, but some changes were a shift in the peak season. All shifts in season for fall taxa were to later in the season, while spring taxa only shifted to earlier in their season, partially supporting expectation three (Table 5). Winter taxa shifted both earlier and later, partially supporting the expectation (Table 5). Genner et al. [23] found a correlation between changes in the timing of larval occurrence and temperature in the English Channel; spawning occurred earlier with increasing temperatures for spring spawners and later with increasing temperature for summer spawners. Our results are consistent with the hypothesis that warming in the NEUS Shelf [36] is causing changes in spawning times and locations for some species, implying a link between changes in distribution and changes in the environment.

The change in larval distributions implies a change in spawning, currents, survival [56], or some combination of the three. Long-term (30 year) changes in circulation over the NEUS Shelf have not been investigated, however, changes in both Gulf of Maine source waters [57] and variability in the along-shelf current in the Mid-Atlantic Bight over the past 15 years [58] have been documented. There have also been long-term changes in the position of the Gulf Stream, which may affect or may be linked to circulation in the NEUS Shelf [59]. It is unclear if these changes in advection are of the magnitude and direction necessary to explain the changes in larval distributions documented here. Changes in larval survival could explain some component of the distribution changes; recruitment per spawning stock biomass has changed for some stocks indicating a change in survival [60–62], but the magnitude of distribution changes was larger than would be expected by change in the distribution of larval survival. Changes in spawning distribution probably explain most of the changes in larval distribution. The number of taxa affected in the larval stage was comparable to the number of taxa affected in the adult stage, but the magnitude of distribution shift was less. Many species in the NEUS Shelf make seasonal migrations as adults, and the bottom trawl survey samples these dynamic distributions twice annually. The distributions of adults have changed more than the distributions of larvae implying that spawning locations are more conservative than overall adult distributions. However, changes in larval distributions do indicate that spawning distributions (or possibly advection) is also changing. These results also highlight the need to better understand life cycle connectivity in the NEUS Shelf [28].

Previous studies have identified changes in larval distributions of individual taxa over time (e.g., [63]), but here we show changes in larval distribution of approximately half the taxa examined. In approximately 40% of taxa examined, changes in larval and adult distributions were similar, partially supporting expectation four (Table 5). However, in approximately 60% of taxa, distribution changes differed between adult and larval life stages. This suggests that the spatial range a species uses throughout its life history (seasonal migrations, spawning locations, planktonic advection) is changing. These changes may have energetic consequences (greater migration distances, greater distances between nursery and feeding habitats) for specific species (e.g., *Enchelyopus cimbrius*, *Merluccius albidus*, *Centropristis striata*, and *Hippoglossoides platessoides*). Further, these results suggest that population connectivity may become more limiting for species whose distribution changes are out of sync between life stages.

The analyses of the effect of habitat on distribution shifts supported the expectation that pelagic species were more responsive than demersal species (Table 5). Shackell et al. [11] reported similar results for adult fish on the Scotian Shelf. Community variation of adult fish in the NEUS Shelf is correlated more with pelagic than demersal habitat variables [64]. These functional group generalities need to be explored in more detail, but mobile pelagic species may respond to changes in their environment with shifts in distribution, while less-mobile demersal species may respond with shifts in abundance and mortality [51, 65]. The results, relative to management status, do not support the expectation that managed species exhibit a greater degree of change than unmanaged species (Table 5). Management status may not be a good proxy for the stress of exploitation on a species since many of the fishery methods used in the ecosystem are not selective for specific species (i.e., trawling), and many managed species are experiencing low rates of exploitation.

**Fig 12:**
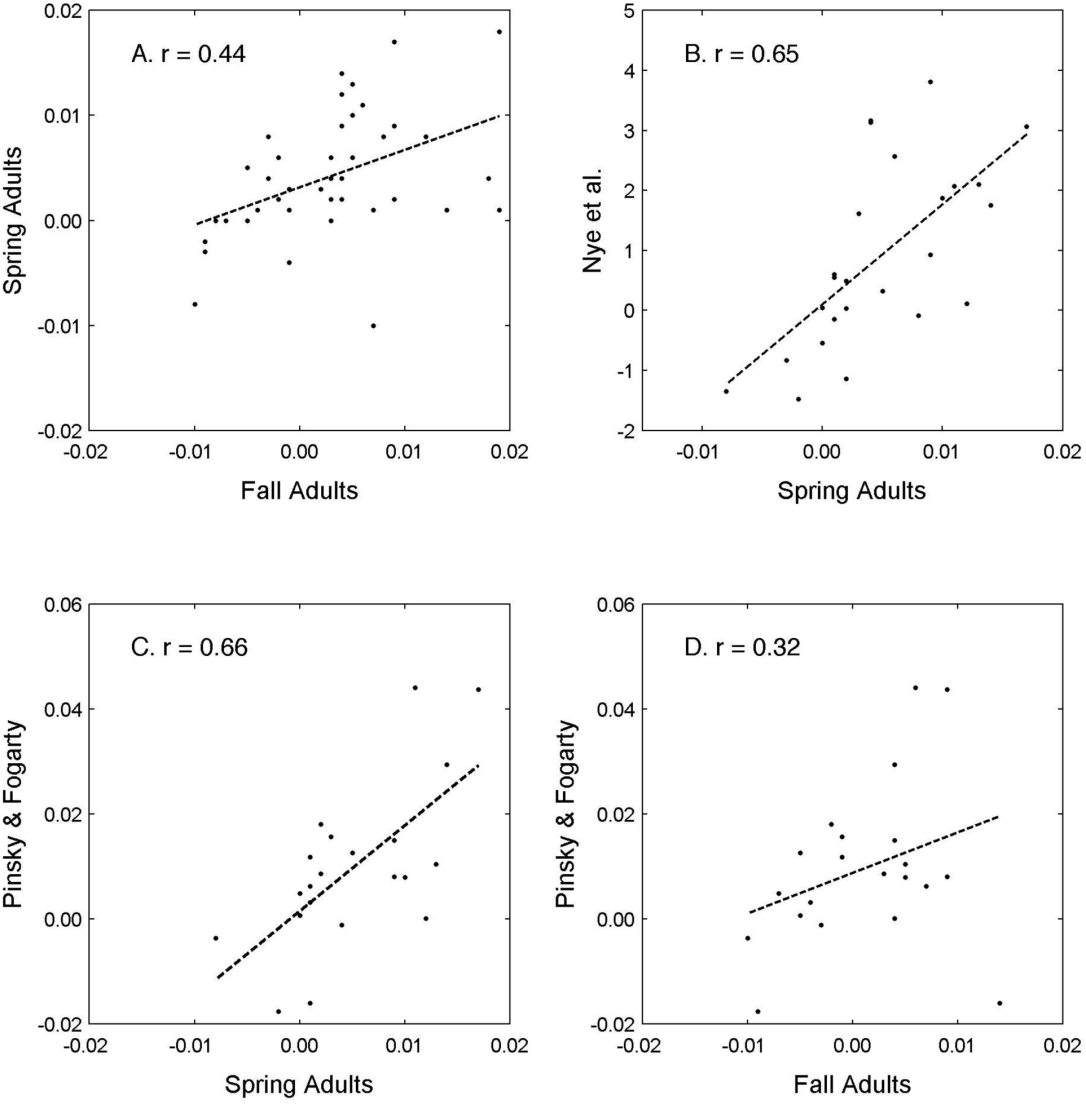
Correlation results among methods used to examine change in distribution of adult fish in the Northeast U.S. Shelf Ecosystem. Methods used to examine change in along-shelf distribution of mature adult fishes were correlated for spring to fall as measured in this study (A), spring from this study to Nye et al. [4] (B), spring from this study to Pinsky and Fogarty [49] (C), and fall from this study to Pinsky and Fogarty [49] (D). Our analyses produced a linear slope based on a spatially explicit change statistic. Nye et al. [4] calculated a linear slope from the annual change in the center of biomass in an along-shelf direction for the spring survey. Pinsky and Fogarty [49] calculated a linear slope for the annual change in the center of biomass in a north-south direction averaged across the spring and fall surveys.

Our study used a non-parametric comparison of two time periods and achieved comparable results to more powerful approaches while also allowing the comparison of two life stages. In addition, our approach allowed us to examine synchrony between larvae and adults in an effort to bridge the gap between the two life stages [28]. Many prior studies examining changes in marine fish distributions have conducted parametric linear regression of distribution statistics over time. Linear regression was not an appropriate approach for our study because the larval data used here was two large decade-long programs separated by years of less intense sampling. Furthermore, linear regression can ignore the strata-based sampling design of many trawl surveys [66], and trawl survey data is actually censored in space (e.g., northern and southern boundaries to the survey area); the distribution of a species is rarely sampled in its entirety [67]. Thus we analyzed two life stages using the same non-parametric methods. Despite the methodological differences between our study and Nye et al. [4] and Pinsky and Fogarty [32], the metrics of change are correlated (Fig 12) indicating that the non-parametric and linear regression methods are comparable. Acknowledging the limitations of non-parametric statistics, it is likely that distribution shifts have occurred for more taxa, but these shifts were not detectable using the non-parametric methods.

Changes in distribution have important implications for stock assessment and fisheries management. First, changes in larval distribution can mean changes in stock structure [56]; spawning time and location is an important component of stock structure and changes in larval distributions provides further justification for reexamining stock structure for species in the NEUS Shelf Ecosystem (see Link et al. [68]). Second, there are approximately 240 closed areas in the NEUS Shelf that are designed to protect a number of species using a variety of fishing regulations [69]; changes in distributions can impact the effectiveness of closed areas for protecting individual species [70]. Third, changes in distribution may cause changes in the structure and function of an ecosystem. The northward movement of a number of warm-temperature species in the NEUS Shelf Ecosystem will likely change trophic interactions and pathways. The increase in spawning of these species in the region may not affect the pelagic ecosystem because fish larvae are rare relative to other components of the system [71]. However, changes in prey and predators may affect the survival of larvae [16, 20], and a shift in spawning may result in a change in the productivity of a unit of spawning stock biomass (see above and Voss et al. [72]). The changes documented here provide further evidence that fish stocks in the NEUS Shelf have changed for both larvae and adults. These changes will impact the productivity and distribution of fish stocks, which has multiple implications for the assessment and management of these living marine resources.

## Supporting Information

S1 File Table and figures for all taxa examined for changes in spatial and seasonal distribution in the Northeast U.S. Shelf Ecosystem. Table A in S1 File lists the family, taxon, common name, size at maturity, and supplemental figure letters. Figs A-CG in S1 File show results for each taxon and life-stage examined.
(PDF)
