# Supplementary material for: Long-Term Changes in the Distributions of Larval and Adult Fish in the Northeast U.S. Shelf Ecosystem
Source: PLoS One. 2015 Sep 23;10(9):e0137382. doi: 10.1371/journal.pone.0137382 (PMC4580593; doi:10.1371/journal.pone.0137382)
Supplement: S1 File — Table A in S1 File lists the family, taxon, common name, size at maturity, and supplemental figure letters. Figs A-CG in S1 File show results for each taxon and life-stage examined. (PDF) [file pone.0137382.s001.pdf]

S1 Table A. List of taxa examined for changes in spatial and seasonal distribution on the Northeast U.S. Shelf Ecosystem. Distribution in the annual relative proportions among strata between 1977 - 1987 and 1999 - 2008 of larvae and spring- and fall-collected adult fish were tested separately using Kruskal-Wallis chi-square. To prevent including strata with rare collections of a taxa, only strata that made up at least 99 % of the empirical cumulative distribution were included in the analyses. The total number of strata (N) and range (south to north) is indicated for each taxa and life stage. Strata locations are shown in Fig 1: 1 – 13 = Mid-Atlantic Bight, 14 – 25 = Southern New England, 26 – 32 = Georges Bank, 33 – 47 = Gulf of Maine. By examining changes in slopes using linear analyses, changes were analyzed in: the along-shelf, the cross-shelf, and the depth directions. Results were classified into categories for each distributional shift: no significant change, southward shift, northward shift, inshore shift, offshore shift, shallower, and deeper. Seasonal changes were also examined for larvae based on annual relative proportion. The resulting change was classified as no significant change, earlier, or later based on the shift in bi-monthly proportions.

S1 File Figs are provided for all taxa and life stages analyzed. Click on the S1 File Fig hyperlink for each taxon and life stage. Blank = no data. There is a hyperlink at the bottom of each graph to get you back to S1 File Table A.

Family	Taxon	Common Name	Size at Maturity (cm)	Larval			Adult		
				Strata			Strata		
				N	Range	S1 Fig	N	Range	S1 Fig
Squalidae	*Squalus acanthias*	Spiny Dogfish	60				45	1-45	A
Rajidae	*Amblyraja radiata*	Thorny Skate	43				19	28-46	B
	Leucoraja erinacea	Little Skate	39				30	5-34	C

	Leucoraja garmani	Roseatte Skate	33				18	1-18	D
	Leucoraja ocellata	Winter Skate	53				31	6-36	E
	Malacoraja senta	Smooth Skate	33				21	26-46	F
	Raja eglanteria	Clearnose Skate	56				12	1-12	G
Clupeidae	*Brevoortia tyrannus*	Atlantic Menhaden		30	2-31	H			
	Clupea harengus	Atlantic Herring	25	23	23-45	I	40	3-42	J
Gonostomatidae	*Cyclothone* spp.	Bristlemouths		39	1-39	K			
Myctophidae	*Benthosema* spp.	Benthosema Lanternfish		37	1-37	L			
	Ceratoscopelus maderensis	Horned Lanternfish		28	4-31	M			
	Diaphus spp.	Diaphus Lanternfishes		28	1-28	N			
Gadidae	*Gadus morhua*	Atlantic Cod	43	20	16-35	O	30	17-46	P
	Melanogrammus aeglefinus	Haddock	36	28	16-43	Q	21	26-46	R
	Pollachius virens	Pollock	44	25	19-43	S	22	25-46	T
Lotidae	*Brosme brosme*	Cusk	47				19	28-46	U
	Enchelyopus cimbrius	Fourbeard Rockling	18	36	8-43	V	32	14-45	W

Phycidae	*Urophycis chuss*	Red Hake	22				36	7-42	X
	Urophycis tenuis	White Hake	32				31	15-45	Y
	Urophycis spp.	Urophycis Hakes		36	2-37	Z			
Merlucciidae	*Merluccius albidus*	Offshore Hake	23	34	7-40	AA	40	1-40	AB
	Merluccius bilinearis	Silver Hake	22	28	10-37	AC	45	1-45	AD
Lophiidae	*Lophius americanus*	Monkfish	37	38	1-38	AE	45	1-45	AF
Sebastidae	*Sebastes* spp.	Redfishes	16	37	10-46	AG	14	32-45	AH
Triglidae	*Prionotus* spp.	Searobins		29	1-29	AI			
Cottidae	*Myoxocephalus aenaeus*	Grubby		22	15-36	AJ			
	Myoxocephalus octodecemspinosus	Longhorn Sculpin	23	29	16-44	AK	28	17-44	AL
Serranidae	*Centropristis striata*	Black Sea Bass	20	25	1-25	AM	20	1-20	AN
Pomatomidae	*Pomatomus saltatrix*	Bluefish	33	17	1-17	AO	33	1-33	AP
Sparidae	*Stenotomus chrysops*	Scup	15				24	1-24	AQ
Sciaenidae	*Cynoscion regalis*	Weakfish	17	17	1-17	AR	14	3-16	AS
	Leiostomus xanthurus	Spot	17	10	2-11	AT	12	1-12	AU

	Menticirrhus spp.	Kingfishes		19	2-20	AV			
	Micropogonias undulatus	Atlantic Croaker	17	15	1-25	AW	11	2-12	AX
Labridae	*Tautoga onitis*	Tautog		33	3-35	AY			
	Tautogolabrus adspersus	Cunner		30	11-40	AZ			
Zoarcidae	*Zoarces americanus*	Ocean Pout	30				32	11-42	BA
Stichaeidae	*Ulvaria subbifurcata*	Radiated Shanny		34	13-46	BB			
Pholidae	*Pholis gunnellus*	Rock Gunnel		38	9-46	BC			
Anarhichadidae	*Anarhichas* spp.	Atlantic Wolffishes	40	31	16-46	BD	19	28-46	BE
Ammodytidae	*Ammodytes* spp.	Sand Lances	8	33	5-37	BF	31	5-35	BG
Scombridae	*Auxis* spp.	Bullet Mackerels		23	1-23	BH			
	Scomber scombrus	Atlantic Mackerel	26	29	7-35	BI	42	2-43	BJ
Stromateidae	*Peprilus* spp.	Butterfishes	12	30	2-31	BK	34	1-34	BL
Scophthalmidae	*Scophthalmus aquosus*	Windowpane	22	30	5-35	BM	30	5-34	BN
Paralichthyidae	*Citharichthys arctifrons*	Gulf Stream Flounder	6	22	2-23	BO	26	1-26	BP
	Etropus spp.	Smallmouth Flounders		23	1-23	BQ			
	Paralichthys dentatus	Summer Flounder	28	29	2-30	BR	26	1-26	BS

	Hippoglossina oblonga	American Fourspot Flounder	20	28	2-29	BT	34	1-34	BU
	Syacium spp.	Dusky Flounders		27	1-27	BV			
Bothidae	*Bothus* spp.	Eyed Flounders		28	1-28	BW			
Pleuronectidae	*Glyptocephalus cynoglossus*	Witch Flounder	31	40	4-43	BX	42	4-45	BY
	Hippoglossoides platessoides	American Plaice	36	23	16-38	BZ	19	28-46	CA
	Hippoglossus hippoglossus	Atlantic Halibut	75				24	23-46	CB
	Limanda ferruginea	Yellowtail Flounder	27	23	10-32	CC	37	10-46	CD
	Pseudopleuronectes americanus	Winter Flounder	27	23	11-33	CE	34	12-45	CF
Cynoglossidae	*Symphurus* spp.	Tonguefishes		22	1-22	CG			

S1 Fig A. Spatial distribution of adult *Squalus acanthias* (Spiny Dogfish) on the Northeast U.S. Shelf Ecosystem. Change in distribution of adult *Squalus acanthias* in the spring (A; ○) and fall (B; •) were examined in the along-shelf (C), cross-shelf (D), and depth (E) directions. *Squalus acanthias* shifted significantly southward in the fall (C).

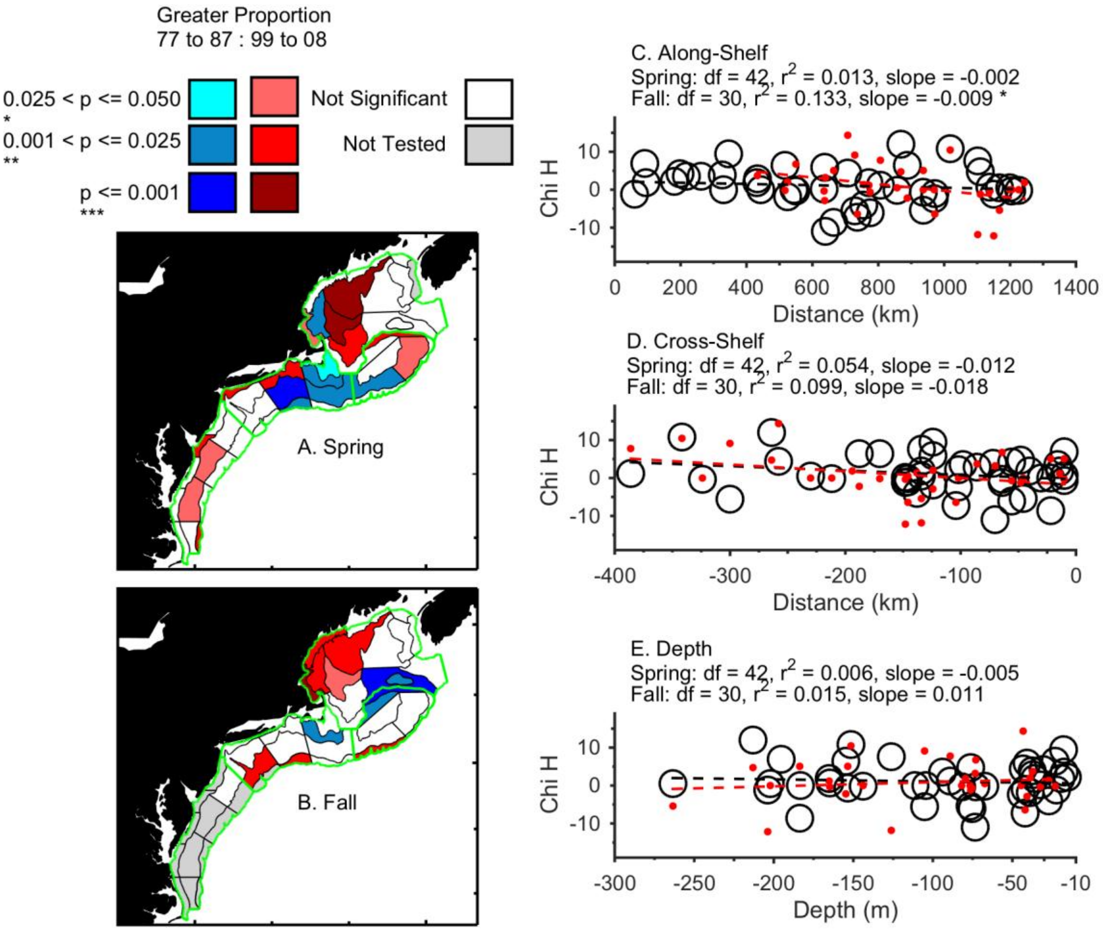

[Return to S1 File Table A](#).

S1 Fig B. Spatial distribution of adult *Amblyraja radiata* (Thorny Skate) on the Northeast U.S. Shelf Ecosystem. Change in distribution of adult *Amblyraja radiata* in the spring (A; ○) and fall (B; •) were examined in the along-shelf (C), cross-shelf (D), and depth (E) directions. *Amblyraja radiata* distribution did not significantly shift.

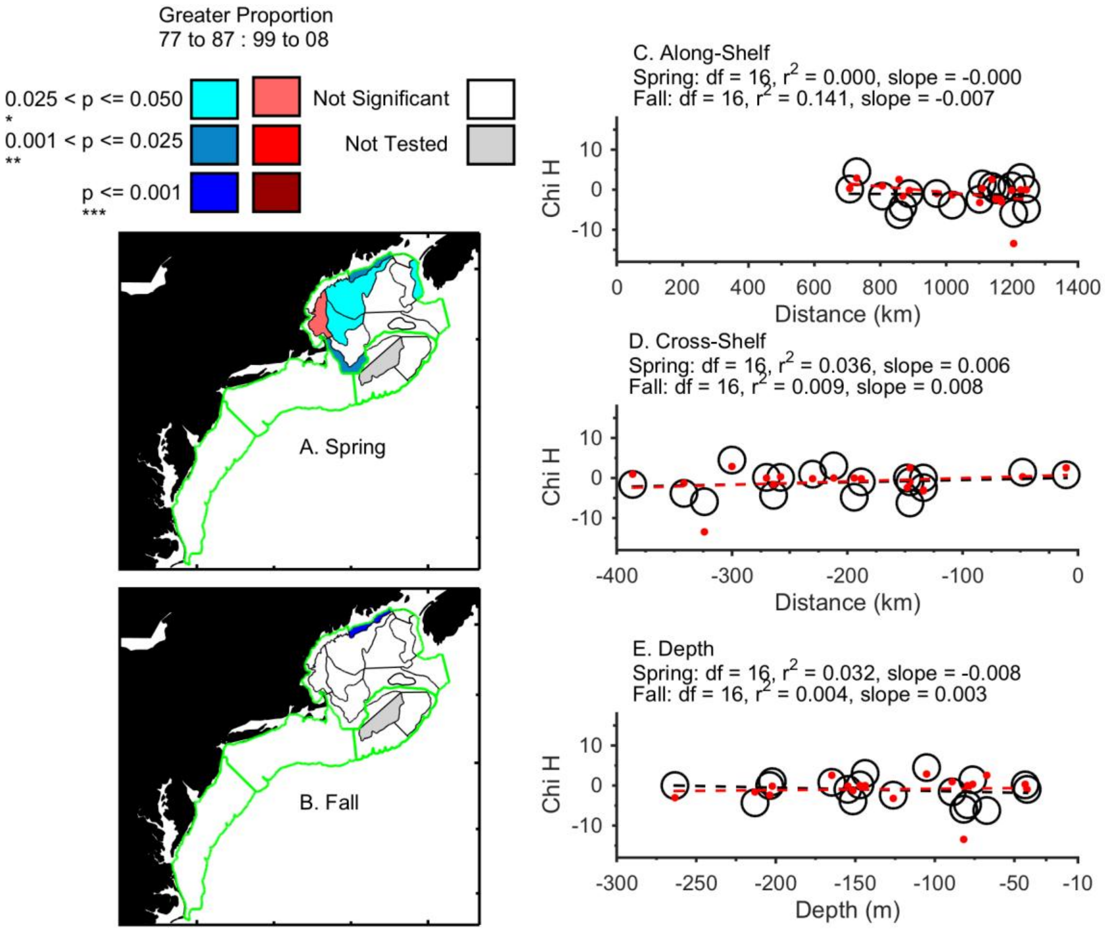

Return to S1 File Table A.

S1 Fig C. Spatial distribution of adult *Leucoraja erinacea* (Little Skate) on the Northeast U.S. Shelf Ecosystem. Change in distribution of adult *Leucoraja erinacea* in the spring (A; ○) and fall (B; •) were examined in the along-shelf (C), cross-shelf (D), and depth (E) directions. *Leucoraja erinacea* distribution significantly shifted south in the spring and fall (C).

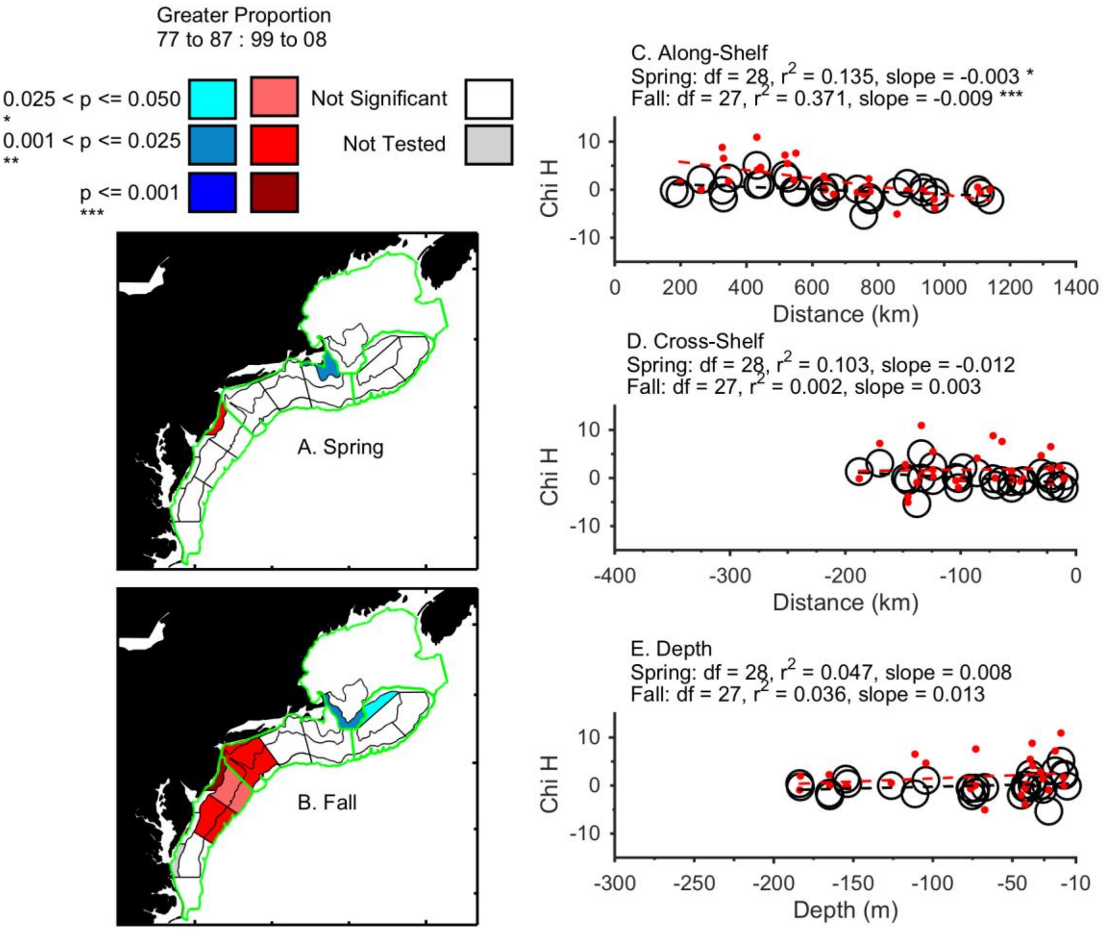

Return to S1 File Table A.

S1 Fig D. Spatial distribution of adult *Leucoraja garmani* (Roseatte Skate) on the Northeast U.S. Shelf Ecosystem. Change in distribution of adult *Leucoraja garmani* in the spring (A; ○) and fall (B; •) were examined in the along-shelf (C), cross-shelf (D), and depth (E) directions. *Leucoraja garmani* distribution did not significantly shift.

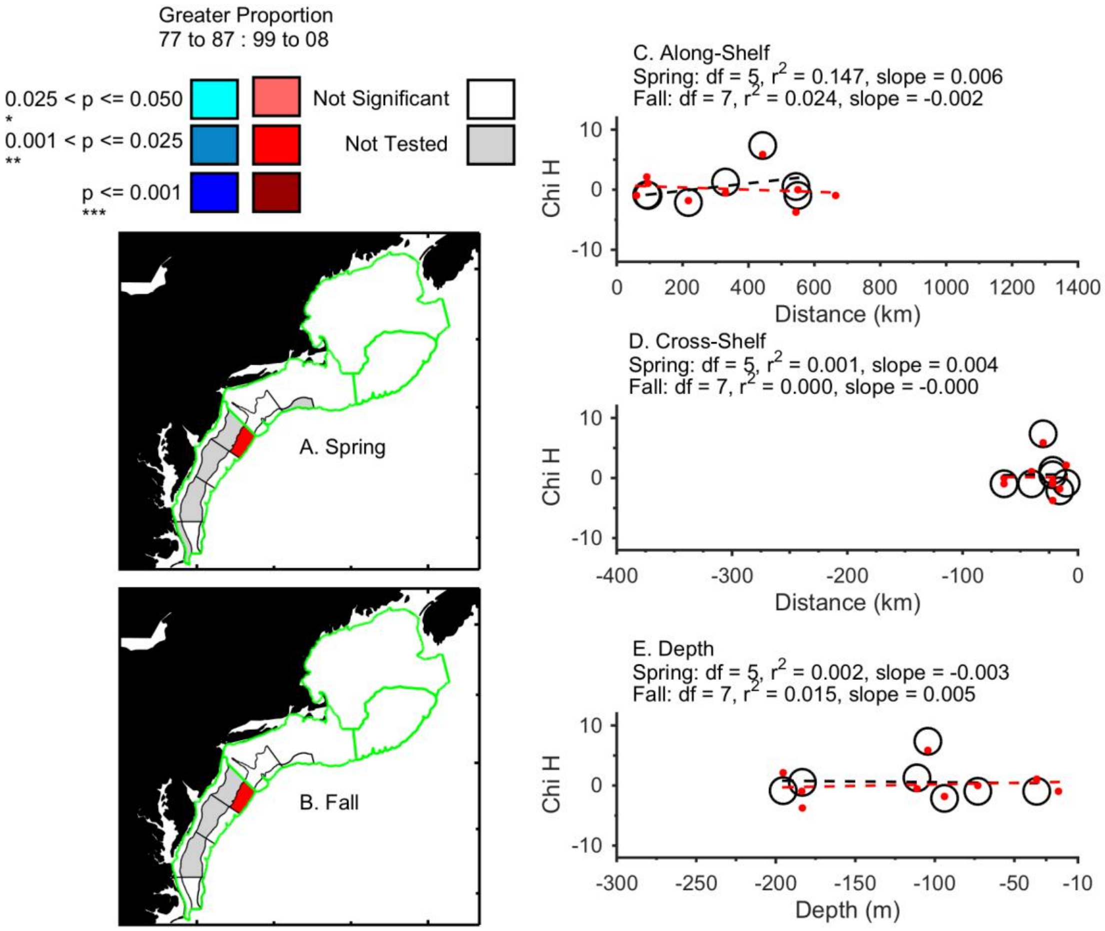

Return to S1 File Table A.

S1 Fig E. Spatial distribution of adult *Leucoraja ocellata* (Winter Skate) on the Northeast U.S. Shelf Ecosystem. Change in distribution of adult *Leucoraja ocellata* in the spring (A; ○) and fall (B; •) were examined in the along-shelf (C), cross-shelf (D), and depth (E) directions. *Leucoraja ocellata* distribution did not shift significantly.

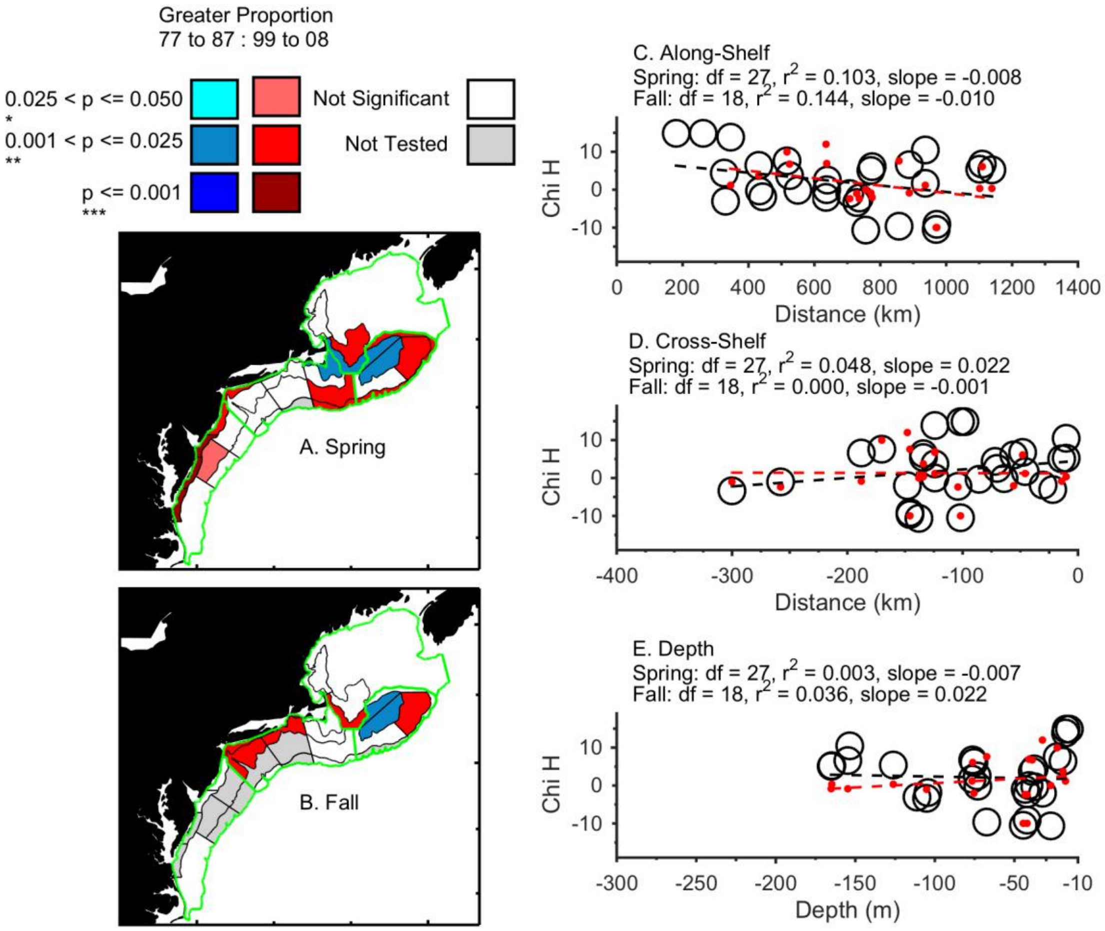

Return to S1 File Table A.

S1 Fig F. Spatial distribution of adult *Malacoraja senta* (Smooth Skate) on the Northeast U.S. Shelf Ecosystem. Change in distribution of adult *Malacoraja senta* in the spring (A; ○) and fall (B; •) were examined in the along-shelf (C), cross-shelf (D), and depth (E) directions. *Malacoraja senta* distribution shifted significantly northward in the fall (C).

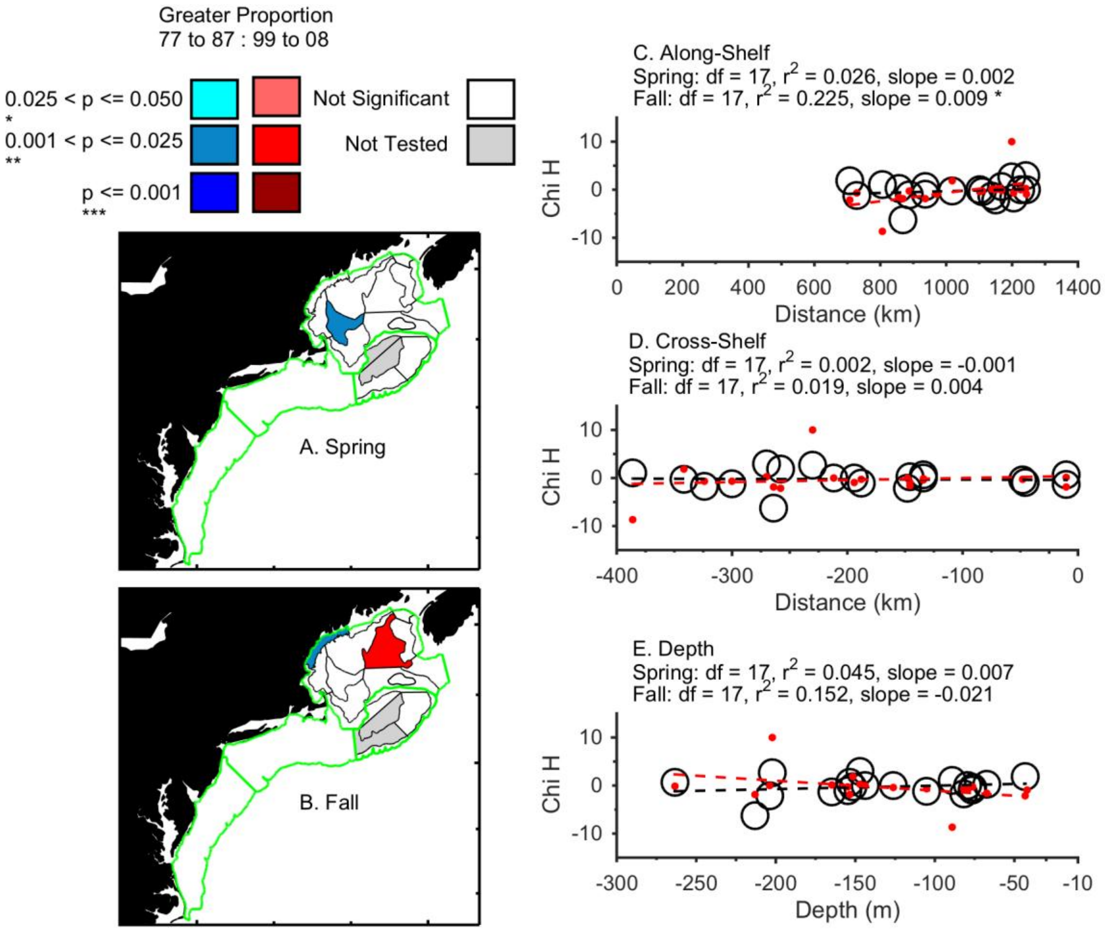

Return to S1 File Table A.

S1 Fig G. Spatial distribution of adult *Raja eglanteria* (Clearnose Skate) on the Northeast U.S. Shelf Ecosystem. Change in distribution of adult *Raja eglanteria* in the spring (A; ○) and fall (B; •) were examined in the along-shelf (C), cross-shelf (D), and depth (E) directions. *Raja eglanteria* distribution shifted significantly deeper in the fall (E).

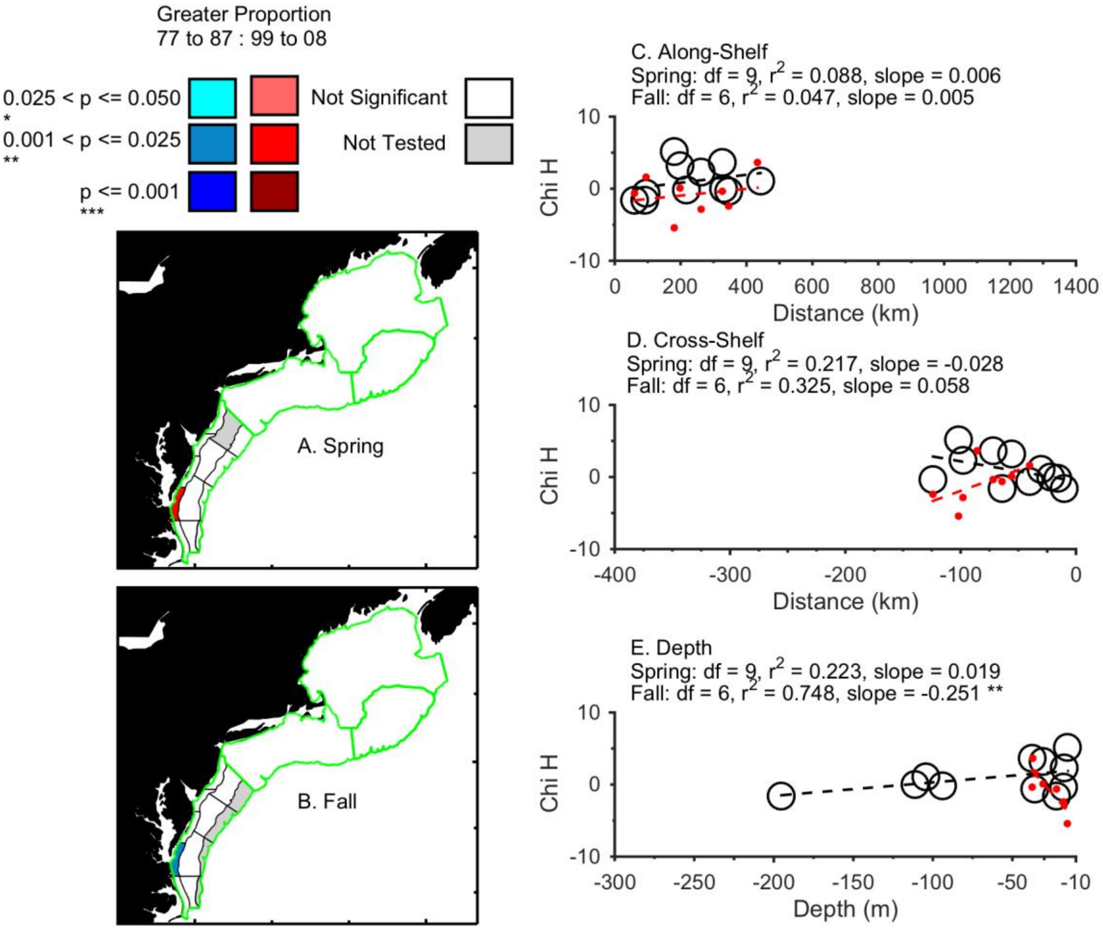

Return to S1 File Table A.

S1 Fig H. Spatial and seasonal distribution of larval *Brevoortia tyrannus* (Atlantic Menhaden) on the Northeast U.S. Shelf Ecosystem. Change in spatial distribution of larval *Brevoortia tyrannus* in the September - October (A; •), November – December (B; ○), January – February (C; •), and March - April (D; •) were examined in the along-shelf (E), cross-shelf (F), and depth (G) directions. *Brevoortia tyrannus* spatial distribution did not significantly shift. Seasonal distribution shifted significantly later (H).

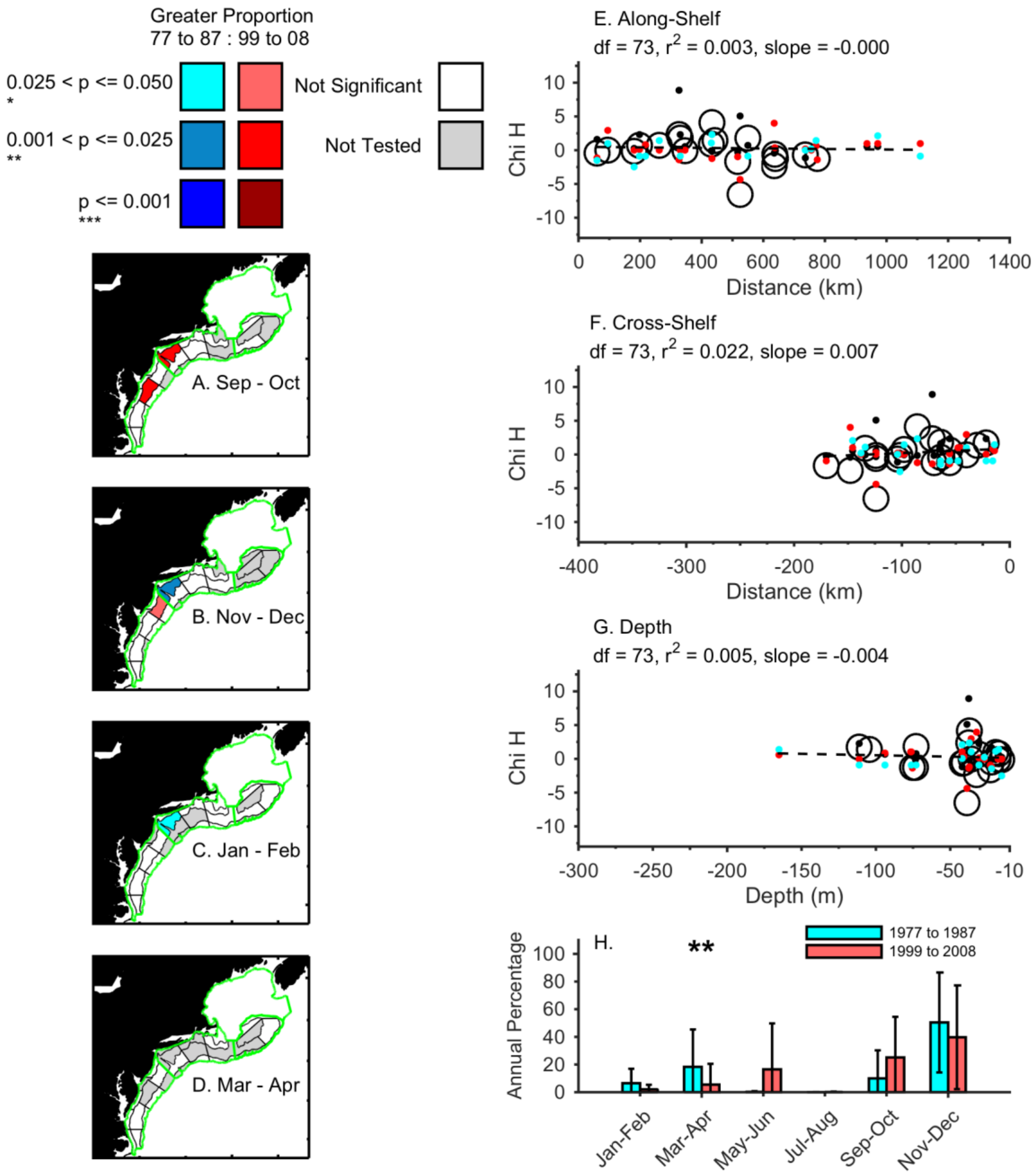

Return to S1 File Table A.

S1 Fig I. Spatial and seasonal distribution of larval *Clupea harengus* (Atlantic Herring) on the Northeast U.S. Shelf Ecosystem. Change in spatial distribution of larval *Clupea harengus* in the September - October (A; •), November – December (B; ○), and January – February (C; •) were examined in the along-shelf (D), cross-shelf (E), and depth (F) directions. Larval *Clupea harengus* shifted significantly offshore (E). Seasonal distribution did not significantly change (G).

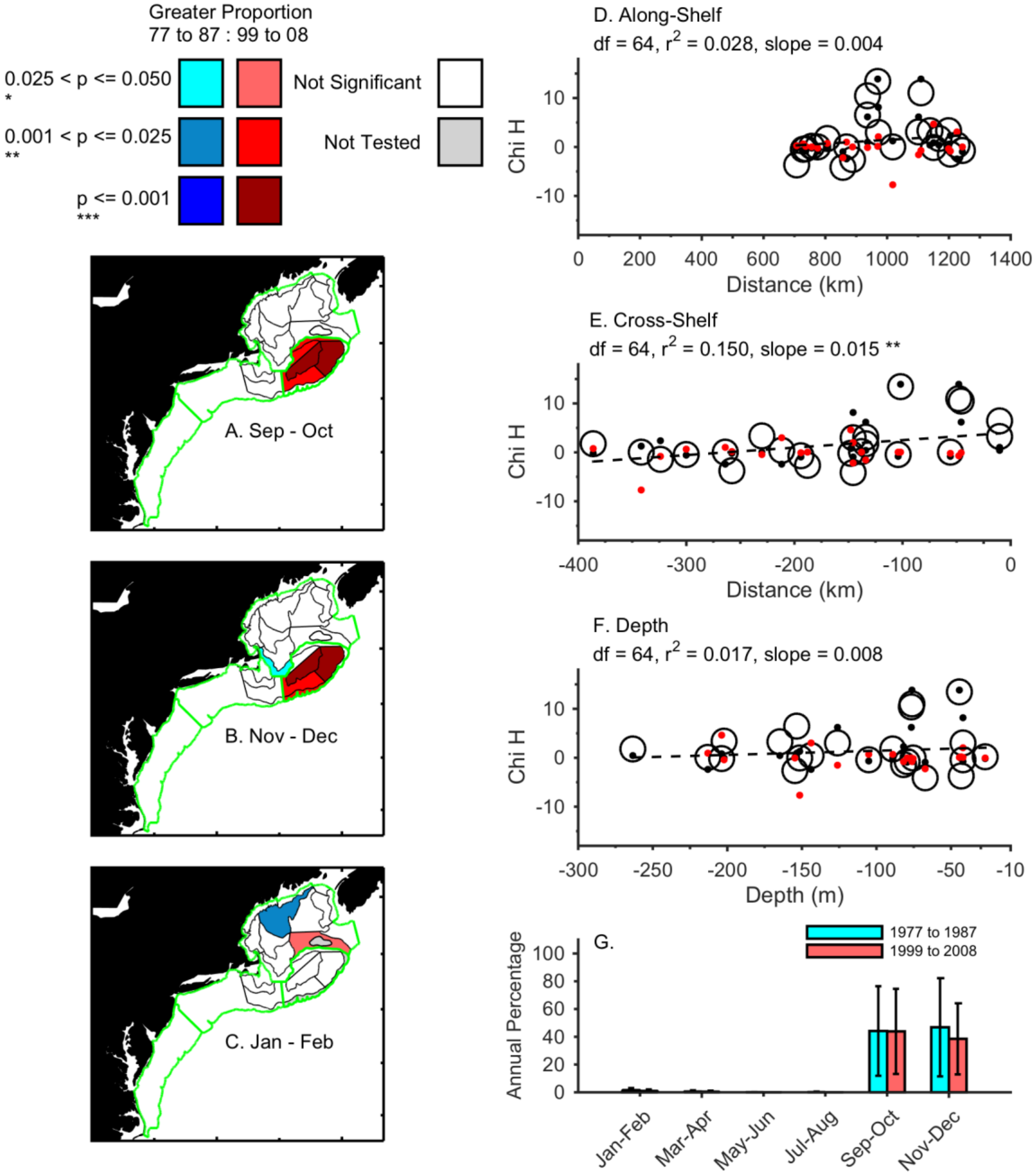

Return to S1 File Table A.

S1 Fig J. Spatial distribution of adult *Clupea harengus* (Atlantic Herring) on the Northeast U.S. Shelf Ecosystem. Change in distribution of adult *Clupea harengus* in the spring (A; ○) and fall (B; •) were examined in the along-shelf (C), cross-shelf (D), and depth (E) directions. *Clupea harengus* distribution shifted significantly offshore in the spring (D) and deeper in the fall (E).

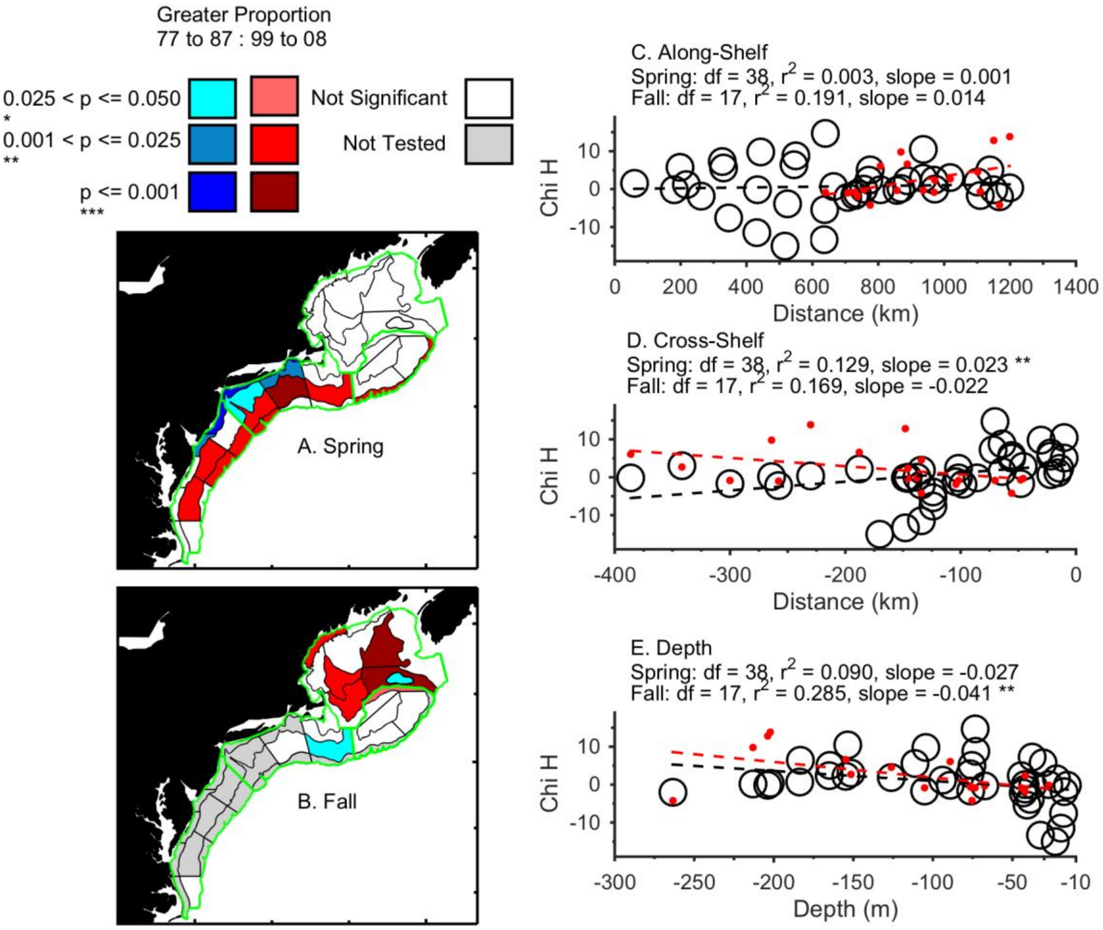

Return to S1 File Table A.

S1 Fig K. Spatial and seasonal distribution of larval *Cyclothone* spp. (Bristlemouths) on the Northeast U.S. Shelf Ecosystem. Change in spatial distribution of larval *Cyclothone* spp. in May - June (A; •), July - August (B; ○), and September - October (C; •) were examined in the along-shelf (D), cross-shelf (E), and depth (F) directions. Larval *Cyclothone* spp. did not shift significantly spatially or seasonally (G).

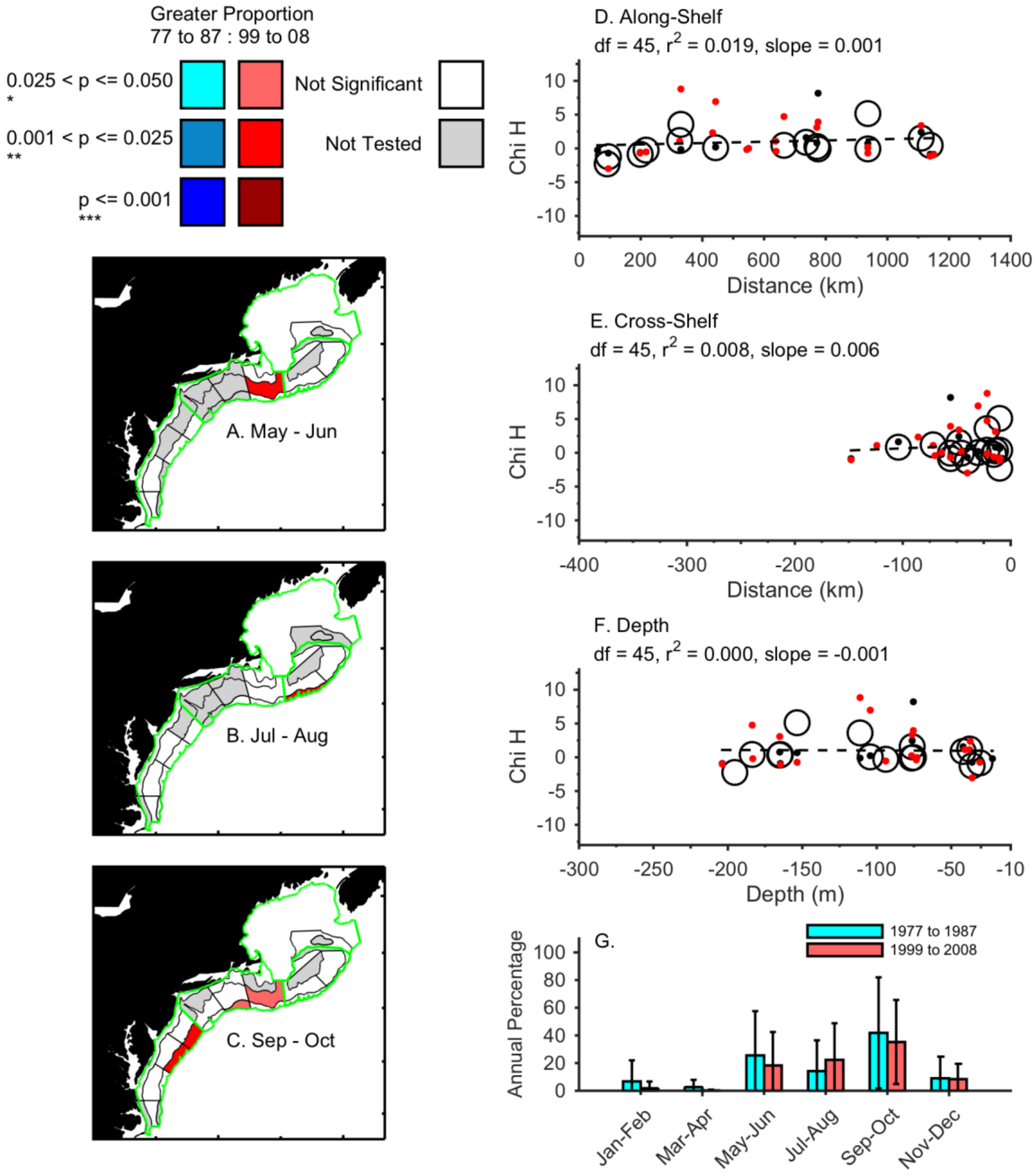

Return to S1 File Table A.

S1 Fig L. Spatial and seasonal distribution of larval *Benthosema* spp. (Benthosema Lanternfish) on the Northeast U.S. Shelf Ecosystem. Change in spatial distribution of larval *Benthosema* spp. in the November – December (A; •), January – February (B; ○), March - April (C; •), and May – June (D; •) were examined in the along-shelf (E), cross-shelf (F), and depth (G) directions. *Benthosema* spp. spatial distribution did not significantly shift. Seasonal distribution shifted significantly earlier (H).

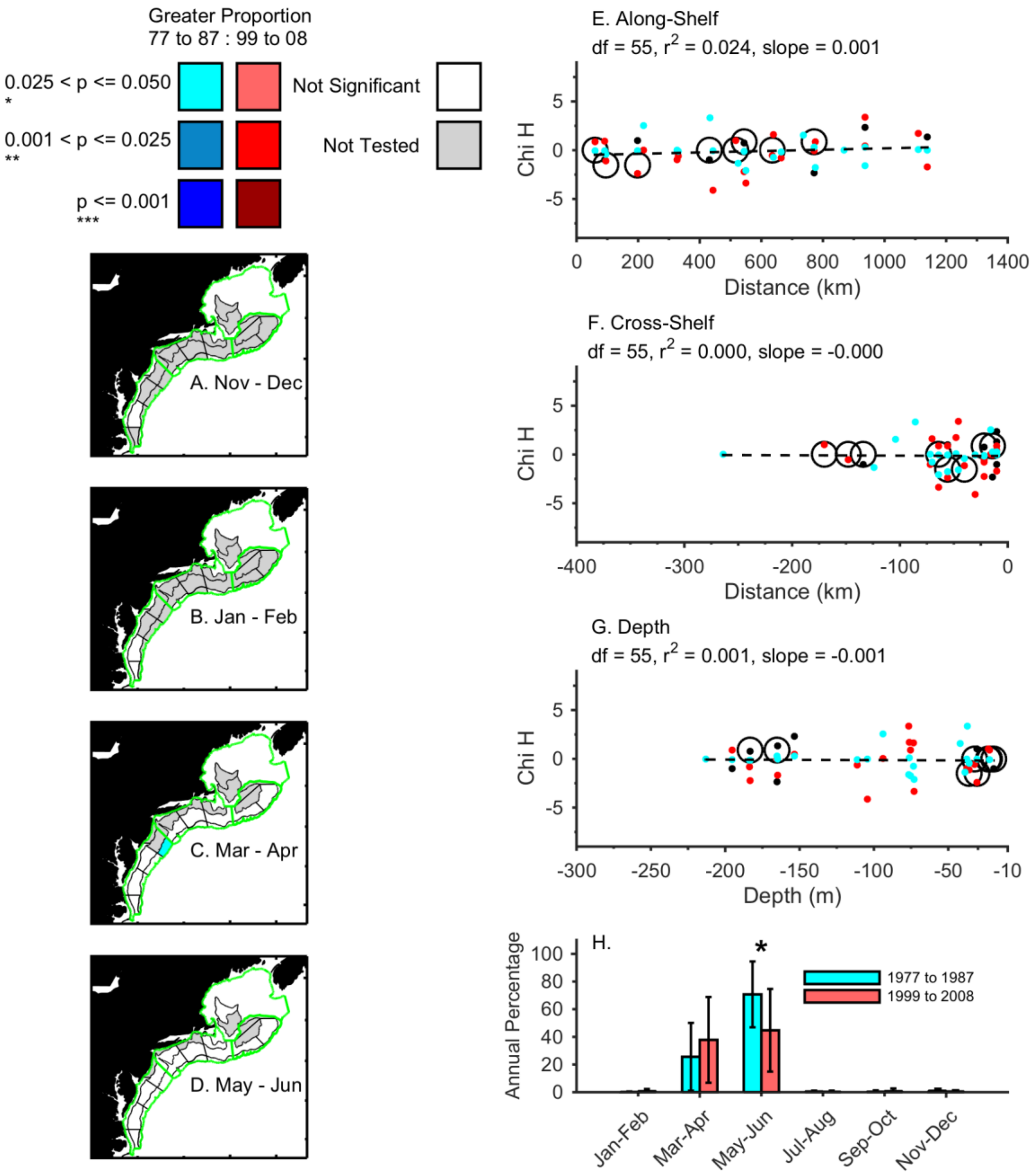

Return to S1 File Table A.

S1 Fig M. Spatial and seasonal distribution of larval *Ceratoscopelus maderensis* (Horned Lanternfish) on the Northeast U.S. Shelf Ecosystem. Change in spatial distribution of larval *Ceratoscopelus maderensis* in the May – June (A; •), July – August (B; O), and September - October (C; •) were examined in the along-shelf (D), cross-shelf (E), and depth (F) directions. *Ceratoscopelus maderensis* shifted significantly northward (D). Seasonal distribution did not significantly change (G).

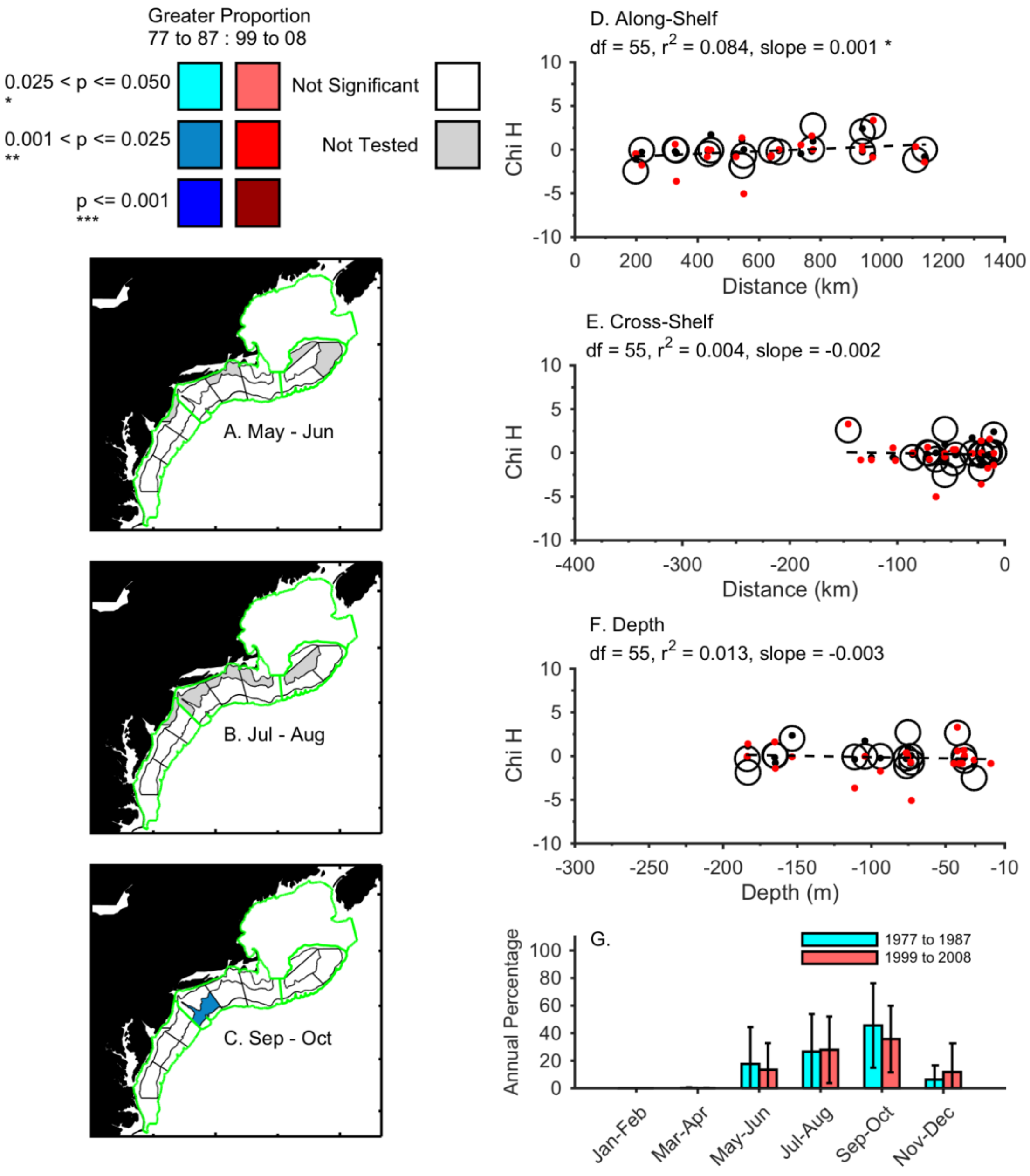

Return to S1 File Table A.

S1 Fig N. Spatial and seasonal distribution of larval *Diaphus* spp. (Diaphus Lanternfish) on the Northeast U.S. Shelf Ecosystem. Change in spatial distribution of larval *Diaphus* spp. in May - June (A; •), July - August (B; ○), September - October (C; •), and November – December (D; •) were examined in the along-shelf (E), cross-shelf (F), and depth (G) directions. Larval *Diaphus* spp. did not shift significantly spatially or seasonally (H).

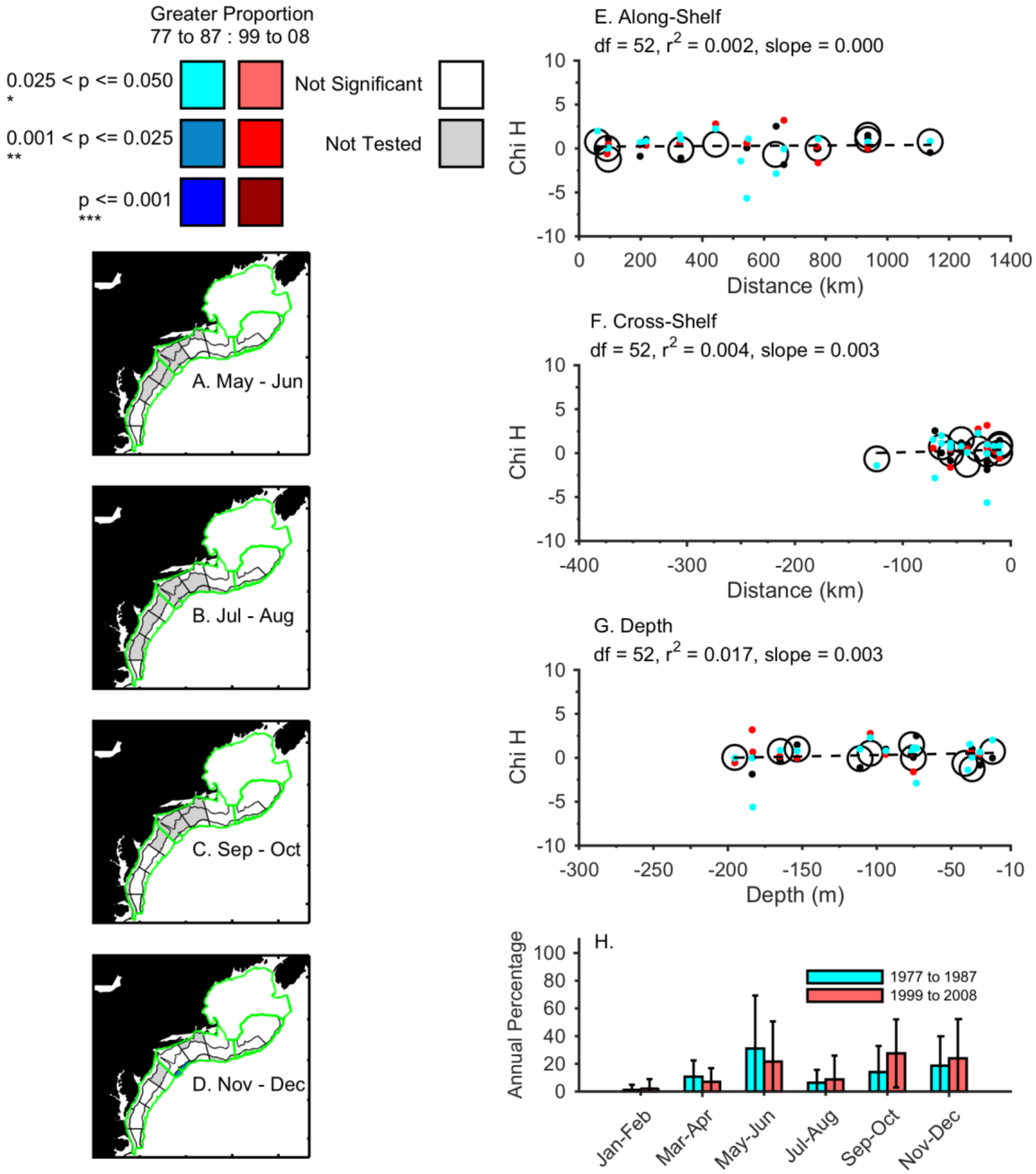

Return to S1 File Table A.

S1 Fig O. Spatial and seasonal distribution of larval *Gadus morhua* (Atlantic Cod) on the Northeast U.S. Shelf Ecosystem. Change in spatial distribution of larval *Gadus morhua* in the January – February (A; •), March – April (B; ○), and May - June (C; •) were examined in the along-shelf (D), cross-shelf (E), and depth (F) directions. *Gadus morhua* shifted significantly northward (D) and deeper (F). Seasonal distribution did not significantly change (G).

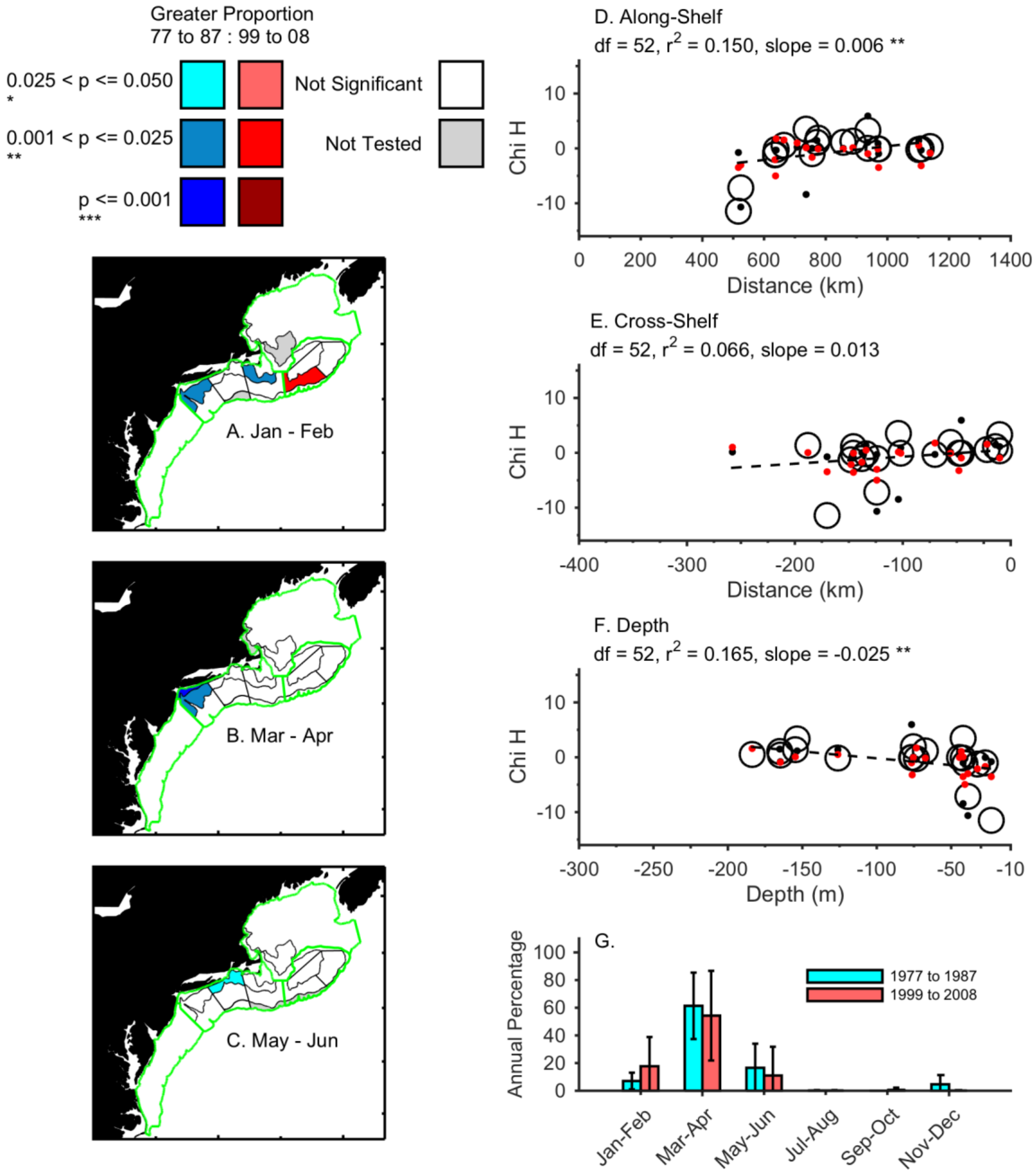

Return to S1 File Table A.

S1 Fig P. Spatial distribution of adult *Gadus morhua* (Atlantic Cod) on the Northeast U.S. Shelf Ecosystem. Change in distribution of adult *Gadus morhua* in the spring (A; ○) and fall (B; •) were examined in the along-shelf (C), cross-shelf (D), and depth (E) directions. *Gadus morhua* distribution shifted significantly deeper in the spring (E).

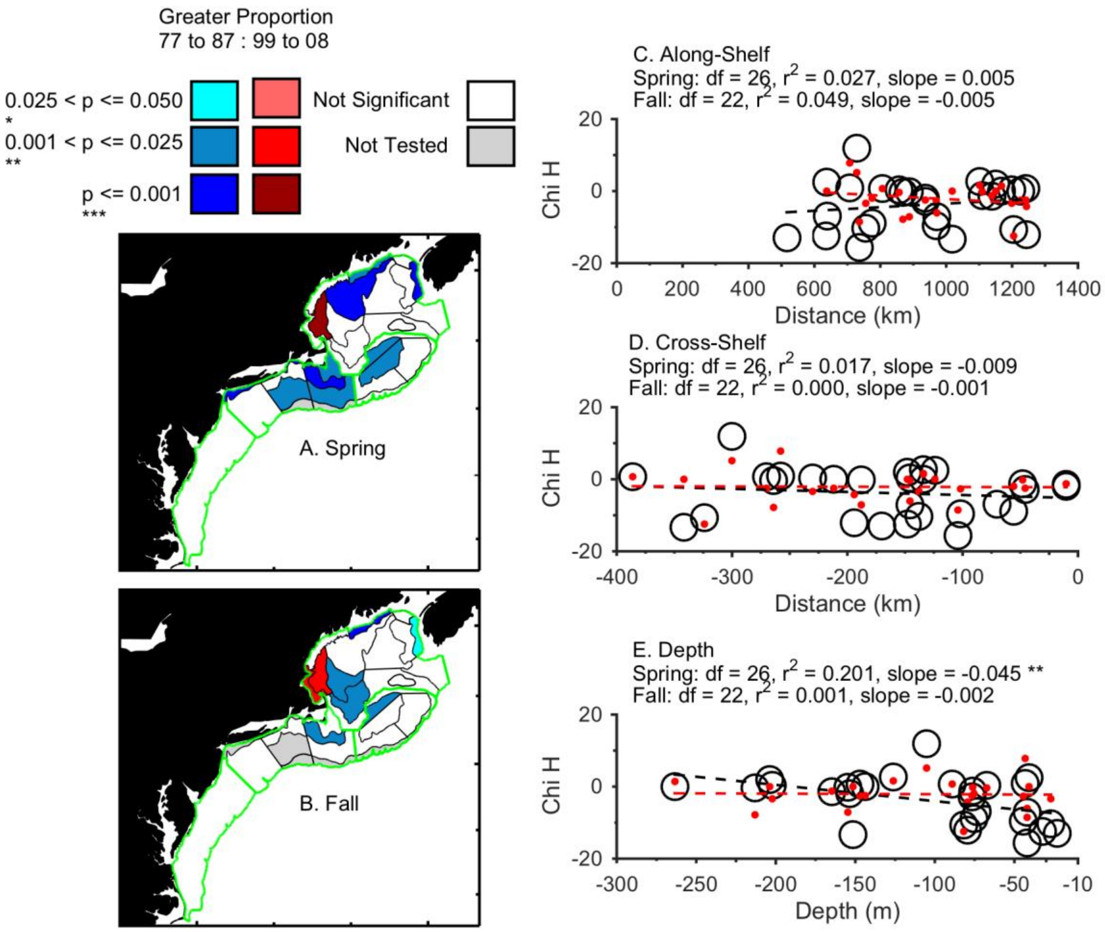

[Return to S1 File Table A](#).

S1 Fig Q. Spatial and seasonal distribution of larval *Melanogrammus aeglefinus* (Haddock) on the Northeast U.S. Shelf Ecosystem. Change in spatial distribution of larval *Melanogrammus aeglefinus* in the January – February (A; •), March – April (B; ○), and May - June (C; •) were examined in the along-shelf (D), cross-shelf (E), and depth (F) directions. Larval *Melanogrammus aeglefinus* distribution did not shift significantly. Seasonal distribution shifted significantly earlier (G).

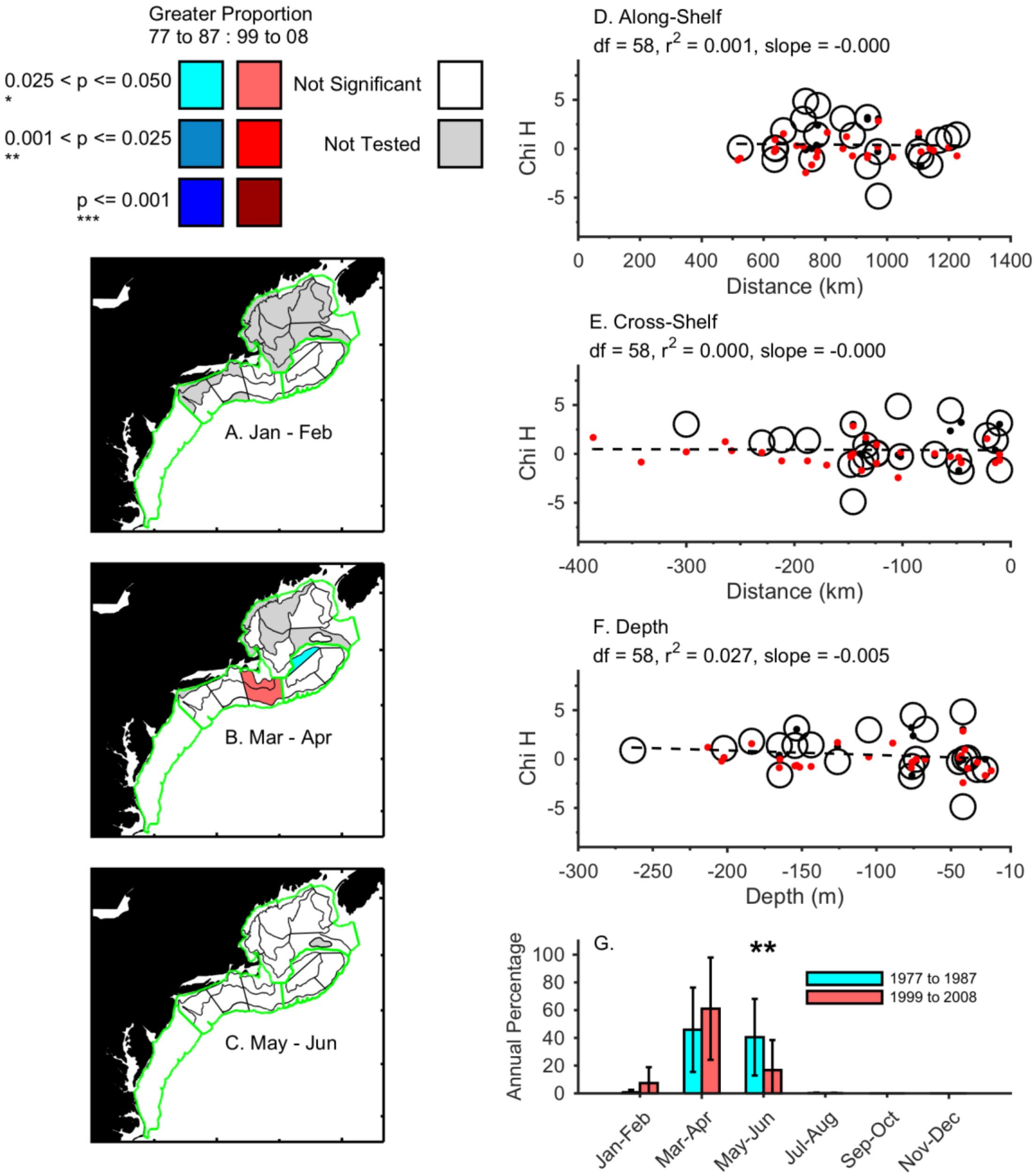

Return to S1 File Table A.

S1 Fig R. Spatial distribution of adult *Melanogrammus aeglefinus* (Haddock) on the Northeast U.S. Shelf Ecosystem. Change in distribution of adult *Melanogrammus aeglefinus* in the spring (A; ○) and fall (B; •) were examined in the along-shelf (C), cross-shelf (D), and depth (E) directions. *Melanogrammus aeglefinus* distribution did not shift significantly.

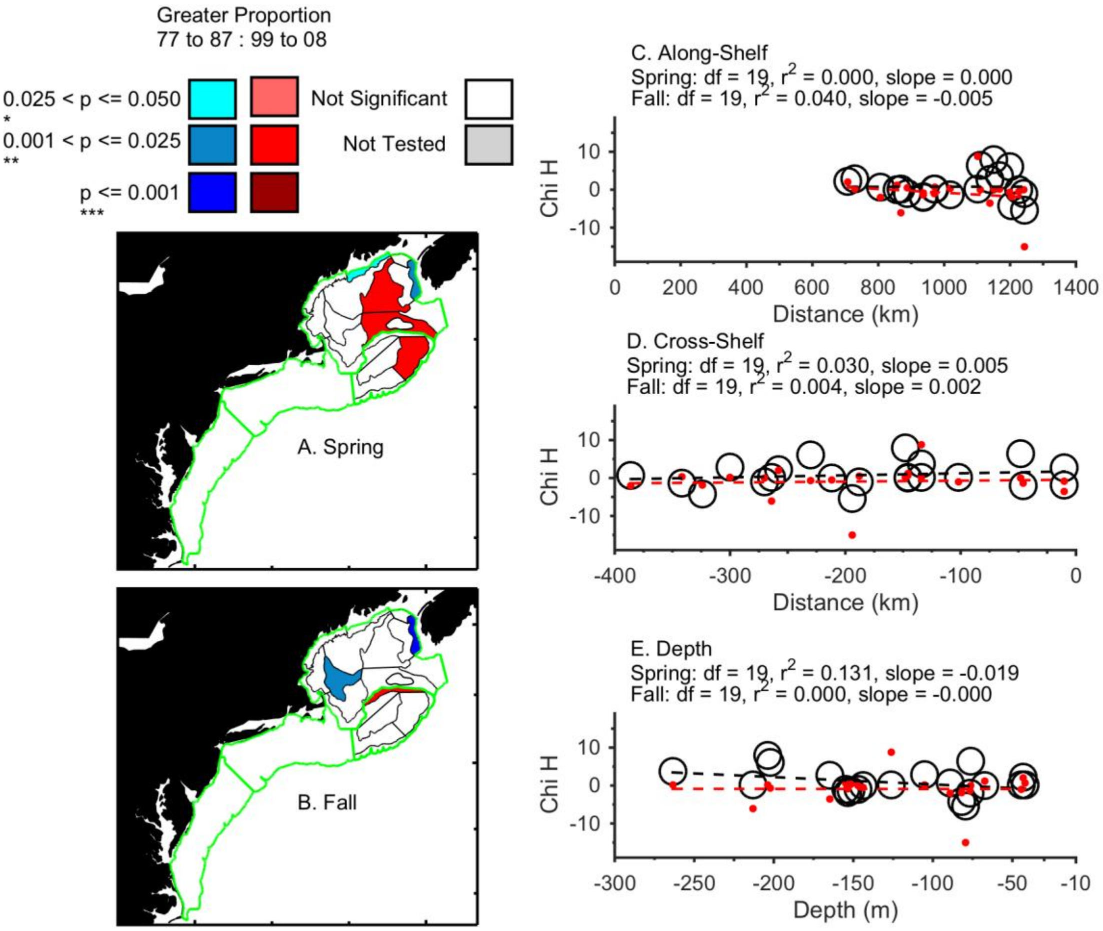

Return to S1 File Table A.

S1 Fig S. Spatial and seasonal distribution of larval *Pollachius virens* (Pollock) on the Northeast U.S. Shelf Ecosystem. Change in spatial distribution of larval *Pollachius virens* in the November – December (A; •), January – February (B; O), and March - April (C; •) were examined in the along-shelf (D), cross-shelf (E), and depth (F) directions. Larval *Pollachius virens* shifted significantly offshore (E) and shallower (F). Seasonal distribution shifted significantly later (G).

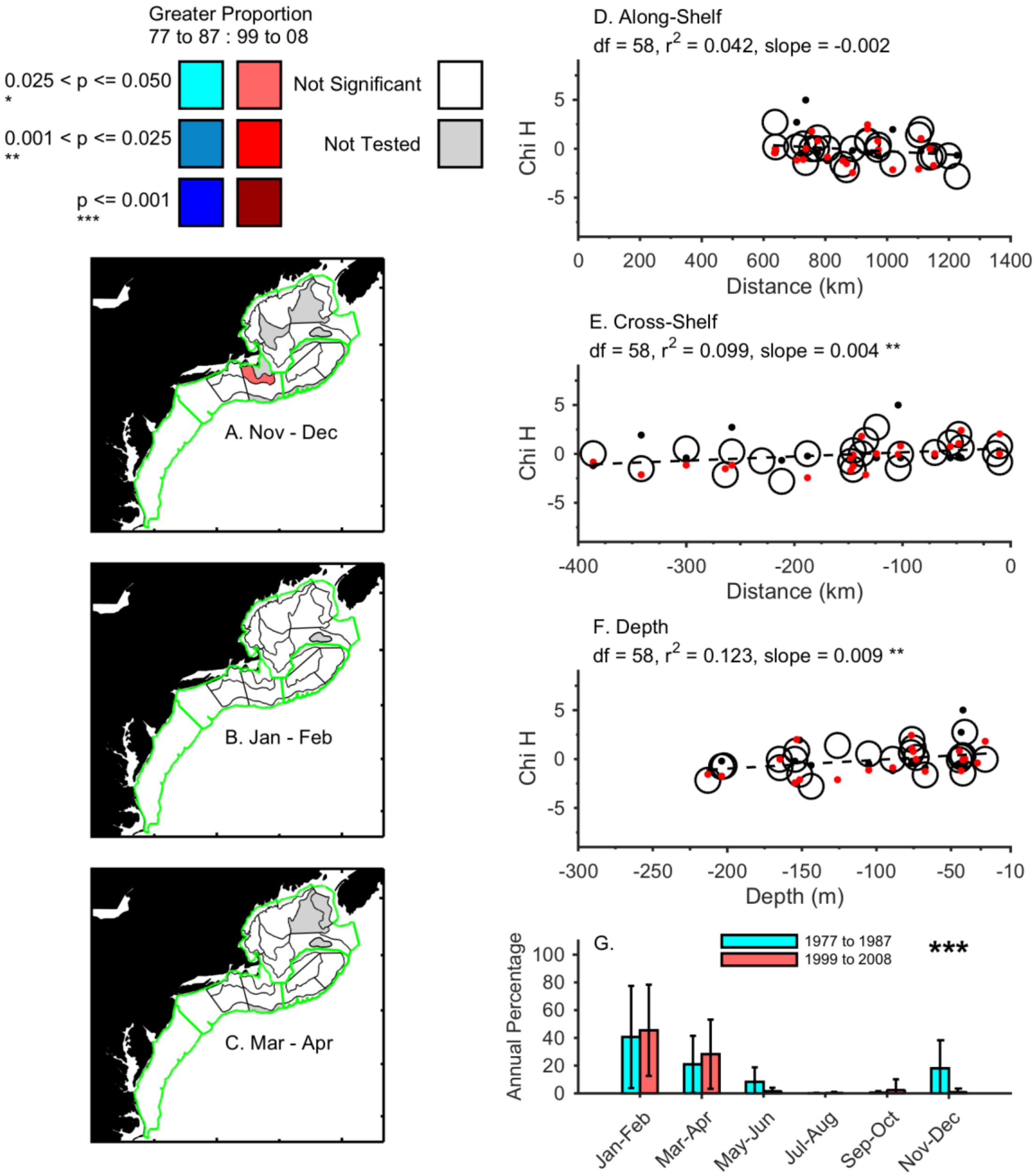

[Return to S1 File Table A](#).

S1 Fig T. Spatial distribution of adult *Pollachius virens* (Pollock) on the Northeast U.S. Shelf Ecosystem. Change in distribution of adult *Pollachius virens* in the spring (A; ○) and fall (B; •) were examined in the along-shelf (C), cross-shelf (D), and depth (E) directions. *Pollachius virens* distribution shifted significantly deeper in the spring and fall (E).

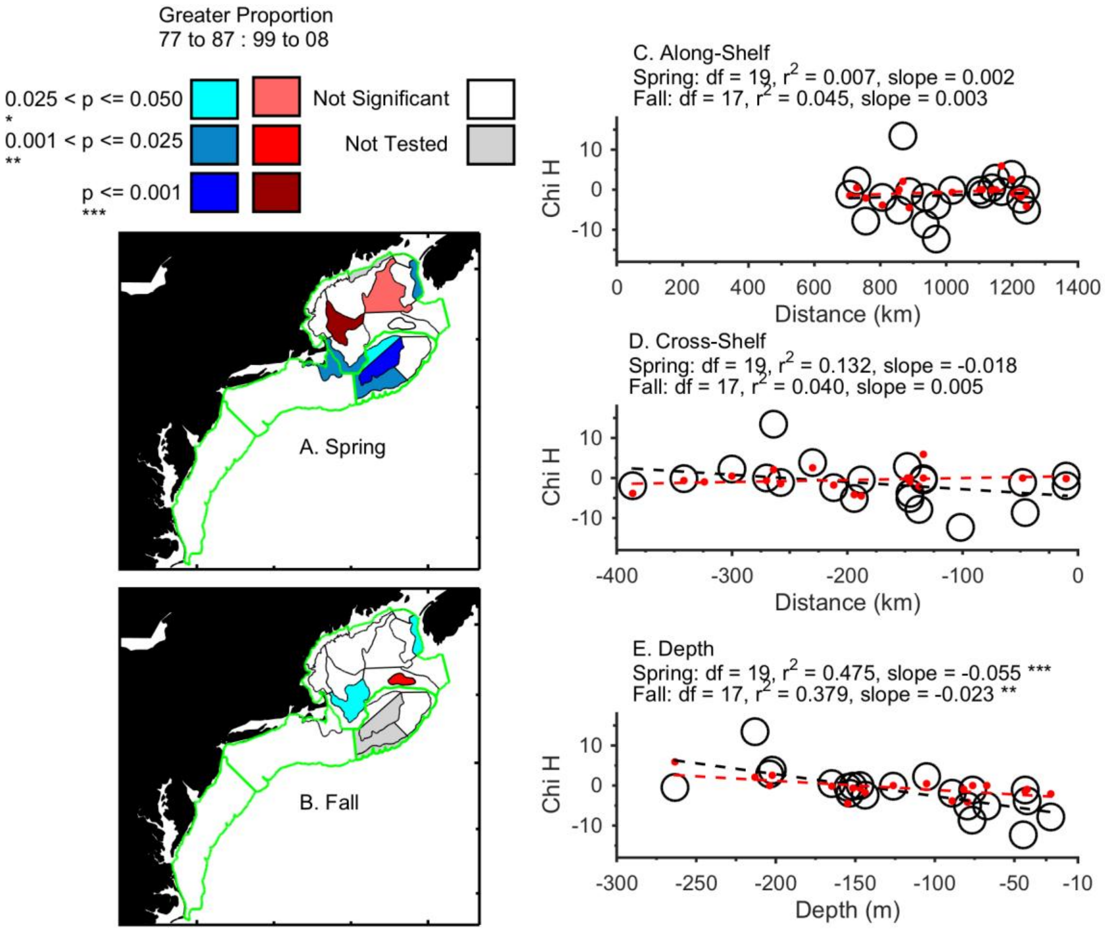

Return to S1 File Table A.

S1 Fig U. Spatial distribution of adult *Brosme brosme* (Cusk) on the Northeast U.S. Shelf Ecosystem. Change in distribution of adult *Brosme brosme* in the spring (A; ○) and fall (B; •) were examined in the along-shelf (C), cross-shelf (D), and depth (E) directions. *Brosme brosme* did not shift significantly.

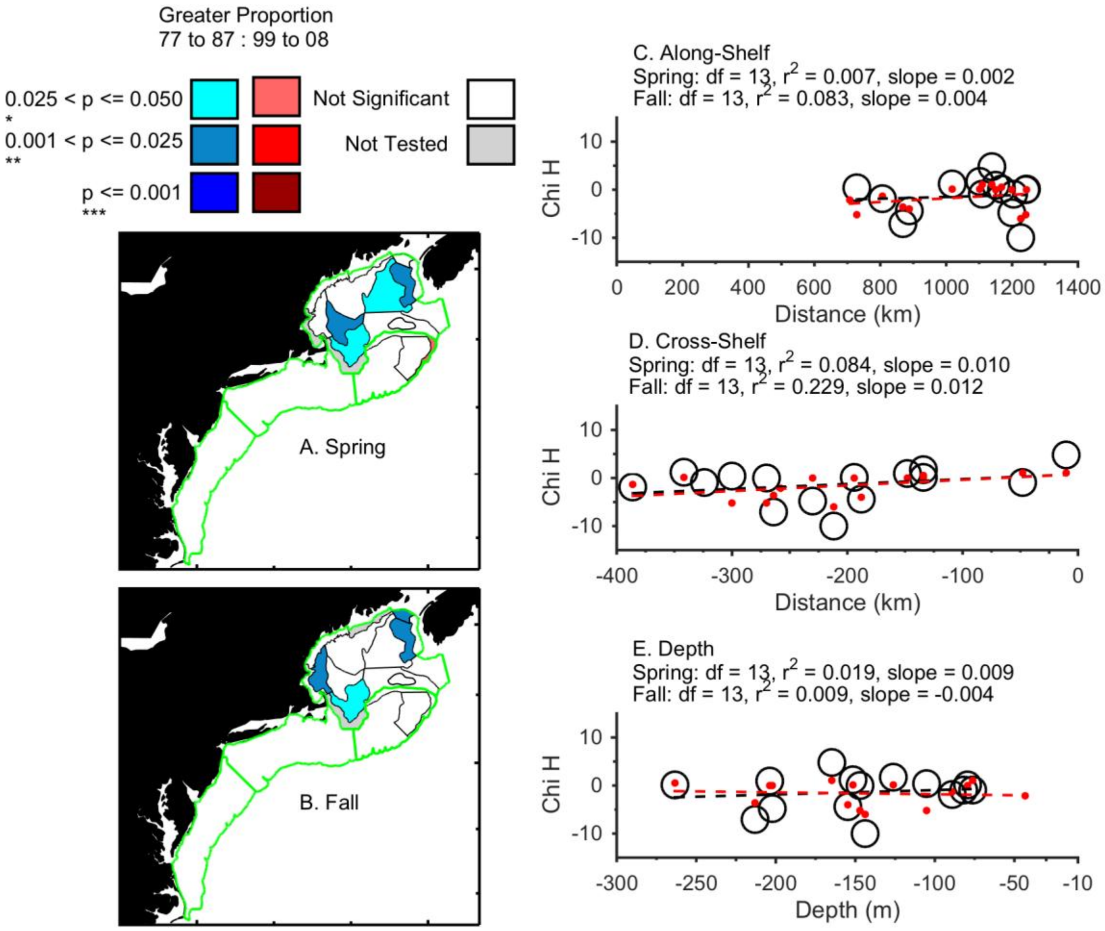

Return to S1 File Table A.

S1 Fig V. Spatial and seasonal distribution of larval *Enchelyopus cimbrius* (Fourbeard Rockling) on the Northeast U.S. Shelf Ecosystem. Change in spatial distribution of larval *Enchelyopus cimbrius* in the May – June (A; •), July – August (B; ○), and September - October (C; •) were examined in the along-shelf (D), cross-shelf (E), and depth (F) directions. Larval *Enchelyopus cimbrius* shifted significantly northward (D). Seasonal distribution shifted significantly earlier (G).

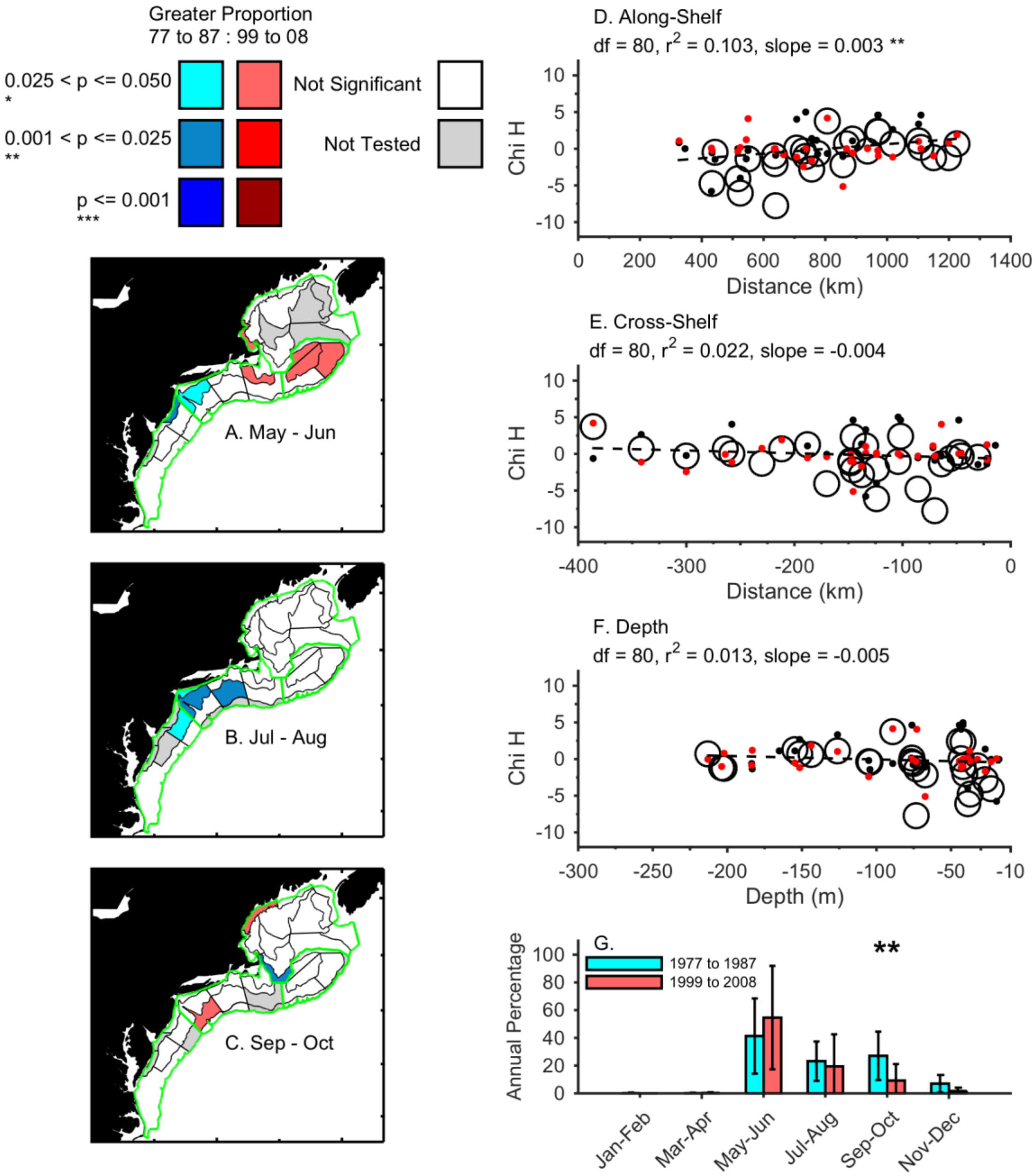

Return to S1 File Table A.

S1 Fig W. Spatial distribution of adult *Enchelyopus cimbrius* (Fourbeard Rockling) on the Northeast U.S. Shelf Ecosystem. Change in distribution of adult *Enchelyopus cimbrius* in the spring (A; ○) and fall (B; •) were examined in the along-shelf (C), cross-shelf (D), and depth (E) directions. *Enchelyopus cimbrius* did not shift significantly.

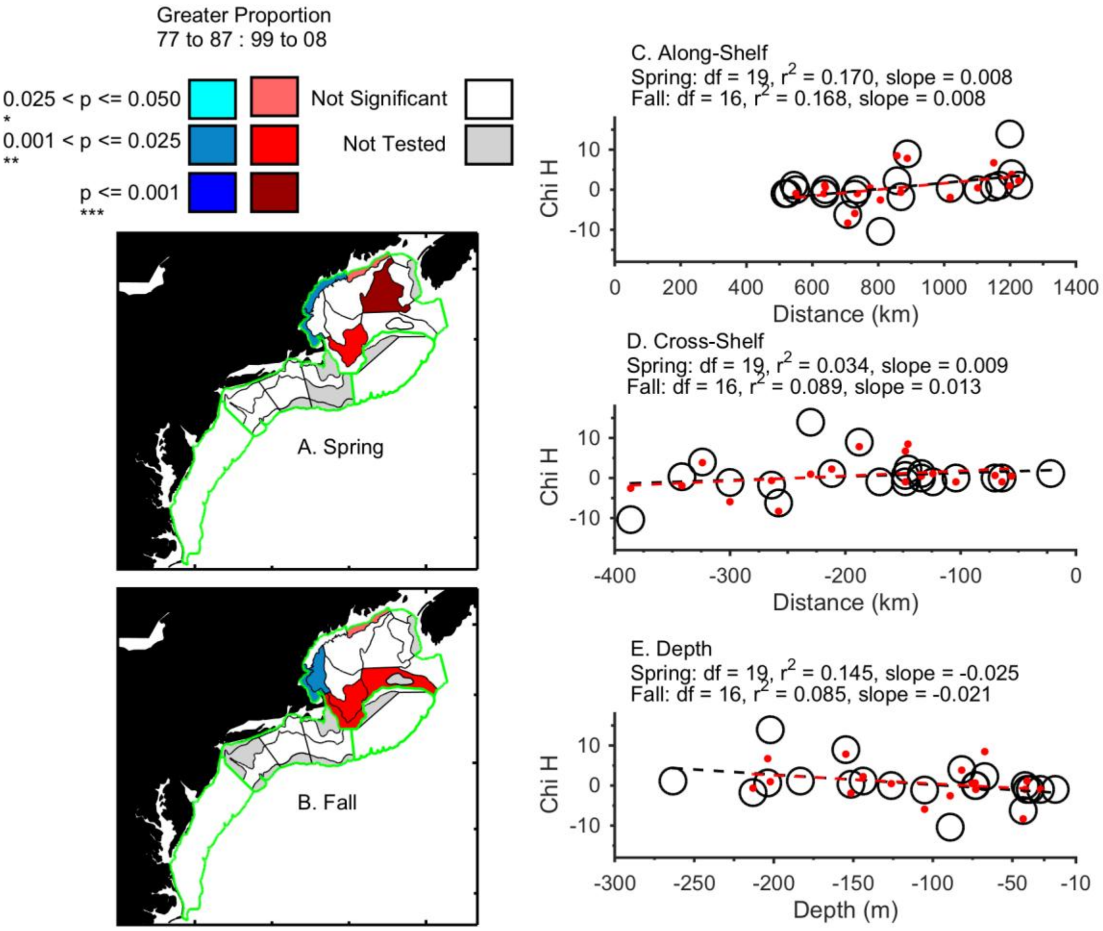

Return to S1 File Table A.

S1 Fig X. Spatial distribution of adult *Urophycis chuss* (Red Hake) on the Northeast U.S. Shelf Ecosystem. Change in distribution of adult *Urophycis chuss* in the spring (A; ○) and fall (B; •) were examined in the along-shelf (C), cross-shelf (D), and depth (E) directions. *Urophycis chuss* shifted significantly northward (C), inshore (D), and deeper (E) in the spring and fall.

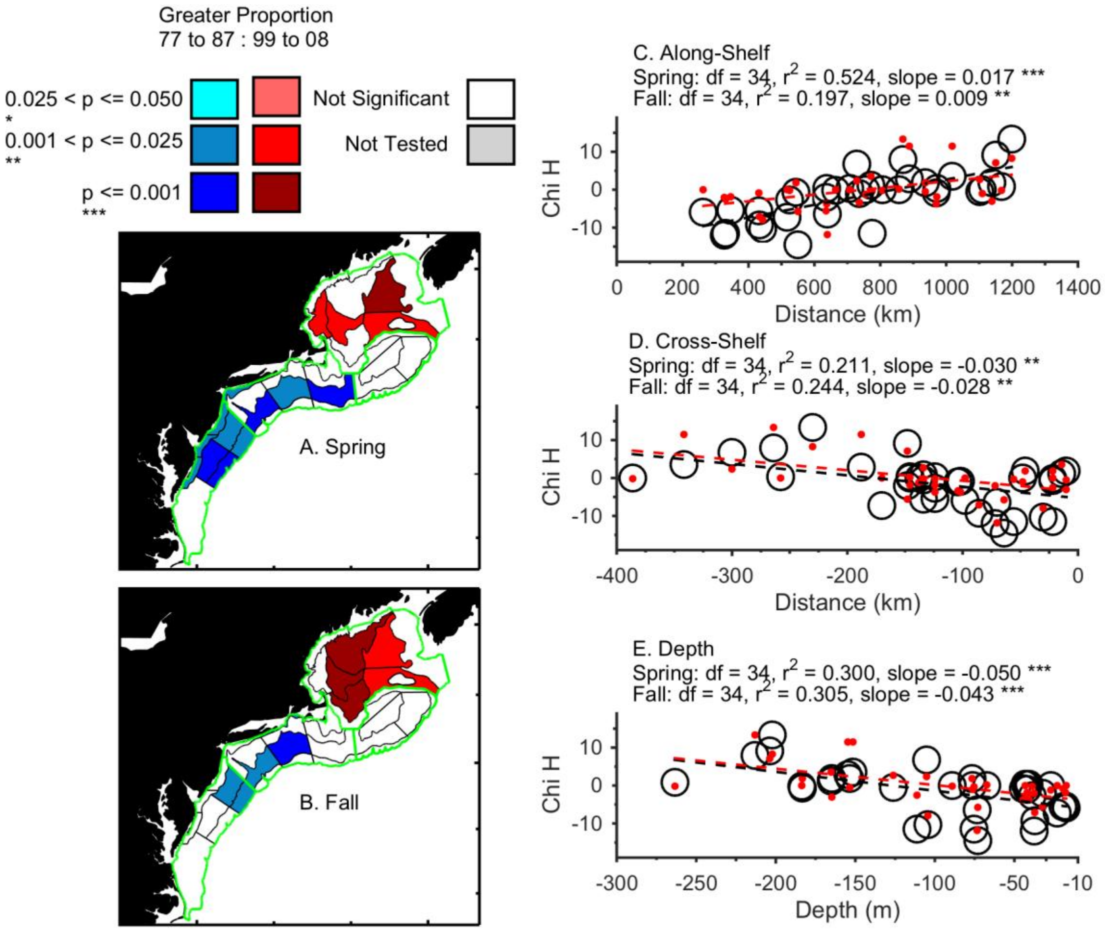

Return to S1 File Table A.

S1 Fig Y. Spatial distribution of adult *Urophycis tenuis* (White Hake) on the Northeast U.S. Shelf Ecosystem. Change in distribution of adult *Urophycis tenuis* in the spring (A; ○) and fall (B; •) were examined in the along-shelf (C), cross-shelf (D), and depth (E) directions. *Urophycis tenuis* shifted significantly northward (C) in the spring and deeper (E) in the spring and fall.

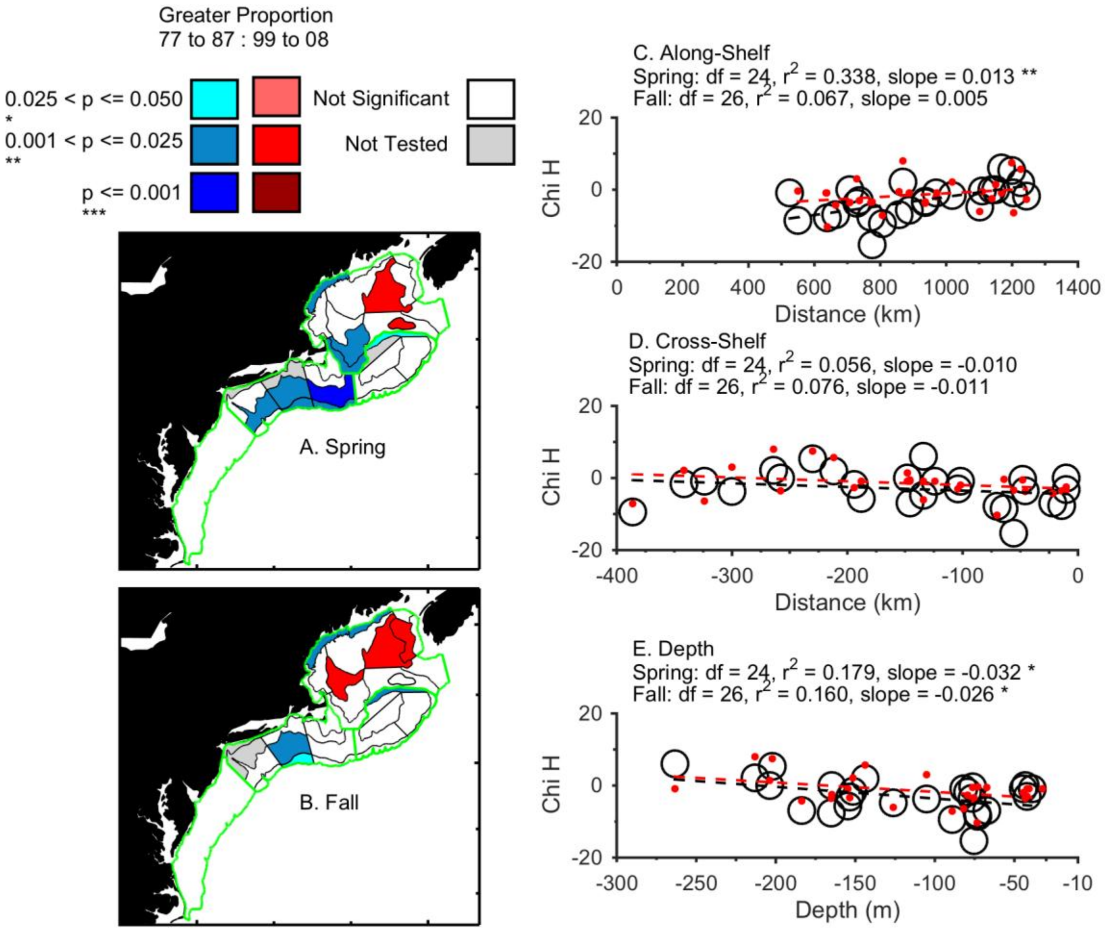

[Return to S1 File Table A](#).

S1 Fig Z. Spatial and seasonal distribution of larval *Urophycis* spp. (Urophycis Hake) on the Northeast U.S. Shelf Ecosystem. Change in spatial distribution of larval *Urophycis* spp. in July - August (A; •), September - October (B; ○), and November – December (C; •) were examined in the along-shelf (D), cross-shelf (E), and depth (F) directions. Larval *Urophycis* spp. did not shift significantly spatially or seasonally (G).

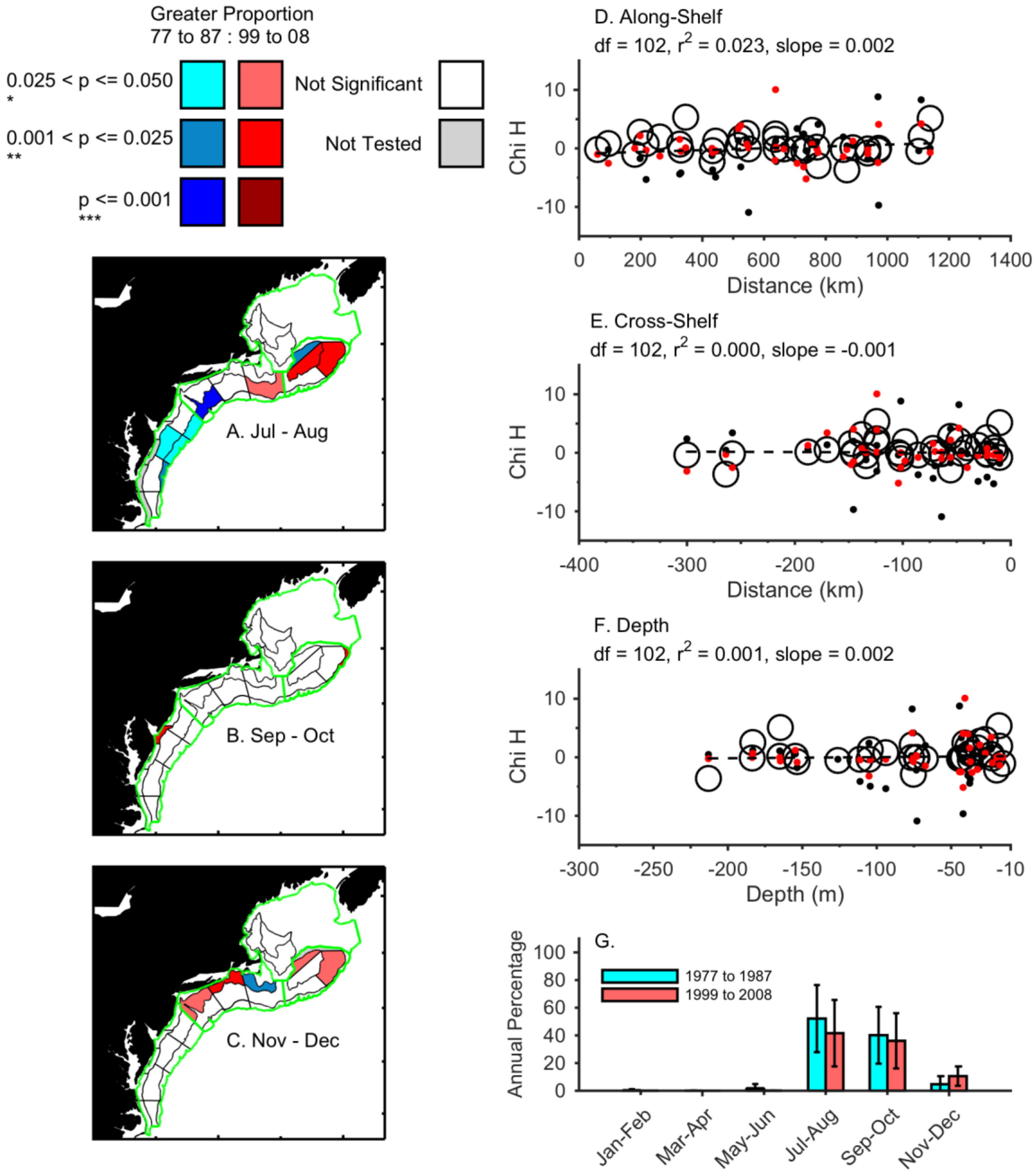

Return to S1 File Table A.

S1 Fig AA. Spatial and seasonal distribution of larval *Merluccius albidus* (Offshore Hake) on the Northeast U.S. Shelf Ecosystem. Change in spatial distribution of larval *Merluccius albidus* in May – June (A; •), July - August (B; ○), and September - October (C; •) were examined in the along-shelf (D), cross-shelf (E), and depth (F) directions. Larval *Merluccius albidus* did not shift significantly spatially. Seasonal distribution shifted significantly later (G).

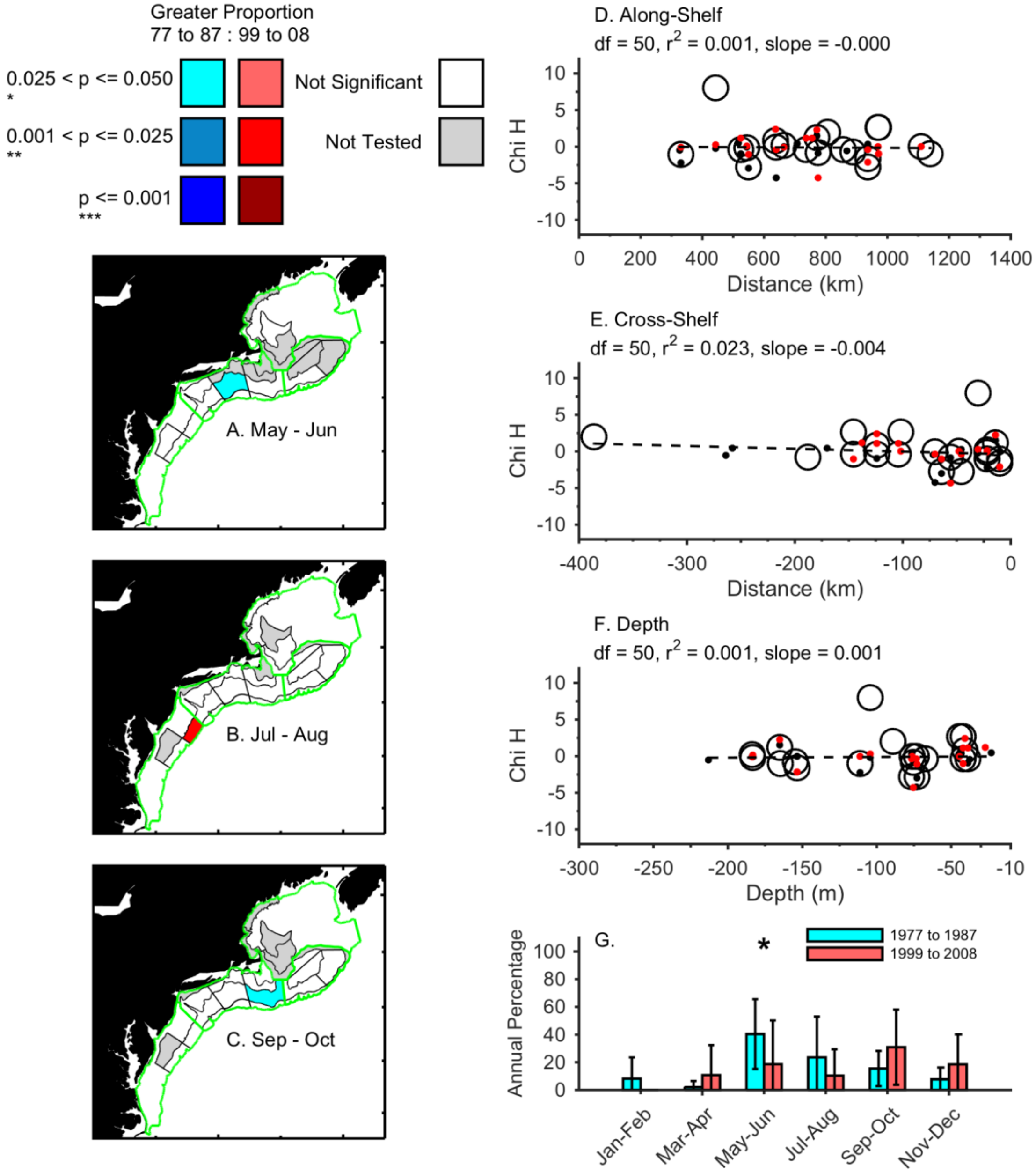

Return to S1 File Table A.

S1 Fig AB. Spatial distribution of adult *Merluccius albidus* (Offshore Hake) on the Northeast U.S. Shelf Ecosystem. Change in distribution of adult *Merluccius albidus* in the spring (A; ○) and fall (B; •) were examined in the along-shelf (C), cross-shelf (D), and depth (E) directions. *Merluccius albidus* shifted significantly southward (C) in the spring and shallower (E) in the fall.

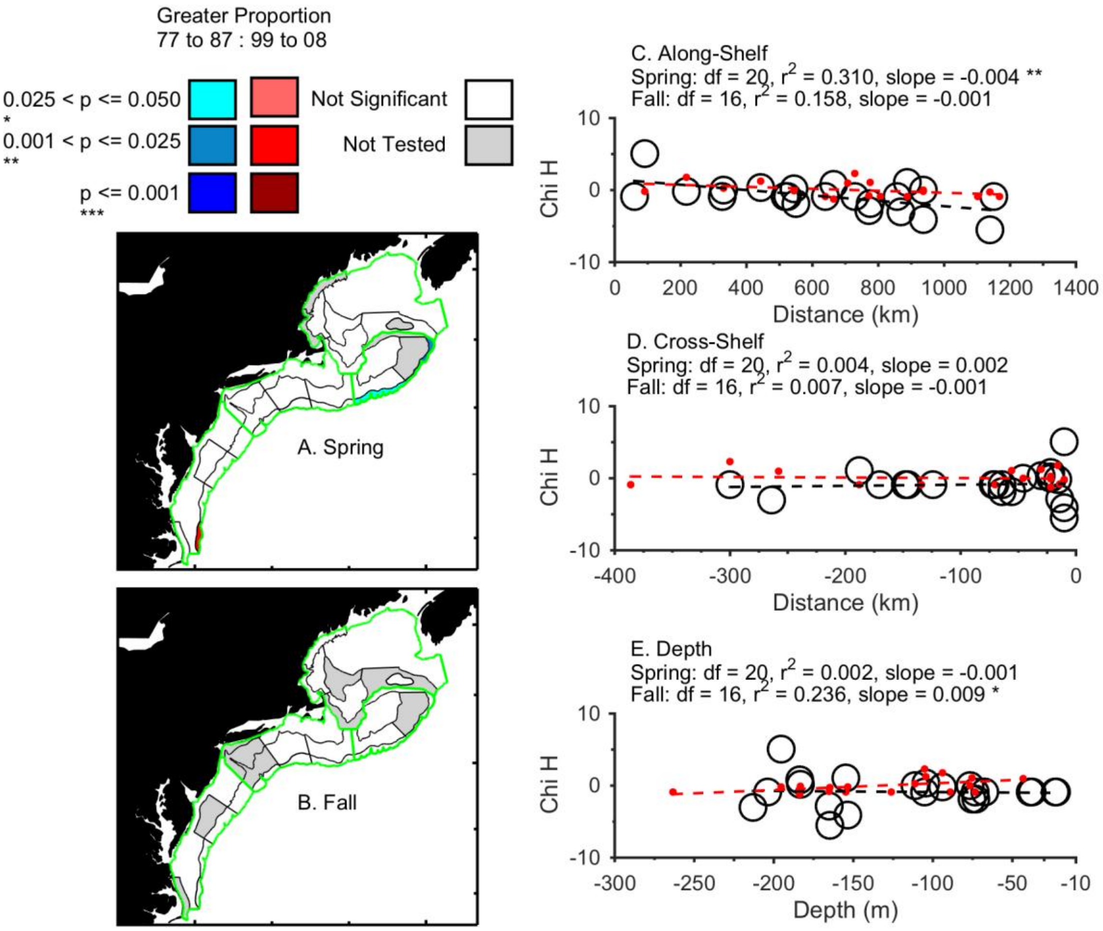

Return to S1 File Table A.

S1 Fig AC. Spatial and seasonal distribution of larval *Merluccius bilinearis* (Silver Hake) on the Northeast U.S. Shelf Ecosystem. Change in spatial distribution of larval *Merluccius bilinearis* in May – June (A; •), July - August (B; ○), and September - October (C; •) were examined in the along-shelf (D), cross-shelf (E), and depth (F) directions. Larval *Merluccius bilinearis* shifted significantly northward (D). Seasonal distribution did not shift significantly (G).

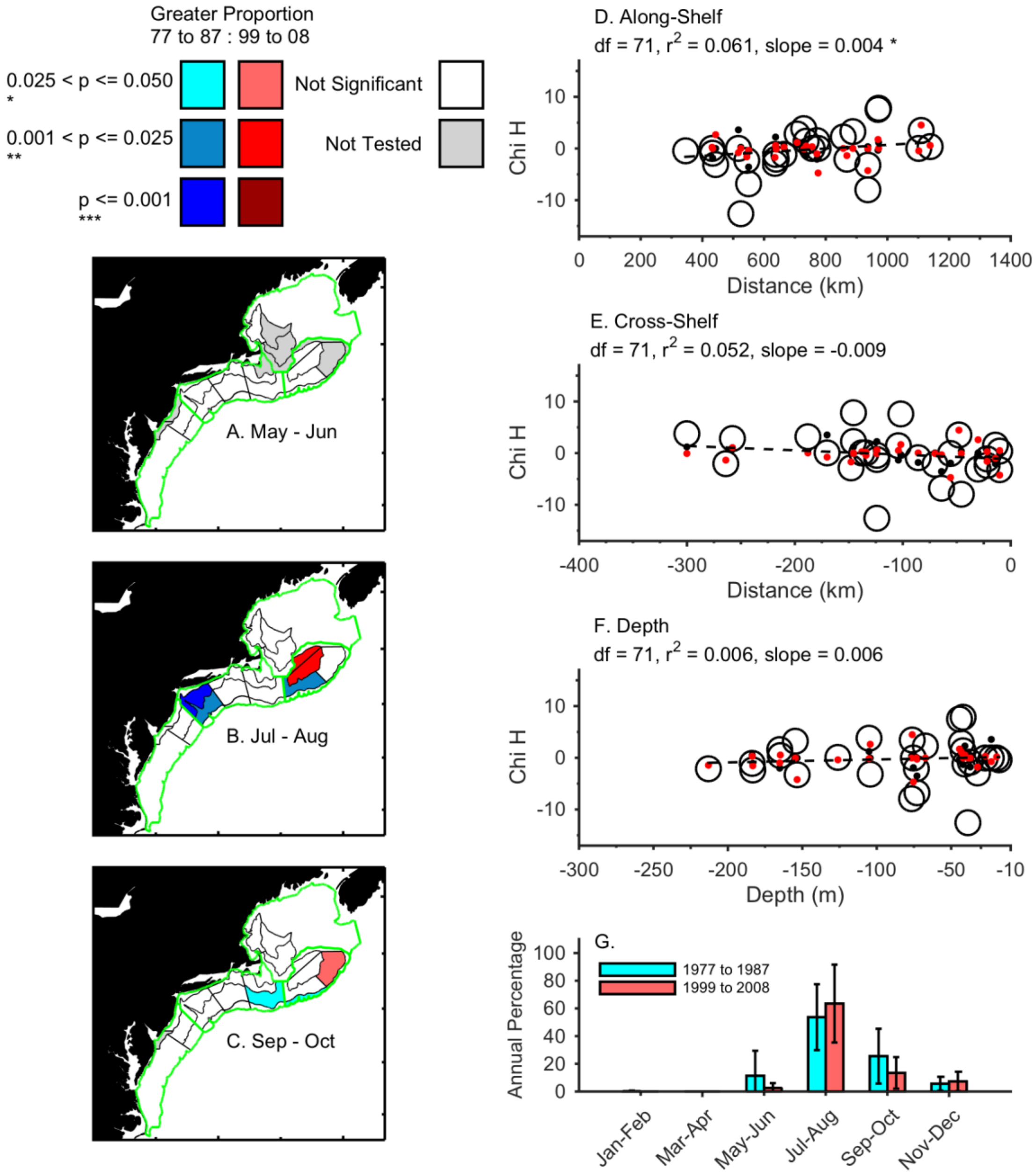

Return to S1 File Table A.

S1 Fig AD. Spatial distribution of adult *Merluccius bilinearis* (Silver Hake) on the Northeast U.S. Shelf Ecosystem. Change in distribution of adult *Merluccius bilinearis* in the spring (A; ○) and fall (B; •) were examined in the along-shelf (C), cross-shelf (D), and depth (E) directions. *Merluccius bilinearis* shifted significantly northward (C), inshore (D) and deeper (E) in the spring, and northward (C) and inshore (D) in the fall.

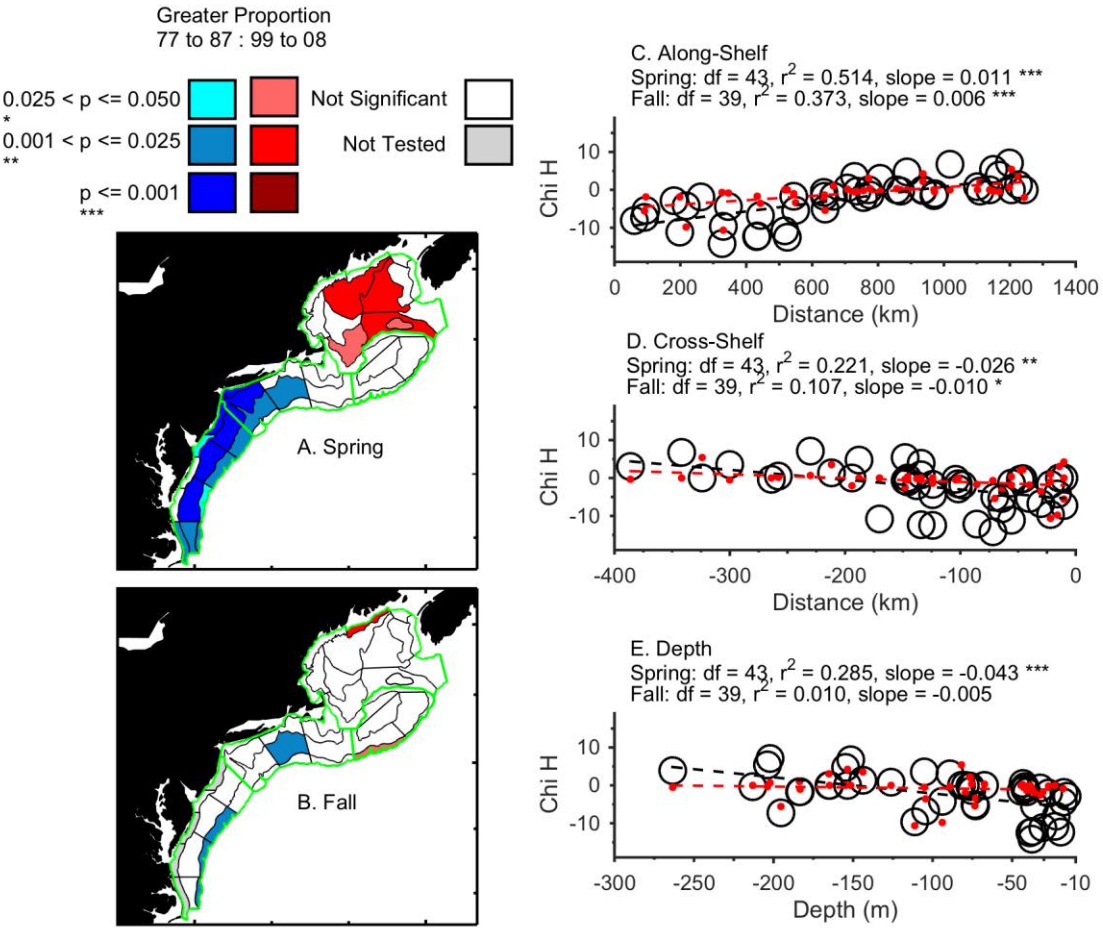

Return to S1 File Table A.

S1 Fig AE. Spatial and seasonal distribution of larval *Lophius americanus* (Monkfish) on the Northeast U.S. Shelf Ecosystem. Change in spatial distribution of larval *Lophius americanus* in May – June (A; •), July - August (B; ○), and September - October (C; •) were examined in the along-shelf (D), cross-shelf (E), and depth (F) directions. Larval *Lophius americanus* shifted significantly northward (D). Seasonal distribution shifted significantly later (G).

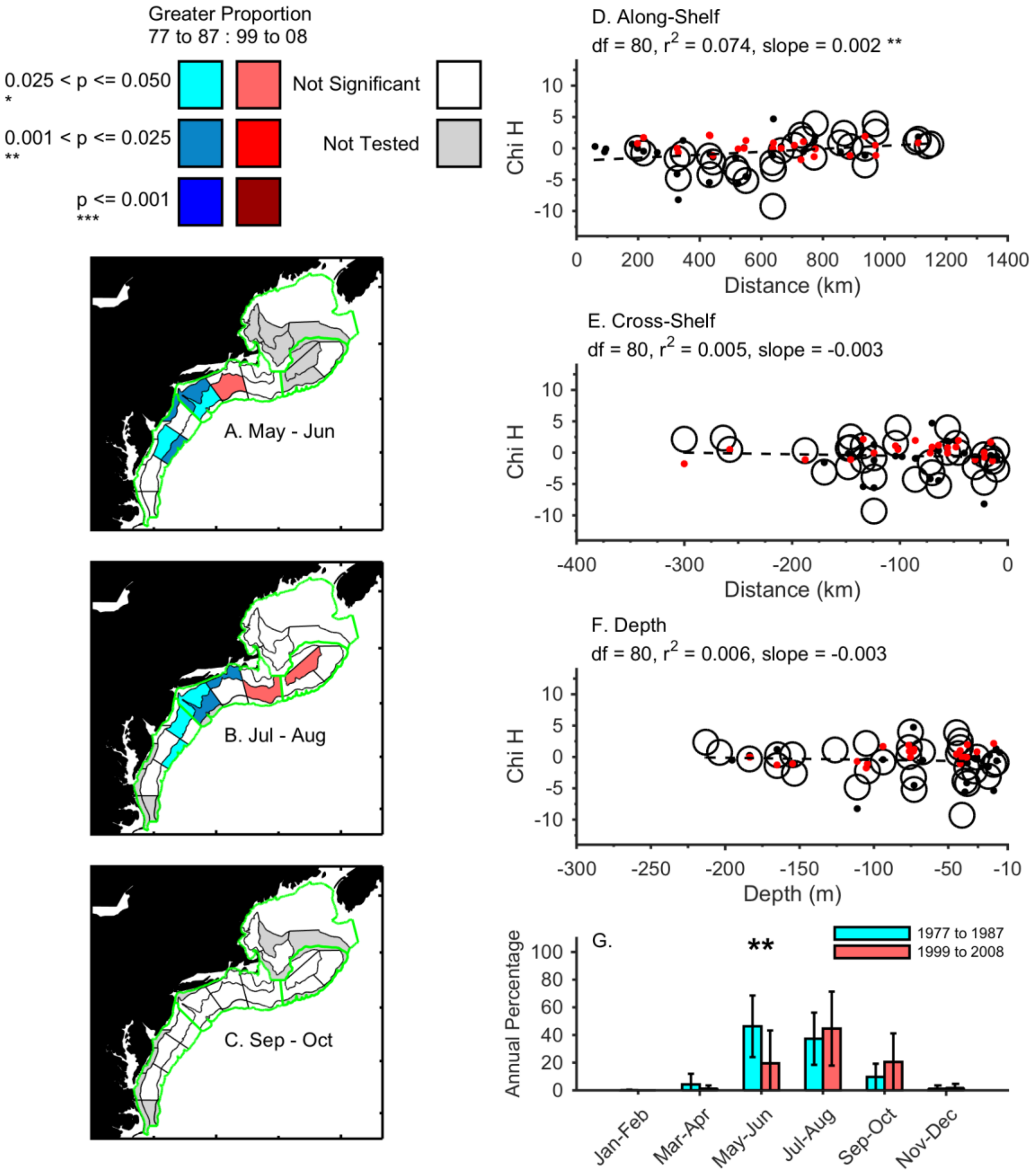

Return to S1 File Table A.

S1 Fig AF. Spatial distribution of adult *Lophius americanus* (Monkfish) on the Northeast U.S. Shelf Ecosystem. Change in distribution of adult *Lophius americanus* in the spring (A; ○) and fall (B; •) were examined in the along-shelf (C), cross-shelf (D), and depth (E) directions. *Lophius americanus* shifted significantly northward (C) and deeper (E) in the spring.

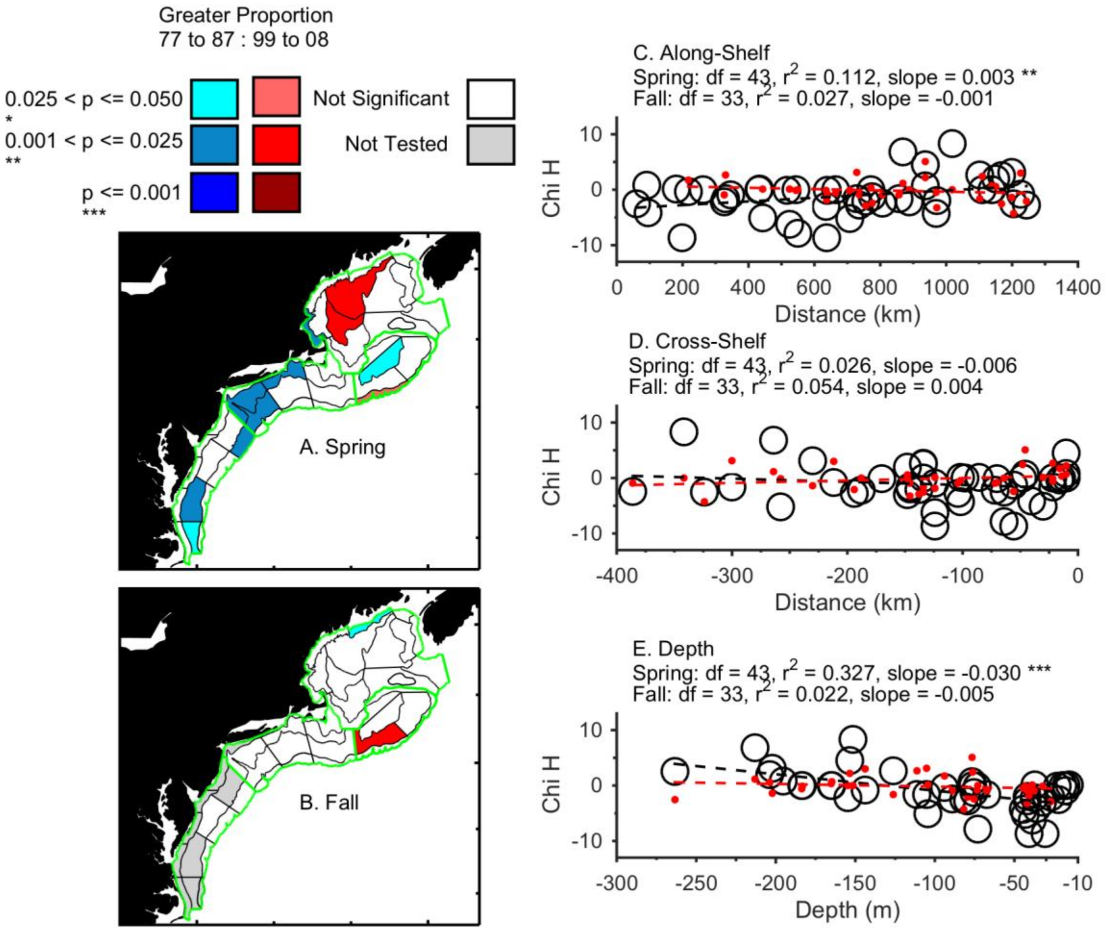

Return to S1 File Table A.

S1 Fig AG. Spatial and seasonal distribution of larval *Sebastes* spp. (Redfishes) on the Northeast U.S. Shelf Ecosystem. Change in spatial distribution of larval *Sebastes* spp. in March – April (A; •), May – June (B; ○), and July - August (C; •) were examined in the along-shelf (D), cross-shelf (E), and depth (F) directions. Larval *Sebastes* spp. did not shift significantly in spatial or seasonal distribution (G).

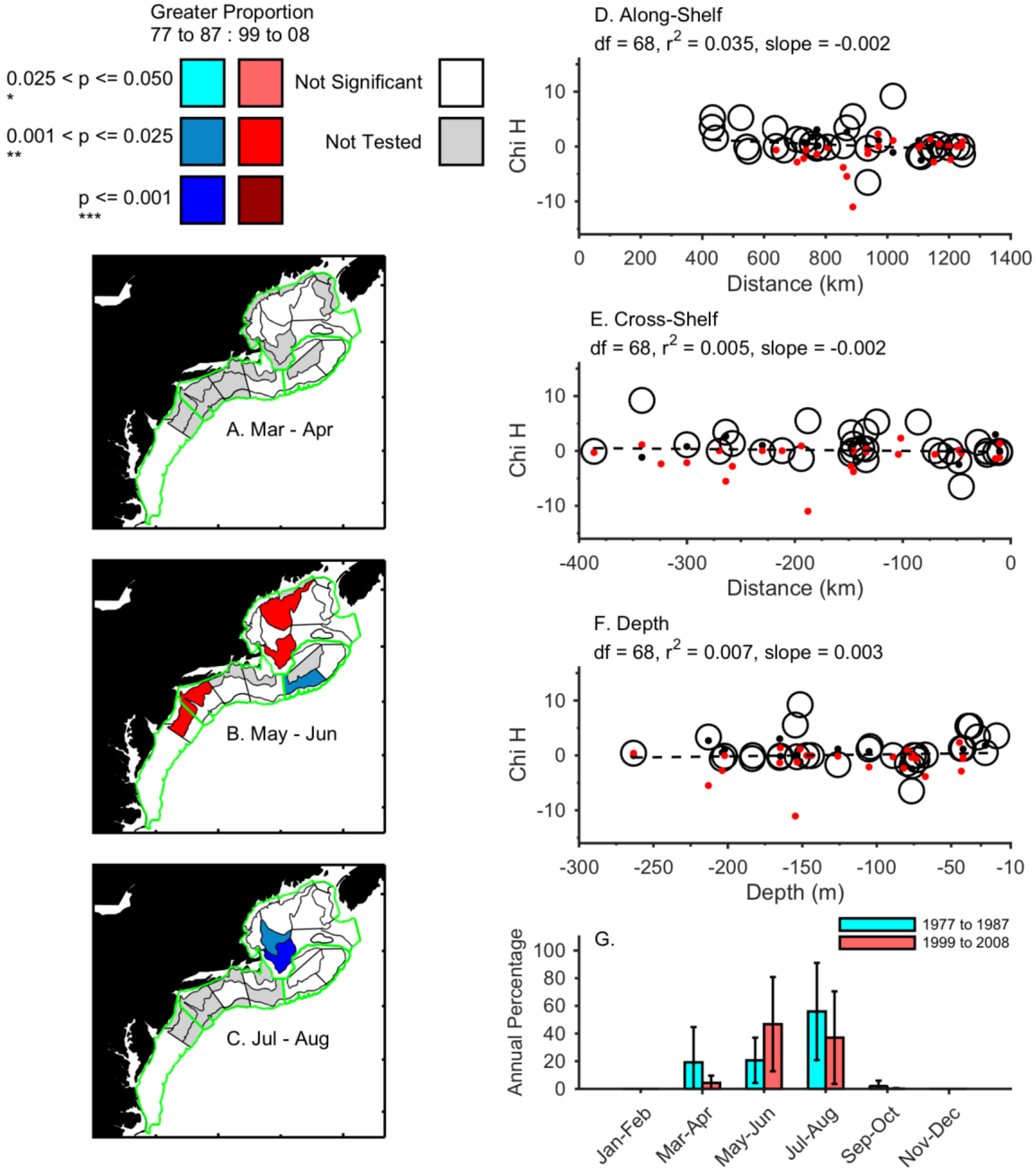

[Return to S1 File Table A](#).

S1 Fig AH. Spatial distribution of adult *Sebastes* spp. (Redfishes) on the Northeast U.S. Shelf Ecosystem. Change in distribution of adult *Sebastes* spp. in the spring (A; ○) and fall (B; •) were examined in the along-shelf (C), cross-shelf (D), and depth (E) directions. *Sebastes* spp. shifted significantly offshore (D) in the spring.

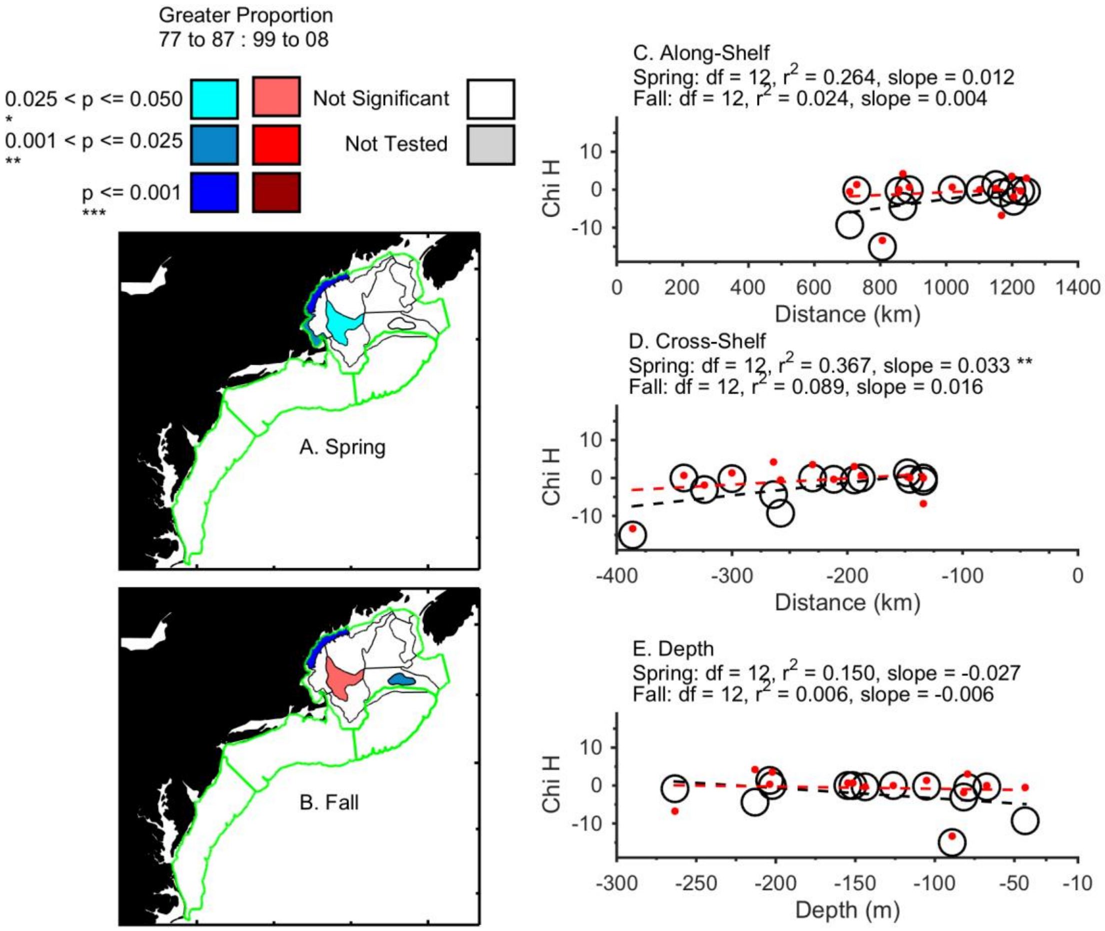

Return to S1 File Table A.

S1 Fig AI. Spatial and seasonal distribution of larval *Prionotus* spp. (Searobins) on the Northeast U.S. Shelf Ecosystem. Change in spatial distribution of larval *Prionotus* spp. in July - August (A; •), September – October (B; ○), and November – December (C; •) were examined in the along-shelf (D), cross-shelf (E), and depth (F) directions. Larval *Prionotus* spp. did not shift significantly in spatial distribution. Seasonal distribution shifted significantly later (G).

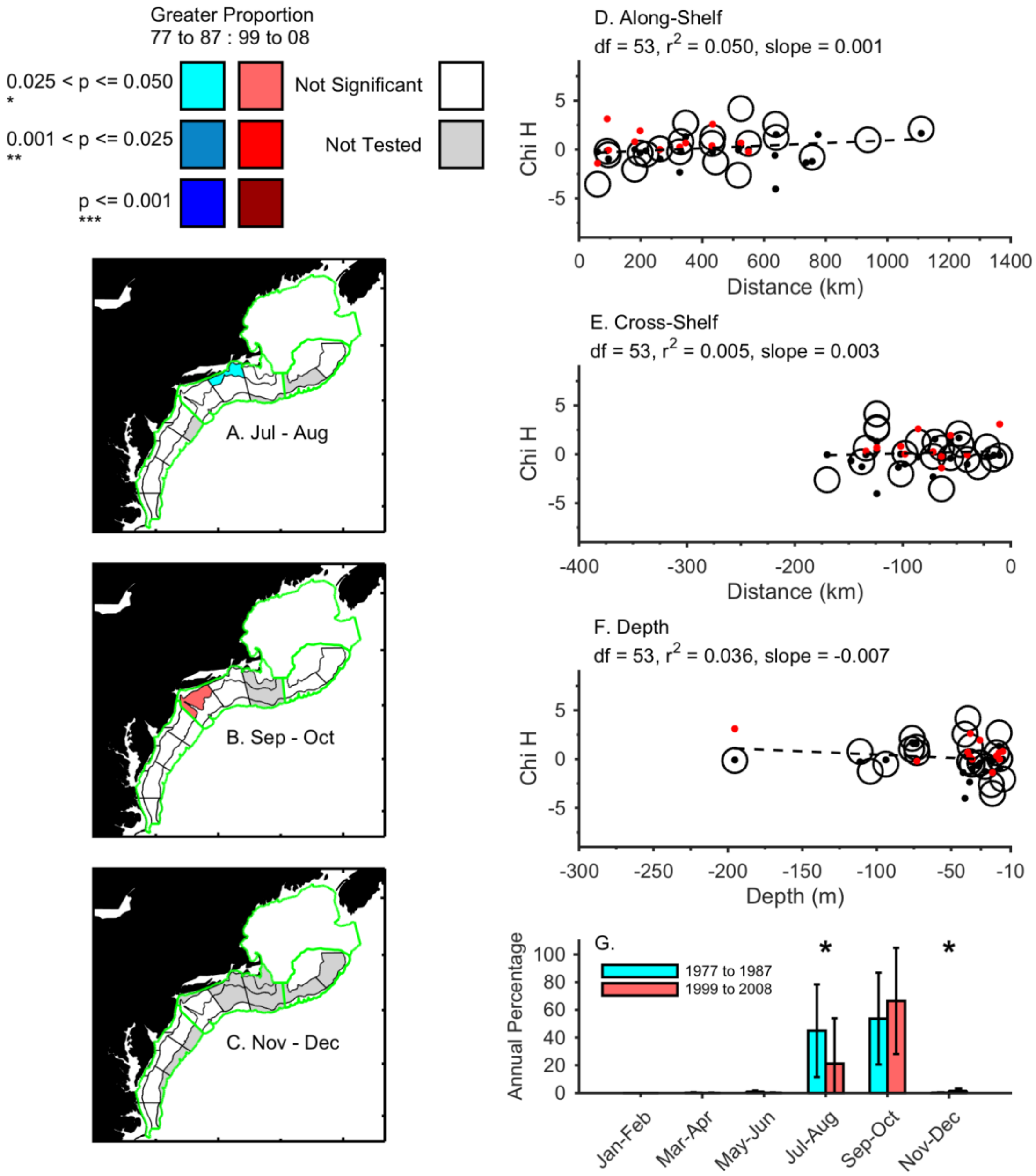

Return to S1 File Table A.

S1 Fig AJ. Spatial and seasonal distribution of larval *Myoxocephalus aenaeus* (Grubby) on the Northeast U.S. Shelf Ecosystem. Change in spatial distribution of larval *Myoxocephalus aenaeus* in March – April (A; •), May – June (B; ○), and July – August (C; •) were examined in the along-shelf (D), cross-shelf (E), and depth (F) directions. Larval *Myoxocephalus aenaeus* shifted significantly northward (D). Seasonal distribution did not shift significantly (G).

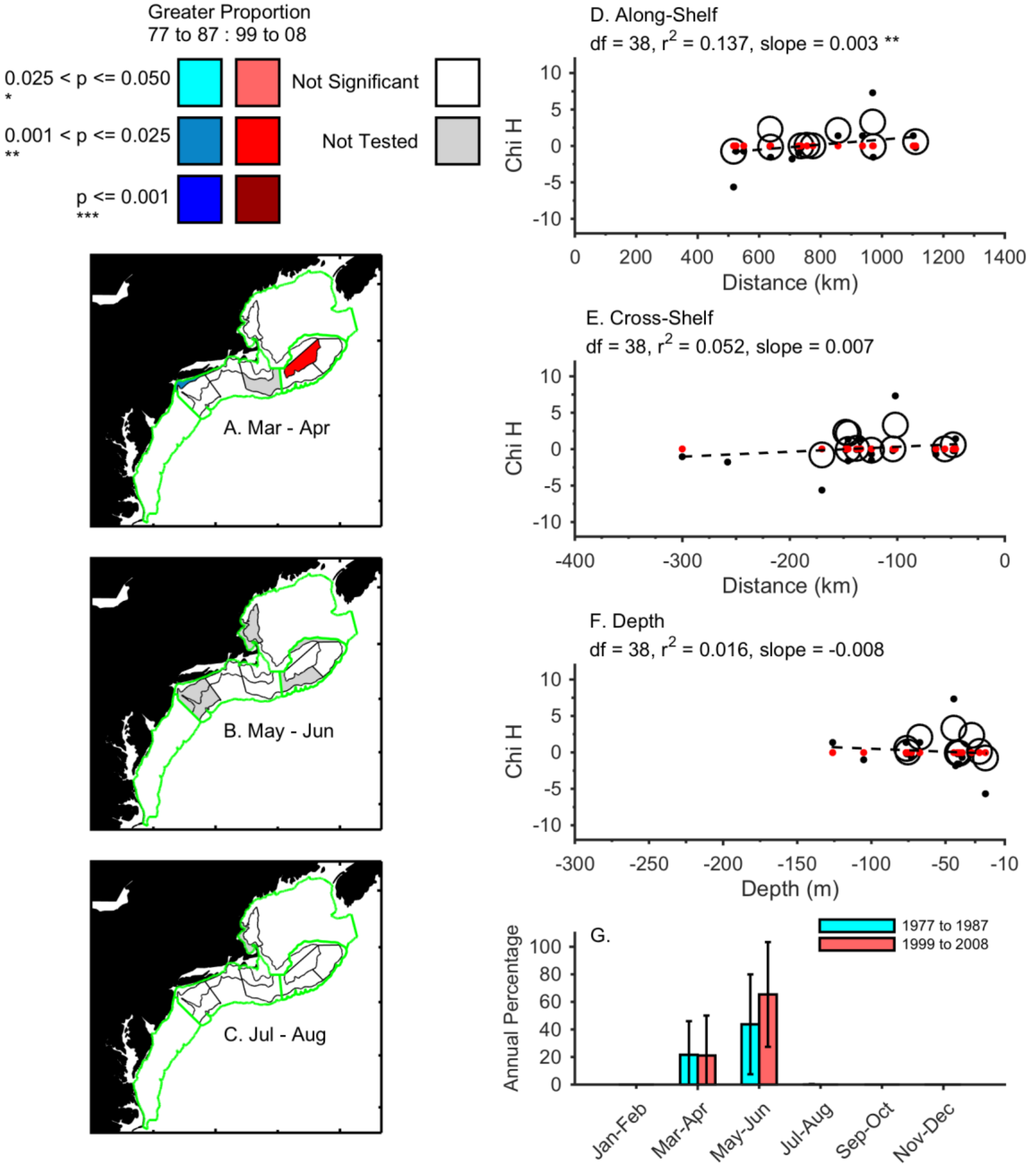

Return to S1 File Table A.

S1 Fig AK. Spatial and seasonal distribution of larval *Myoxocephalus octodecemspinosus* (Longhorn Sculpin) on the Northeast U.S. Shelf Ecosystem. Change in spatial distribution of larval *Myoxocephalus octodecemspinosus* in January – February (A; •), March – April (B; ○), and May – June (C; •) were examined in the along-shelf (D), cross-shelf (E), and depth (F) directions. Larval *Myoxocephalus octodecemspinosus* did not shift significantly in spatial or seasonal distribution (G).

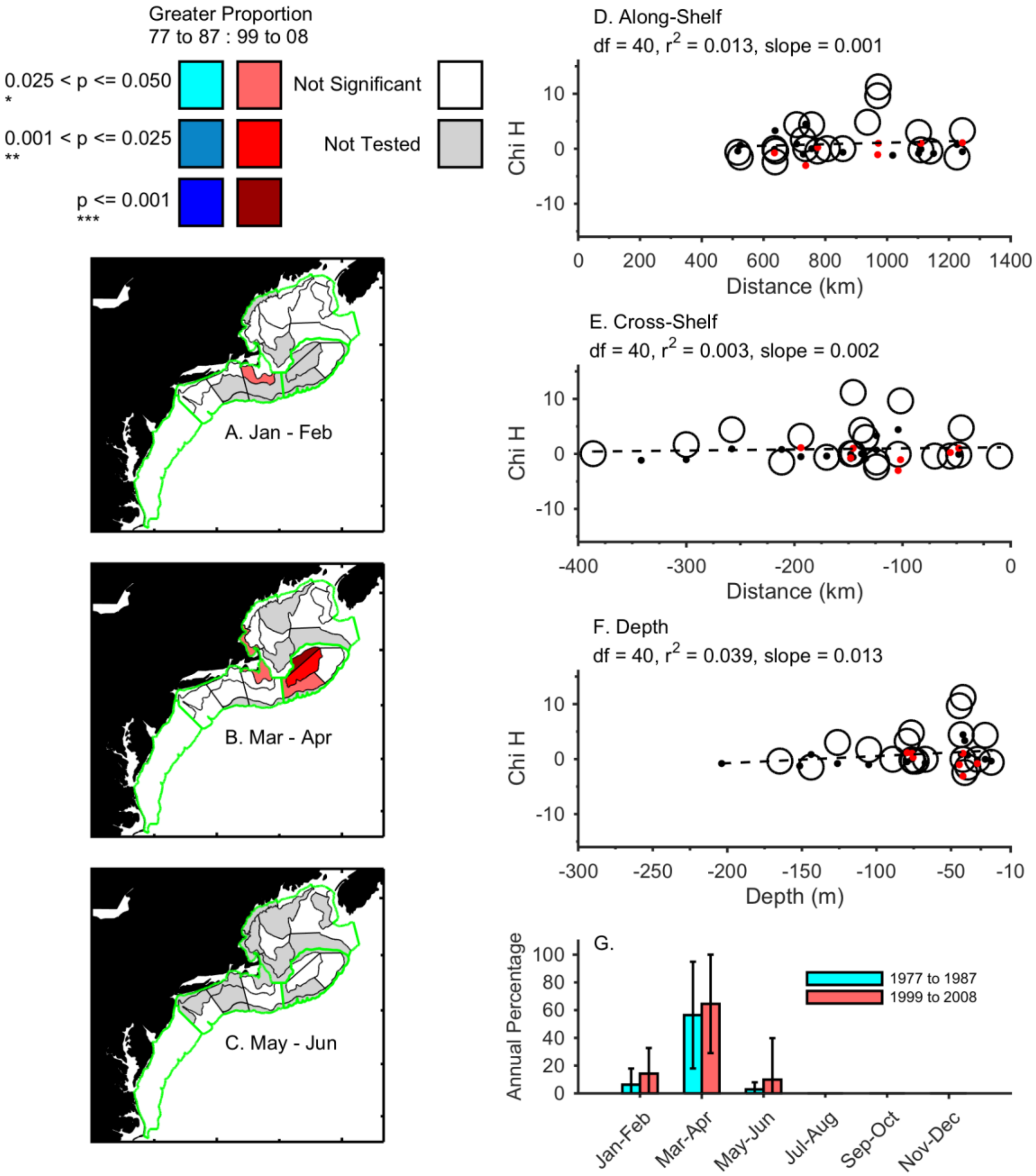

Return to S1 File Table A.

S1 Fig AL. Spatial distribution of adult *Myoxocephalus octodecemspinosus* (Longhorn Sculpin) on the Northeast U.S. Shelf Ecosystem. Change in distribution of adult *Myoxocephalus octodecemspinosus* in the spring (A; ○) and fall (B; •) were examined in the along-shelf (C), cross-shelf (D), and depth (E) directions. *Myoxocephalus octodecemspinosus* shifted significantly northward (C) in the spring.

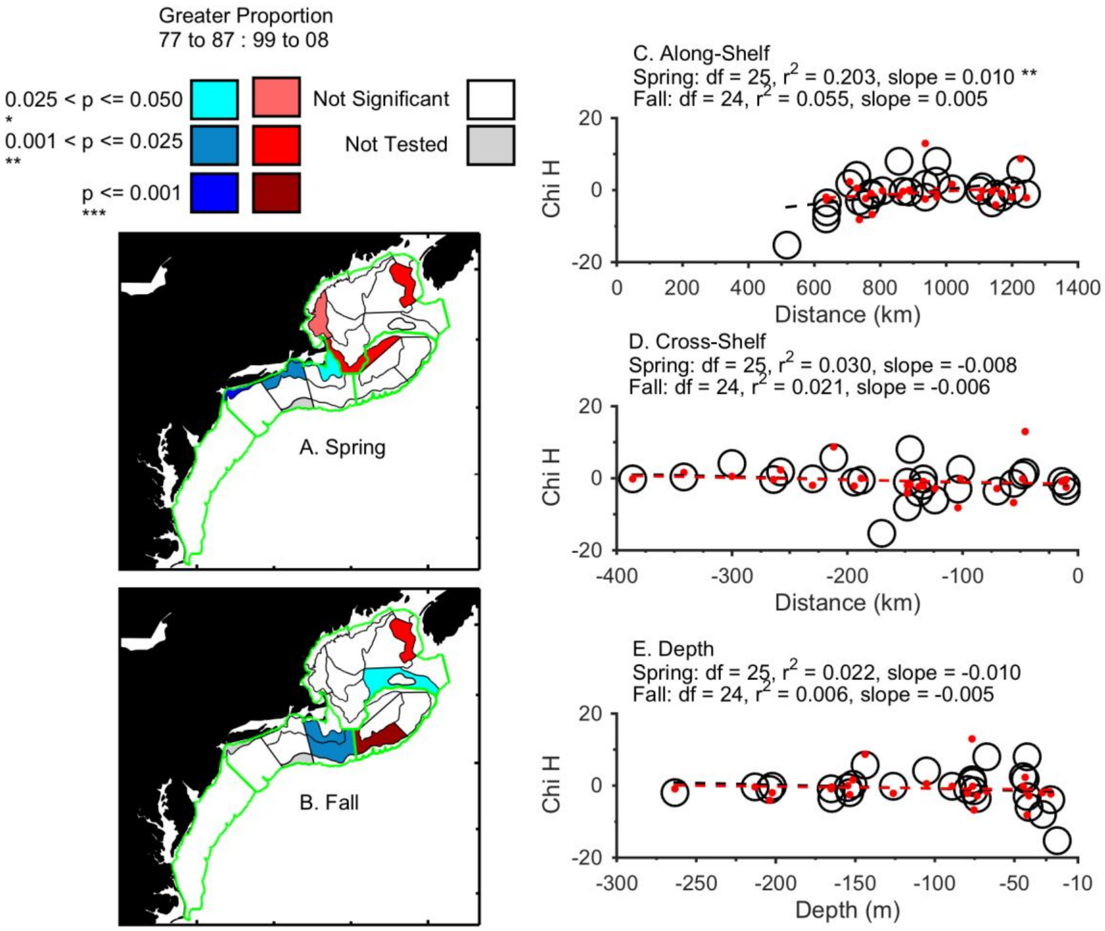

Return to S1 File Table A.

S1 Fig AM. Spatial and seasonal distribution of larval *Centropristis striata* (Black Sea Bass) on the Northeast U.S. Shelf Ecosystem. Change in spatial distribution of larval *Centropristis striata* in May – June (A; •), July – August (B; ○), and September – October (C; •) were examined in the along-shelf (D), cross-shelf (E), and depth (F) directions. Larval *Centropristis striata* shifted significantly northward and inshore (D) spatially and later (G) seasonally.

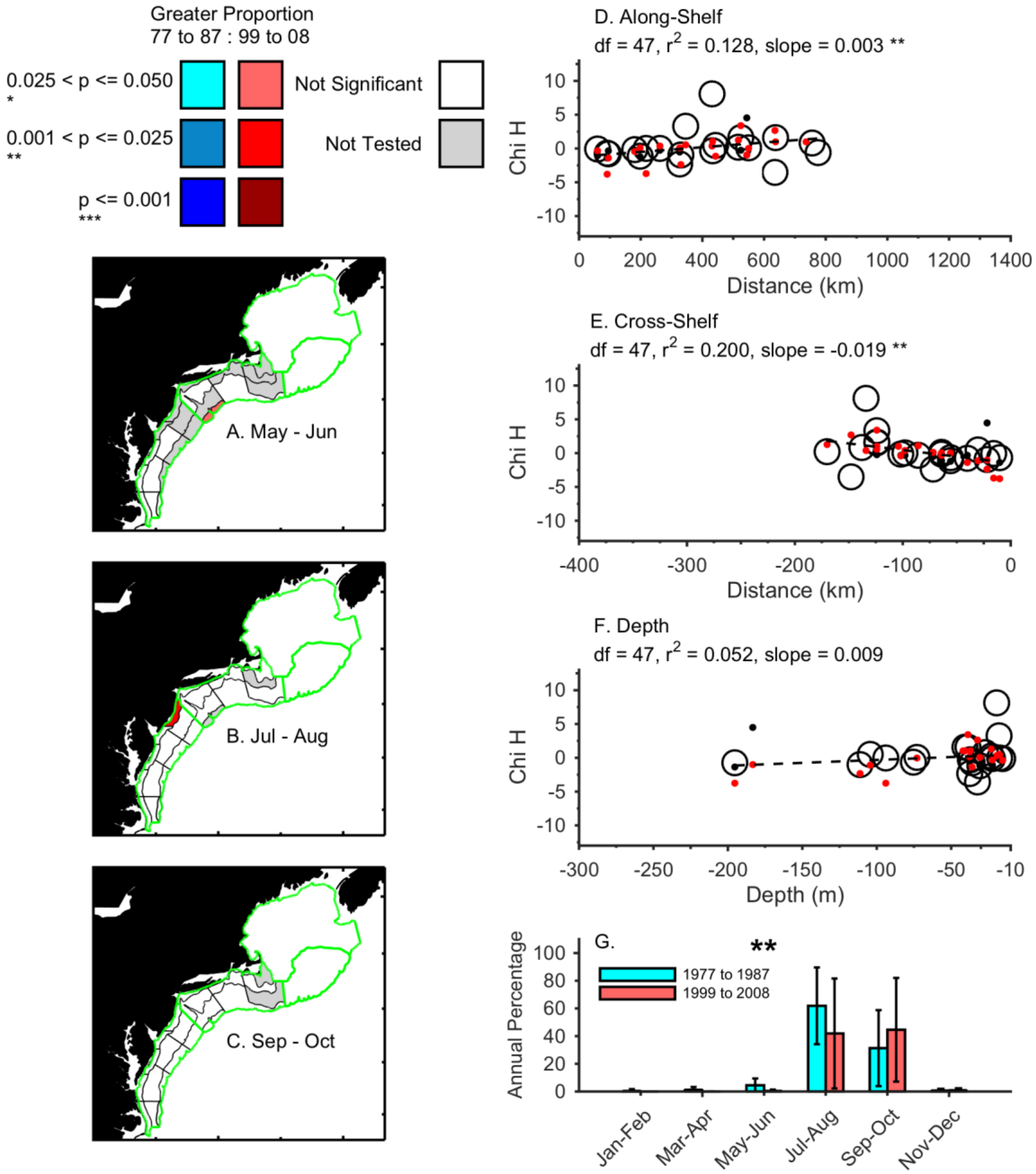

Return to S1 File Table A.

S1 Fig AN. Spatial distribution of adult *Centropristis striata* (Black Sea Bass) on the Northeast U.S. Shelf Ecosystem. Change in distribution of adult *Centropristis striata* in the spring (A; ○) and fall (B; •) were examined in the along-shelf (C), cross-shelf (D), and depth (E) directions. *Centropristis striata* did not shift significantly.

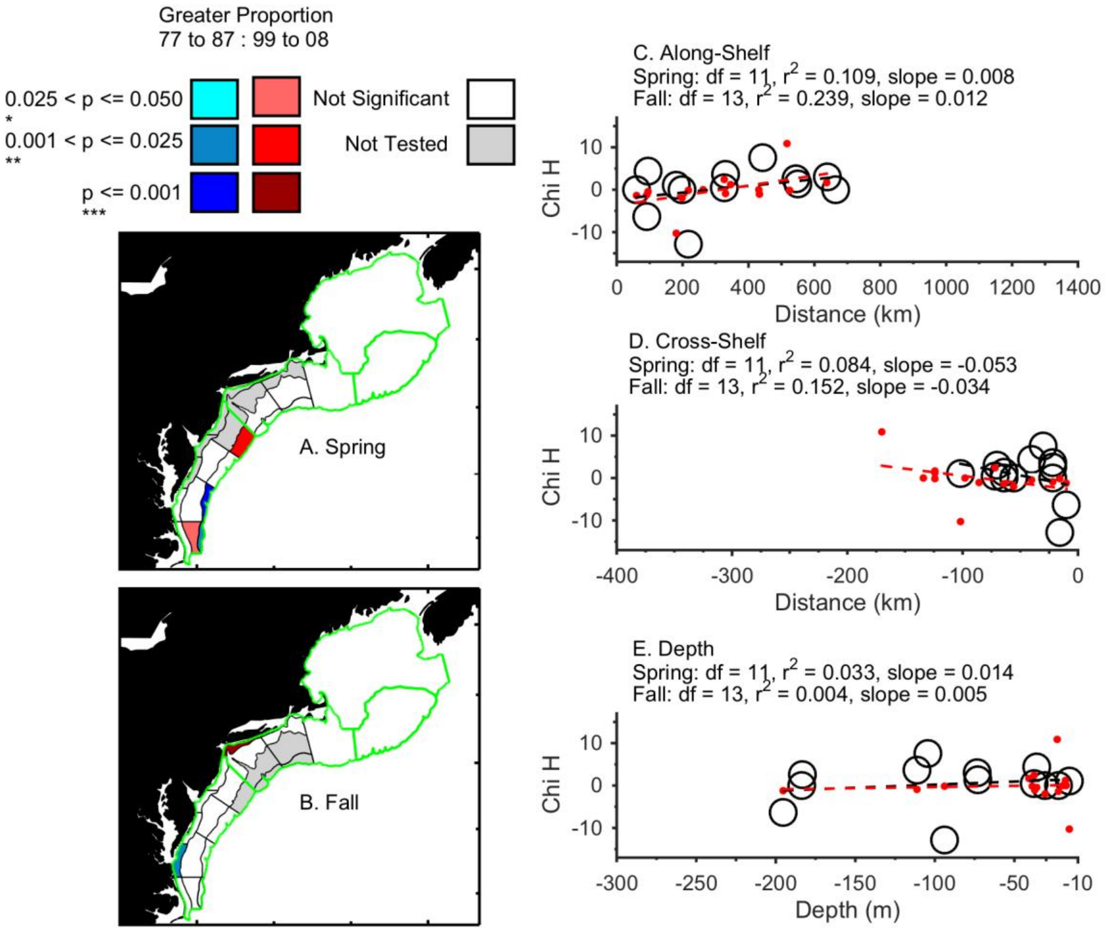

Return to S1 File Table A.

S1 Fig AO. Spatial and seasonal distribution of larval *Pomatomus saltatrix* (Bluefish) on the Northeast U.S. Shelf Ecosystem. Change in spatial distribution of larval *Pomatomus saltatrix* in May – June (A; •), July – August (B; ○), and September – October (C; •) were examined in the along-shelf (D), cross-shelf (E), and depth (F) directions. Larval *Pomatomus saltatrix* did not shift significantly spatially or seasonally (G).

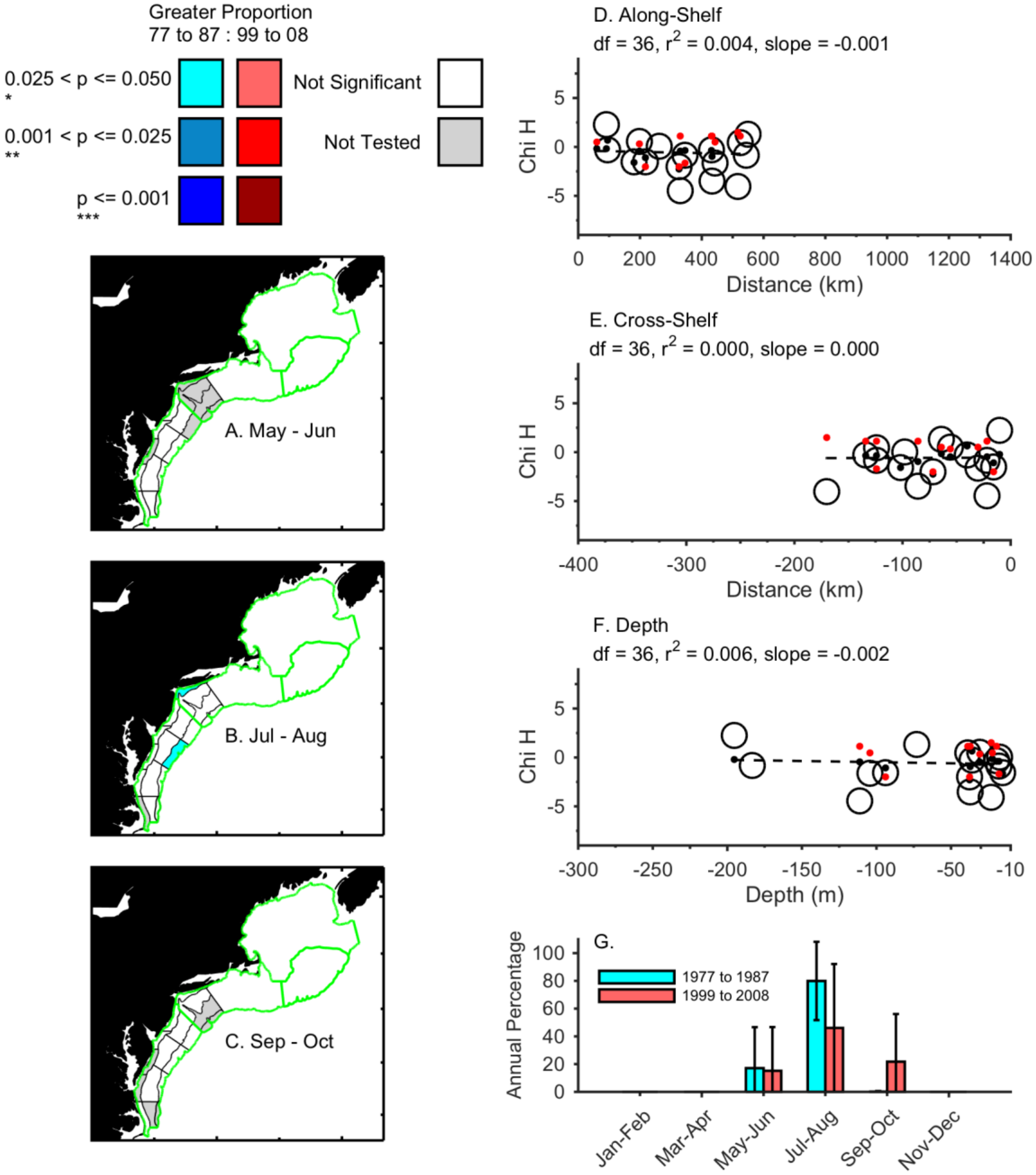

Return to S1 File Table A.

S1 Fig AP. Spatial distribution of adult *Pomatomus saltatrix* (Bluefish) on the Northeast U.S. Shelf Ecosystem. Change in distribution of adult *Pomatomus saltatrix* in the spring (A; ○) and fall (B; •) were examined in the along-shelf (C), cross-shelf (D), and depth (E) directions. *Pomatomus saltatrix* did not shift significantly.

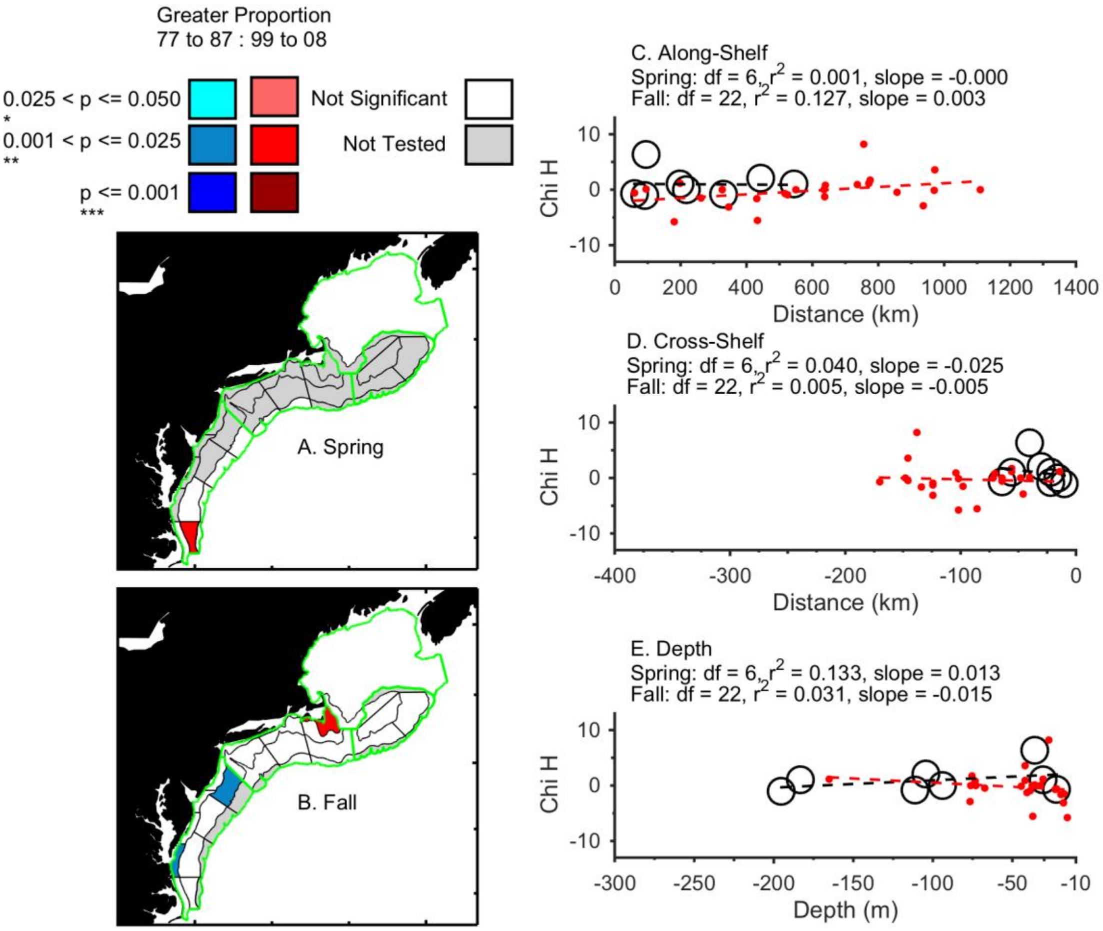

Return to S1 File Table A.

S1 Fig AQ. Spatial distribution of adult *Stenotomus chrysops* (Scup) on the Northeast U.S. Shelf Ecosystem. Change in distribution of adult *Stenotomus chrysops* in the spring (A; ○) and fall (B; •) were examined in the along-shelf (C), cross-shelf (D), and depth (E) directions. *Stenotomus chrysops* did not shift significantly.

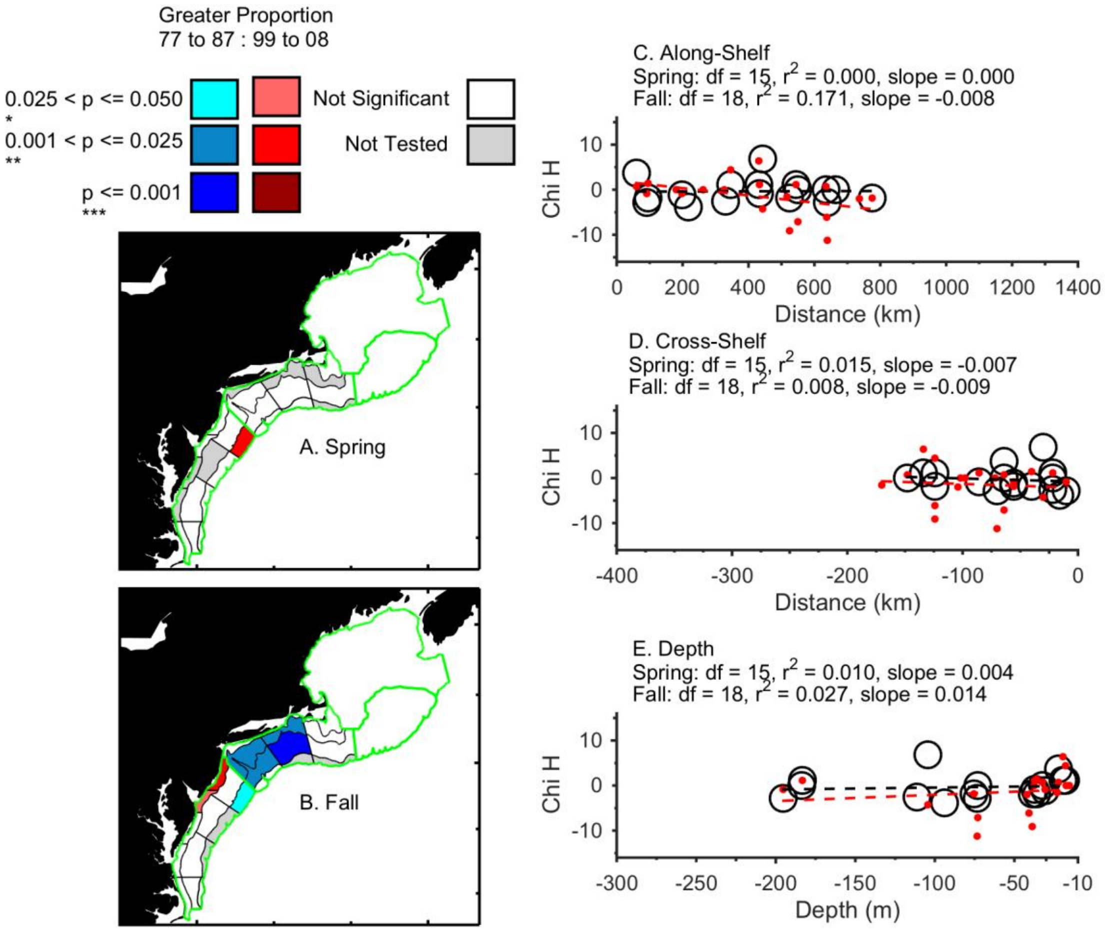

Return to S1 File Table A.

S1 Fig AR. Spatial and seasonal distribution of larval *Cynoscion regalis* (Weakfish) on the Northeast U.S. Shelf Ecosystem. Change in spatial distribution of larval *Cynoscion regalis* in May – June (A; •), July – August (B; ○), and September – October (C; •) were examined in the along-shelf (D), cross-shelf (E), and depth (F) directions. Larval *Cynoscion regalis* did not shift significantly spatially. Seasonal distribution shifted significantly earlier (G).

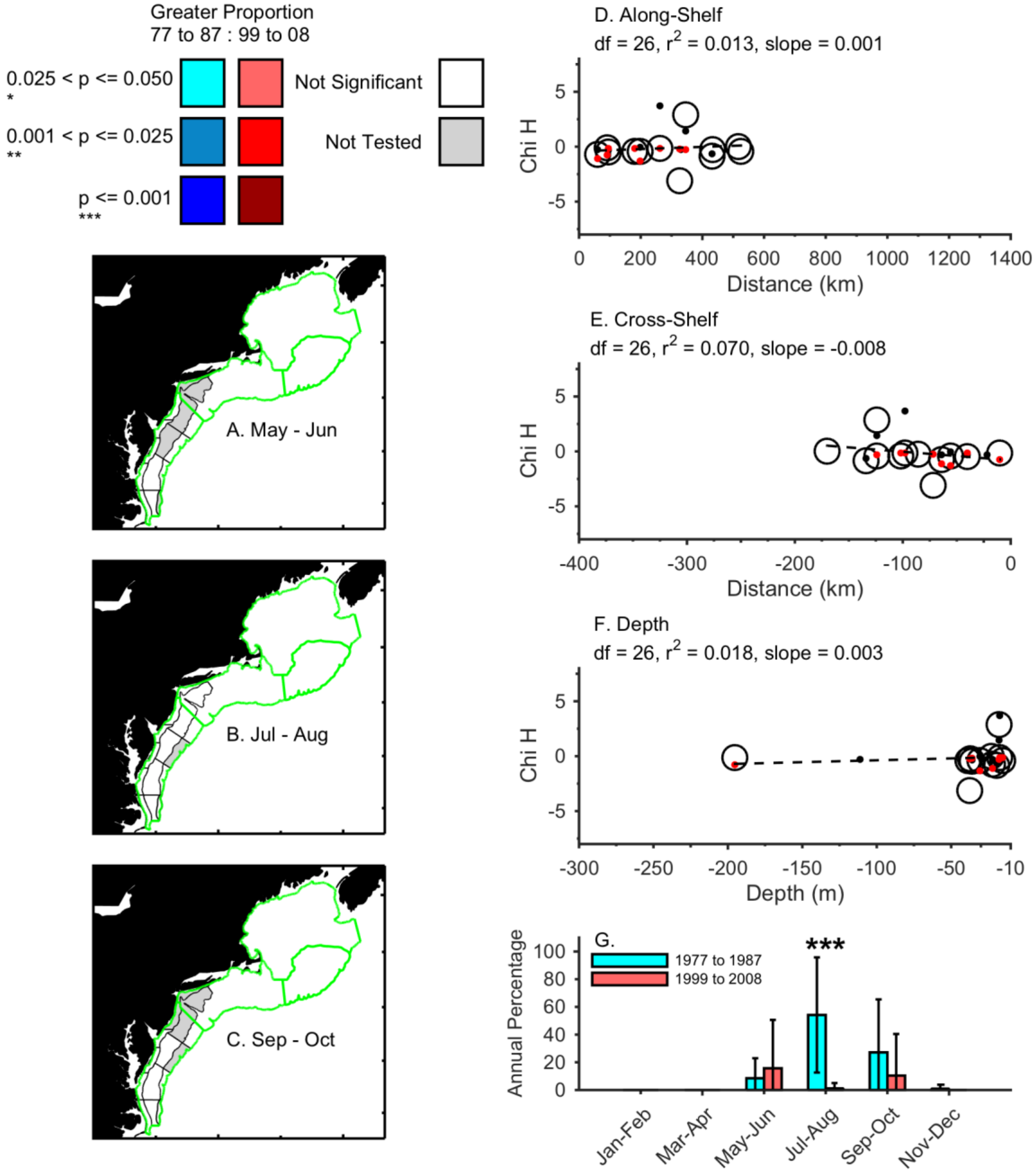

Return to S1 File Table A.

S1 Fig AS. Spatial distribution of adult *Cynoscion regalis* (Weakfish) on the Northeast U.S. Shelf Ecosystem. Change in distribution of adult *Cynoscion regalis* in the spring (A; ○) and fall (B; •) were examined in the along-shelf (C), cross-shelf (D), and depth (E) directions. *Cynoscion regalis* shifted significantly northward (C) in the fall.

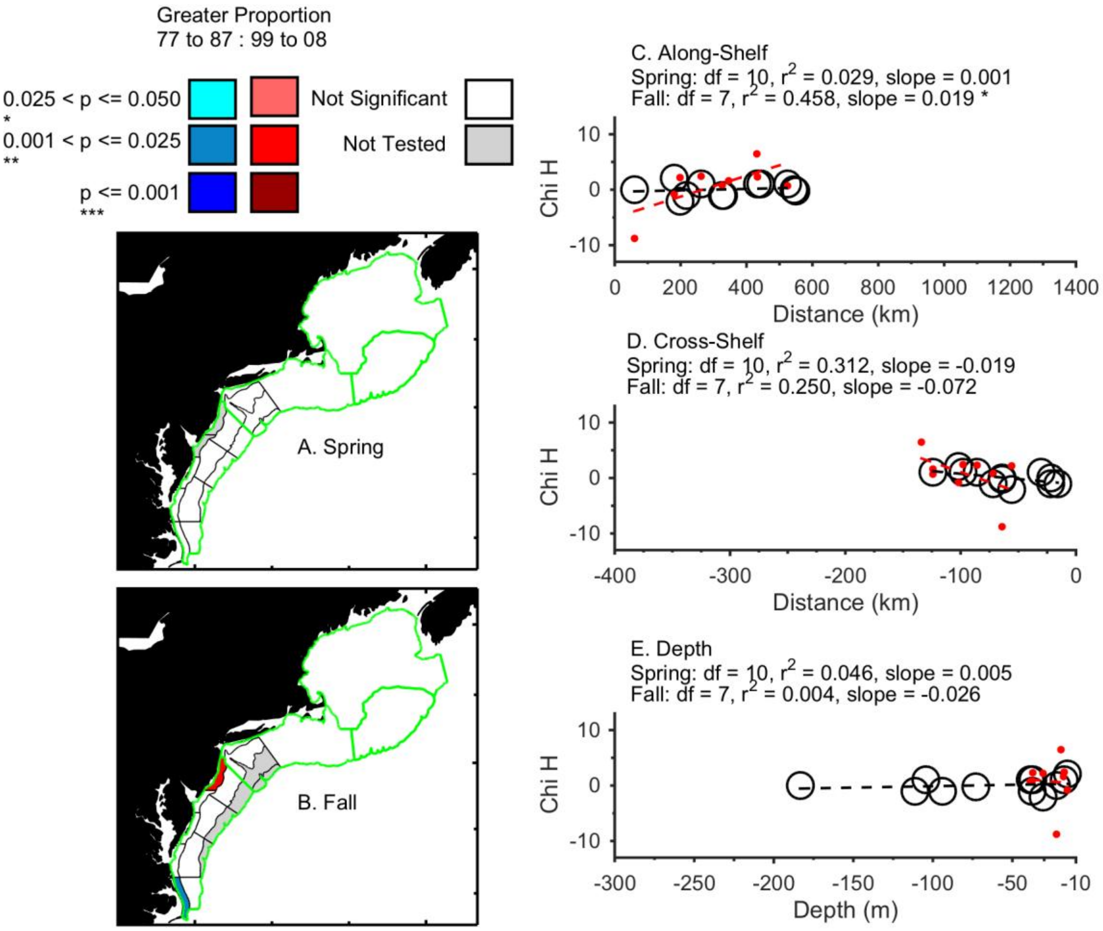

Return to S1 File Table A.

S1 Fig AT. Spatial and seasonal distribution of larval *Leiostomus xanthurus* (Spot) on the Northeast U.S. Shelf Ecosystem. Change in spatial distribution of larval *Leiostomus xanthurus* in January – February (A; •), March – April (B; ○), and May – June (C; •) were examined in the along-shelf (D), cross-shelf (E), and depth (F) directions. Larval *Leiostomus xanthurus* did not shift significantly spatially or seasonally (G).

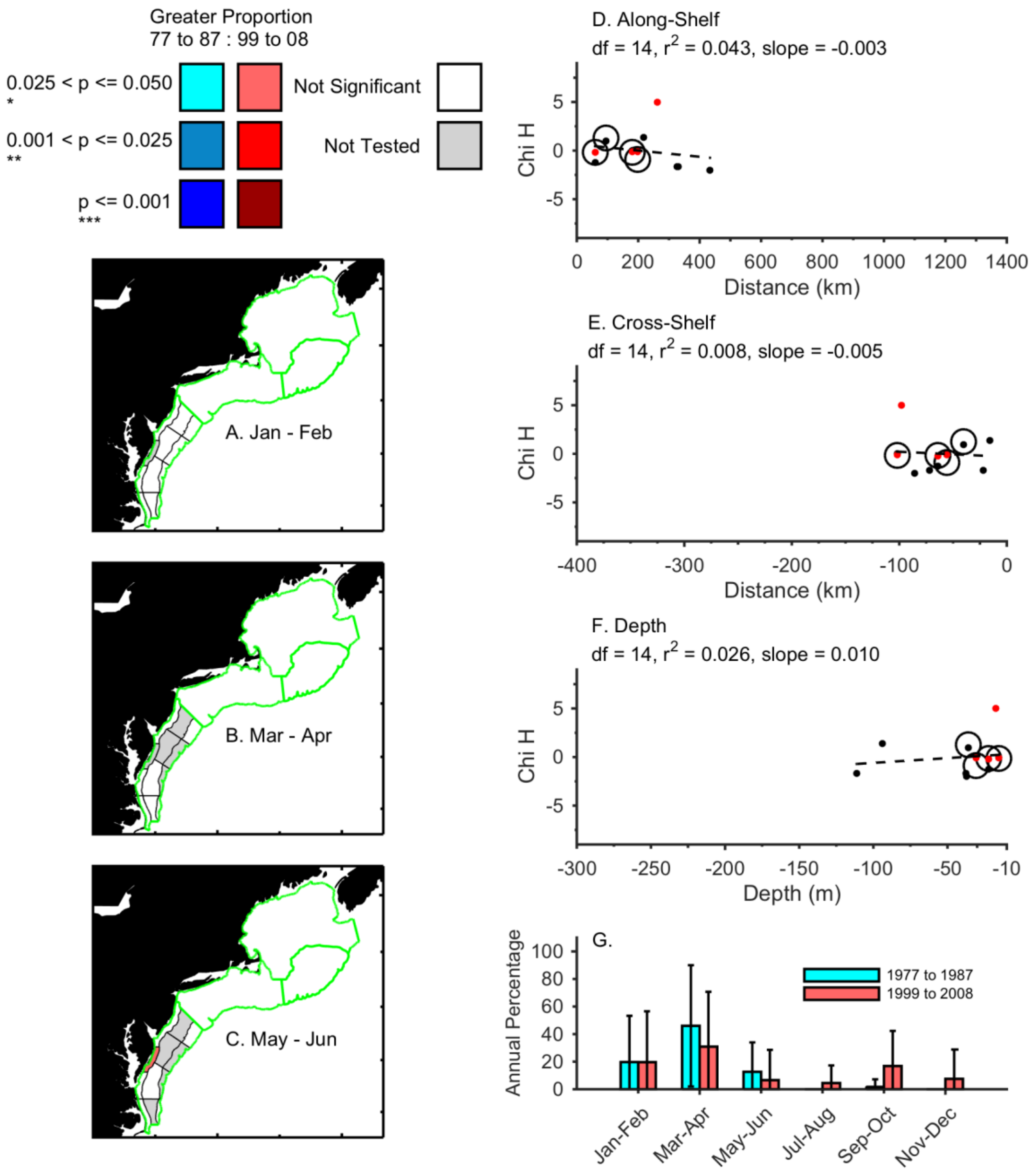

[Return to S1 File Table A](#).

S1 Fig AU. Spatial distribution of adult *Leiostomus xanthurus* (Spot) on the Northeast U.S. Shelf Ecosystem. Change in distribution of adult *Leiostomus xanthurus* in the spring (A; ○) and fall (B; •) were examined in the along-shelf (C), cross-shelf (D), and depth (E) directions. *Leiostomus xanthurus* did not shift significantly.

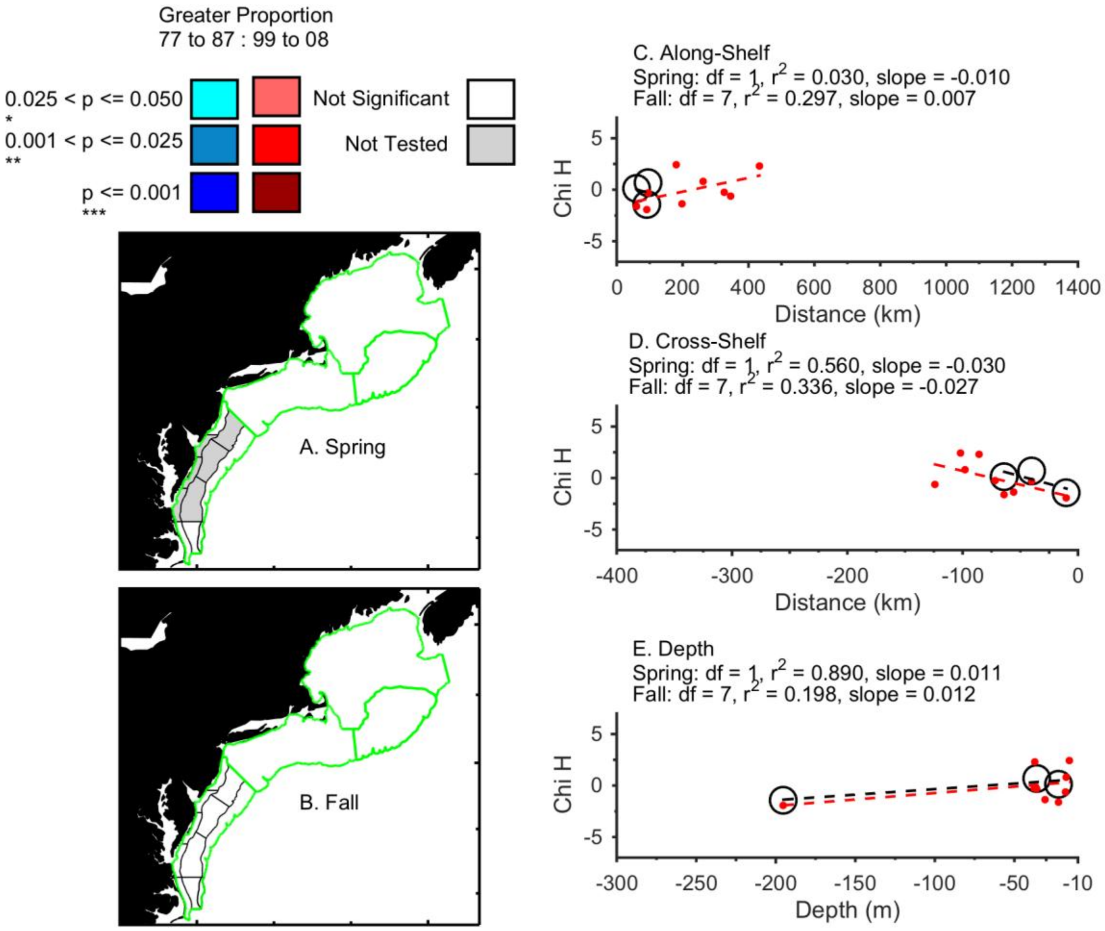

Return to S1 File Table A.

S1 Fig AV. Spatial and seasonal distribution of larval *Menticirrhus* spp. (Kingfishes) on the Northeast U.S. Shelf Ecosystem. Change in spatial distribution of larval *Menticirrhus* spp. in May – June (A; •), July – August (B; ○), and September – October (C; •) were examined in the along-shelf (D), cross-shelf (E), and depth (F) directions. Larval *Menticirrhus* spp. did not shift significantly spatially. Seasonal distribution shifted significantly later (G).

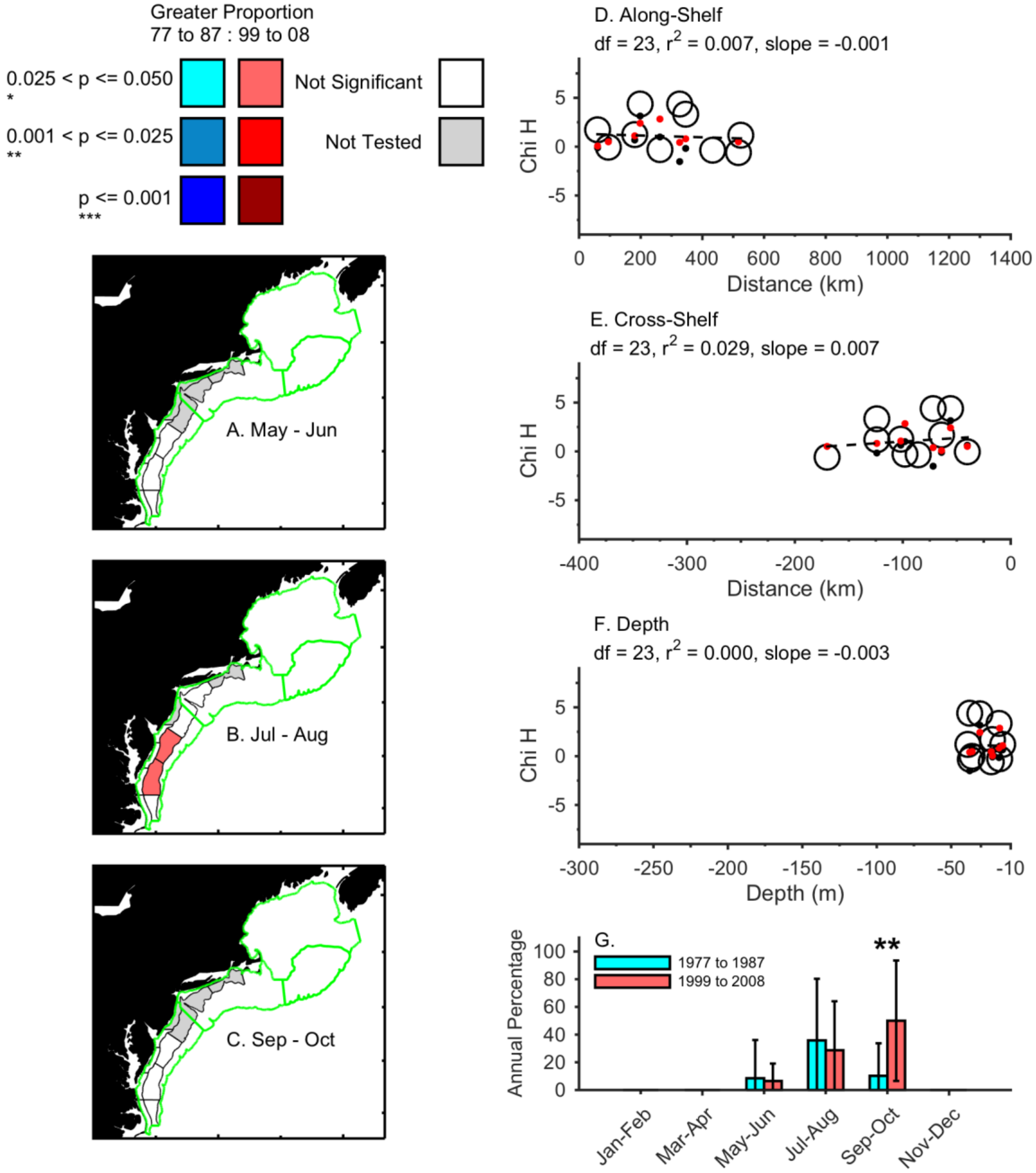

Return to S1 File Table A.

S1 Fig AW. Spatial and seasonal distribution of larval *Micropogonias undulatus* (Atlantic Croaker) on the Northeast U.S. Shelf Ecosystem. Change in spatial distribution of larval *Micropogonias undulatus* in July - August (A; •), September - October (B; ○), and November - December (C; •) were examined in the along-shelf (D), cross-shelf (E), and depth (F) directions. Larval *Micropogonias undulatus* shifted significantly northward (D) and inshore (E). Seasonal distribution did not shift significantly (G).

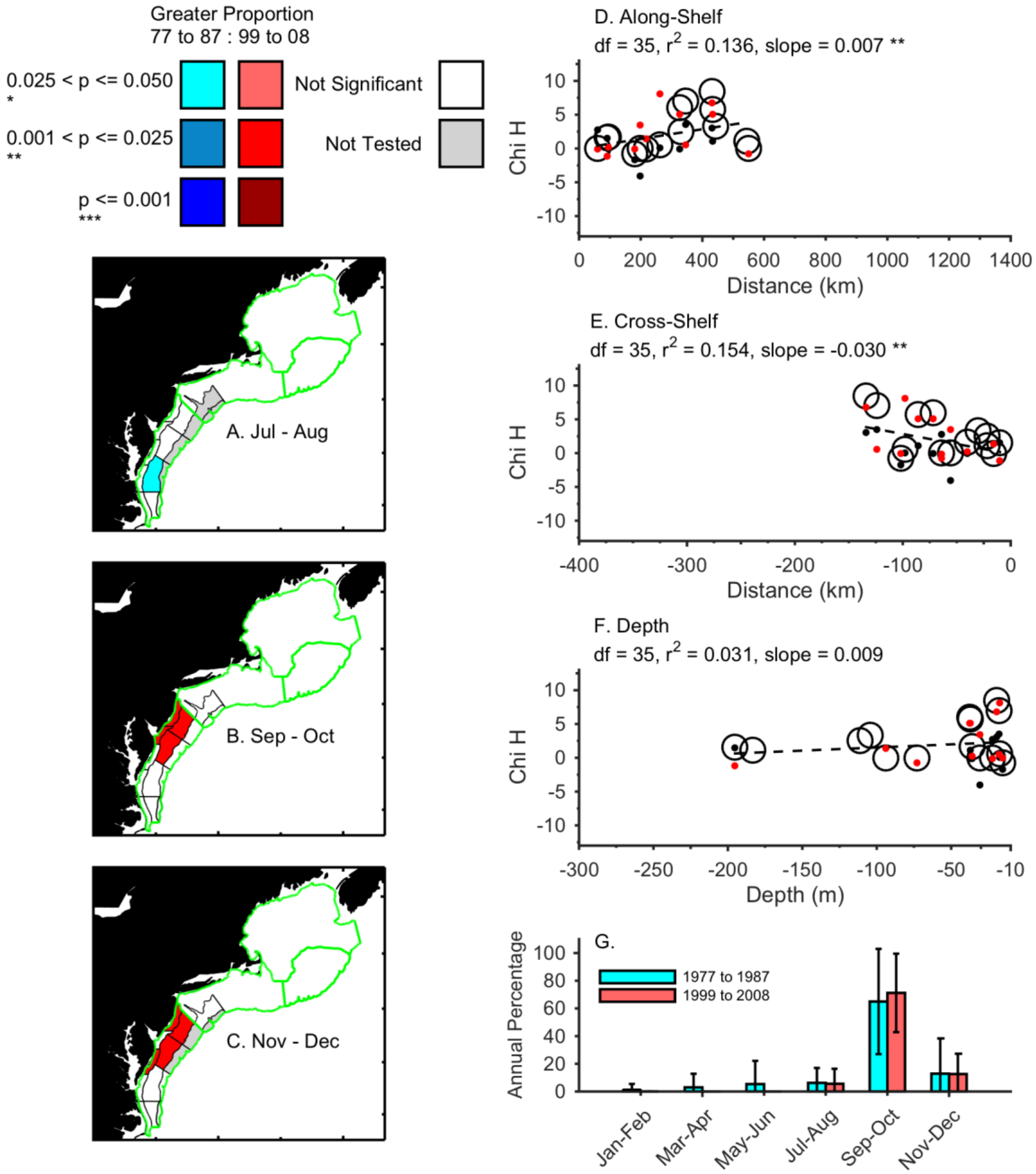

Return to S1 File Table A.

S1 Fig AX. Spatial distribution of adult *Micropogonias undulatus* (Atlantic Croaker) on the Northeast U.S. Shelf Ecosystem. Change in distribution of adult *Micropogonias undulatus* in the spring (A; ○) and fall (B; •) were examined in the along-shelf (C), cross-shelf (D), and depth (E) directions. *Micropogonias undulatus* shifted significantly inshore (D) in the fall.

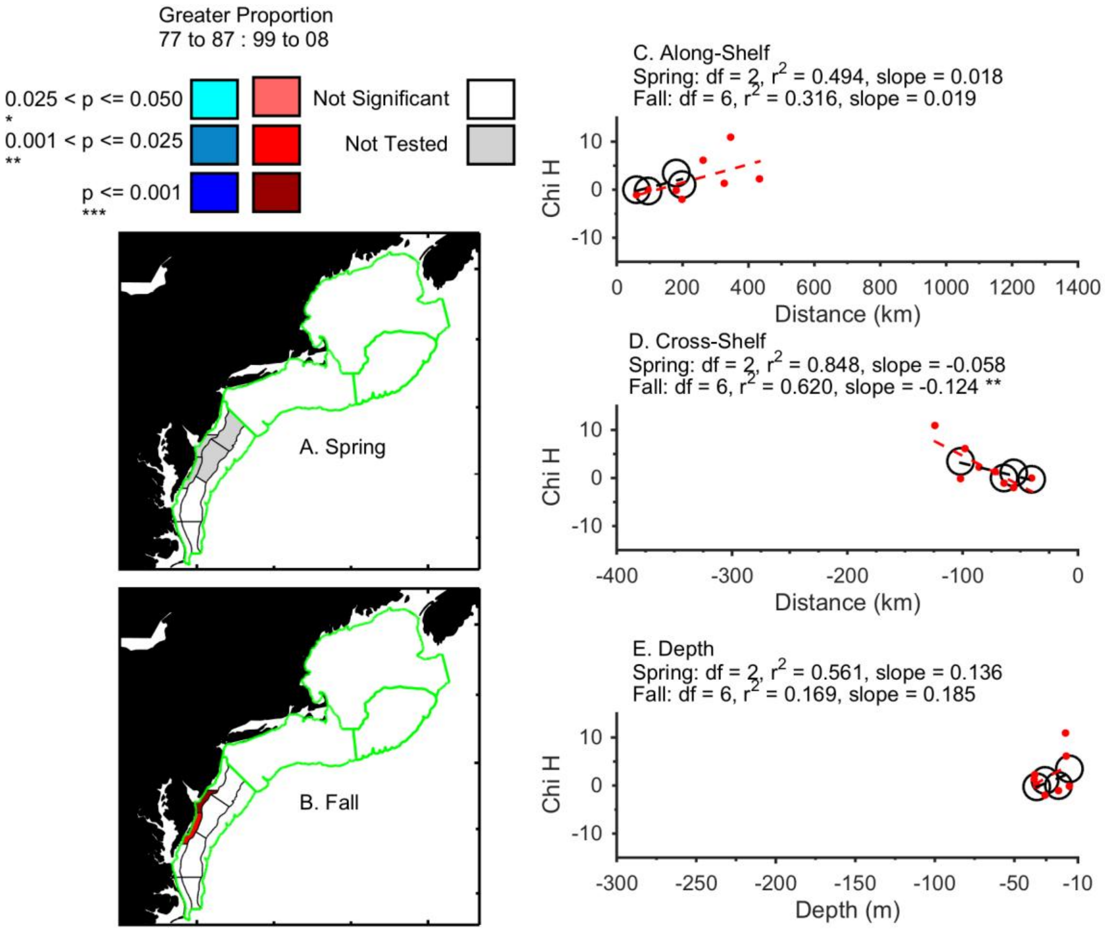

Return to S1 File Table A.

S1 Fig AY. Spatial and seasonal distribution of larval *Tautoga onitis* (Tautog) on the Northeast U.S. Shelf Ecosystem. Change in spatial distribution of larval *Tautoga onitis* in May – June (A; •), July – August (B; ○), and September – October (C; •) were examined in the along-shelf (D), cross-shelf (E), and depth (F) directions. Larval *Tautoga onitis* did not shift significantly spatially. Seasonal distribution shifted significantly earlier (G).

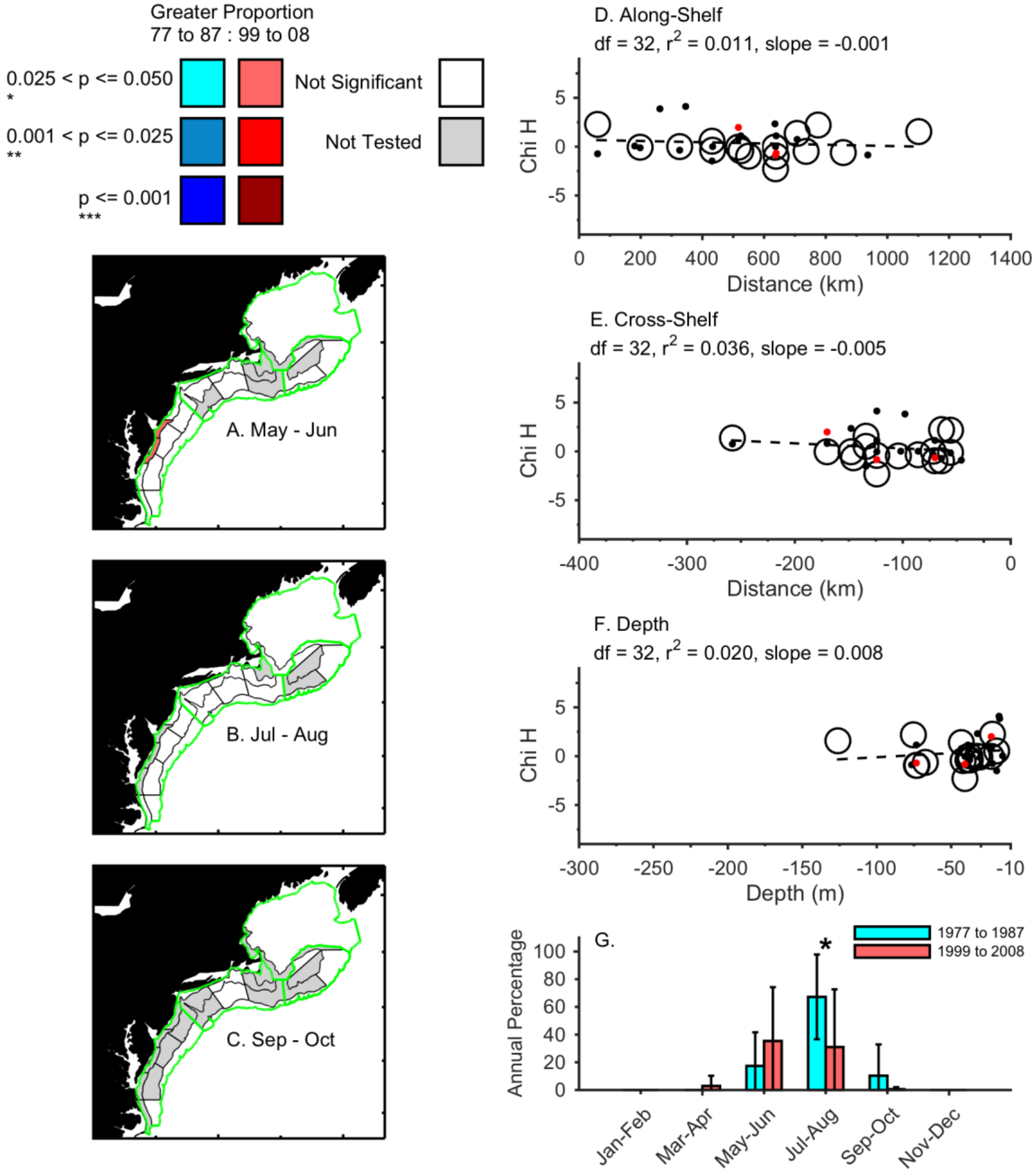

Return to S1 File Table A.

S1 Fig AZ. Spatial and seasonal distribution of larval *Tautogolabrus adspersus* (Cunner) on the Northeast U.S. Shelf Ecosystem. Change in spatial distribution of larval *Tautogolabrus adspersus* in May – June (A; •), July – August (B; ○), and September – October (C; •) were examined in the along-shelf (D), cross-shelf (E), and depth (F) directions. Larval *Tautogolabrus adspersus* shifted significantly northward (D). Seasonal distribution did not shift significantly (G).

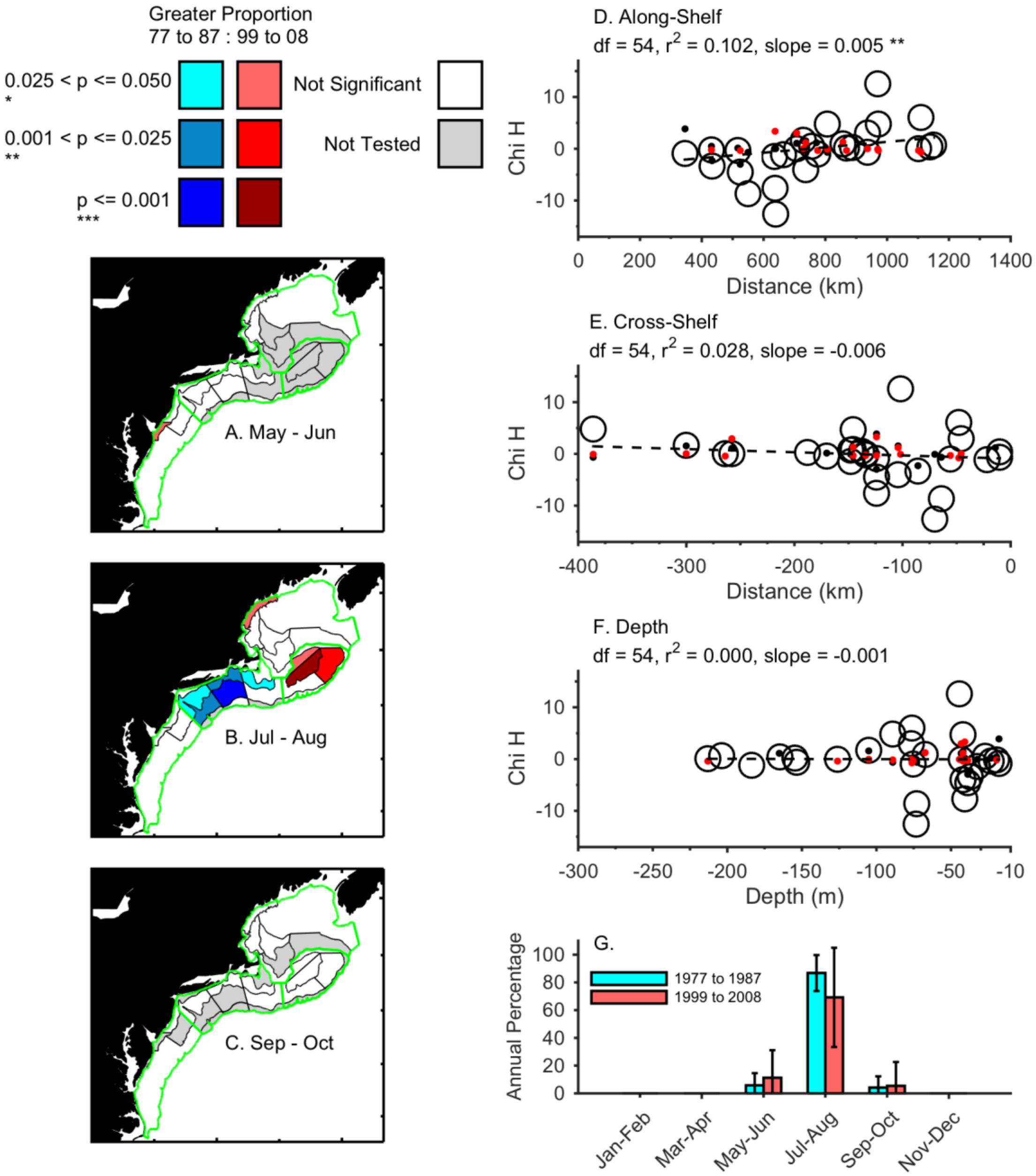

Return to S1 File Table A.

S1 Fig BA. Spatial distribution of adult *Zoarces americanus* (Ocean Pout) on the Northeast U.S. Shelf Ecosystem. Change in distribution of adult *Zoarces americanus* in the spring (A; ○) and fall (B; •) were examined in the along-shelf (C), cross-shelf (D), and depth (E) directions. *Zoarces americanus* shifted significantly northward (C) in the fall.

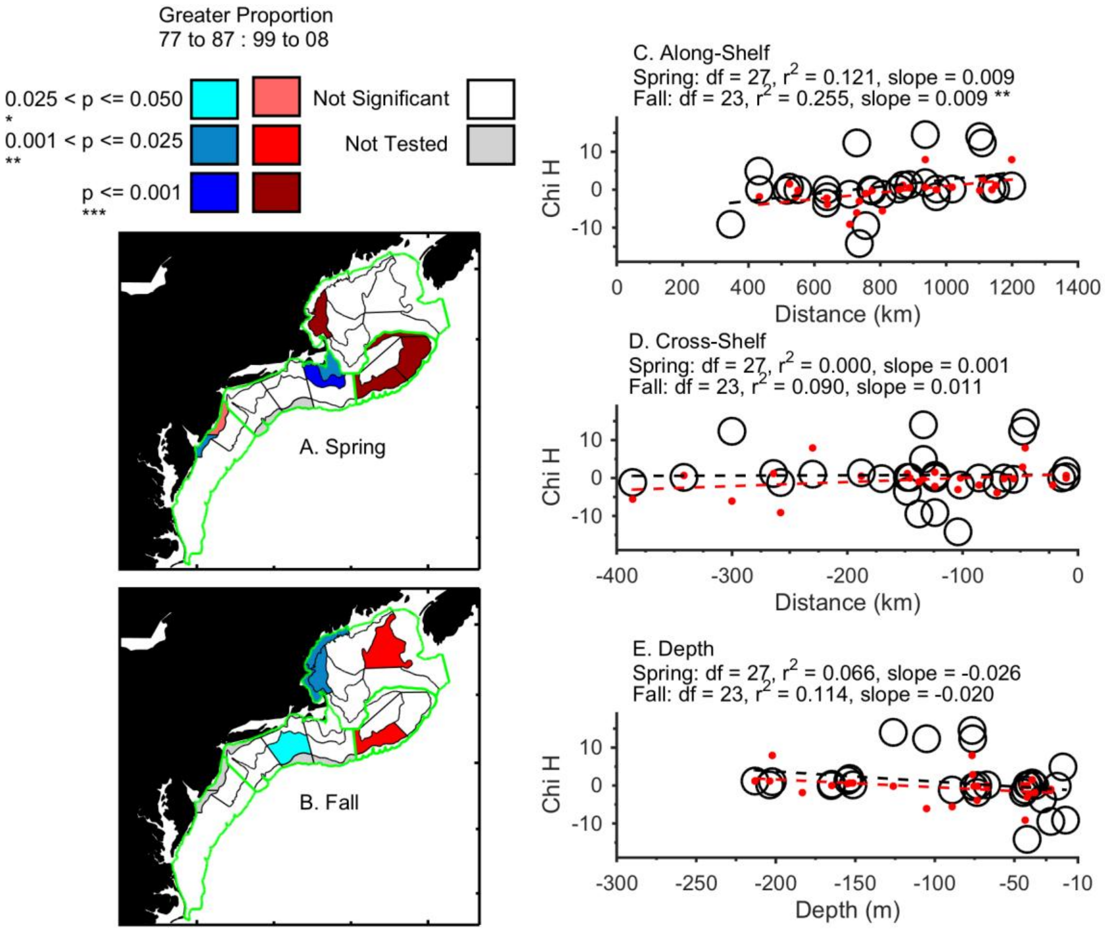

Return to S1 File Table A.

S1 Fig BB. Spatial and seasonal distribution of larval *Ulvaria subbifurcata* (Radiated Shanny) on the Northeast U.S. Shelf Ecosystem. Change in spatial distribution of larval *Ulvaria subbifurcata* in March – April (A; •), May – June (B; ○), and July – August (C; •) were examined in the along-shelf (D), cross-shelf (E), and depth (F) directions. Larval *Ulvaria subbifurcata* did not shift significantly spatially or seasonally (G).

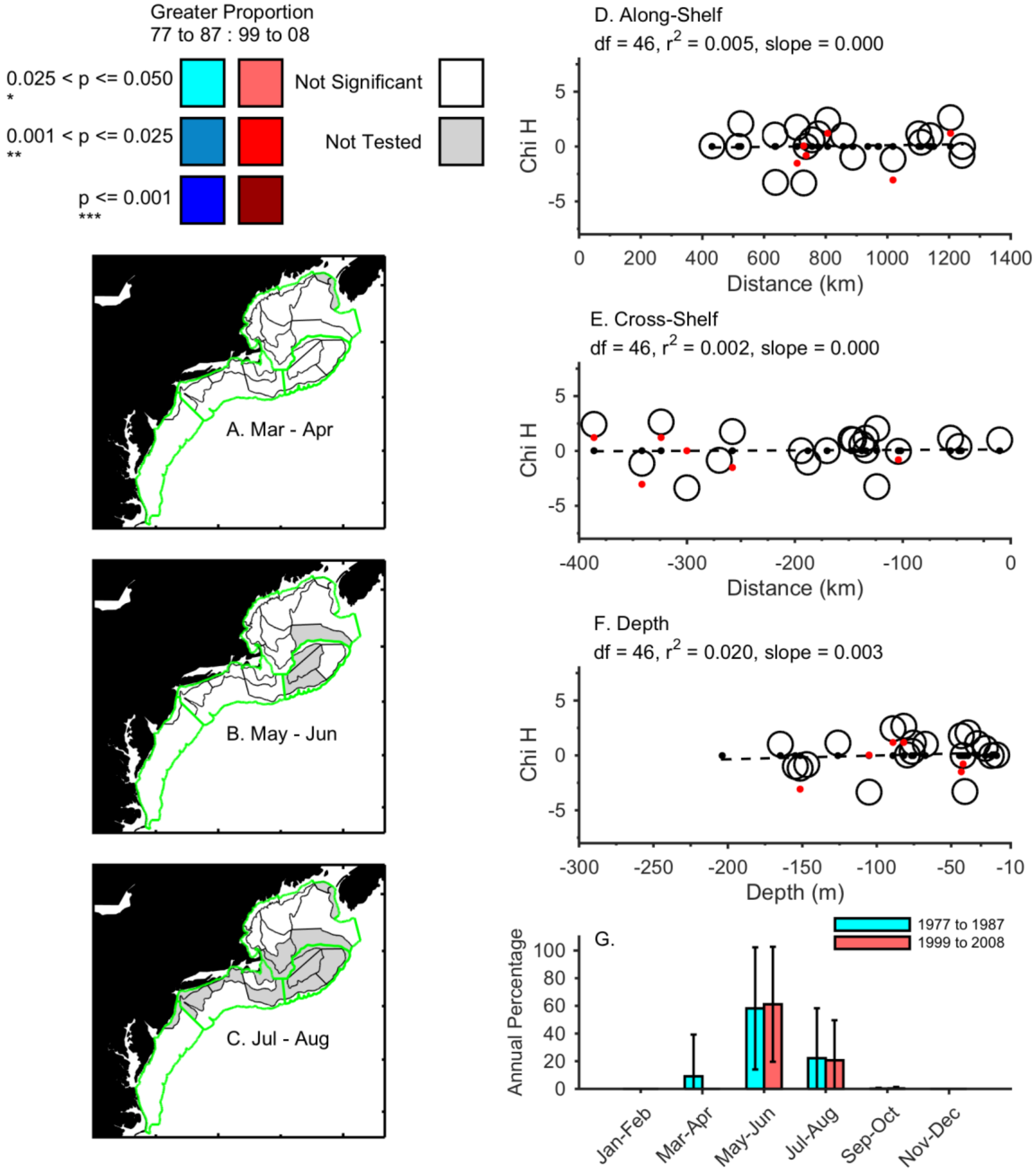

Return to S1 File Table A.

S1 Fig BC. Spatial and seasonal distribution of larval *Pholis gunnellus* (Rock Gunnel) on the Northeast U.S. Shelf Ecosystem. Change in spatial distribution of larval *Pholis gunnellus* in January – February (A; •), March – April (B; ○), and May – June (C; •) were examined in the along-shelf (D), cross-shelf (E), and depth (F) directions. Larval *Pholis gunnellus* did not shift significantly spatially. Seasonal distribution shifted significantly earlier (G).

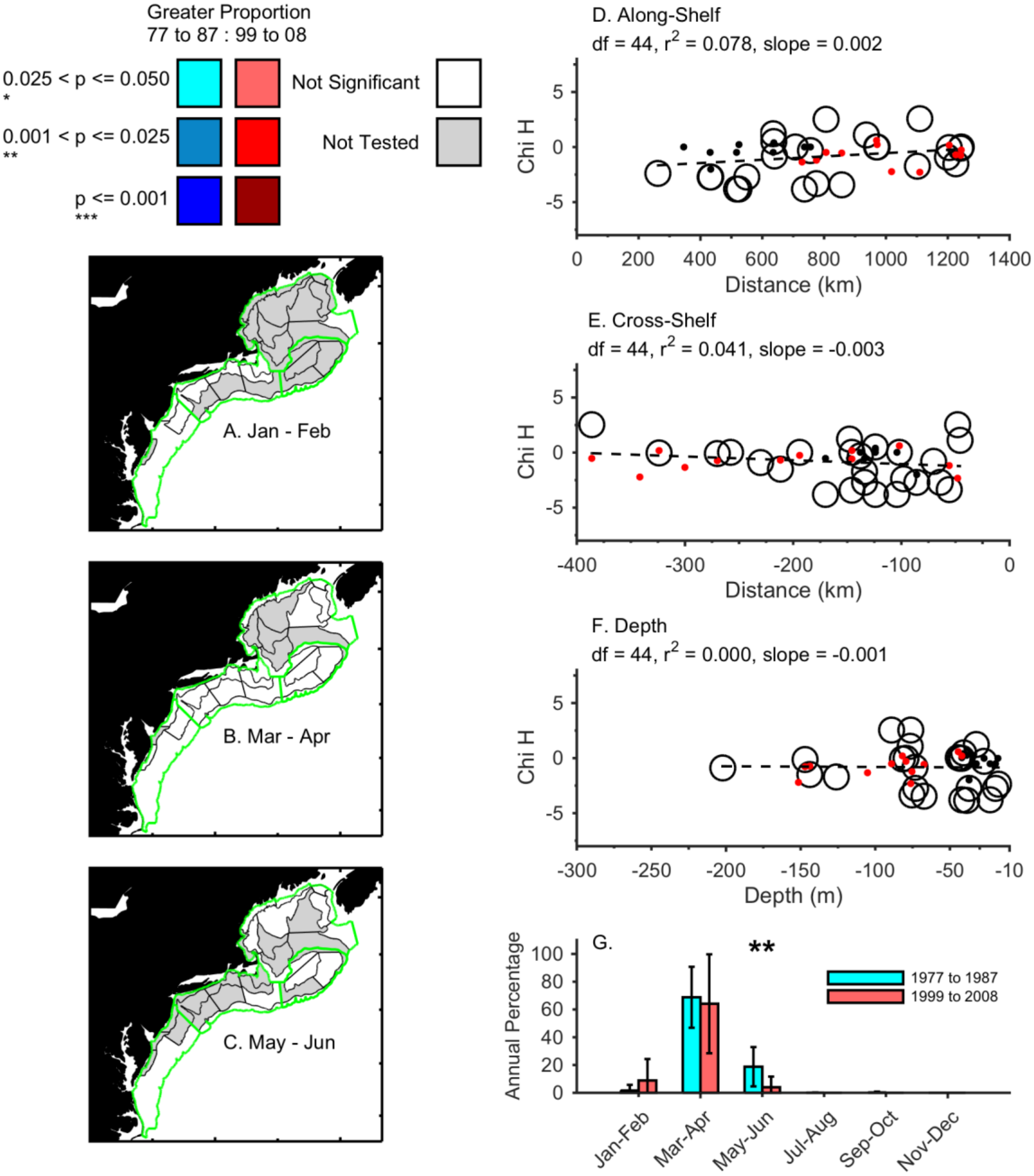

[Return to S1 File Table A](#).

S1 Fig BD. Spatial and seasonal distribution of larval *Anarhichas* spp. (Atlantic Wolffish) on the Northeast U.S. Shelf Ecosystem. Change in spatial distribution of larval *Anarhichas* spp. in January – February (A; •), March – April (B; ○), and May – June (C; •) were examined in the along-shelf (D), cross-shelf (E), and depth (F) directions. Larval *Anarhichas* spp. did not shift significantly spatially. Seasonal distribution shifted significantly earlier (G).

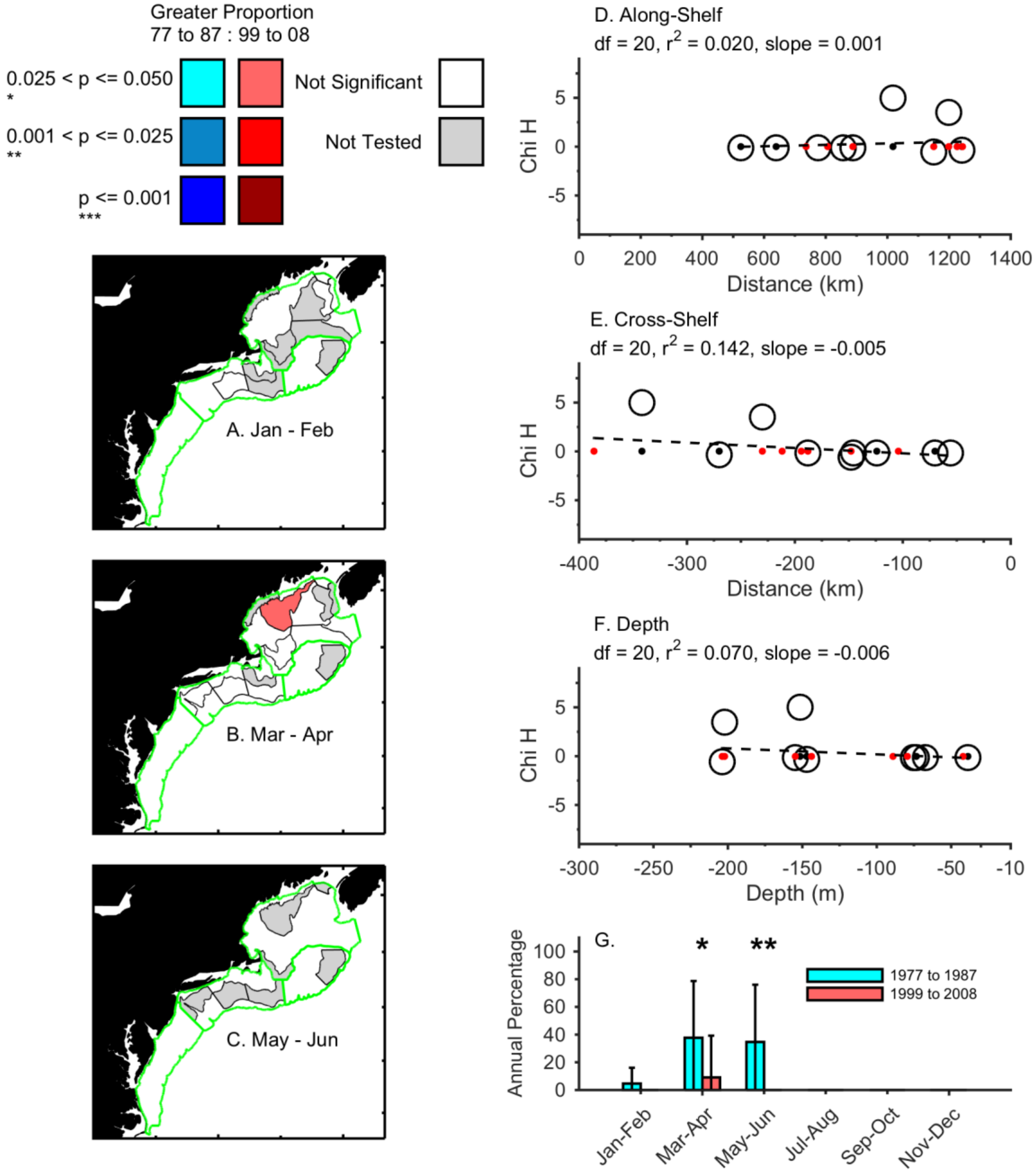

Return to S1 File Table A.

S1 Fig BE. Spatial distribution of adult *Anarhichas* spp. (Atlantic Wolffish) on the Northeast U.S. Shelf Ecosystem. Change in distribution of adult *Anarhichas* spp. in the spring (A; ○) and fall (B; •) were examined in the along-shelf (C), cross-shelf (D), and depth (E) directions. *Anarhichas* spp. shifted significantly inshore (D) in the fall.

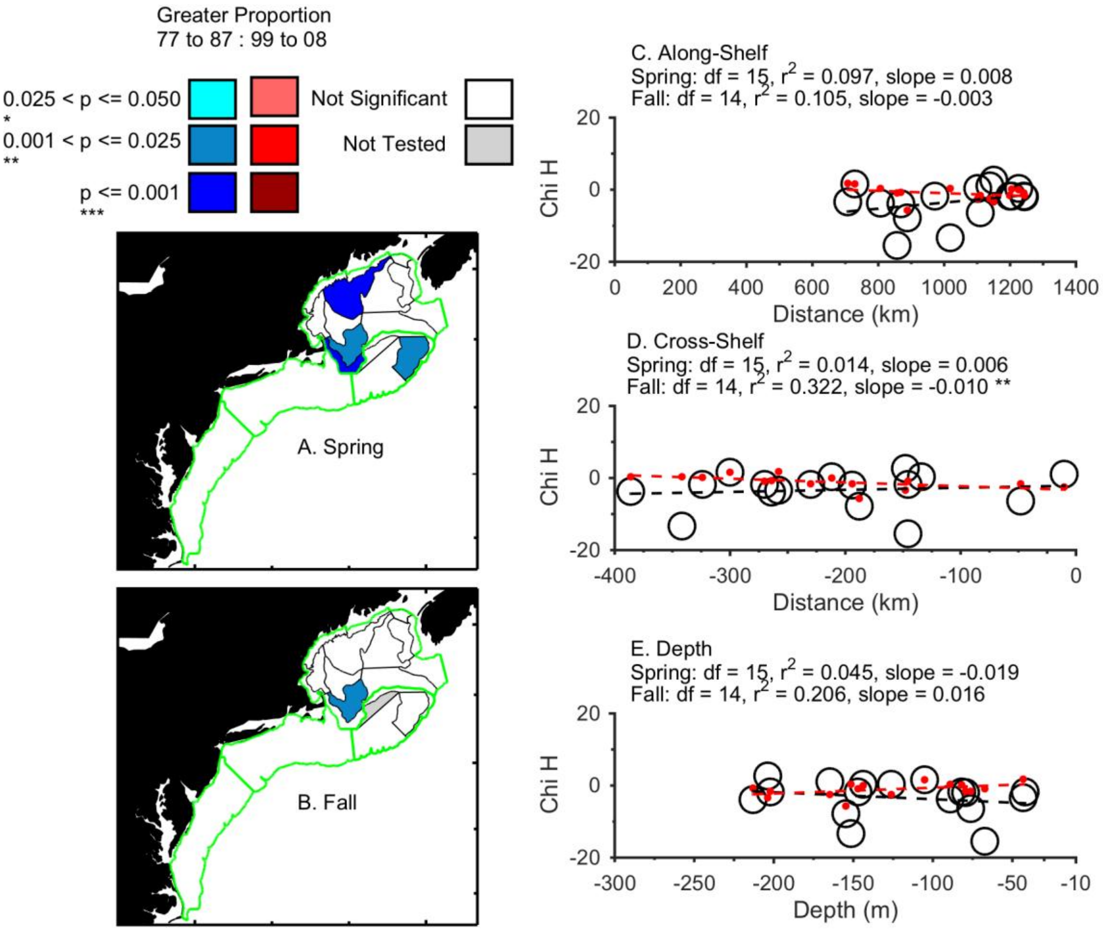

Return to S1 File Table A.

S1 Fig BF. Spatial and seasonal distribution of larval *Ammodytes* spp. (Sand Lances) on the Northeast U.S. Shelf Ecosystem. Change in spatial distribution of larval *Ammodytes* spp. in November – December (A; •), January – February (B; ○), and March – April (C; •) were examined in the along-shelf (D), cross-shelf (E), and depth (F) directions. Larval *Ammodytes* spp. shifted significantly northward (D) and deeper (F) spatially and significantly later (G) seasonally.

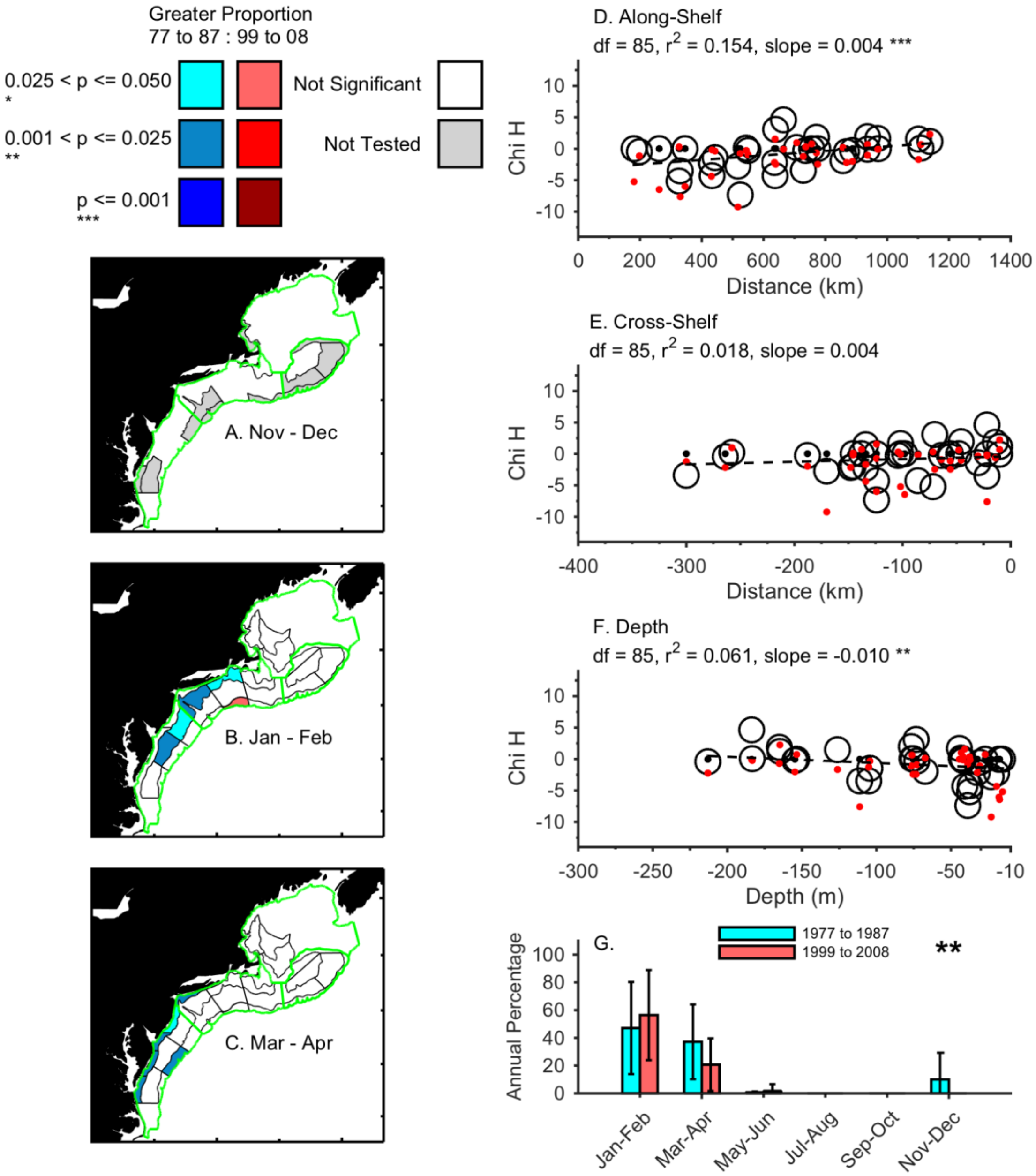

Return to S1 File Table A.

S1 Fig BG. Spatial distribution of adult *Ammodytes* spp. (Sand Lances) on the Northeast U.S. Shelf Ecosystem. Change in distribution of adult *Ammodytes* spp. in the spring (A; ○) and fall (B; •) were examined in the along-shelf (C), cross-shelf (D), and depth (E) directions. *Ammodytes* spp. did not shift significantly.

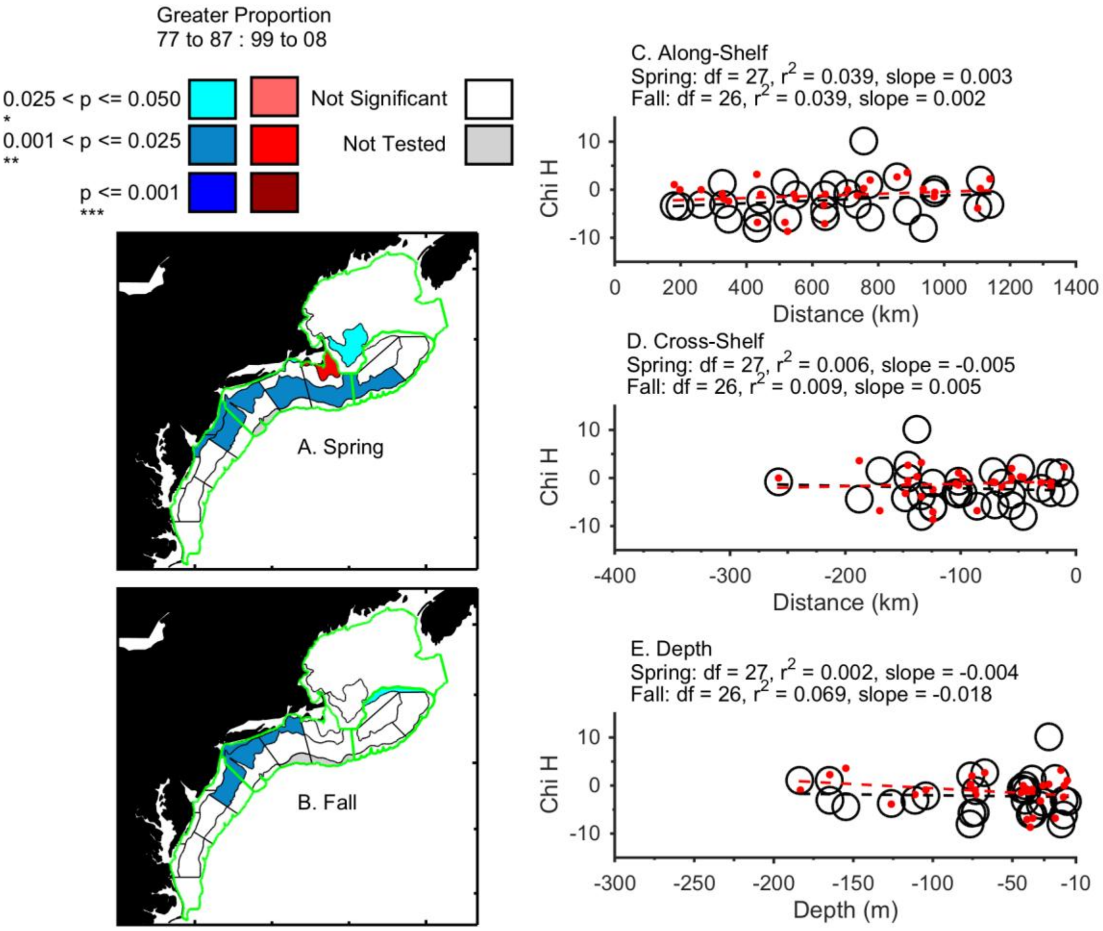

Return to S1 File Table A.

S1 Fig BH. Spatial and seasonal distribution of larval *Auxis* spp. (Bullet Mackerels) on the Northeast U.S. Shelf Ecosystem. Change in spatial distribution of larval *Auxis* spp. in May – June (A; •), July – August (B; ○), and September – October (C; •) were examined in the along-shelf (D), cross-shelf (E), and depth (F) directions. Larval *Auxis* spp. shifted significantly inshore (E). Larval *Auxis* spp. did not significantly shift seasonally (G).

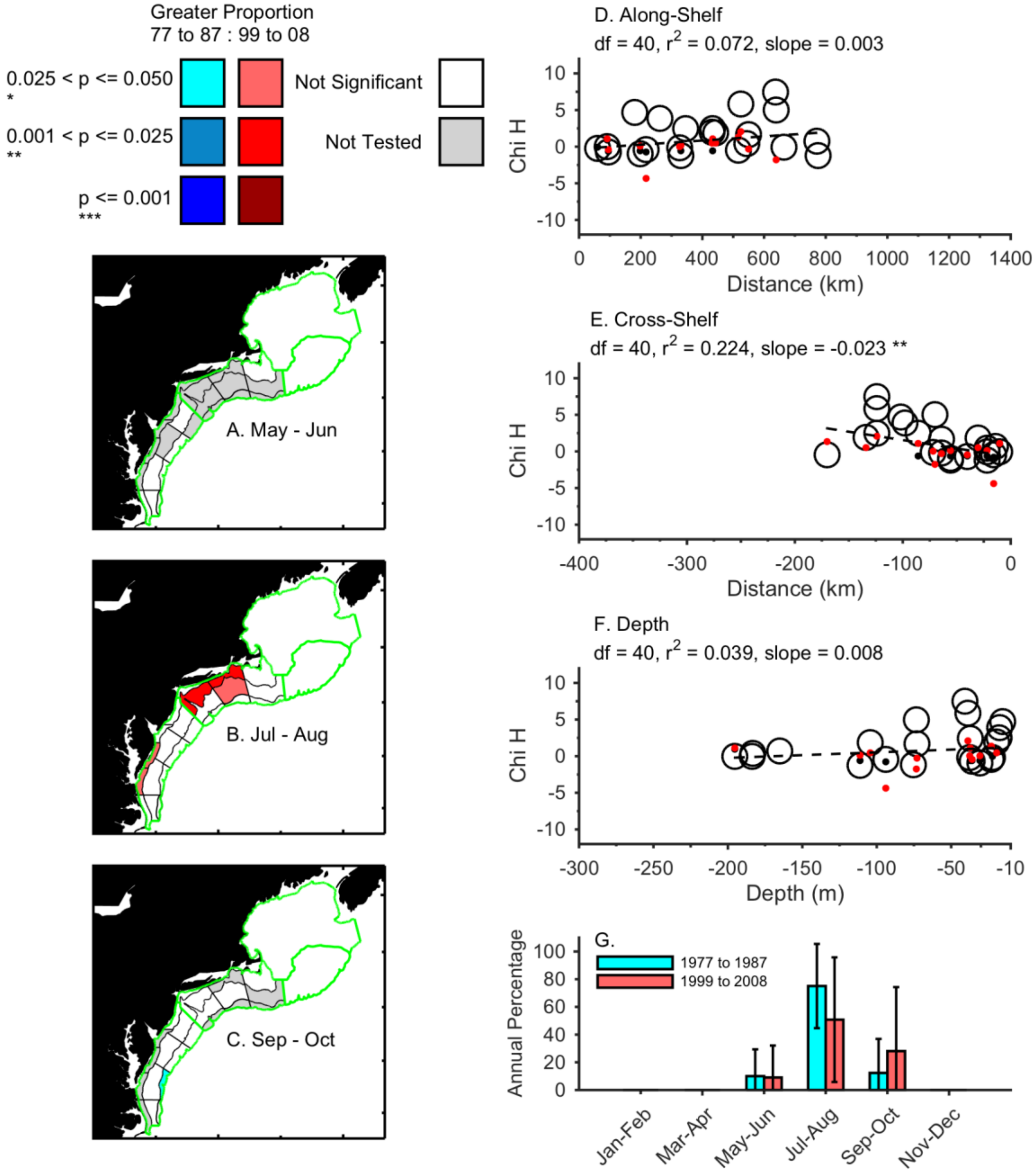

[Return to S1 File Table A](#).

S1 Fig BI. Spatial and seasonal distribution of larval *Scomber scombrus* (Atlantic Mackerel) on the Northeast U.S. Shelf Ecosystem. Change in spatial distribution of larval *Scomber scombrus* in May - June (A; •), July - August (B; ○), and September - October (C; •) were examined in the along-shelf (D), cross-shelf (E), and depth (F) directions. Larval *Scomber scombrus* shifted significantly inshore (E) and later in the season (G).

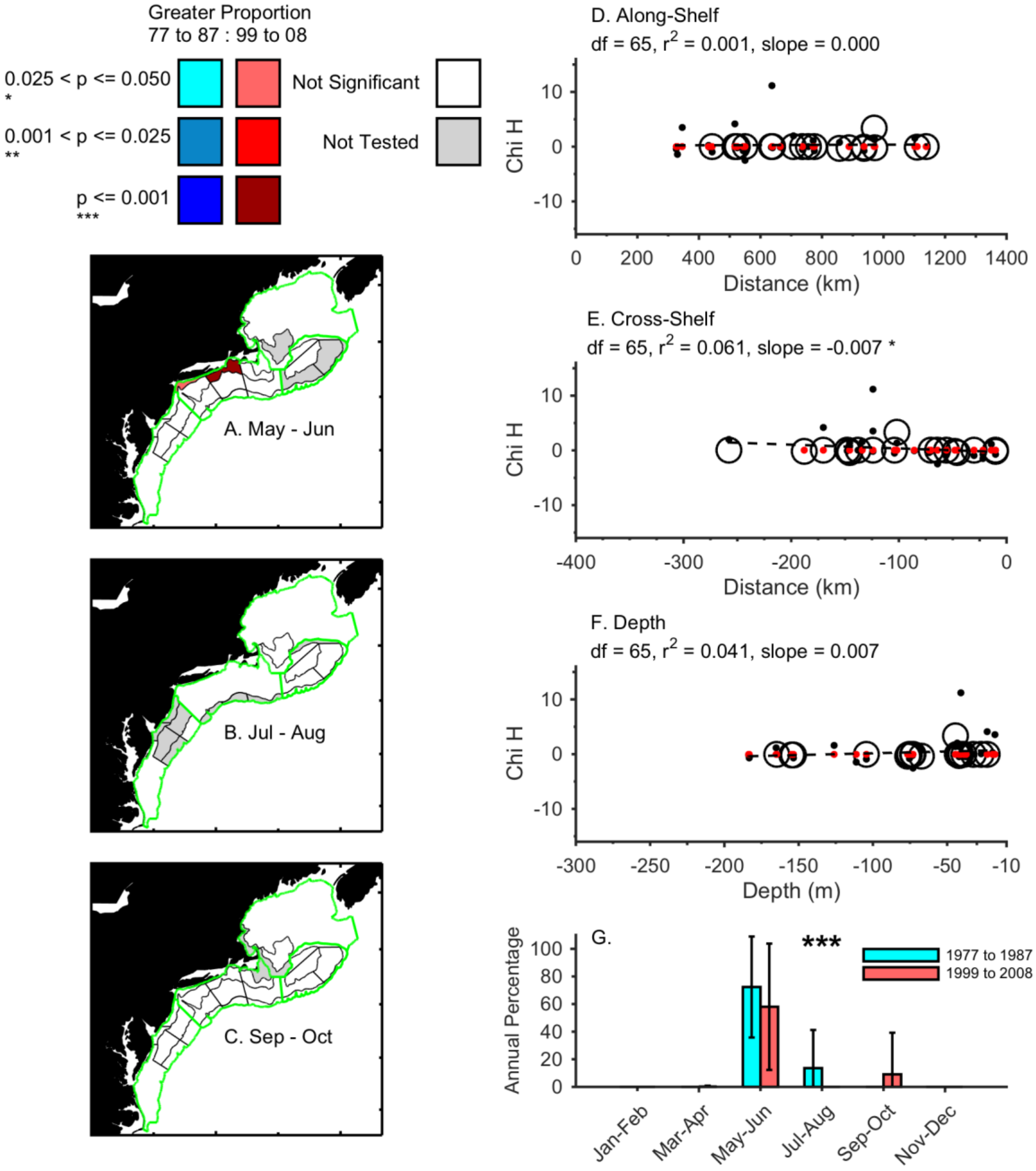

Return to S1 File Table A.

S1 Fig BJ. Spatial distribution of adult *Scomber scombrus* (Atlantic Mackerel) on the Northeast U.S. Shelf Ecosystem. Change in distribution of adult *Scomber scombrus* in the spring (A; ○) and fall (B; •) were examined in the along-shelf (C), cross-shelf (D), and depth (E) directions. *Scomber scombrus* distribution shifted significantly northward (C) and inshore (D) in the spring and northward (C) in the fall.

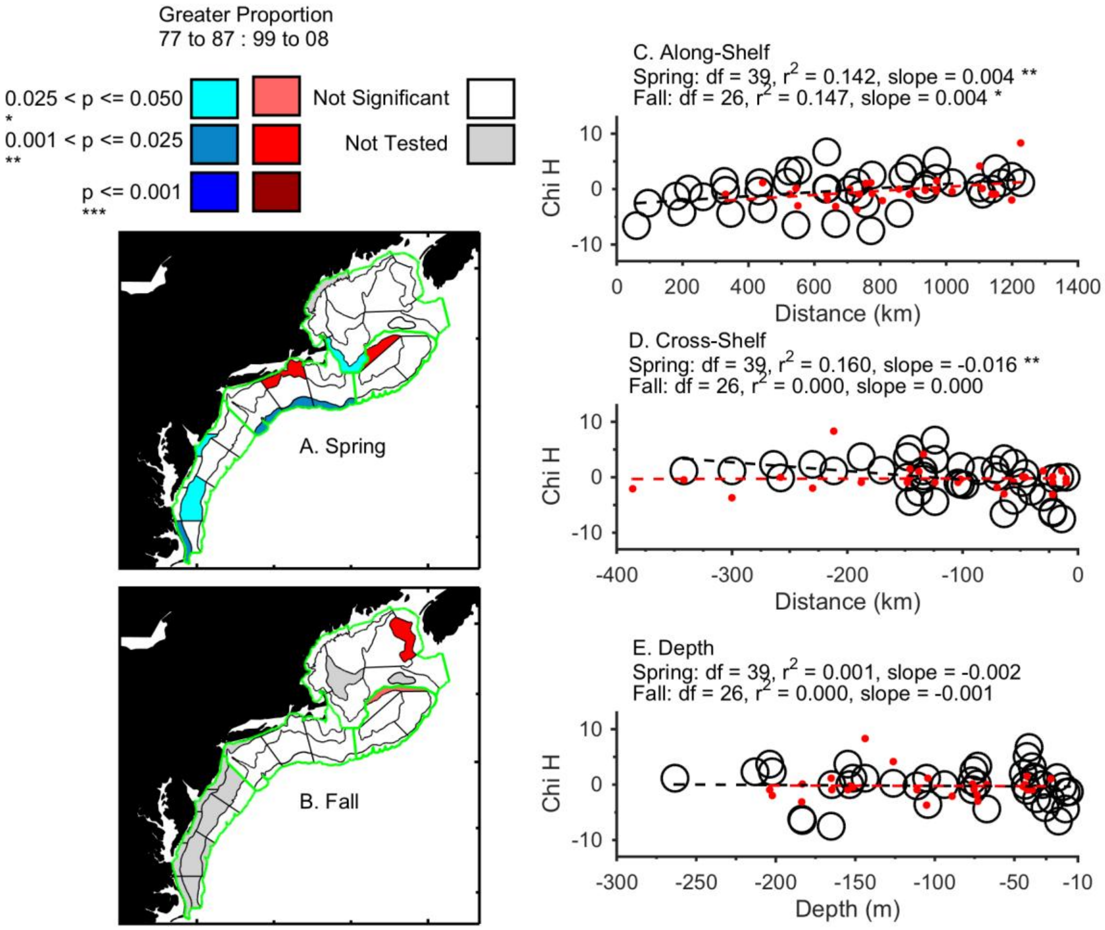

Return to S1 File Table A.

S1 Fig BK. Spatial and seasonal distribution of larval *Peprilus* spp. (Butterfishes) on the Northeast U.S. Shelf Ecosystem. Change in spatial distribution of larval *Peprilus* spp. in May - June (A; •), July - August (B; ○), and September - October (C; •) were examined in the along-shelf (D), cross-shelf (E), and depth (F) directions. Larval *Peprilus* spp. did not shift significantly spatially or seasonally (G).

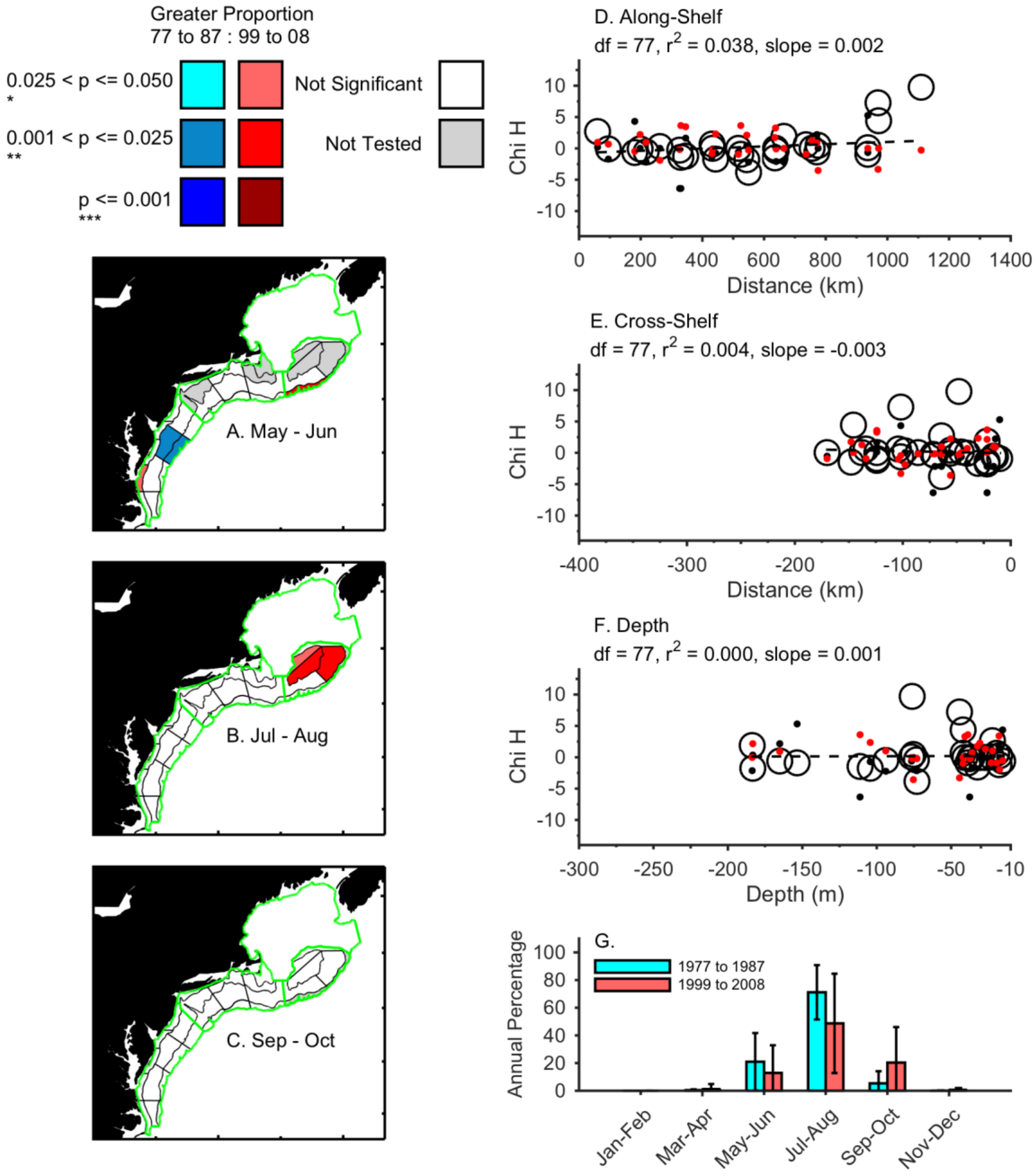

[Return to S1 File Table A](#).

S1 Fig BL. Spatial distribution of adult *Peprilus* spp. (Butterfishes) on the Northeast U.S. Shelf Ecosystem. Change in distribution of adult *Peprilus* spp. in the spring (A; ○) and fall (B; •) were examined in the along-shelf (C), cross-shelf (D), and depth (E) directions. *Peprilus* spp. distribution shifted significantly inshore (D) in the spring and fall and shallower (E) in the fall.

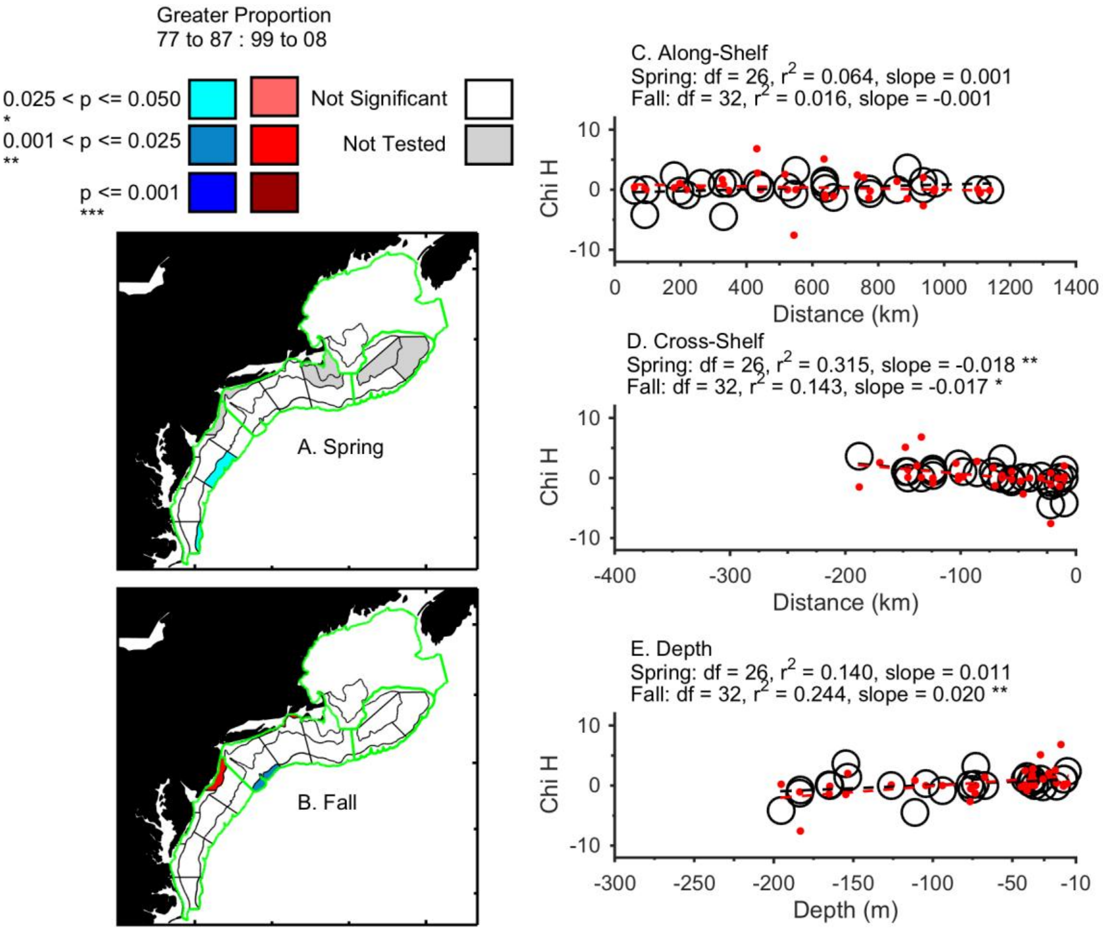

Return to S1 File Table A.

S1 Fig BM. Spatial and seasonal distribution of larval *Scophthalmus aquosus* (Windowpane) on the Northeast U.S. Shelf Ecosystem. Change in spatial distribution of larval *Scophthalmus aquosus* in May - June (A; •), July - August (B; ○), and September - October (C; •) were examined in the along-shelf (D), cross-shelf (E), and depth (F) directions. Larval *Scophthalmus aquosus* shifted significantly northward (D) spatially and earlier seasonally (G).

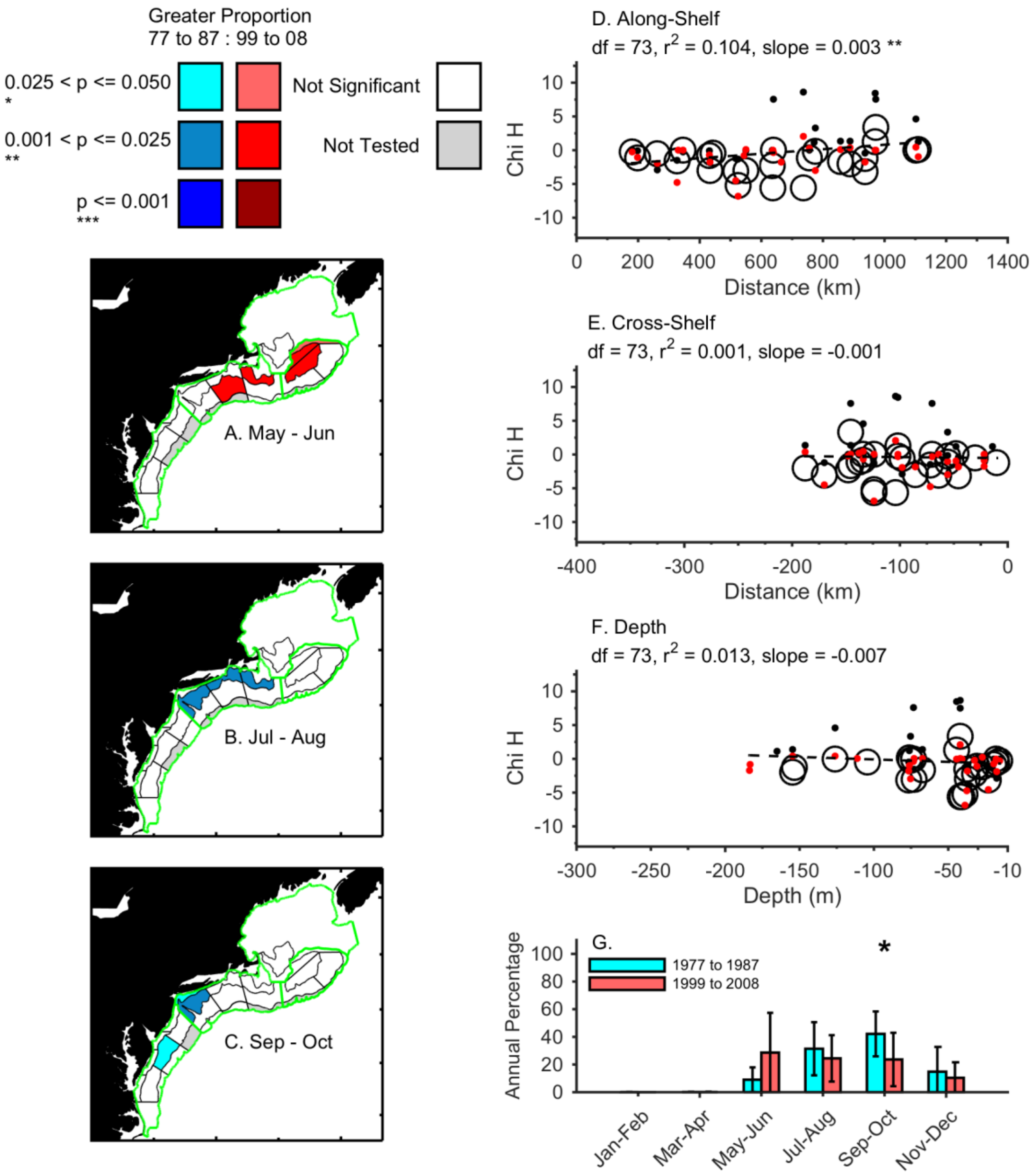

[Return to S1 File Table A](#).

S1 Fig BN. Spatial distribution of adult *Scophthalmus aquosus* (Windowpane) on the Northeast U.S. Shelf Ecosystem. Change in distribution of adult *Scophthalmus aquosus* in the spring (A; ○) and fall (B; •) were examined in the along-shelf (C), cross-shelf (D), and depth (E) directions. *Scophthalmus aquosus* distribution shifted significantly northward (D) in the spring.

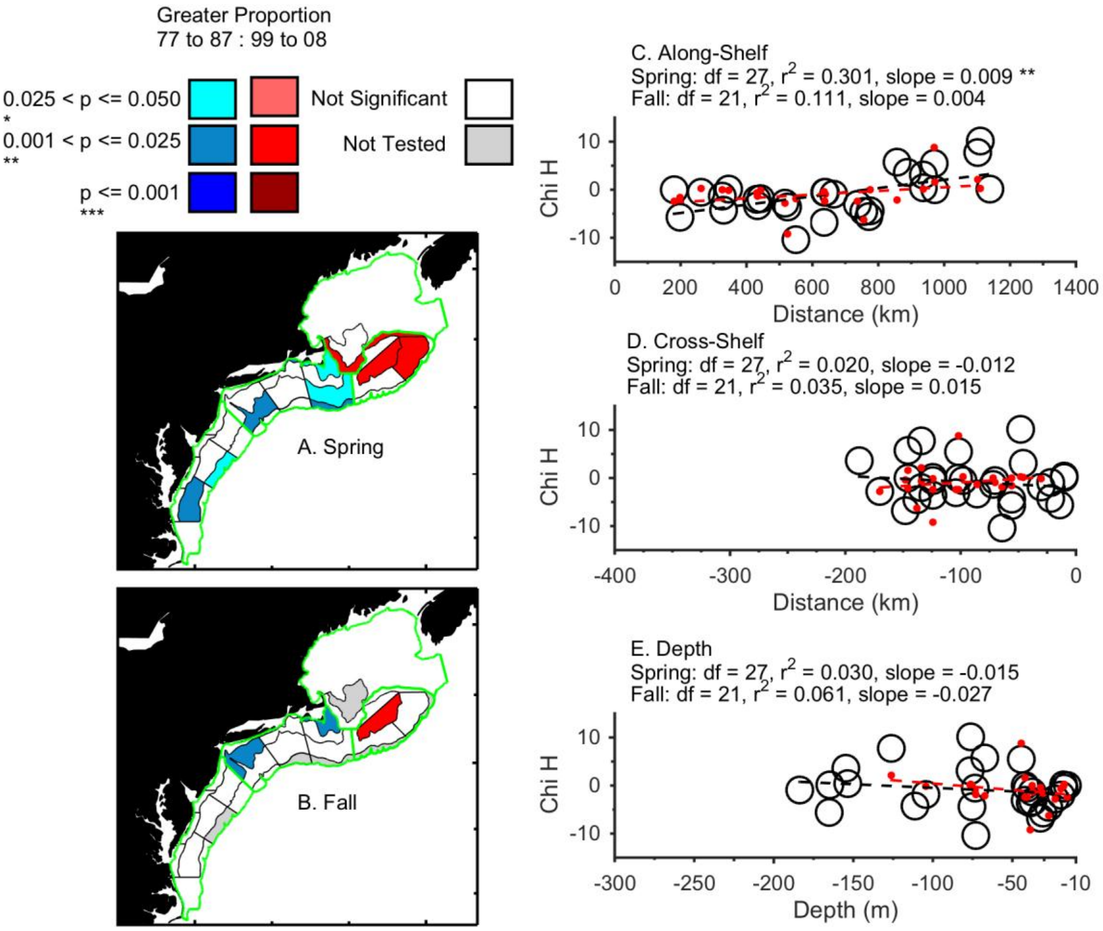

Return to S1 File Table A.

S1 Fig BO. Spatial and seasonal distribution of larval *Citharichthys arctifrons* (Gulf Stream Flounder) on the Northeast U.S. Shelf Ecosystem. Change in spatial distribution of larval *Citharichthys arctifrons* in July - August (A; •), September - October (B; ○), and November - December (C; •) were examined in the along-shelf (D), cross-shelf (E), and depth (F) directions. Larval *Citharichthys arctifrons* shifted significantly northward (D) and inshore (E). Seasonal distribution shifted significantly later (G).

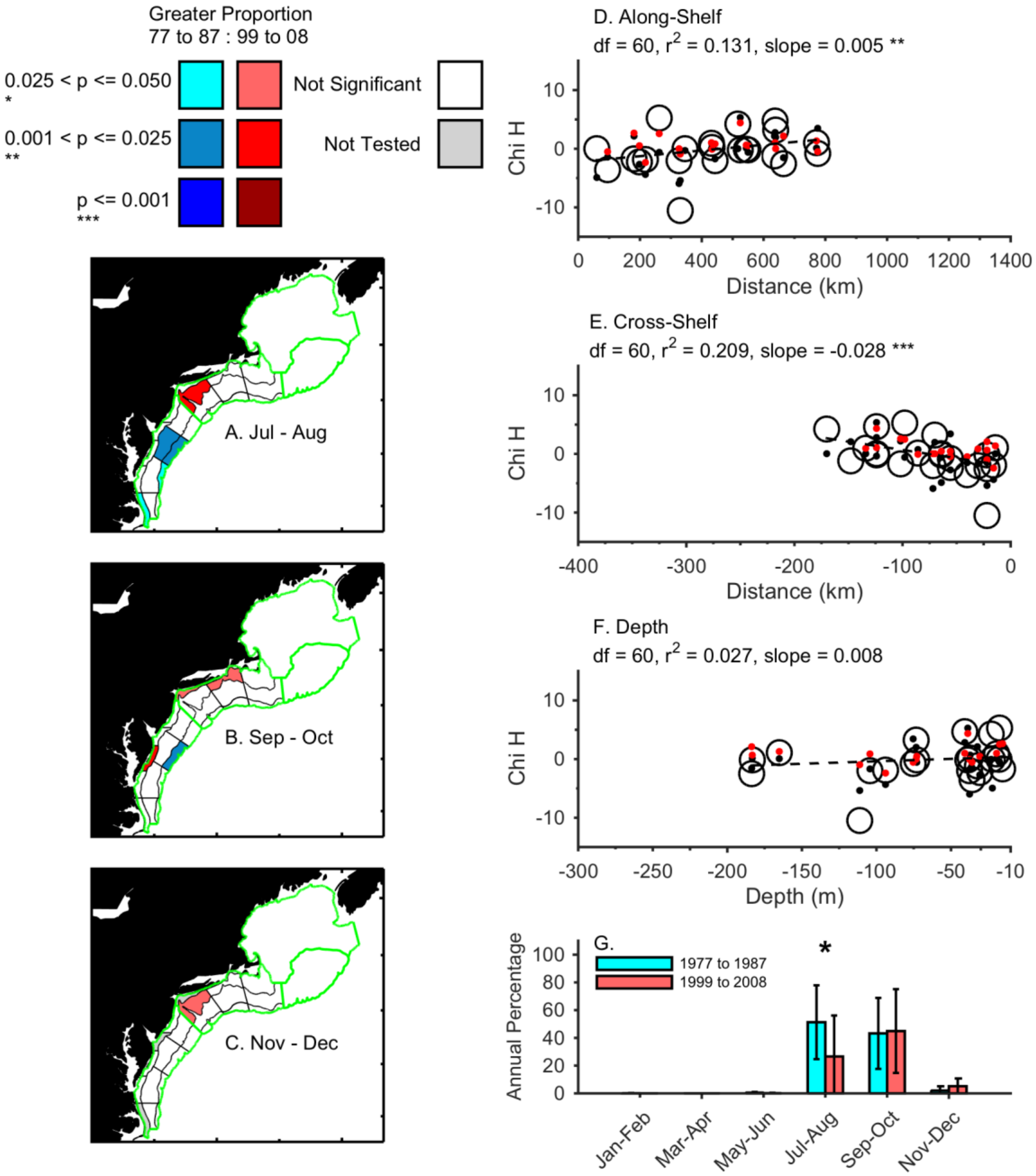

Return to S1 File Table A.

S1 Fig BP. Spatial distribution of adult *Citharichthys arctifrons* (Gulf Stream Flounder) on the Northeast U.S. Shelf Ecosystem. Change in distribution of adult *Citharichthys arctifrons* in the spring (A; ○) and fall (B; •) were examined in the along-shelf (C), cross-shelf (D), and depth (E) directions. *Citharichthys arctifrons* shifted significantly northward (C) in the spring and inshore (D) in the fall.

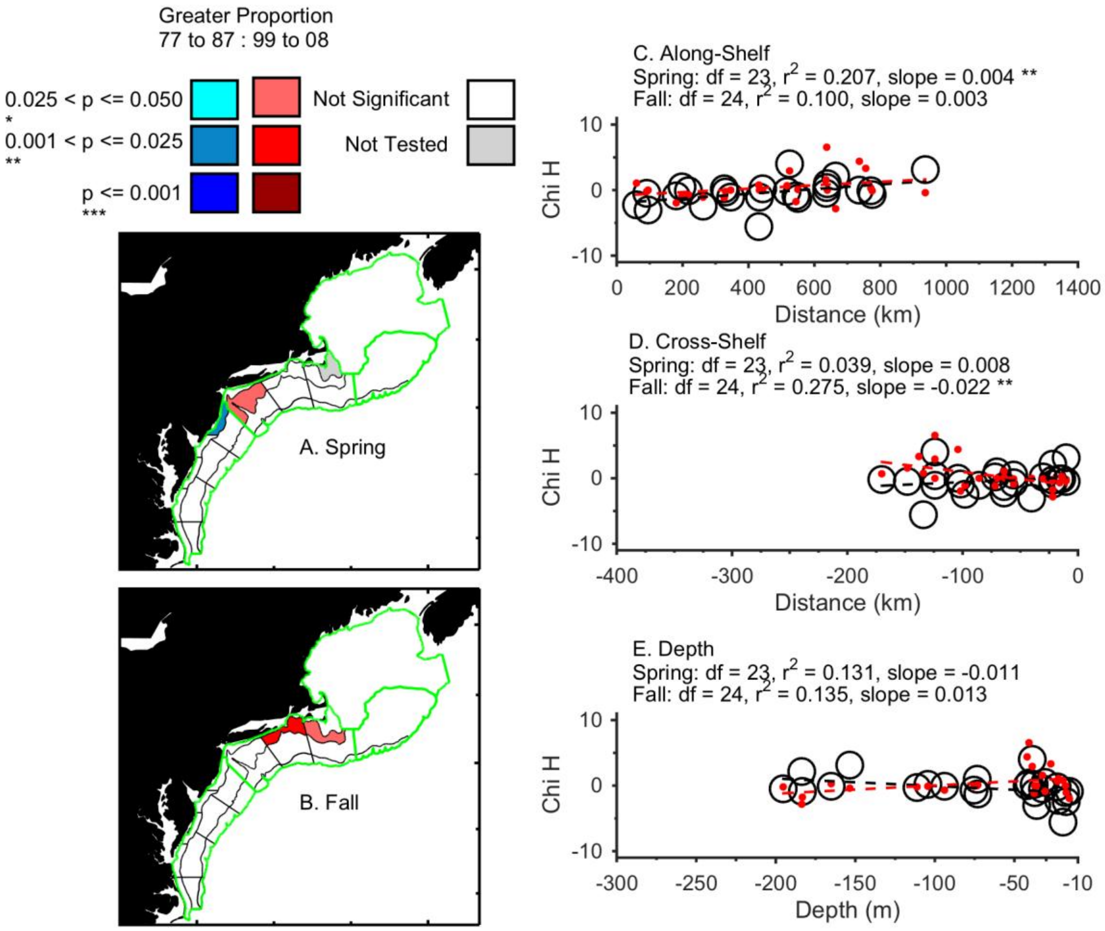

Return to S1 File Table A.

S1 Fig BQ. Spatial and seasonal distribution of larval *Etropus* spp. (Smallmouth Flounders) on the Northeast U.S. Shelf Ecosystem. Change in spatial distribution of larval *Etropus* spp. in July - August (A; •), September - October (B; ○), and November - December (C; •) were examined in the along-shelf (D), cross-shelf (E), and depth (F) directions. Larval *Etropus* spp. shifted significantly northward (D). Seasonal distribution did not shift significantly (G).

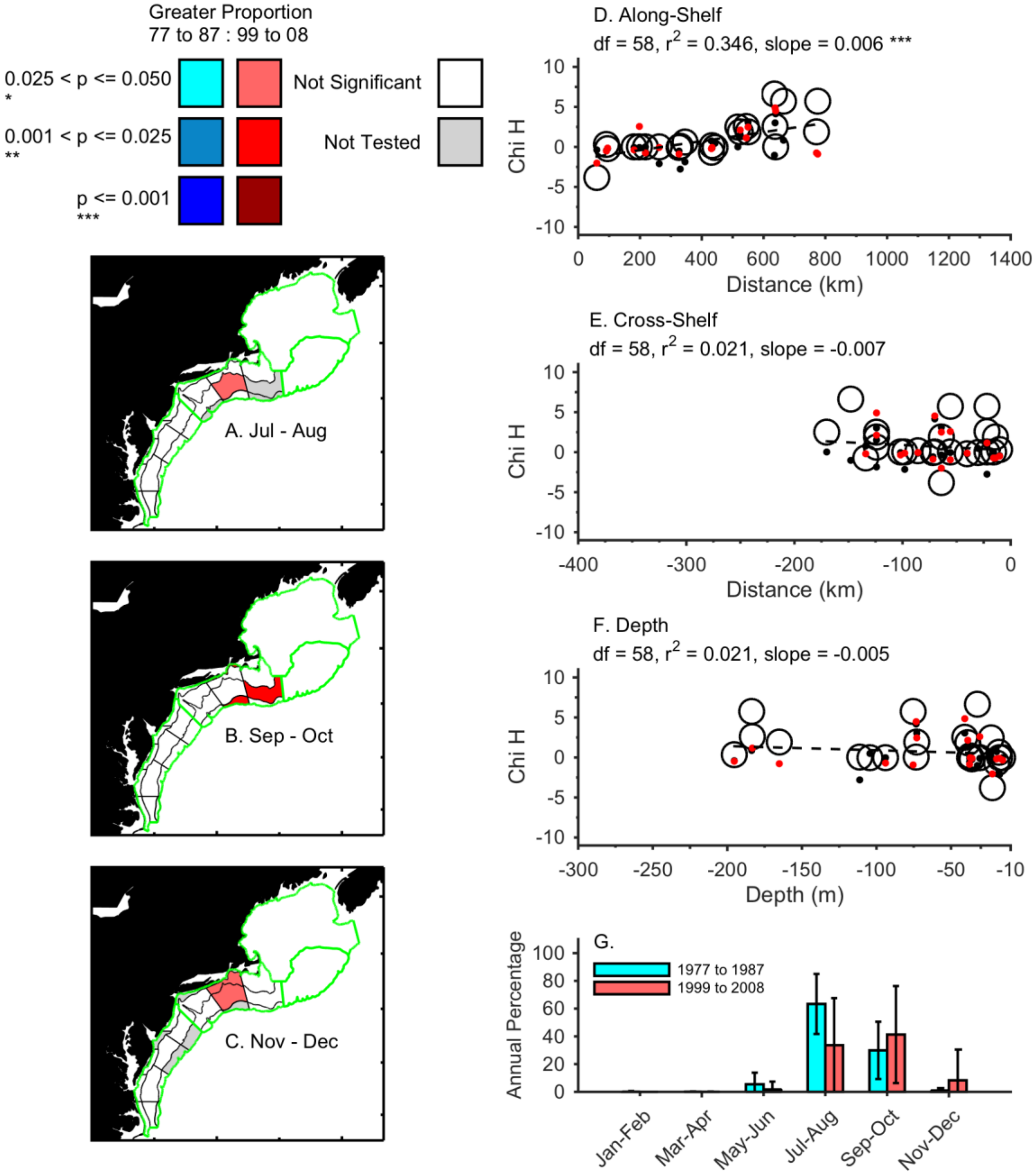

Return to S1 File Table A.

S1 Fig BR. Spatial and seasonal distribution of larval *Paralichthys dentatus* (Summer Flounder) on the Northeast U.S. Shelf Ecosystem. Change in spatial distribution of larval *Paralichthys dentatus* in September - October (A; •), November – December (B; ○), and January – February (C; •) were examined in the along-shelf (D), cross-shelf (E), and depth (F) directions. Larval *Paralichthys dentatus* did not shift significantly spatially or seasonally (G).

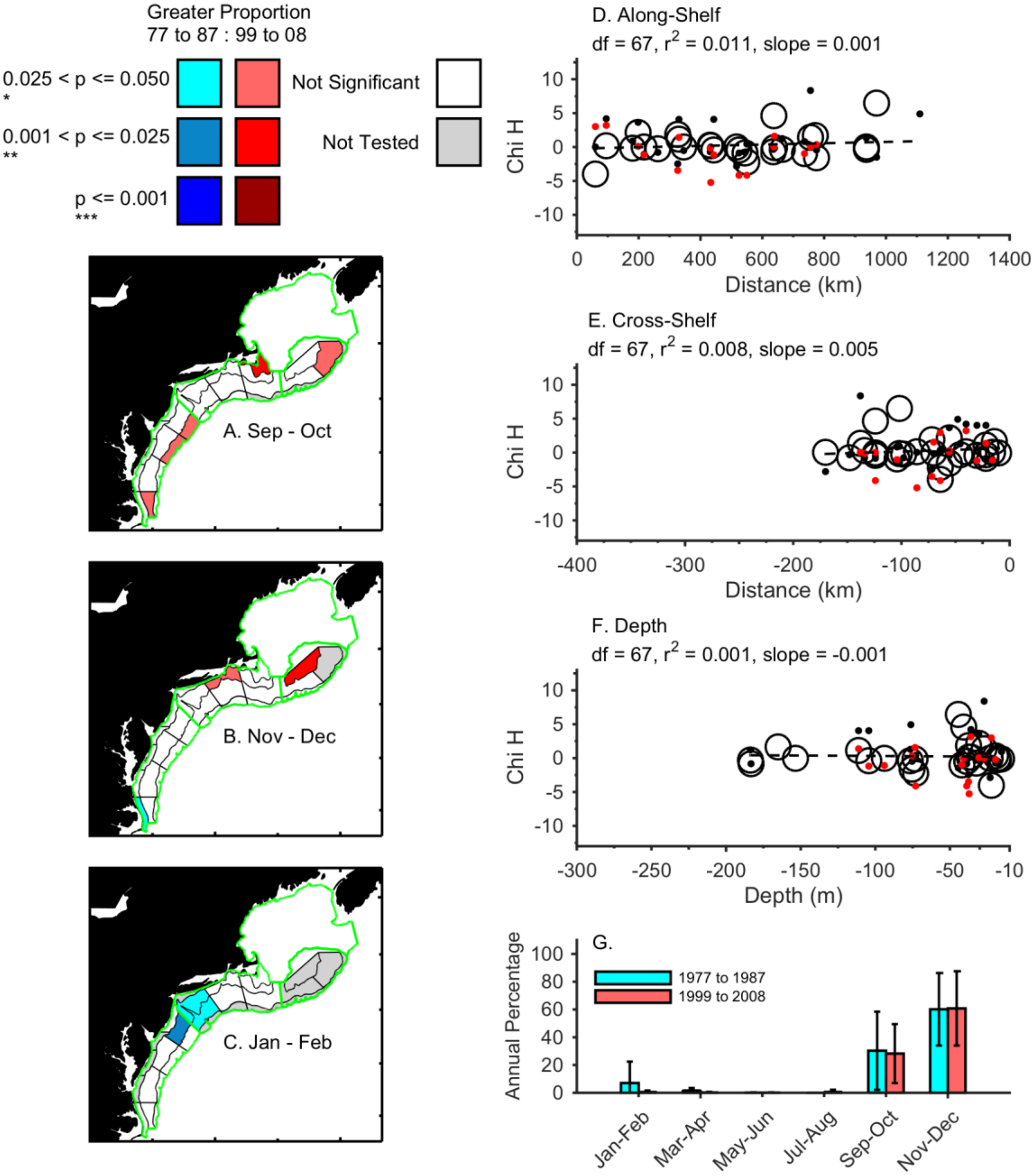

Return to S1 File Table A.

S1 Fig BS. Spatial distribution of adult *Paralichthys dentatus* (Summer Flounder) on the Northeast U.S. Shelf Ecosystem. Change in distribution of adult *Paralichthys dentatus* in the spring (A; ○) and fall (B; •) were examined in the along-shelf (C), cross-shelf (D), and depth (E) directions. *Paralichthys dentatus* shifted significantly northward (C) in the spring and fall.

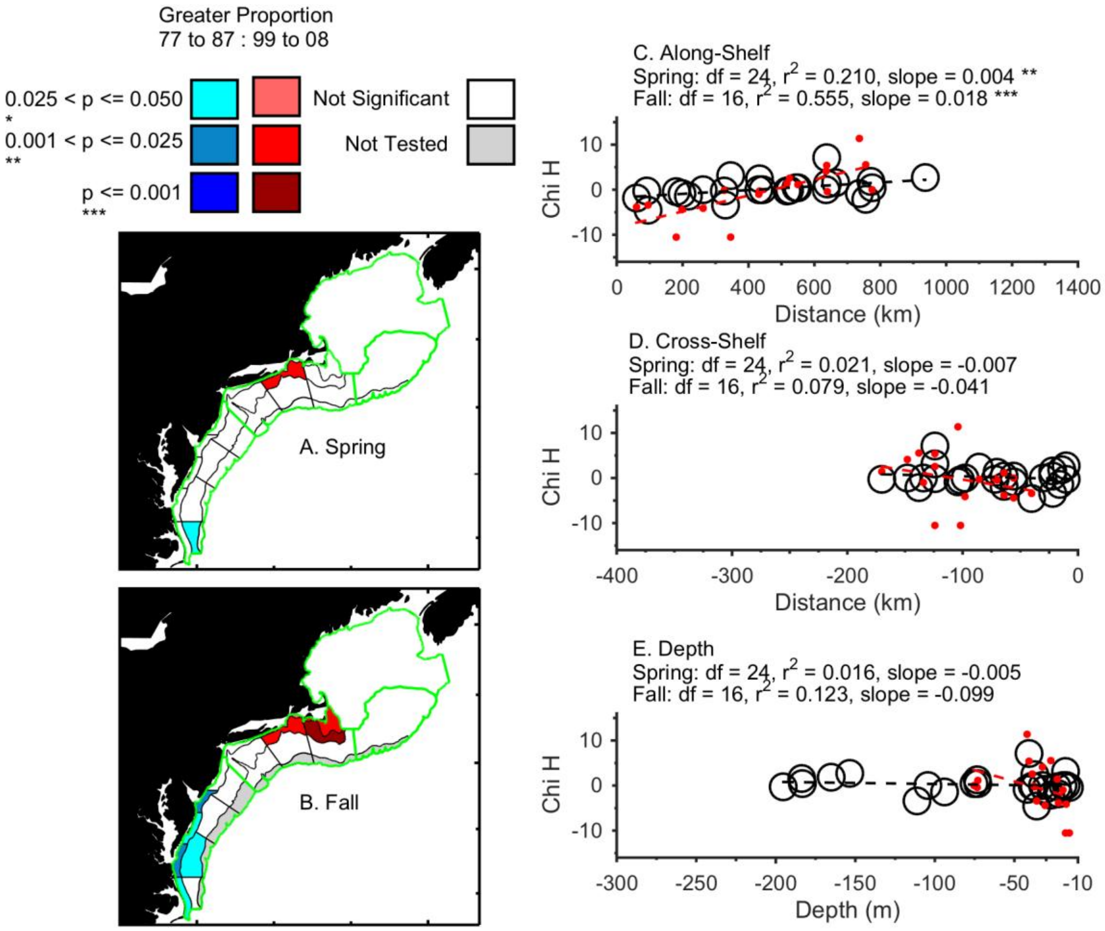

Return to S1 File Table A.

S1 Fig BT. Spatial and seasonal distribution of larval *Hippoglossina oblonga* (American Fourspot Flounder) on the Northeast U.S. Shelf Ecosystem. Change in spatial distribution of larval *Hippoglossina oblonga* in May - June (A; •), July - August (B; ○), and September - October (C; •) were examined in the along-shelf (D), cross-shelf (E), and depth (F) directions. Larval *Hippoglossina oblonga* shifted significantly northward (D) spatially and later seasonally (G).

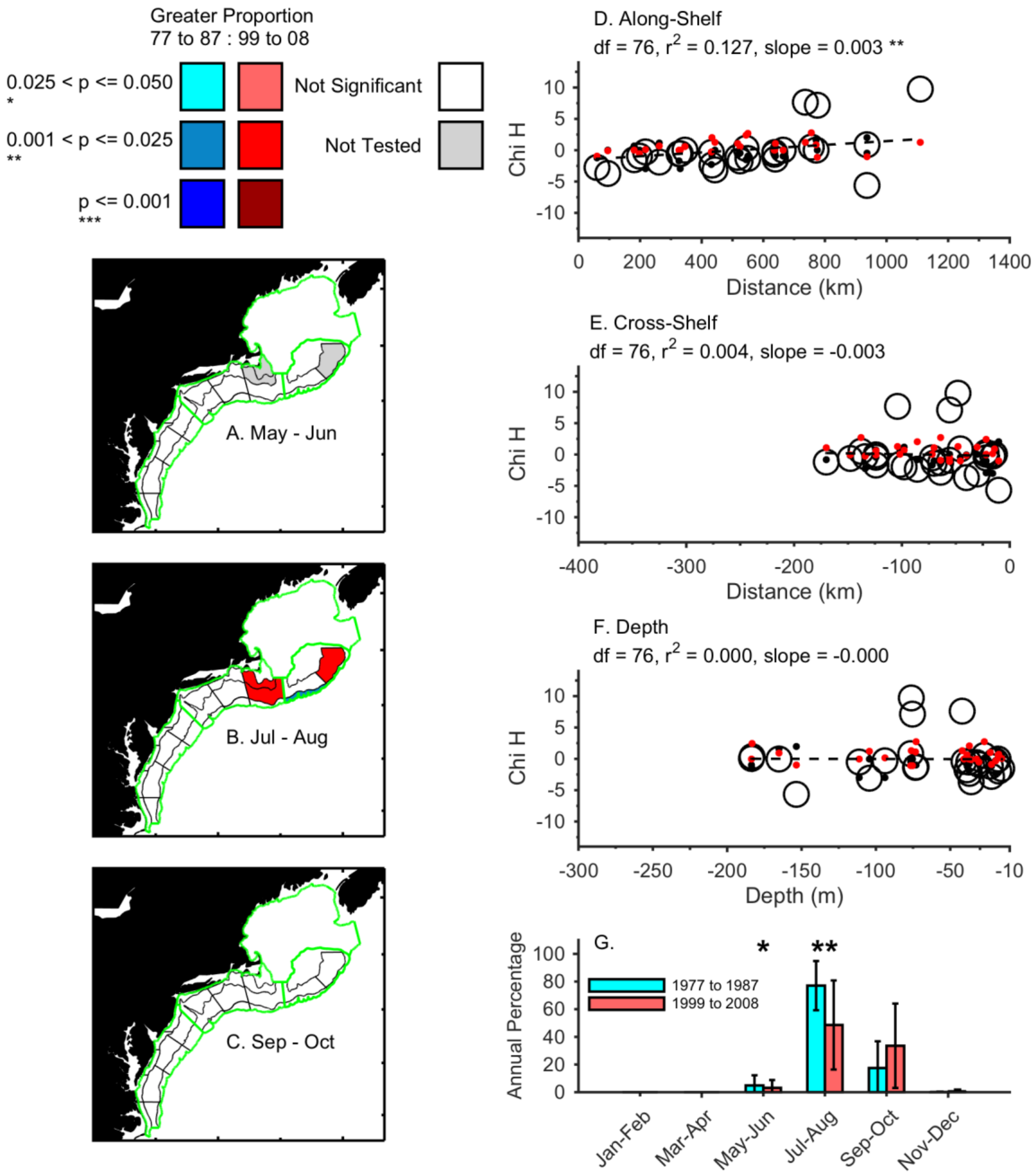

Return to S1 File Table A.

S1 Fig BU. Spatial distribution of adult *Hippoglossina oblonga* (American Fourspot Flounder) on the Northeast U.S. Shelf Ecosystem. Change in distribution of adult *Hippoglossina oblonga* in the spring (A; ○) and fall (B; •) were examined in the along-shelf (C), cross-shelf (D), and depth (E) directions. *Hippoglossina oblonga* distribution shifted significantly deeper (E) in the spring and northward (C) in the fall.

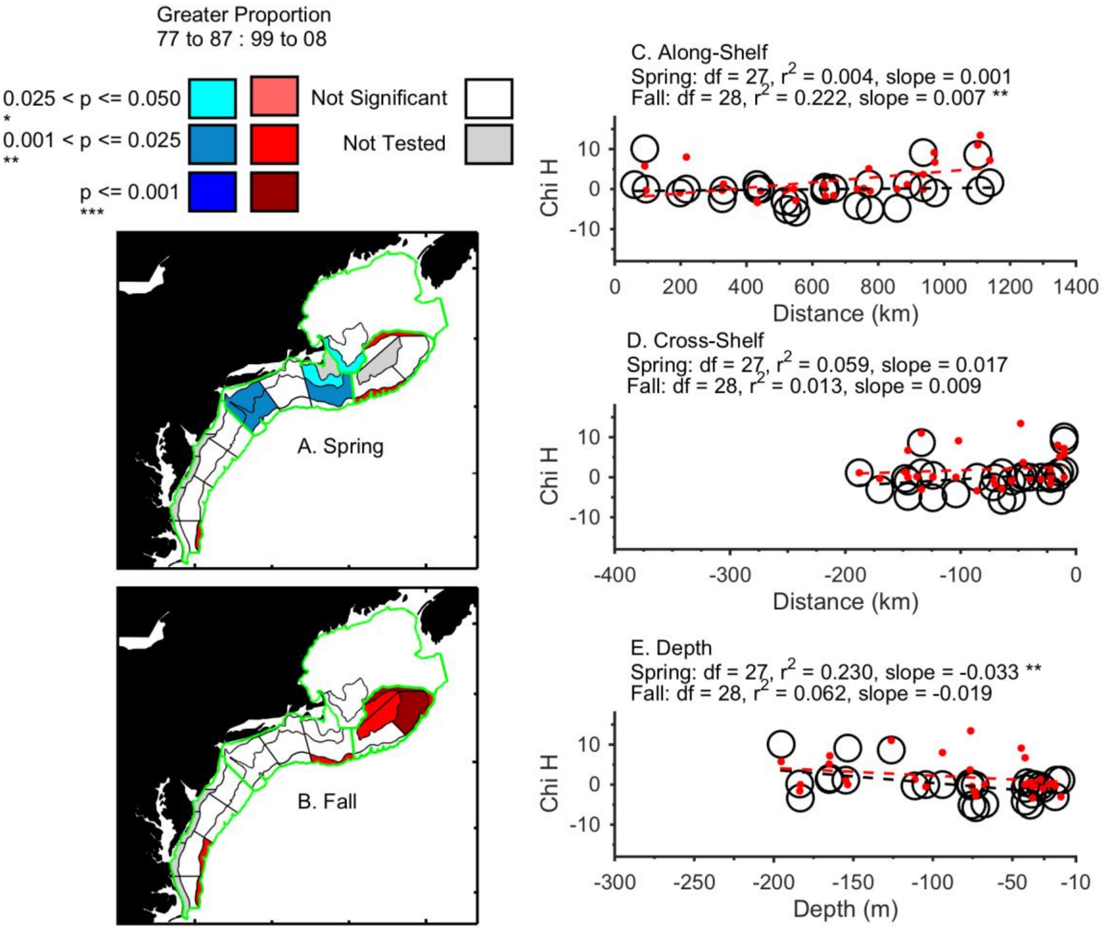

[Return to S1 File Table A](#).

S1 Fig BV. Spatial and seasonal distribution of larval *Syacium* spp. (Dusky Flounders) on the Northeast U.S. Shelf Ecosystem. Change in spatial distribution of larval *Syacium* spp. in May - June (A; •), July - August (B; ○), and September - October (C; •) were examined in the along-shelf (D), cross-shelf (E), and depth (F) directions. Larval *Syacium* spp. did not shift significantly spatially or seasonally (G).

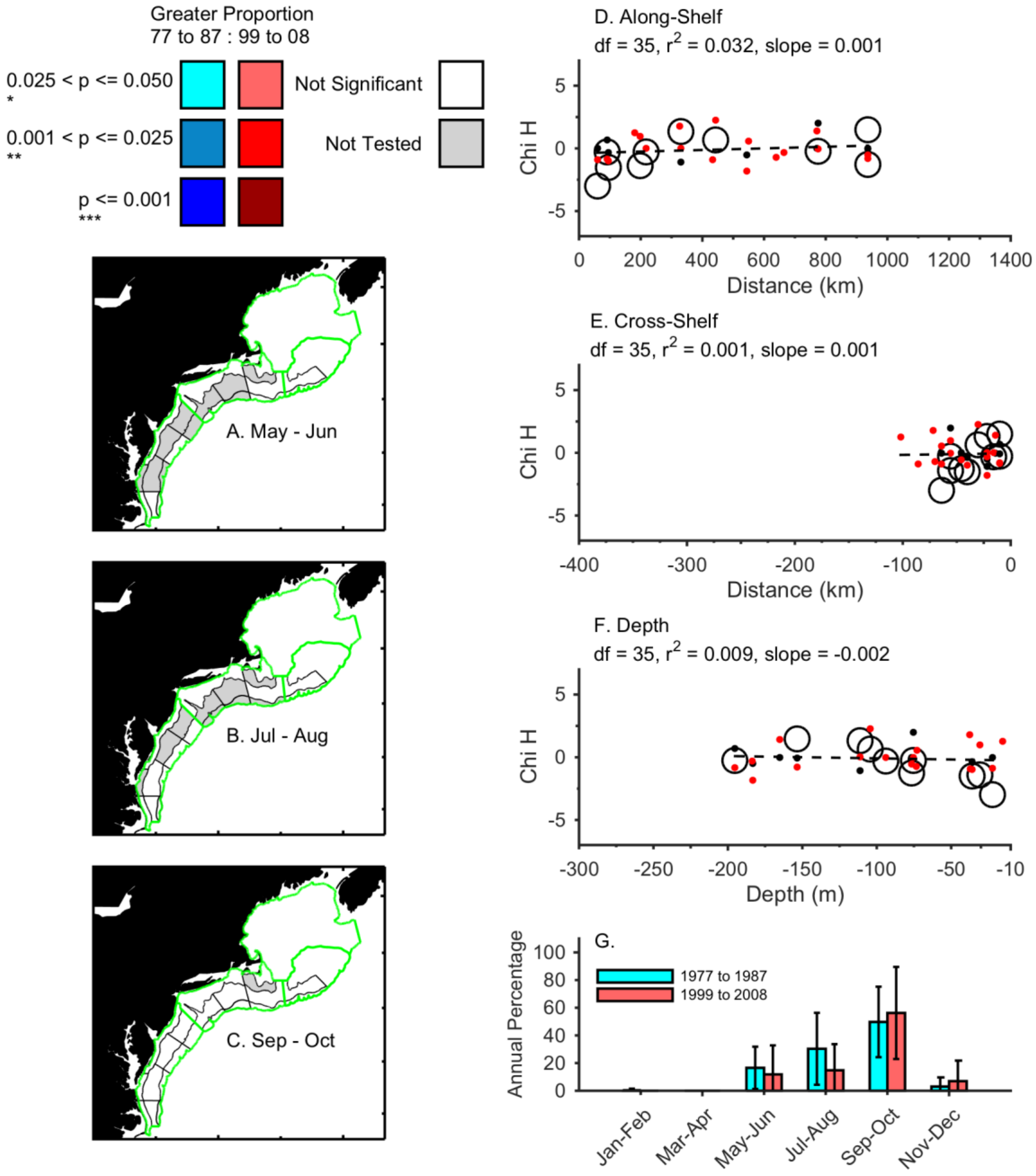

Return to S1 File Table A.

S1 Fig BW. Spatial and seasonal distribution of larval *Bothus* spp. (Eyed Flounders) on the Northeast U.S. Shelf Ecosystem. Change in spatial distribution of larval *Bothus* spp. in July - August (A; •), September - October (B; ○), and November – December (C; •) were examined in the along-shelf (D), cross-shelf (E), and depth (F) directions. Larval *Bothus* spp. did not shift significantly spatially or seasonally (G).

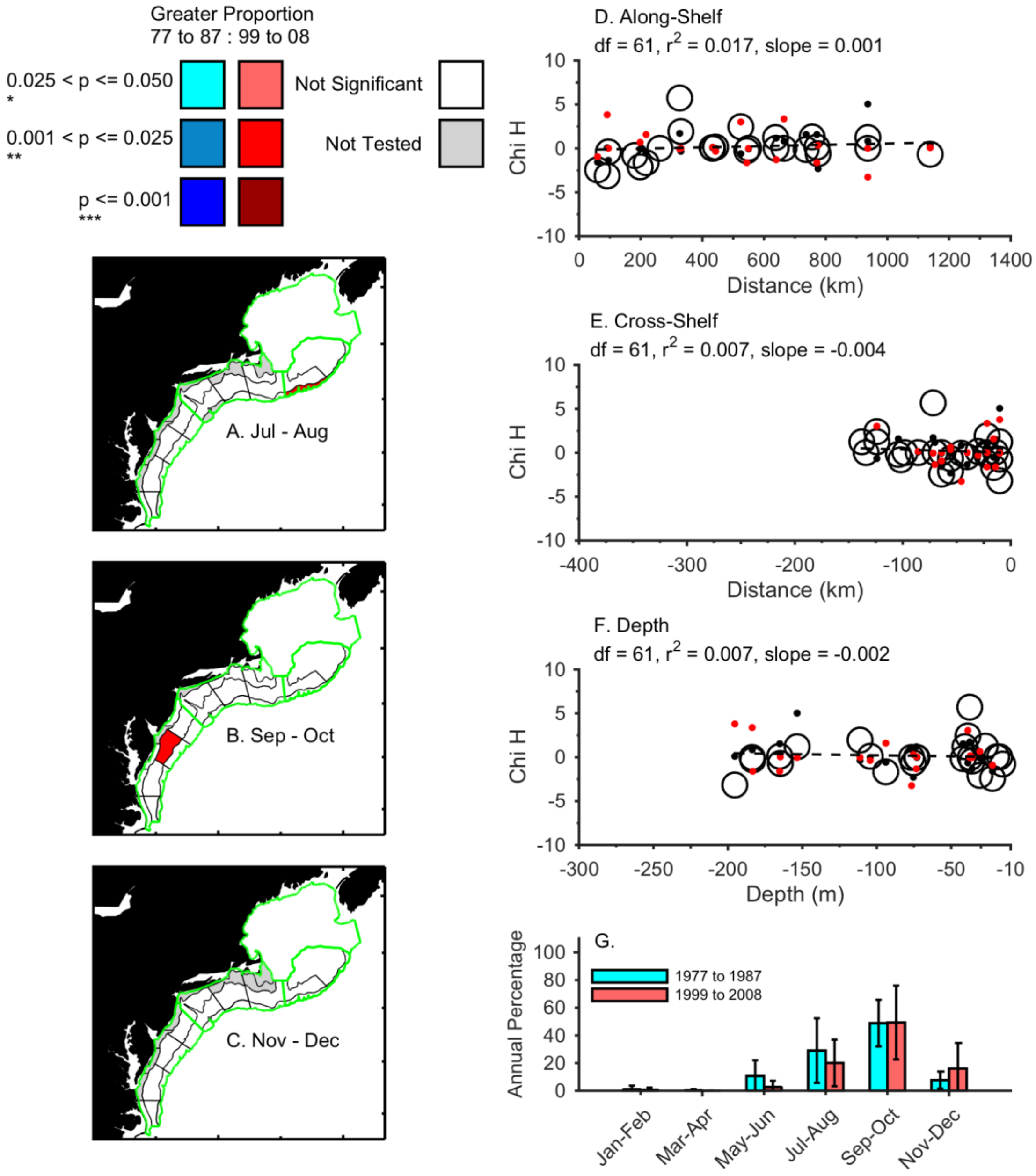

Return to S1 File Table A.

S1 Fig BX. Spatial and seasonal distribution of larval *Glyptocephalus cynoglossus* (Witch Flounder) on the Northeast U.S. Shelf Ecosystem. Change in spatial distribution of larval *Glyptocephalus cynoglossus* in the March – April (A; •), May – June (B; ○), and July – August (C; •) were examined in the along-shelf (D), cross-shelf (E), and depth (F) directions. Larval *Glyptocephalus cynoglossus* did not shift significantly spatially or seasonally (G).

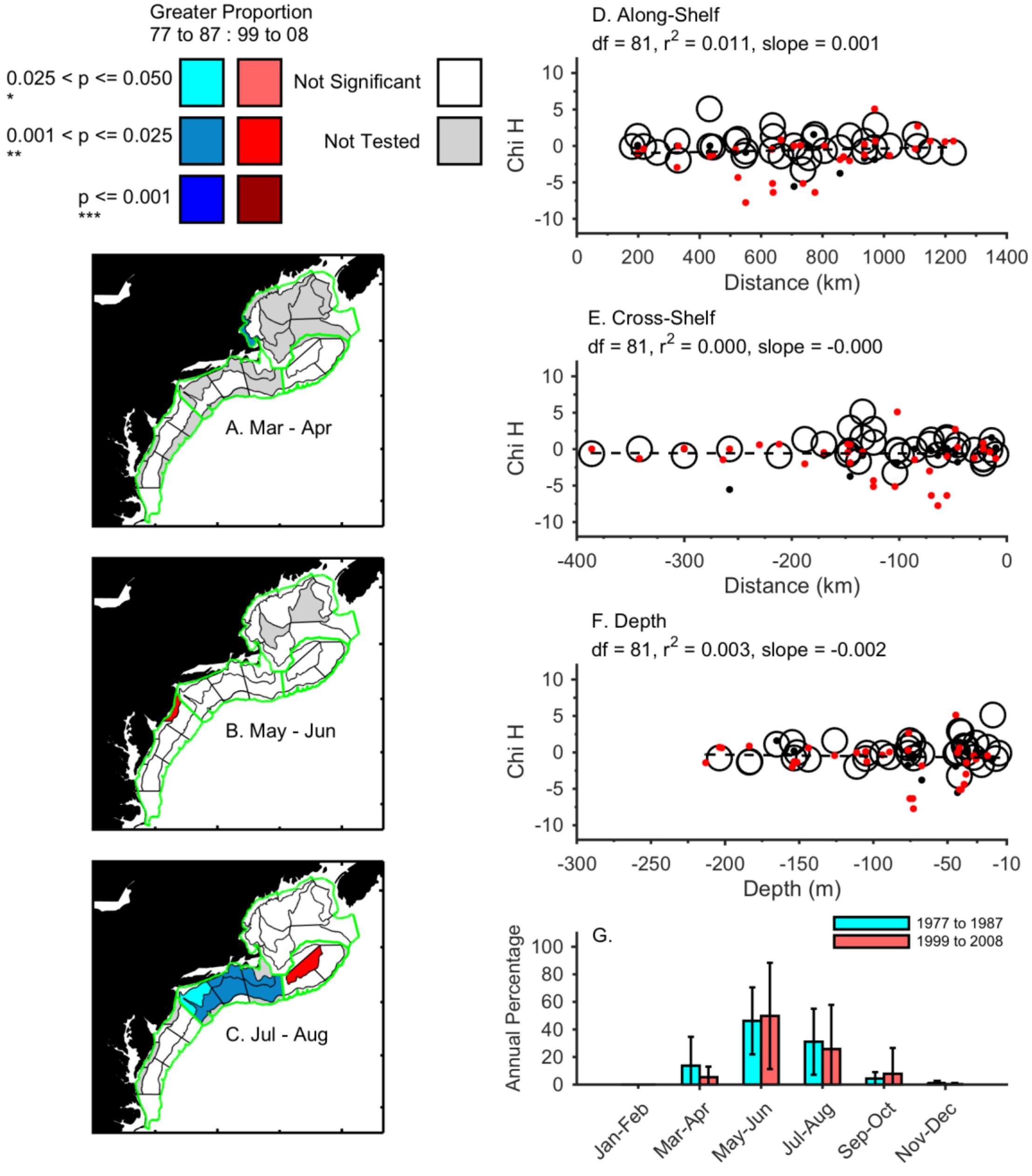

Return to S1 File Table A.

S1 Fig BY. Spatial distribution of adult *Glyptocephalus cynoglossus* (Witch Flounder) on the Northeast U.S. Shelf Ecosystem. Change in distribution of adult *Glyptocephalus cynoglossus* in the spring (A; ○) and fall (B; •) were examined in the along-shelf (C), cross-shelf (D), and depth (E) directions. *Glyptocephalus cynoglossus* shifted significantly deeper (E) in the spring.

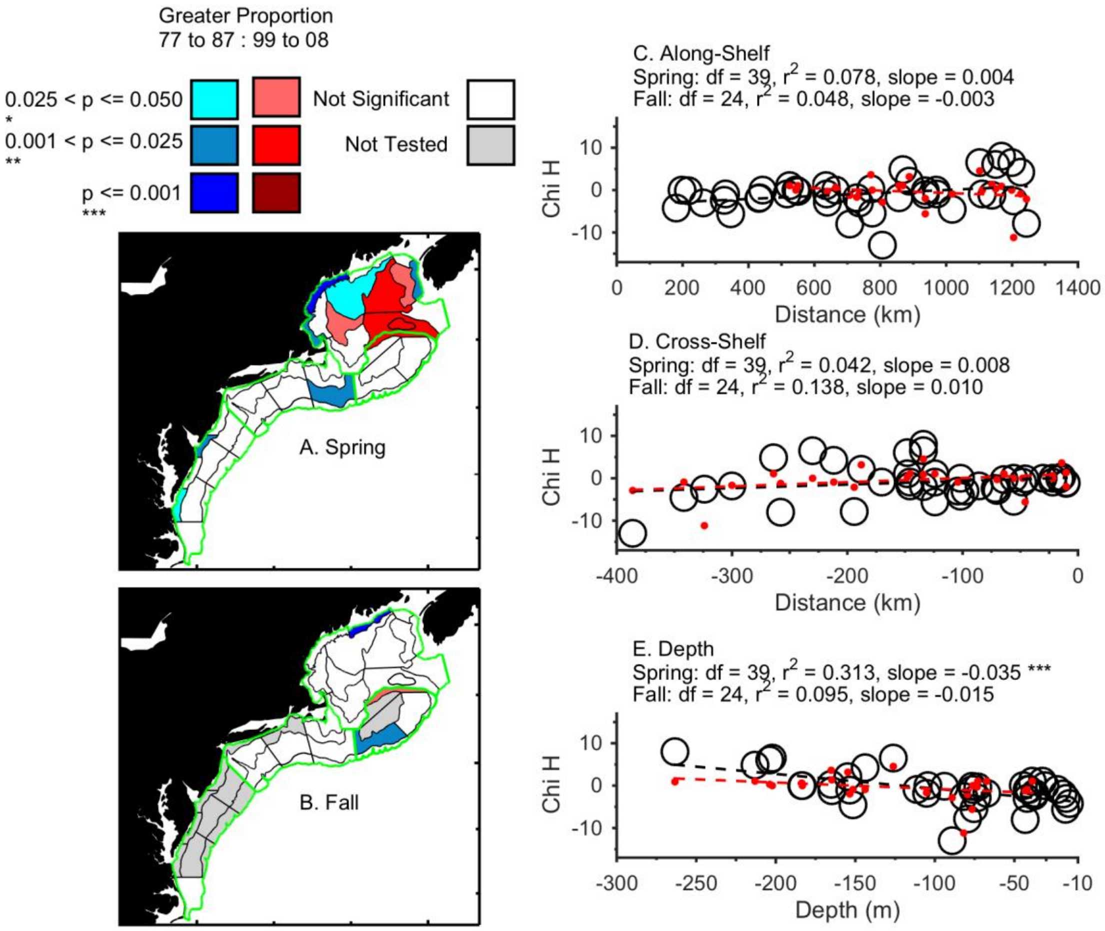

Return to S1 File Table A.

S1 Fig BZ. Spatial and seasonal distribution of larval *Hippoglossoides platessoides* (American Plaice) on the Northeast U.S. Shelf Ecosystem. Change in spatial distribution of larval *Hippoglossoides platessoides* in the March – April (A; •), May – June (B; ○), and July – August (C; •) were examined in the along-shelf (D), cross-shelf (E), and depth (F) directions. Larval *Hippoglossoides platessoides* did not shift significantly spatially or seasonally (G).

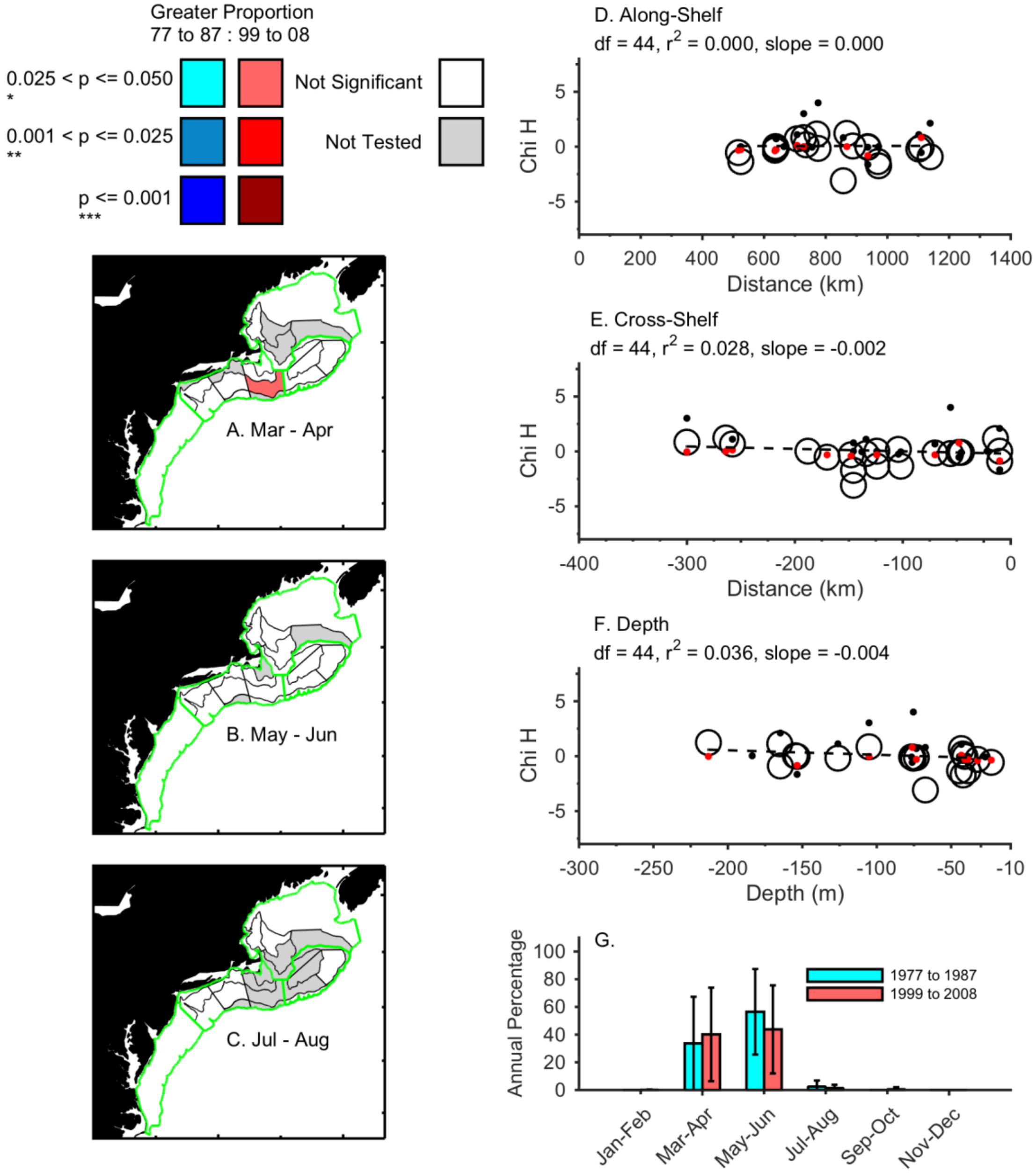

Return to S1 File Table A.

S1 Fig CA. Spatial distribution of adult *Hippoglossoides platessoides* (American Plaice) on the Northeast U.S. Shelf Ecosystem. Change in distribution of adult *Hippoglossoides platessoides* in the spring (A; ○) and fall (B; •) were examined in the along-shelf (C), cross-shelf (D), and depth (E) directions. *Hippoglossoides platessoides* shifted significantly offshore (E) in the spring and fall.

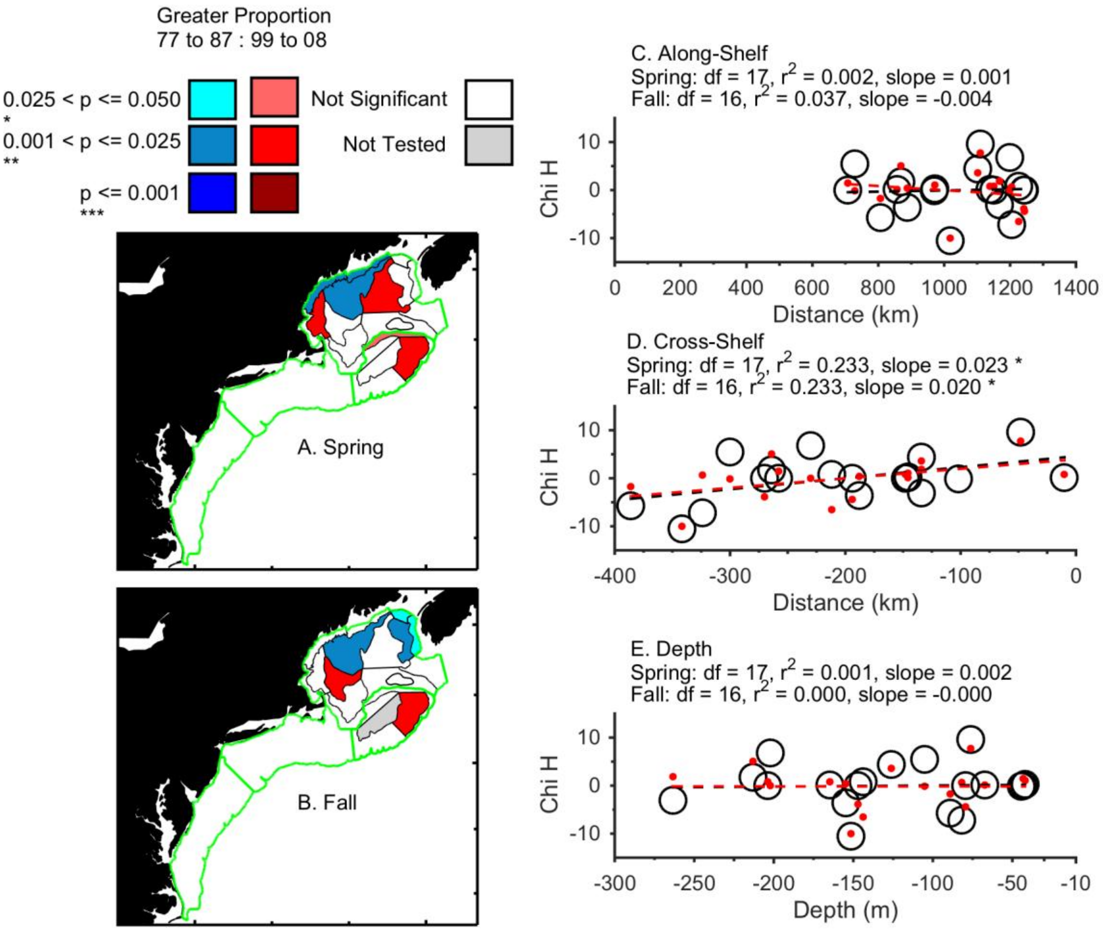

Return to S1 File Table A.

S1 Fig CB. Spatial distribution of adult *Hippoglossus hippoglossus* (Atlantic Halibut) on the Northeast U.S. Shelf Ecosystem. Change in distribution of adult *Hippoglossus hippoglossus* in the spring (A; ○) and fall (B; •) were examined in the along-shelf (C), cross-shelf (D), and depth (E) directions. *Hippoglossus hippoglossus* did not shift significantly.

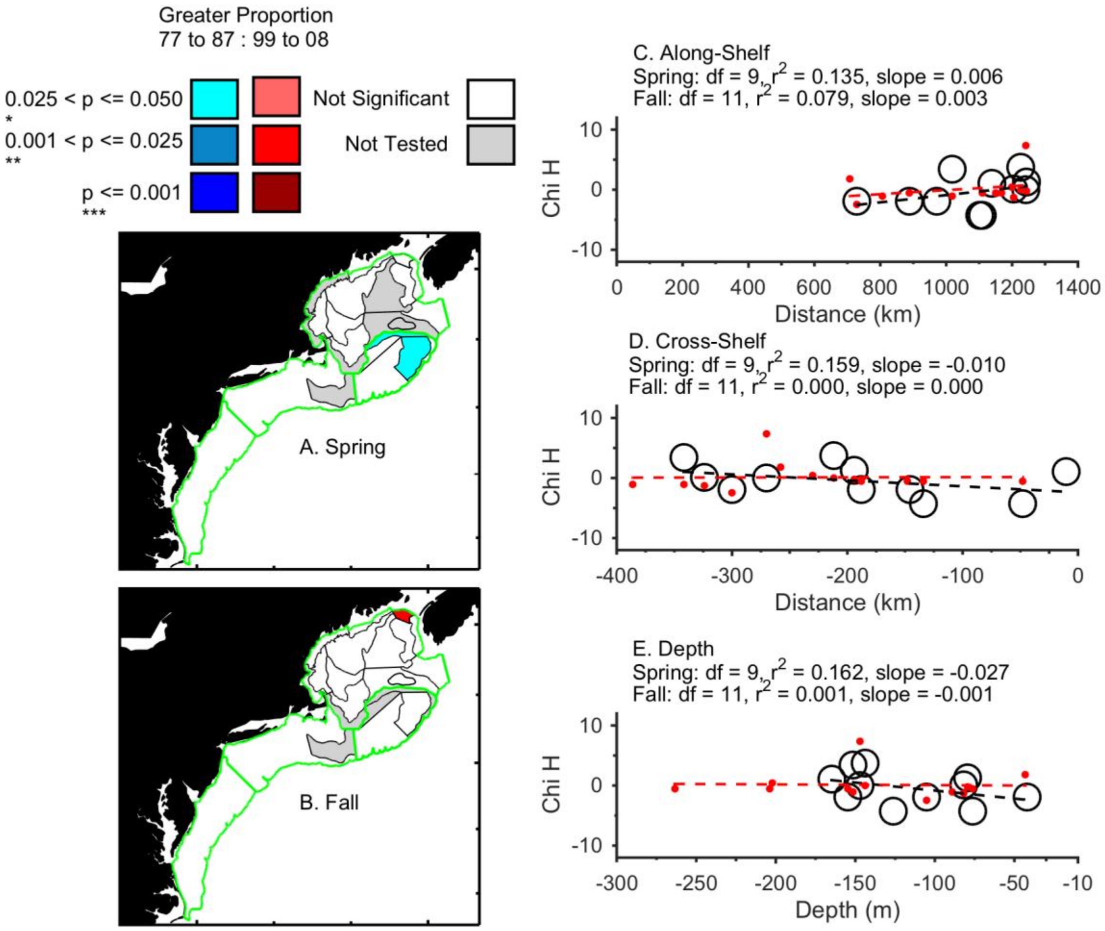

[Return to S1 File Table A].

S1 Fig CC. Spatial and seasonal distribution of larval *Limanda ferruginea* (Yellowtail Flounder) on the Northeast U.S. Shelf Ecosystem. Change in spatial distribution of larval *Limanda ferruginea* in the March – April (A; •), May – June (B; ○), and July – August (C; •) were examined in the along-shelf (D), cross-shelf (E), and depth (F) directions. Larval *Limanda ferruginea* shifted significantly northward (D) spatially and earlier seasonally (G).

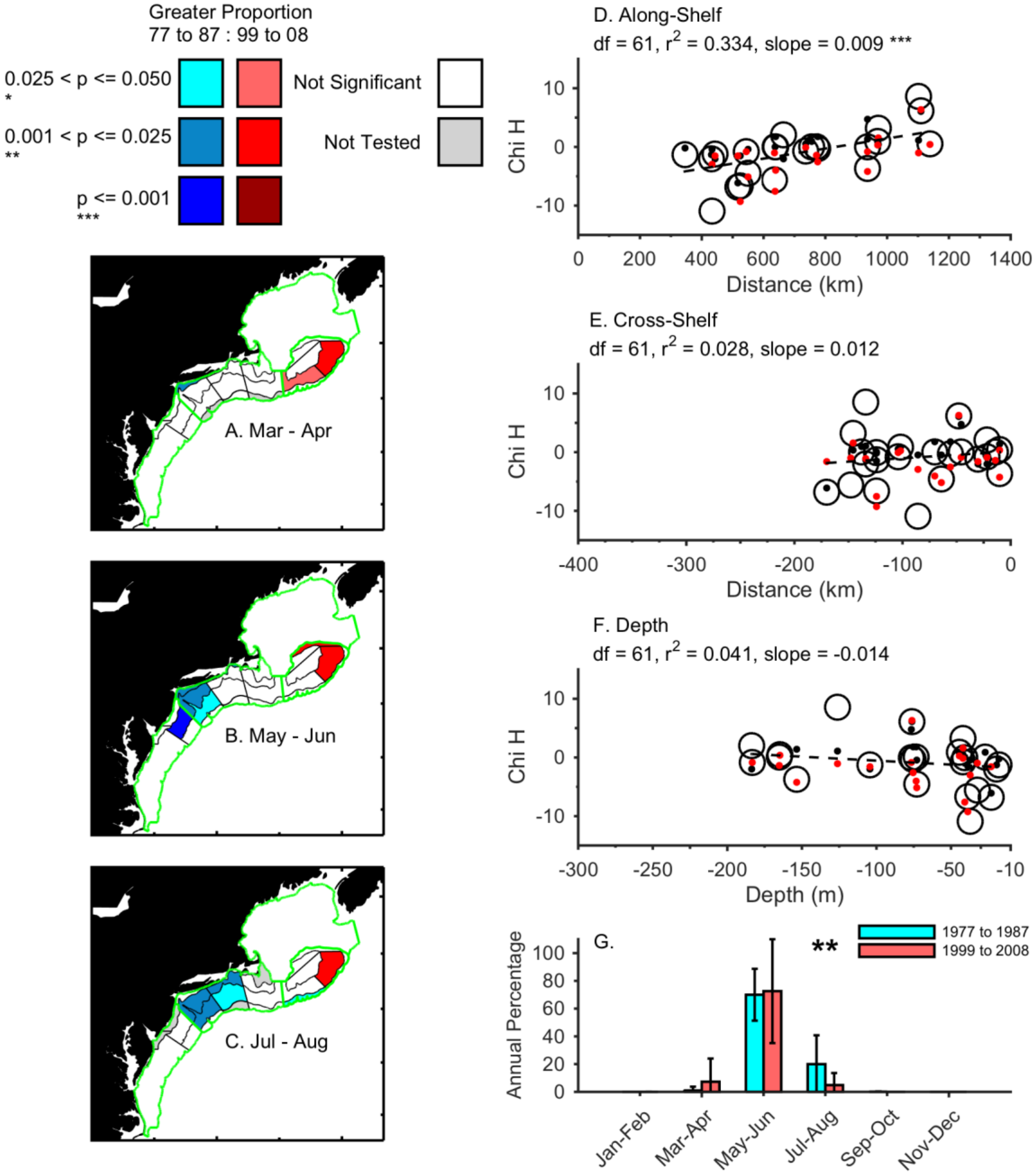

Return to S1 File Table A.

S1 Fig CD. Spatial distribution of adult *Limanda ferruginea* (Yellowtail Flounder) on the Northeast U.S. Shelf Ecosystem. Change in distribution of adult *Limanda ferruginea* in the spring (A; ○) and fall (B; •) were examined in the along-shelf (C), cross-shelf (D), and depth (E) directions. *Limanda ferruginea* shifted significantly northward (C) and deeper (E) in the spring.

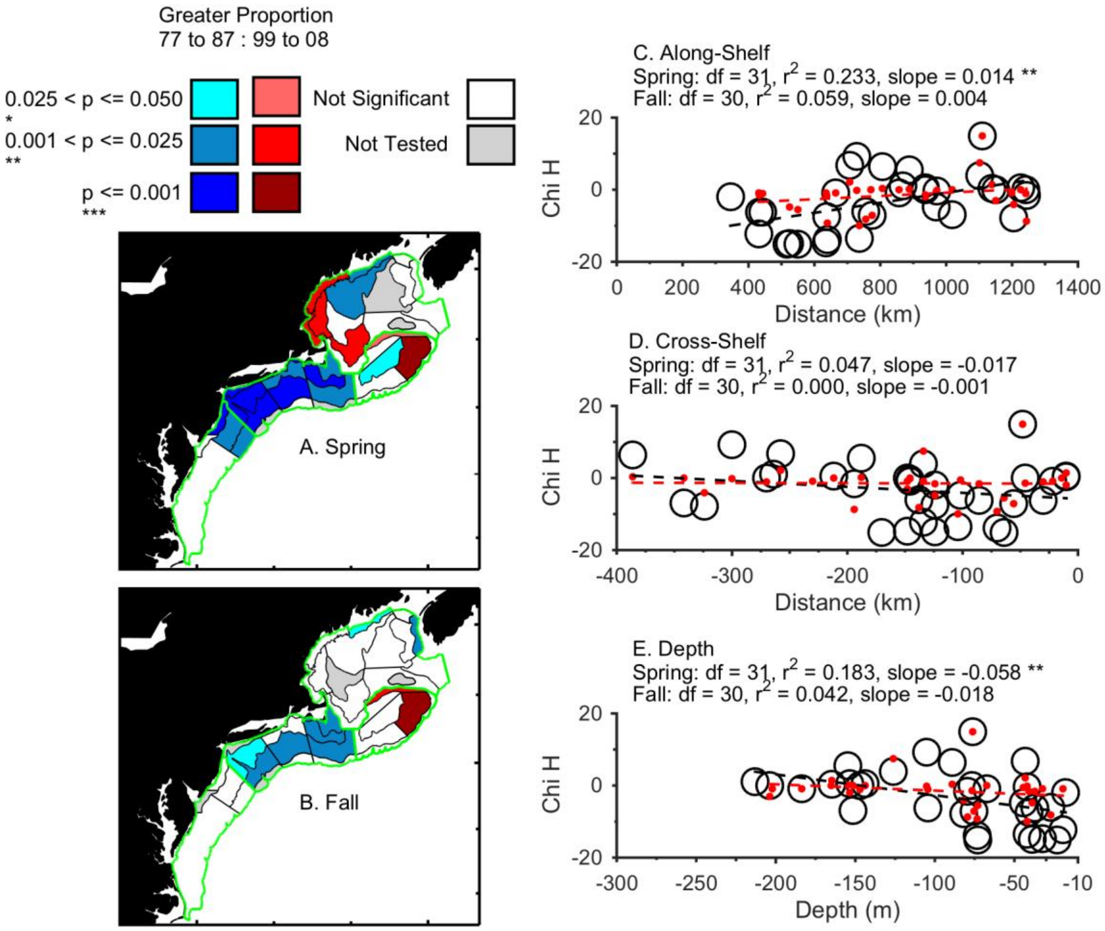

Return to S1 File Table A.

S1 Fig CE. Spatial and seasonal distribution of larval *Pseudopleuronectes americanus* (Winter Flounder) on the Northeast U.S. Shelf Ecosystem. Changes distribution in January – February (A; •), March – April (B; ○), and May – June (C; •) were examined in the along-shelf (D), cross-shelf (E), and depth (F) directions. Larval *Pseudopleuronectes americanus* did not shift significantly spatially. Seasonal distribution shifted significantly earlier (G).

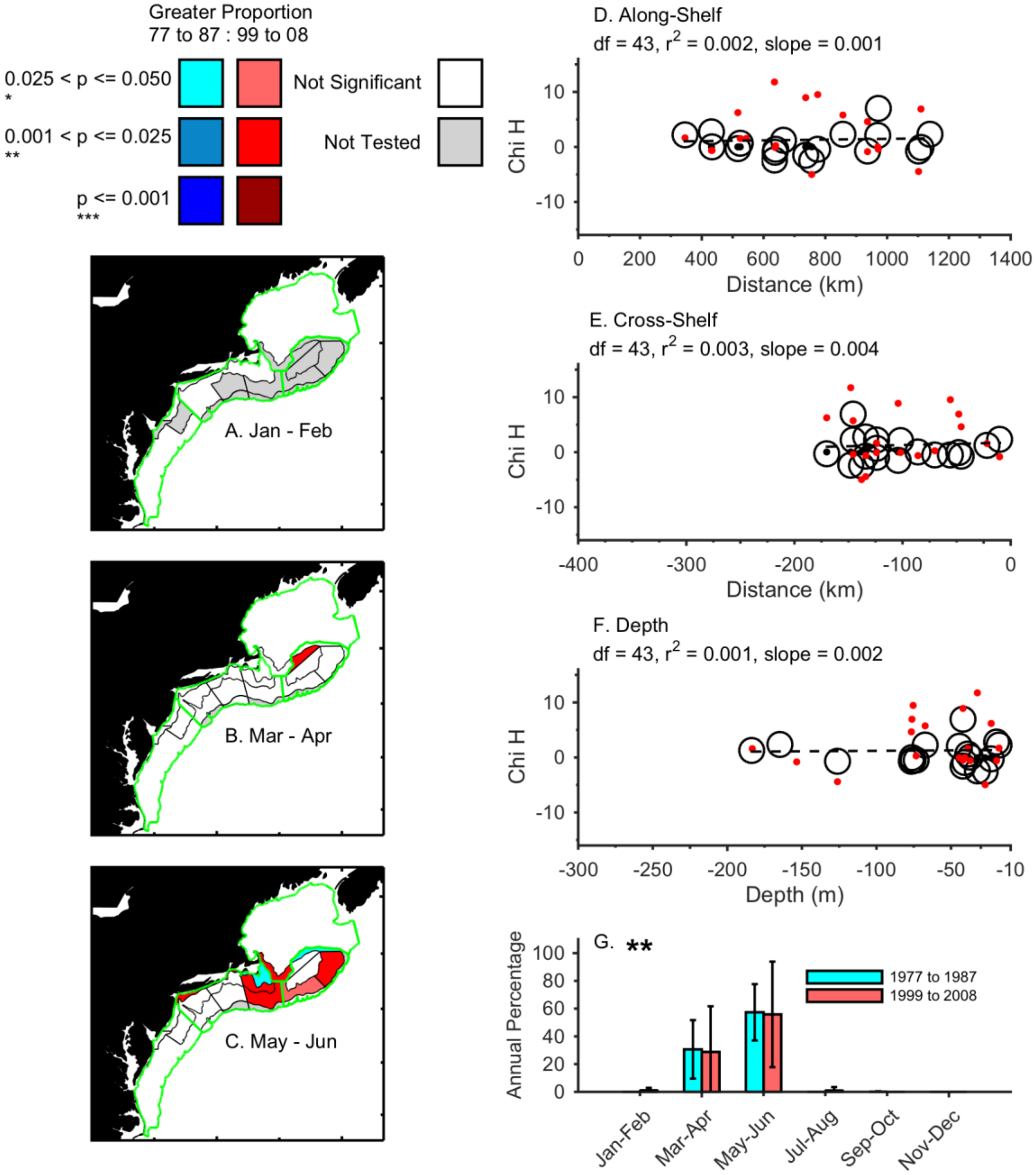

Return to S1 File Table A.

S1 Fig CF. Spatial distribution of adult *Pseudopleuronectes americanus* (Winter Flounder) on the Northeast U.S. Shelf Ecosystem. Change in distribution of adult *Pseudopleuronectes americanus* in the spring (A; ○) and fall (B; •) were examined in the along-shelf (C), cross-shelf (D), and depth (E) directions. *Pseudopleuronectes americanus* did not shift significantly.

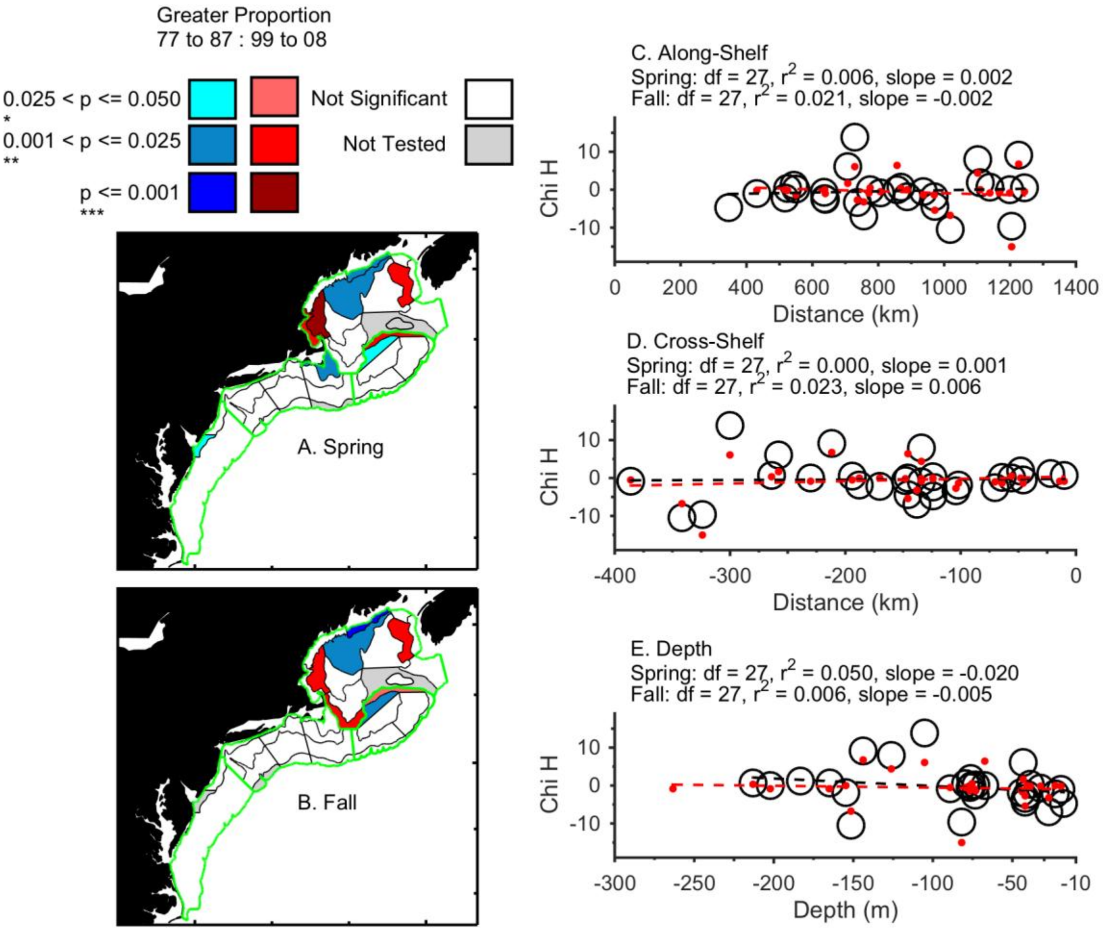

Return to S1 File Table A.

S1 Fig CG. Spatial and seasonal distribution and seasonal occurrence of larval *Symphurus* spp. (Tonguefishes) on the Northeast U.S. Shelf Ecosystem. Change in spatial distribution of larval *Symphurus* spp. in May - June (A; •), July - August (B; ○), and September - October (C; •) were examined in the along-shelf (D), cross-shelf (E), and depth (F) directions. Larval *Symphurus* spp. did not shift significantly spatially. Seasonal distribution shifted significantly later (G).

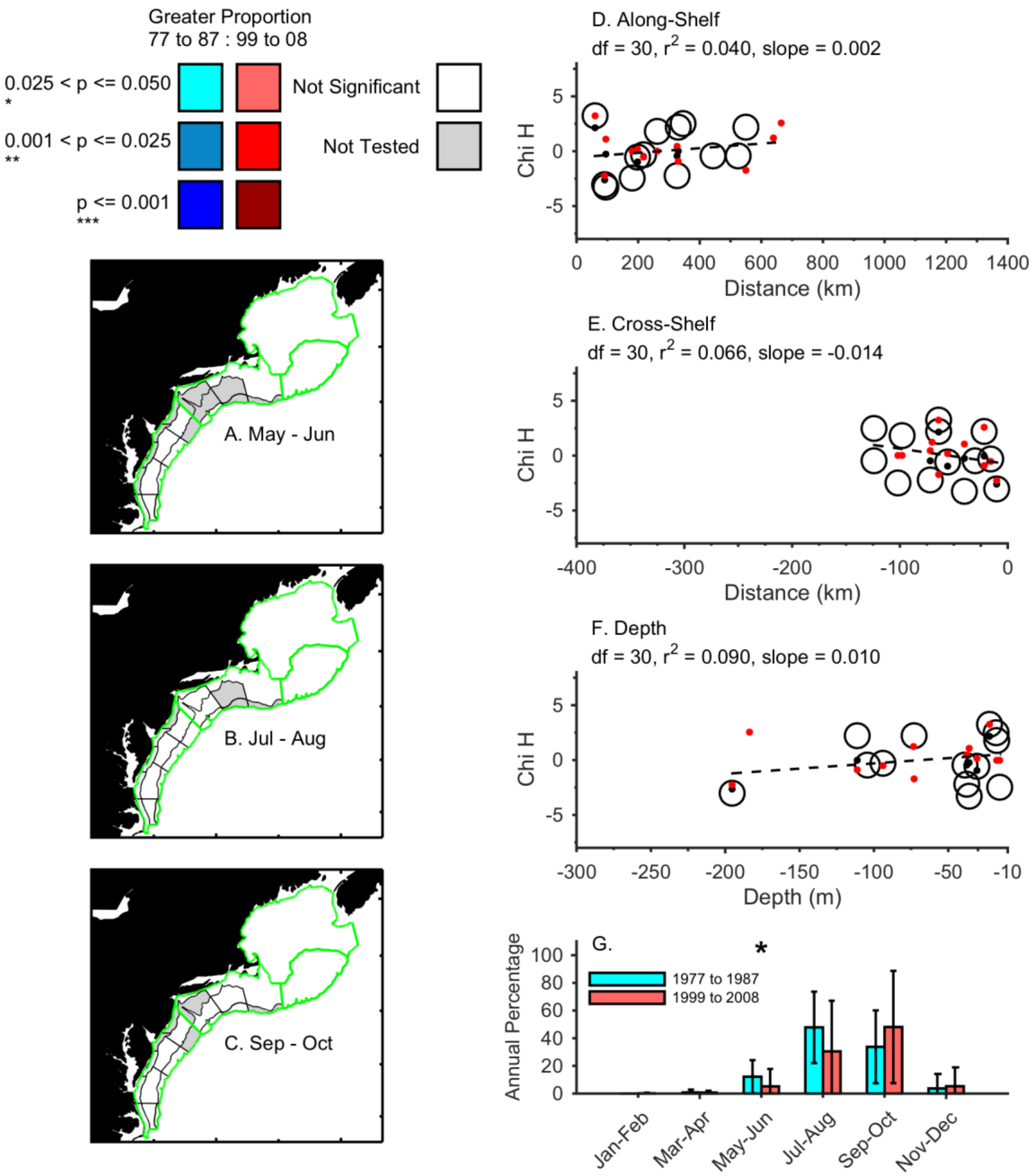

Return to S1 File Table A.
